# Dynamics of Translation of Single mRNA Molecules In Vivo

**DOI:** 10.1016/j.cell.2016.04.034

**Published:** 2016-05-05

**Authors:** Xiaowei Yan, Tim A. Hoek, Ronald D. Vale, Marvin E. Tanenbaum

**Affiliations:** 1 Hubrecht Institute, The Royal Netherlands Academy of Arts and Sciences (KNAW) and University Medical Center Utrecht, Utrecht 3584CT, the Netherlands; 2 Department of Cellular and Molecular Pharmacology, Howard Hughes Medical Institute, University of California, San Francisco, San Francisco, CA 94158-2517, USA

## Abstract

Regulation of mRNA translation, the process by which ribosomes decode mRNAs into polypeptides, is used to tune cellular protein levels. Currently, methods for observing the complete process of translation from single mRNAs in vivo are unavailable. Here, we report the long-term (>1 hr) imaging of single mRNAs undergoing hundreds of rounds of translation in live cells, enabling quantitative measurements of ribosome initiation, elongation, and stalling. This approach reveals a surprising heterogeneity in the translation of individual mRNAs within the same cell, including rapid and reversible transitions between a translating and non-translating state. Applying this method to the cell-cycle gene Emi1, we find strong overall repression of translation initiation by specific 5′ UTR sequences, but individual mRNA molecules in the same cell can exhibit dramatically different translational efficiencies. The ability to observe translation of single mRNA molecules in live cells provides a powerful tool to study translation regulation.

## Highlights

• A new imaging method to study dynamics of translation of single mRNAs in vivo
• Measurements of ribosome initiation and elongation rates on single mRNA molecules
• mRNAs can rapidly switch between a translating and non-translating state
• Substantial heterogeneity in ribosome pausing on the Xbp1/Hac1 pause sequence

## Graphical Abstract

**Figure:**
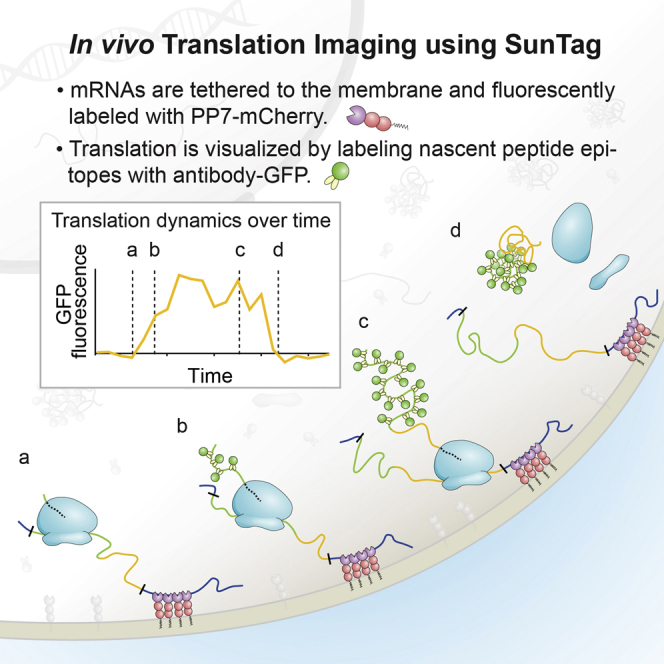

## Teaser

An imaging method is developed for visualizing translation of single mRNA molecules in living cells over long periods of time (>1 hr), revealing a surprising heterogeneity in the translation of mRNA molecules expressed from the same gene in a single cell.

## Introduction

Precise tuning of the expression of each gene in the genome is critical for many aspects of cell function. The level of gene expression is regulated at multiple distinct steps, including transcription, mRNA degradation, and translation (Schwanhäusser et al., 2011). Regulation of all of these steps in gene expression is important, though the relative contribution of each control mechanism varies for different biological processes (Brar et al., 2012, Jovanovic et al., 2015, Peshkin et al., 2015, Tanenbaum et al., 2015, Vardy and Orr-Weaver, 2007).

Measuring the translation rate from individual mRNAs over time provides valuable information on the mechanisms of translation and translational regulation. In vitro experiments, mainly using bacterial ribosomes, have revealed exquisite information on ribosome translocation dynamics at the single molecule level (Blanchard, 2009, Chen et al., 2012, Cornish et al., 2008, Fei et al., 2008, Wen et al., 2008, Zaher and Green, 2009), but such methods have not yet been applied in vivo. In contrast, a genome-wide snapshot of the translational efficiency of endogenous mRNAs in vivo can be obtained through the method of ribosomal profiling (Ingolia et al., 2009, Ingolia et al., 2011). However, this method requires averaging of many cells and provides limited temporal information because of the requirement to lyse cells to make these measurements. Single cell imaging studies have succeeded in measuring average protein synthesis rates (Aakalu et al., 2001, Brittis et al., 2002, Han et al., 2014, Leung et al., 2006, Tanenbaum et al., 2015, Yu et al., 2006), observing the first translation event of an mRNA (Halstead et al., 2015), localizing sub-cellular sites of translation by co-localizing mRNAs and ribosomes (Katz et al., 2016, Wu et al., 2015), and staining nascent polypeptides with small molecule dyes (Rodriguez et al., 2006).

While ribosomal profiling and other recently developed methods have provided many important new insights into the regulation of translation, many questions cannot be addressed using current technologies. For example, it is unclear to what extent different mRNA molecules produced in a single cell from the same gene behave similarly. Many methods to study translation in vivo require averaging of many mRNAs, masking potential differences between individual mRNA molecules. Such differences could arise from differential post-transcriptional regulation, such as nucleotide modifications (Choi et al., 2016, Wang et al., 2015), differential transcript lengths through use of alternative transcriptional start sites (Rojas-Duran and Gilbert, 2012) or polyadenylation site selection (Elkon et al., 2013, Gupta et al., 2014), differences in ribonucleic protein (RNP) composition (Wu et al., 2015), distinct intracellular localization (Hüttelmaier et al., 2005), or different states of RNA secondary structure (Babendure et al., 2006, Kertesz et al., 2010). Heterogeneity among mRNA molecules could have a profound impact on the total amount of polypeptide produced, as well as the localization of protein synthesis, but remains poorly studied. Furthermore, the extent to which translation of single mRNA molecules varies over time is also largely unknown. For example, translation may occur in bursts, rather than continuously (Tatavarty et al., 2012, Yu et al., 2006), and regulation of protein synthesis may occur by modulating burst size and/or frequency, which could occur either globally or on each mRNA molecule individually. In addition, the ability of an mRNA molecule to initiate translation may vary with time or spatial location, for example as cells progress through the cell cycle (Stumpf et al., 2013, Tanenbaum et al., 2015) or undergo active microtubule-based transport to particular cellular destinations (Holt and Schuman, 2013). Such regulation could involve changes in the rates of translation initiation and/or the ribosome elongation. To address these questions, new methods are required for visualizing translation of single mRNA molecules in live cells over time.

Here, we present a method, based on the SunTag fluorescence tagging system that we recently developed (Tanenbaum et al., 2014), for measuring the translation of single mRNA molecules over long periods of time. Using this system, we have measured initiation, elongation, and stalling on individual mRNA molecules and have uncovered unexpected heterogeneity among different mRNA molecules encoded by the same gene within a single cell. Our system will be widely applicable to the study of mRNA translation in live cells.

## Results

### An Assay for Long-Term Observation of Translation of Individual mRNAs

**Figure 1:**
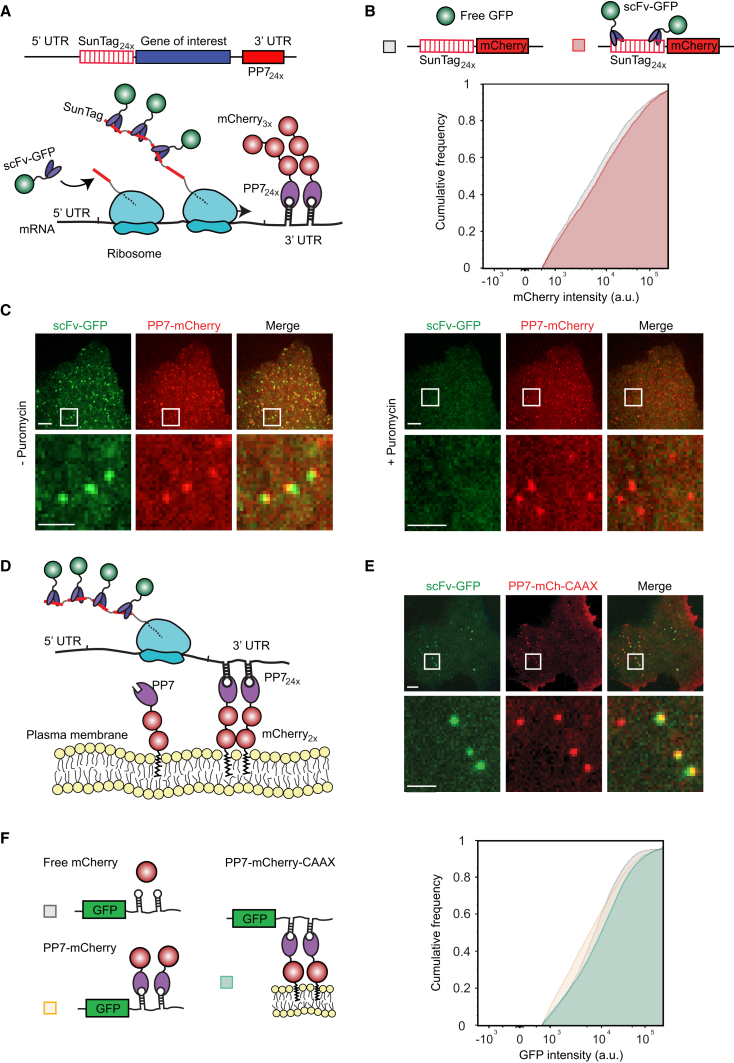
Fluorescence Labeling of Nascent Chains to Visualize Translation of Single mRNA Molecules (A) Schematic of nascent polypeptide labeling using the SunTag system and mRNA labeling (A) and membrane tethering (D) using the PP7 system. (B) A mCherry-SunTag_24x_ reporter gene was co-transfected with either GFP or scFv-GFP, and the expression of the SunTag_24x_-mCherry reporter was determined by FACS (Experimental Procedures). Binding of the scFv-GFP to the SunTag nascent chain did not detectably alter protein expression. (C) A representative U2OS cell is shown expressing scFv-GFP, PP7-3xmCherry, and the translation reporter (SunTag_24x_-Kif18b-PP7_24x_). Cytosolic translation sites (scFv-GFP) co-localize with mRNAs (PP7-3xmCherry). Ribosomes were dissociated from mRNA by addition of puromycin (right panel). Note that translation sites and mRNA do not perfectly overlap because of the brief time difference in acquiring GFP and mCherry images. (D) Schematic of nascent polypeptide labeling and membrane tethering of the mRNA using the PP7 system. (E) U2OS cells expressing scFv-GFP (green), PP7-2xmCherry-CAAX (red), and the translation reporter (SunTag_24x_-Kif18b-PP7_24x_). A single time point of the cell (top panel) and a zoomed-in view from the white-boxed area containing a few mRNAs (lower) are shown. (F) U2OS cells were transfected with mCherry, PP7-mCherry, or PP7-mCherry-CAAX together with a GFP reporter transcript with 24 PP7 binding sites in the 3′ UTR, and GFP expression was analyzed by FACS (Experimental Procedures). Cumulative distribution of GFP expression levels from GFP-mCherry double positive cells are shown in (B) and (F) (n = 3 independent experiments). Scale bars, 5 μm (upper) and 2 μm (lower). See also Figure S1 and Movie S1. Visualizing Translation of Single mRNAs in Live Cells, Related to Figure 1, Movie S2. GFP Foci Represent Sites of Translation, Related to Figure 1, Movie S3. Visualizing Translation of Membrane-Tethered mRNAs, Related to Figure 1.

Observing the synthesis of a genetically encoded fluorescent protein, such as GFP, in vivo is difficult because of the relatively long maturation time required to achieve a fluorescent state. Thus, a GFP-fusion protein typically will not fluoresce until after its translation is completed. To overcome this temporal challenge and to create a sufficiently bright signal to observe protein synthesis from single mRNAs in vivo, we used our recently developed SunTag system (Tanenbaum et al., 2014). In this assay, cells are co-transfected with a reporter transcript containing an array of 24 SunTag peptides followed by a gene of interest, along with a second construct expressing a GFP-tagged single-chain intracellular antibody (scFv-GFP) that binds to the SunTag peptide with high affinity. As the SunTag peptides are translated and emerge from the ribosome exit tunnel, they are rapidly bound by the soluble and already fluorescent scFv-GFP (Figure 1A). Importantly, labeling of nascent chains using the SunTag antibody did not detectably alter protein synthesis rates of a reporter mRNA in human U2OS cells, as determined by FACS (fluorescence-activated cell sorting) analysis (Figure 1B). At the same time, the mRNA was fluorescently labeled by introducing 24 copies of a short hairpin sequence into the 3′ UTR and co-expressing the PP7 bacteriophage coat protein (Chao et al., 2008), which binds with high affinity to the hairpin sequence, fused to three copies of mCherry (PP7-mCherry) (Figure 1A).

When observed by spinning disk confocal microscopy, the co-expression of a reporter construct (SunTag_24x_-Kif18b-PP7_24x_, with Kif18b being a kinesin motor with a 2.5 kb coding sequence; Tanenbaum et al., 2011), scFv-GFP and PP7-mCherry, resulted in the appearance of a small number (10–50) of very bright green and red fluorescent spots per cell that co-migrated in time-lapse movies (Figure 1C; Movie S1). Spot tracking revealed that these spots diffused with a diffusion coefficient of 0.047 μm^2^/s, which is slightly slower than previous measurements of mRNA diffusion (0.1–0.4 μm^2^/s) (Katz et al., 2016), consistent with the fact that our reporter mRNA contains a larger open reading frame (4.4 kb versus 1.1 kb) and thus more associated ribosomes. In addition, we observed many dim GFP spots that did not co-migrate with an mCherry signal in time-lapse movies. The bright spots rapidly disappeared upon terminating translation by addition of a protein synthesis inhibitor, puromycin, which dissociates nascent polypeptides and ribosomes from mRNA (Figure 1C; Movie S2), indicating that they are sites of active translation where multiple ribosomes are engaged on a single mRNA molecule. The dim spots were unaffected by puromycin treatment, suggesting that they represent individual, fully synthesized SunTag_24x_-Kif18b proteins that had already been released from the ribosome. Thus, this translation imaging assay allows visualization of ongoing translation of single mRNA molecules.

Rapid 3D diffusion of mRNAs makes it difficult to track single mRNAs for >1 min, as mRNAs continuously diffuse in and out of the z-plane of observation, and mRNAs regularly cross paths, complicating identification and tracking of individual mRNA molecules over time. To track mRNAs unambiguously for long periods of time, we added a CAAX sequence, a prenylation sequence that gets inserted into the inner leaflet of the plasma membrane, to the PP7-mCherry protein that served to tether mRNAs to the 2D plane of the plasma membrane (Figures 1D and 1E). As a result of many PP7-mCherry molecules clustering through their interaction with the multiple recognition sites on a single mRNA, bright red dots appeared on the plasma membrane at the bottom of the cell, representing a tethered mRNA molecule (Figure 1E). Tethered mRNA molecules co-migrated with scFv-GFP foci, indicating that they are sites of active translation (Figure 1E; Movie S3). Membrane tethering of the mRNA had minimal effects on the protein expression of a GFP reporter construct as analyzed by FACS (Figure 1F). While membrane tethering greatly improves the ability to visualize translation on single mRNA molecules over long periods of time and does not appear to grossly perturb mRNA translation, it is important to note that some aspects of translation, especially localized translation, may be altered due to tethering (Discussion).

**Figure S1:**
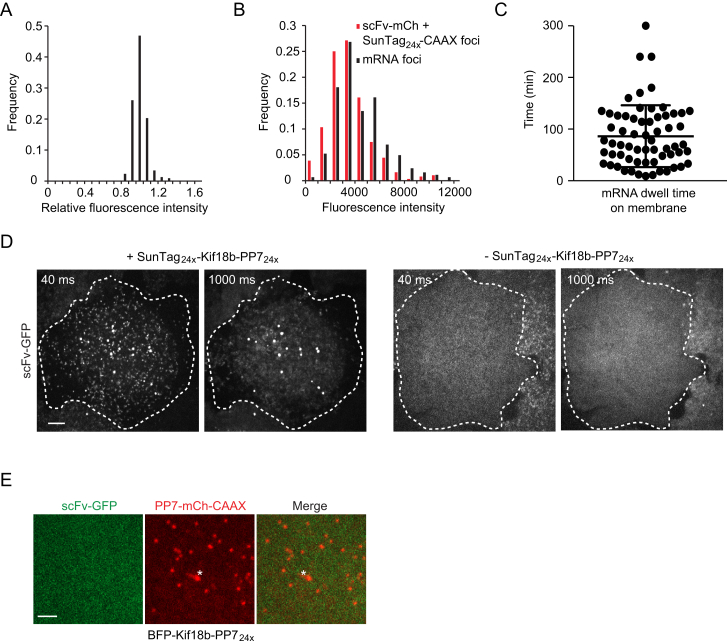
Validation of Single Molecule Translation Visualization Assay, Related to Figure 1 (A–C) U2OS cells expressing scFv-GFP, PP7-2xmCherry-CAAX and the SunTag_24x_-Kif18b-PP7_24x_ translation reporter. (A) Fluorescence intensity of mRNA foci was measured and was corrected for background fluorescence. The average corrected mRNA fluorescence intensity was set to 1 for each separate cell (n = 3 experiments, 14 cells, 278 mRNAs). (B) Intensity of single mRNA foci was measured and corrected for background, but intensity was not normalized as in (A) to allow comparison of absolute intensities (Black bars, n = 3 independent experiments, 22 cells, 377 mRNAs). In parallel, U2OS cells co-expressing SunTag_24x_–CAAX and scFv-mCherry were imaged and the intensities of single membrane bound scFv-mCherry-SunTag_24x_ foci was measured (Red bars, n = 4 independent experiments, 24 cells, 162 mRNAs). (C) Dwell time of tethered mRNAs on the membrane. The time between mRNA appearance at the focal plane of the membrane and its disappearance was scored. mRNA disappearance was due to mRNA detachment or degradation, not photobleaching. Mean and SD are indicated. (D) Cells expressing scFv-GFP with (left two images) or without (right two images) the SunTag_24x_-Kif18b-PP7_24x_ reporter were imaged with indicated exposure time. Dotted line shows outline of the cell. (E) U2OS cells expressing scFv-GFP, PP7-2xmCherry-CAAX and the BFP-Kif18b-PP7_24x_ translation reporter. Representative image is shown. Asterisk indicates lysosome. Scale bars are 5 μm (D) and 2 μm (E).

We first analyzed the PP7-mCherry spots observed on the plasma membrane to confirm that they contained only a single mRNA molecule. The fluorescence intensities of PP7-mCherry foci were very homogeneous (Figure S1A). Their absolute intensity was ∼1.4-fold brighter, on average, than single, membrane-tethered SunTag_24x_-CAAX proteins bound with scFv-mCherry, which is expected to contain 24 mCherry molecules (Figure S1B). PP7 binds as a dimer to the RNA hairpin, and each PP7 was tagged with two tandem copies of mCherry. Thus, mRNAs’ spots could be expected to be four times as bright as single scFv-mCherry-SunTag_24x_-CAAX spots, but previous studies suggested that only about half of PP7 binding sites may be occupied (Wu et al., 2015); thus, mRNA spots would be about 2-fold brighter than single mCherry-SunTag_24x_ spots if they contain a single mRNA molecule but ≥4-fold brighter if they contained two or more mRNAs. These results are therefore most consistent with the mCherry-PP7 foci being single mRNA molecules rather than multiple copies of mRNAs. Further supporting this idea, we tracked 63 single mRNA foci for 30–45 min and did not find a single case in which one spot split into two, which would have been indicative of more than one mRNA molecule being present in a single spot.

Because single mRNAs were tethered to the plasma membrane through multiple PP7 molecules and thus through many CAAX membrane insertion domains, the 2D diffusion of mRNAs was extremely slow (1.06 × 10^−3^ μm^2^/s, n = 211 mRNAs). This slow diffusion made it possible to track individual mRNAs and their associated translation sites for extended periods of time (mean tracking time >1 hr) (Figure S1C). Furthermore, the very slow diffusion rate of tethered mRNAs allowed us to image tethered translation sites using long exposure times (500–1000 ms). During this time interval, rapidly diffusing, non-tethered fully synthesized polypeptides only produced a blurred, diffuse image on the camera sensor, which enabled sites of translation to be easily distinguished from fully synthesized molecules (Figure S1D). Finally, to confirm that the scFv-GFP was binding to nascent SunTag peptides, we replaced the SunTag epitope peptides in our reporter mRNA with an unrelated nucleotide sequence (encoding BFP) and found no GFP foci formation near mRNAs (Figure S1E).

In conclusion, we have developed assays that enable both single mRNAs and their associated nascent translating polypeptides to be imaged over time. This general SunTag-based method can be performed with either freely diffusing mRNAs or mRNAs tethered to the plasma membrane, each of which has unique advantages depending on the specific biological question (Discussion). For further experiments in this study, we used the membrane-tethered system to follow translation for long periods of time.

### Measurement of Ribosome Number, Initiation Rate, and Elongation Rate on Single mRNAs

**Figure 2:**
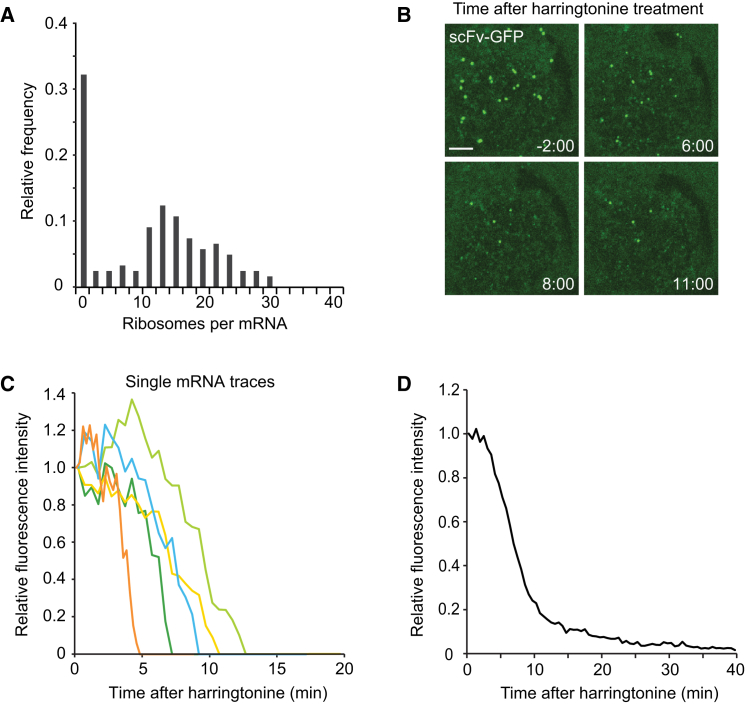
Measurements of Ribosome Initiation and Elongation Rates on Single mRNA Molecules U2OS cells expressing scFv-GFP, PP7-2xmCherry-CAAX, and the translation reporter (SunTag_24x_-Kif18b-PP7_24x_). (A) Distribution of the number of ribosomes bound to single mRNAs of the translation reporter (SunTag_24x_-Kif18b-PP7_24x_) (n = 2 independent experiments, 16 cells, 124 mRNAs), see Supplemental Experimental Procedures. (B–D) U2OS cells expressing the translation reporter (SunTag_24x_-Kif18b-PP7_24x_) were treated with harringtonine at t = 0. (B) Representative images from a time-lapse movie. (C) Five representative traces of fluorescence decay on single mRNAs (of >100 analyzed). (D) Normalized quantification of the decrease in fluorescence over time from many translation sites (n = 4 independent experiments, 37 cells, 536 mRNAs). Scale bars, 5 μm. See also Figure S2 and Movie S4.

**Figure S2:**
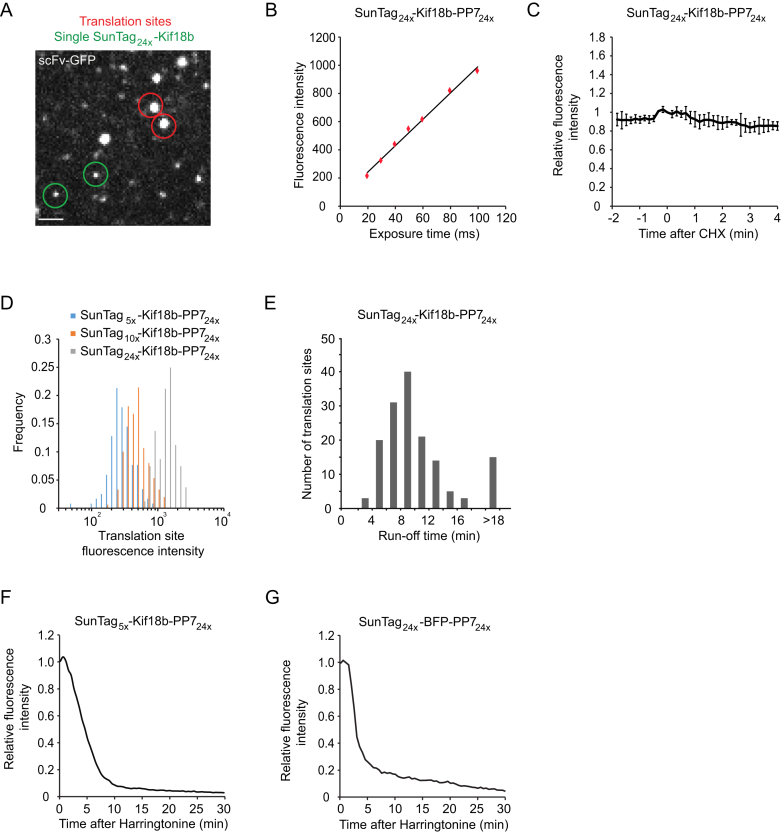
Quantification of Ribosome Number and Elongation Speed on Single mRNAs, Related to Figure 2 U2OS cells expressing scFv-GFP, PP7-2xmCherry-CAAX and indicated translation reporters. (A) Images were acquired using short exposure times (40 ms), limiting motion blurring of fast moving particles, so both translation sites (red circle) and single, fully synthesized, freely diffusing SunTag proteins (green circles) could be observed as distinct foci. Fluorescence intensity of single SunTag_24x_-Kif18b foci and single translation site was quantified in the same cell using a ROI with fixed size. (n = 45 translation sites, 15 cells, 3 experiments). (B) To determine whether the exposure time of 40 ms used in (A) was sufficiently short to prevent a reduction in fluorescence intensity of foci due to motion blurring, we measured the intensity of single fully synthesized SunTag_24x_-Kif18b foci at different exposure times. Fluorescence intensities of the ∼25 brightest foci per image were measured. Results show a linear relationship between exposure time and fluorescence intensity at short exposure times, indicating that exposure times where short enough to prevent reduction in fluorescence intensity of foci due to motion blurring (n = 3 independent experiments, 18 cells and 400-500 spots). (C) Cells were treated with 200 μg/mL CHX at t = 0 and fluorescence intensities of translation sites were measured over time. Note that fluorescence does not increase upon CHX treatment (n = 3 independent experiments, 31 cells, 209 mRNAs). Error bars indicate SD. (D) scFv-GFP fluorescence intensity of translation sites using reporters with varying numbers of SunTag peptides (5×, 10× and 24×). (n = 117, 149, 80 translation sites for the 5×, 10× and 24× reporters, respectively). (E–G) Cells were treated with harringtonine at t = 0 and translation-site intensity was quantified over time. (E) Histogram of the total run-off time, measured from the time of harringtonine treatment to the final disappearance of the scFv-GFP signal. 60 s was subtracted from all times to correct for the time required for harringtonine to enter the cell. (F and G) ScFv-GFP fluorescence intensity was measured over time after harringtonine addition (F, n = 3 independent experiments, 39 cells, 1883 mRNAs) (G, n = 3 independent experiments, 30 cells, 378 mRNAs). Scale bar, 2 μm.

To estimate the number of ribosomes translating each mRNA, we compared the scFv-GFP fluorescence intensity of translation sites with that of the single, fully synthesized SunTag_24x_-Kif18b molecules present in the same cell (Figures S2A and S2B). Several considerations need to be taken into account to calculate ribosome number from the fluorescence intensities of translation sites and fully synthesized single SunTag proteins (Supplemental Experimental Procedures). First, ribosomes present at the 5′ end of the reporter transcript have translated only a subset of the 24 SunTag peptides, so the nascent polypeptide associated with these ribosomes will have lower fluorescence intensity due to fewer bound scFv-GFPs. We generated a mathematical model to correct for the difference in fluorescence intensity for ribosomes at different positions along the transcript (Supplemental Experimental Procedures). Second, if scFv-GFP-peptide has a slow on rate for the epitope in vivo, a lag time could exist between the synthesis of a SunTag peptide and binding of a scFv-GFP, which could result in the underestimation of the number of ribosomes per mRNA. To test this, cells were treated with the translation inhibitor cycloheximide (CHX), which blocks ribosome elongation by locking ribosomes on the mRNA and prevents the synthesis of new SunTag peptides, while allowing binding of scFv-GFP to existing peptides to reach equilibrium. The translation site scFv-GFP signal did not substantially increase after CHX treatment (Figure S2C), indicating that under our experimental conditions, the lag time between peptide synthesis and scFv-GFP binding does not detectably affect translation-site intensity. Based on the above controls and our mathematical model, we could estimate the ribosome number per mRNA from the fluorescence intensity of the translation site. Approximately 30% of the mRNAs did not have a corresponding GFP signal, suggesting that they were not actively translating. For the remaining 70% of the mRNAs that were translating, the majority (76%) had between 10–25 ribosomes (Figure 2A; Supplemental Experimental Procedures), corresponding to an average inter-ribosome distance of ∼200–400 nucleotides (nt). We also compared translation-site intensity of two additional reporter mRNAs with either 5× or 10× SunTag peptides with the 24× peptide reporter. This analysis revealed that ribosome density was very similar on the 5× and 10× reporter (1.26-fold and 1-fold, respectively) (Figure S2D), indicating that the long 24× SunTag array does not grossly perturb ribosome loading on the reporter mRNA.

Next, we measured the translocation speed of ribosomes on single mRNAs by treating cells with harringtonine, a small molecule inhibitor of translation that stalls new ribosomes at the start of the mRNA coding sequence without affecting ribosomes further downstream (Ingolia et al., 2011). As mRNA-bound ribosomes complete translation one-by-one after harringtonine treatment, the GFP signal on mRNAs decreases (Figures 2B–2D; Movie S4). Using a simple mathematical model to fit the decay in fluorescence of a cumulative curve from many mRNAs (Figure S7; Supplemental Experimental Procedures), we estimate a ribosome translocation rate of 3.5 ± 1.1 codons/s. In a parallel approach, we also measured the total time required for runoff of all ribosomes from individual mRNAs (Figure S2E), from which we calculated a similar translation elongation rate (3.1 ± 0.14 codons/s) as the one obtained through our model (Supplemental Experimental Procedures). A reporter with only 5 instead of 24 SunTag peptides showed similar elongation kinetics (3.1 ± 0.4 codons/s) (Figure S2F), indicating that translocation rates are likely not affected by SunTag labeling of the nascent chain. Finally, we measured elongation rates of a shorter and codon-optimized reporter gene, which revealed a somewhat faster elongation rate of 4.9 codons/s (Figure S2G), indicating that elongation rates may differ on different transcripts. Using the elongation rate and ribosome density described above, we were able to estimate the translation initiation rate to be between 1.4–3.6 min^−1^ on the Kif18b reporter (Supplemental Experimental Procedures).

Together, these results provide the first in vivo measurements of the rates of ribosome initiation and translocation on single mRNA molecules in live cells.

### Temporal Changes in Translation of Single mRNA Molecules

**Figure 3:**
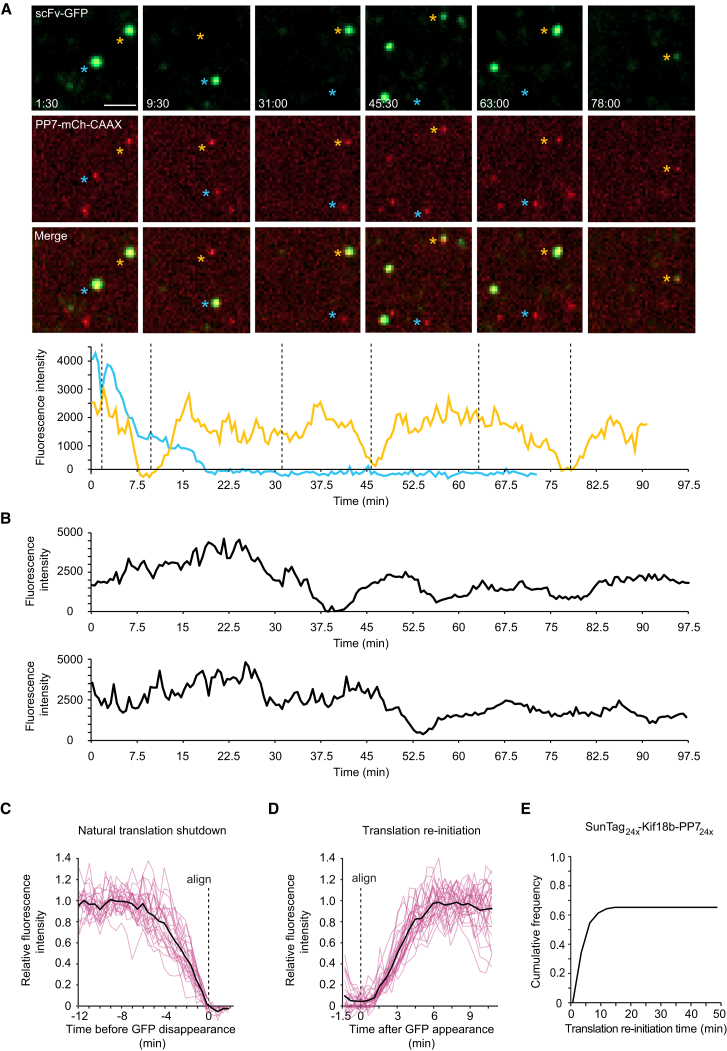
Long-Term Dynamics of Translation of Single mRNA Molecules U2OS cells expressing scFv-GFP, PP7-2xmCherry-CAAX, and the translation reporter (SunTag_24x_-Kif18b-PP7_24x_). (A) U2OS cell expressing the SunTag_24x_-Kif18b-PP7_24x_ reporter was imaged by time-lapse microscopy. Blue and yellow asterisks mark two different mRNAs undergoing changes in translation over time (upper). Intensity of scFv-GFP was measured over time for the two mRNAs (lower). Colors of lines correspond to scFv-GFP intensity of translation sites marked by asterisk with the same color. (B) ScFv-GFP intensity traces of two additional mRNA molecules. (C) mRNAs undergoing permanent translation shutdown. Fluorescence intensity quantification is shown (n = 24 mRNAs). Average (black line) and single traces (pink lines) are shown. (D) mRNAs undergoing translation re-activation after shutdown. Average (black line) and single traces (pink lines) are shown (n = 30 mRNAs). (E) Time to reappearance of the first scFv-GFP fluorescence from translation sites that underwent complete translational shutdown. ∼60% of the mRNAs re-initiated translation after complete shutdown and did so within 10 min (n = 104 translational sites analyzed). Scale bar, 2 μm. See also Figure S3 and Movie S5.

**Figure S3:**
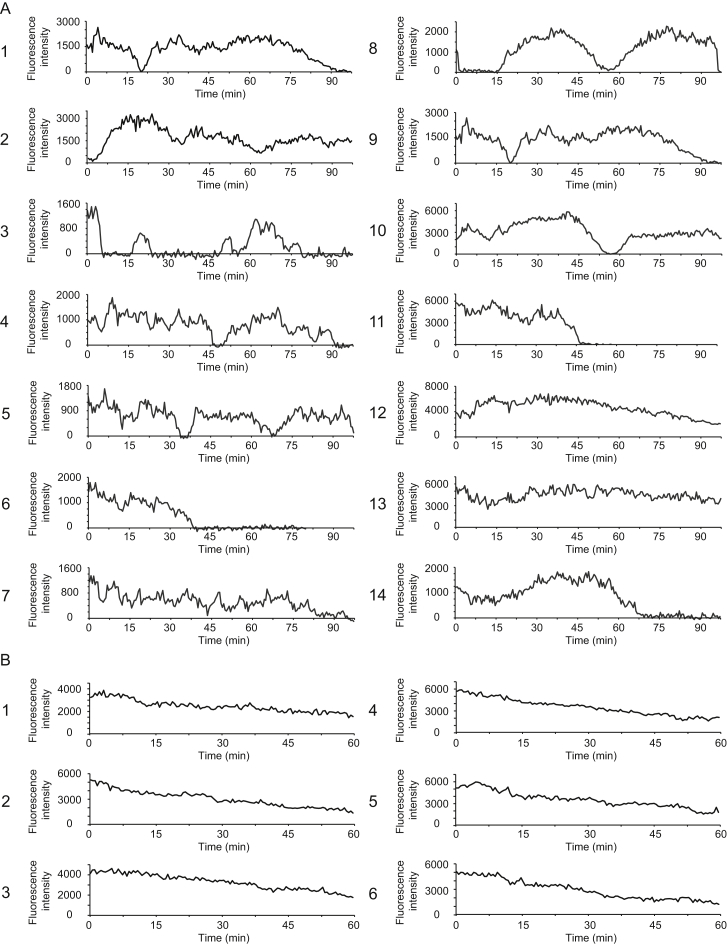
Translation Dynamics of Single mRNA Molecules, Related to Figure 3 U2OS cells expressing scFv-GFP, PP7-2xmCherry-CAAX and the translation reporter (SunTag_24x_-Kif18b-PP7_24x_) were imaged by time-lapse microscopy for 2 hr (A) or 1 hr (B) and the fluorescence intensity of single translation sites was tracked over time. 14 traces of untreated cells (A) or 6 traces of CHX treated cells (B) are shown. Note that the intensity of translation sites in CHX-treated cells slowly decreases over time, which is likely due to a decrease in the ribosome number per mRNA after prolonged CHX treatment.

To study translation over time, we imaged cells for 2 hr and quantified the scFv-GFP signal from single mRNA molecules that could be tracked for >1 hr (Figures 3A, 3B, and S3A). The results show considerable fluctuations in the translational state of individual mRNAs over time (Figures 3A, 3B, and S3A). Such large fluctuations were not observed when cells were treated with the translation inhibitor CHX (Figure S3B), indicating they were due to changes in translation initiation and/or elongation rather than measurement noise. We also observed heterogeneity of behavior between different mRNAs. Some remained in a high translating state for >1 hr (e.g., Figures S3A12 and 13). Others shut down translation initiation and lost their scFv-GFP signal (e.g., Figures 3A, 3B, and S3A1, 3–11, and 14), which may account for the population of non-translating mRNAs observed in steady-state measurements (Figure 2A). From the progressive decline in scFv-GFP fluorescence (Figure 3C; Movie S5), we could estimate a ribosome run-off rate of 3 codons/s (Figure 3C), which is similar to that measured after addition of harringtonine (3.5 ± 1.1 codons/s) (Figure 2). Interestingly, a subset (67 of 104 mRNAs, three independent experiments, 19 cells) of these mRNAs later reinitiated translation and largely recovered their original scFv-GFP fluorescence (Figures 3A, 3B, 3D, and S3A1, 3, 5, and 8–10). Individual mRNAs even showed repeated cycling between non-translating and translating states (Figure 3A, yellow line, and S3A3, 5 and 8). Such cycles of complete translational shutdown and re-initiation occurred 0.29 ± 0.10 times per mRNA per hour (n = 4 independent experiments, 27 cells, 106 mRNAs), suggesting that most mRNAs will undergo one or more translational shutdown and re-initiation events in their lifetime. Thus, single mRNA imaging reveals reversible switching between translational shutdown and polysome formation.

**Figure 4:**
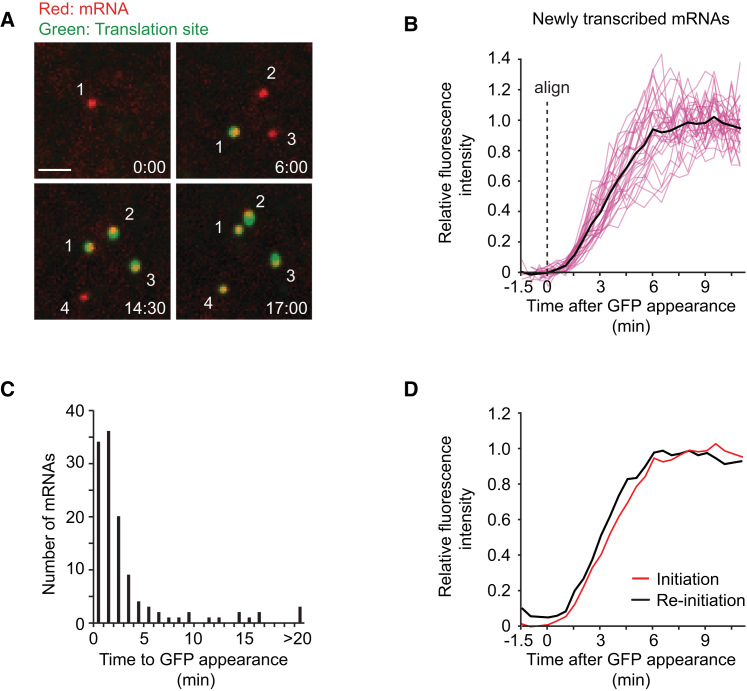
Analysis of Polysome Build Up on Newly Transcribed mRNAs U2OS cells expressing scFv-GFP, PP7-2xmCherry-CAAX, and the translation reporter (SunTag_24x_-Kif18b-PP7_24x_). (A) Images from a time-lapse movie of newly transcribed mRNAs undergoing the first rounds of translation. (B) Quantification of the fluorescence intensity increase, aligned at the first time point at which scFv-GFP signal was detected (n = 30 individual mRNAs [pink lines], and average [black line] is shown). (C) Quantification of the time between mRNA appearance and the first detection of translation by scFv-GFP fluorescence. (D) Comparison of scFv-GFP fluorescence buildup on either new transcripts (red line) or on re-initiating mRNAs (black line). Data are re-plotted from Figures 3D and 4B. Scale bar, 2 μm. See also Movie S6.

After synchronized expression of the reporter construct using an inducible promoter, we often observed the initial binding events of newly transcribed mRNAs to the PP7-mCherry at the membrane (Figures 4A and 4B). Of these initial binding events, 44% of the mRNAs were associated with scFv-GFP fluorescence, indicating that they had already begun translation. However, the majority, 56% of mRNAs, initially appeared at the membrane in a non-translating state and subsequently converted to a translating state, usually within 1–5 min (Figure 4C; Movie S6). These mRNAs are likely newly transcribed mRNAs that are translating for the first time, rather than mRNAs that have already undergone translation but transitioned temporarily to a non-translating state. In support of this argument, long-term (>1 hr) imaging of single mRNAs reveals that mRNAs spend on average only 2.5% of their lifetime in such a temporary non-translating state (n = 4 independent experiments, 27 cells, 106 mRNAs), which is not sufficient to explain the 56% non-translating mRNAs that appeared at the membrane after synchronized transcription of the reporter. Rapid initiation of translation on newly transcribed mRNAs was described recently (Halstead et al., 2015), but our assay additionally allows an analysis of polysome buildup on new mRNAs (Figure 4B). Our analysis of the increase in scFv-GFP fluorescence indicates that, once the first ribosome begins chain elongation, additional ribosomes initiate translation with a rate indistinguishable from that on polysomes at steady state (Supplemental Experimental Procedures). We also examined the rate of fluorescence recovery (corresponding to polysome buildup) after complete shutdown of translation and subsequent re-initiation (Figure 4D). The polysome buildup on new transcripts was comparable to that observed for mRNAs that were cycling between translating and non-translating states (Figure 4D).

### Ribosome Stalling

**Figure S4:**
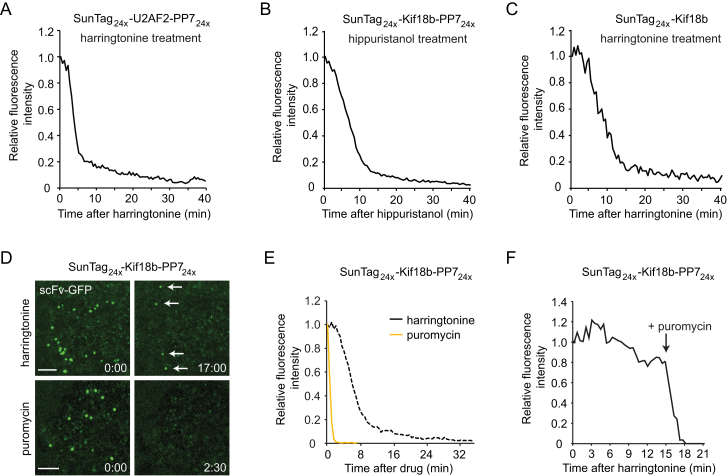
Validation of the Ribosome Stalling Phenotype, Related to Figure 5 (A) U2OS cells expressing scFv-GFP, PP7-2xmCherry-CAAX and indicated translation reporters were treated with harringtonine at t = 0 and translation-site intensity was quantified over time. Reporter containing a codon optimized version of the U2AF2 coding sequence (n = 3 independent experiments, 29 cells, 512 mRNAs). (B) U2OS cells expressing the translation reporter (SunTag_24x_-Kif18b-PP7_24x_) were treated with another translation initiation inhibitor (hippuristanol) and translation-site intensity was quantified over time (n = 2 independent experiments, 14 cells, 515 mRNAs). (C) U2OS cells expressing scFv-GFP, PP7-2xmCherry-CAAX and indicated translation reporters were treated with harringtonine at t = 0 and translation-site intensity was quantified over time. Harringtonine run-off experiments were also performed on a translation reporter lacking PP7 binding sites (SunTag_24x_-Kif18b) (n = 2 independent experiments, 19 cells, 248 mRNAs). (D) U2OS cells expressing scFv-GFP, PP7-2xmCherry-CAAX and indicated translation reporters were treated with harringtonine at t = 0 and translation-site intensity was quantified over time. Representative images in which stalled ribosome can be observed after harringtonine treatment (arrows). No ribosome stalling is observed after puromycin treatment (lower panel). (E) U2OS cells expressing scFv-GFP, PP7-2xmCherry-CAAX and indicated translation reporters were treated with harringtonine at t = 0 and translation-site intensity was quantified over time. At t = 0, either harringtonine (re-plotted from Figure 2D) or puromycin (n = 3 independent experiments, 22 cells, 403 mRNAs) was added and translation-site intensity was quantified over time. (F) U2OS cells expressing scFv-GFP, PP7-2xmCherry-CAAX and indicated translation reporters were treated with harringtonine at t = 0 and translation-site intensity was quantified over time. Sequential addition of harringtonine and then puromycin (n = 7 mRNAs). Scale bar, 5 μm.

Several studies reported that ribosomes can pause or stall at a defined nucleic acid sequence with a regulatory function (Walter and Blobel, 1981, Yanagitani et al., 2011), at chemically modified or damaged nucleotides (Simms et al., 2014), or at regions in the RNA with a strong secondary structure (Tholstrup et al., 2012, Wen et al., 2008). We found that a subset (∼5%–10%) of mRNAs retained a bright scFv-GFP signal 15 min after harringtonine treatment (Figures 2B and 2D), a time at which ribosomes translocating at ∼3 codons/s should have finished translating the reporter. A similar percentage of stalled ribosomes was observed on two additional reporter transcripts, both of which were designed using optimal codon usage (Figures S2G and S4A). Ribosome stalling also was observed using hippuristanol (Figure S4B), a translation initiation inhibitor with a different mechanism of inhibition (Bordeleau et al., 2006), indicating that the stalling was not caused by harringtonine. We also observed stalls when examining ribosome runoff from non-tethered cytosolic mRNAs lacking PP7 binding sites (Figure S4C). Importantly, stalls were not observed after puromycin treatment (Figures S4D and S4E) and the prolonged (>15 min) scFv-GFP signal on mRNAs from harringtonine-treated cells rapidly disappeared upon the addition of puromycin, confirming that the observed signal indeed represents stalled ribosomes (Figure S4F). The majority of mRNAs with stalled ribosomes (33 of 43) could be tracked for >40 min, the typical duration of our harringtonine runoff experiments, indicating that they were not readily targeted by the no-go mRNA decay machinery within this time frame.

**Figure 5:**
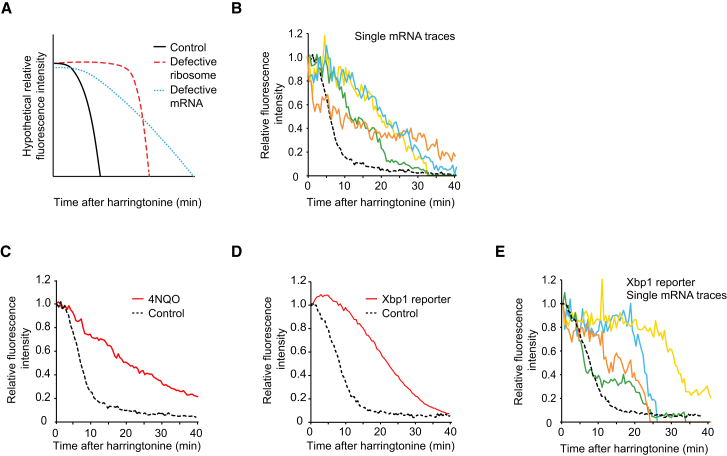
Dynamics of Ribosome Stalling U2OS cells expressing scFv-GFP, PP7-2xmCherry-CAAX, and the SunTag_24x_-Kif18b-PP7_24x_ translation reporter (A–C) or the Xbp1 translation reporter (D–E). (A and B) Ribosome stalling likely results from mRNA defects, model (A) and experiment (B). (B) Fluorescence intensity over time is shown for four representative stalled translation sites (colors; of 20 analyzed). Since intensity values of single mRNAs were derived from the experiments presented in Figure 2D, the average fluorescence decay presented in Figure 2D is re-plotted here for comparison (dashed black line). (C) Nucleic acid damage through 4NQO treatment (red line) induces ribosome stalling (n = 3 independent experiments, 40 cells, 455 mRNAs). For comparison, the harringtonine runoff from control cells with the SunTag_24x_-Kif18b-PP7_24x_ reporter from Figure 2D is re-plotted, as these experiments were performed in parallel. (D and E) Harringtonine runoff for the Xbp1 pause site (red line, n = 3 independent experiments, 31 cells, 990 mRNAs) (D) and control reporter (black dashed lines, n = 3 independent experiments, 27 cells, 437 mRNAs) (E). See also Figure S4.

Ribosome stalls could be due to defective ribosomes causing roadblocks on the mRNA or due to defects in the mRNA. These models can potentially be distinguished by examining how such stalls are resolved. A single defective ribosome will inhibit ribosome runoff until the stalled ribosome is removed, after which, the remaining ribosomes will run off at a normal rate. In contrast, if the stalls are caused by defects to the mRNA, such as chemical damage, then each ribosome passing over the damaged nucleotide will be delayed, resulting in an overall slower scFv-GFP decay rate (Figure 5A). Long-term tracking of stalled ribosomes on single mRNAs was consistent with the latter model, indicating that ribosome stalling is likely caused by defective mRNA (Figure 5B). Consistent with the hypothesis that chemical damage to mRNA causes ribosome stalling, treatment of cells with 4-nitroquilone-1-oxide (4NQO), a potent nucleic-acid-damaging agent that causes 8-oxoguanine modifications and stalls ribosomes in vitro (Simms et al., 2014), resulted in a slow runoff on the majority of mRNAs, indicating widespread ribosome stalling (Figure 5C). Thus, chemical damage to mRNAs stalls ribosome elongation in vivo.

Regulated ribosome pausing occurs both in vitro and in vivo at asparagine 256 in the stress-related transcription factor Xbp1 (Ingolia et al., 2011, Yanagitani et al., 2011), and this ribosome pausing is important for membrane targeting of the mRNA (Yanagitani et al., 2011). To test whether our translation imaging system could recapitulate such translation pausing, we introduced a strong ribosome-pausing sequence (a point mutant of the wild-type Xbp1-pausing sequence that shows enhanced ribosome pausing [Yanagitani et al., 2011]) into the 3′ region of the coding sequence of our reporter (hereafter referred to as Xbp1 reporter). Harringtonine ribosome runoff experiments on the Xbp1 reporter revealed a delay in ribosome runoff (Figure 5D), confirming that our reporter faithfully reproduced the ribosome-pausing phenotype. To study the behavior of individual ribosomes on the Xbp1 ribosome-pausing sequence, we tracked single mRNAs during ribosome runoff. Surprisingly, the fluorescence decay was not linear, as would be expected if each ribosome paused a similar amount of time on the pause site. Rather, fluorescence decay occurred in bursts interspaced with periods in which no decay was detectable (Figures 4 and 5E, representative traces shown out of 25 analyzed). These results indicate that most ribosomes are only briefly delayed at the Xbp1 pause site, but a small subset of ribosomes remain stalled for an extended (>10 min) period of time, explaining the strong ribosome stalling phenotype observed in ensemble experiments.

### Translational Regulation of the Cell-Cycle Regulator Emi1

**Figure 6:**
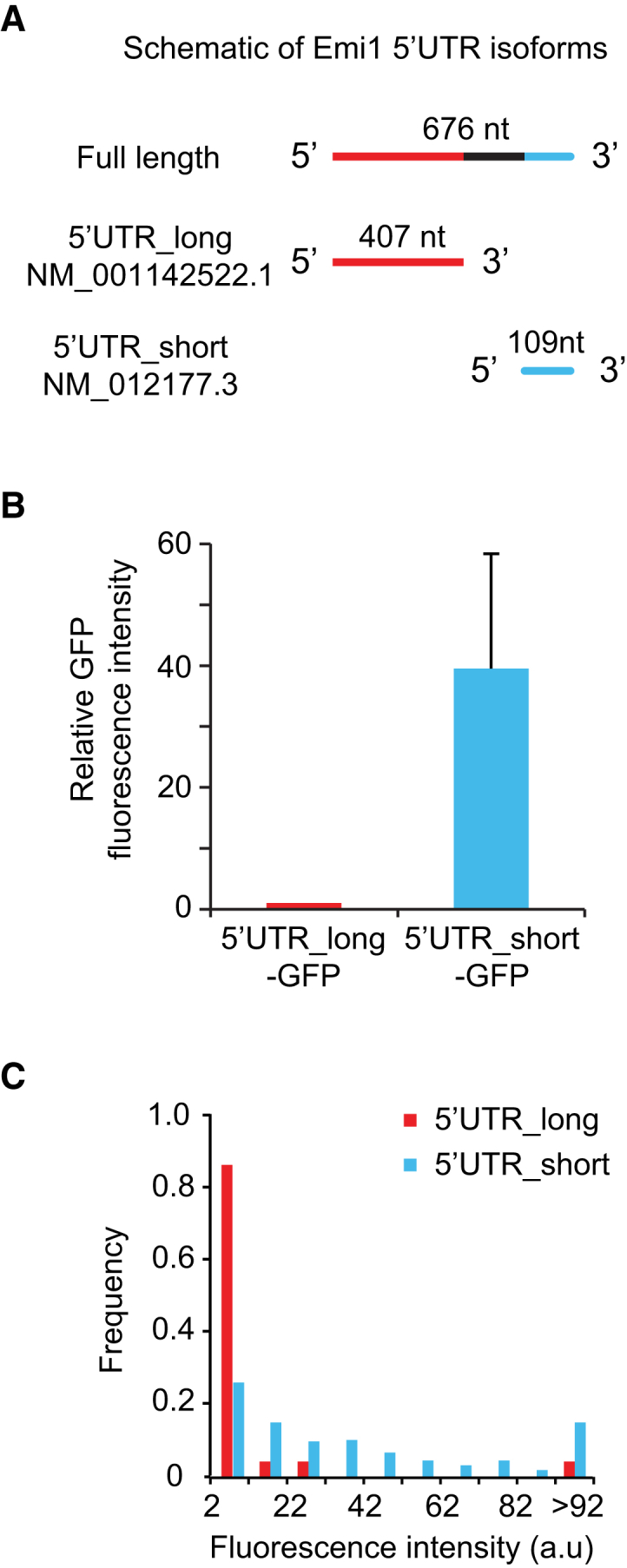
Differential Control of Translation Initiation by Two Emi1 Splicing Isoforms (A) Schematic of the 5′ UTR of two Emi1 splicing isoforms. (B) Fluorescence intensity of a GFP reporter under control of the two Emi1 isoforms (5′ UTR_long and 5′ UTR_short) expressed in HEK293 cells was measured by microscopy for single cells. Mean intensities were determined, which was corrected for background fluorescence in untransfected cells. At least 20 cells were measured per experiment per condition. Error bars, SD between experiments. (C) Fluorescence intensity distributions of single translation sites of indicated reporters, n = 3 independent experiments, 283 mRNAs, 14 cells (5′ UTR_long) and n = 3 independent experiments, 433 mRNAs, 16 cells (5′ UTR_short). Background from adjacent regions was subtracted. Only mRNAs are plotted that had translation signal above background (with an intensity value >2; 16% and 53% of mRNAs for 5′ UTR_long and 5′ UTR_short, respectively). See also Figure S5.

**Figure S5:**
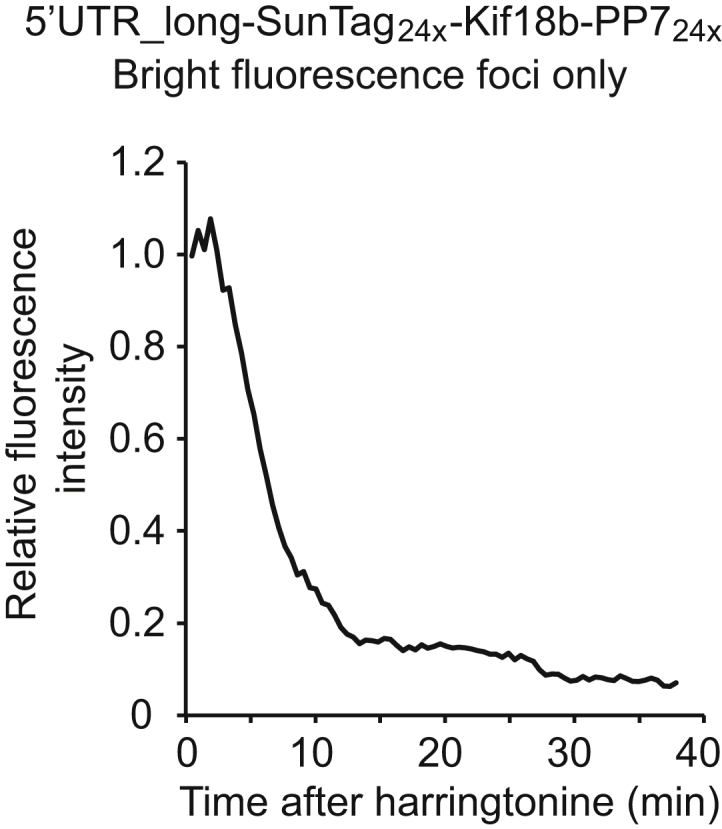
Ribosome Elongation Rates on Emi1 5′UTR_Long Containing mRNAs, Related to Figure 6 U2OS cells expressing scFv-GFP, PP7-2xmCherry-CAAX and the Emi1 5′UTR_long translation reporter were treated with harringtonine at t = 0. The fluorescence intensity of very bright translation sites was quantified over time (n = 3 independent experiments, 29 cells, 39 mRNAs).

We also applied our assay to study the transcript-specific translational regulation of Emi1, a key cell-cycle regulatory protein. Our recent work reported strong translational repression of Emi1 during mitosis and found that the 3′ UTR of Emi1 is involved in this regulation (Tanenbaum et al., 2015), but a role of its 5′ UTR in translational regulation was not established. Interestingly, Emi1 has at least two splicing isoforms that differ in their 5′ UTR sequence: NM_001142522.1 (hereafter referred to as 5′ UTR_long) and NM_012177.3 (hereafter referred to as 5′ UTR_short) (Figure 6A). We found that a GFP protein fused downstream of the 5′ UTR_long was expressed at 40-fold lower levels than a GFP fused to the 5′ UTR_short (Figure 6B). Such difference in protein expression could be due to a difference in transcription rate, mRNA stability, or reduced translation initiation or elongation rates. To distinguish between these possibilities, we prepared translation reporter constructs bearing either the short or long 5′ UTR of Emi1. Robust translation was observed on ∼50% of mRNAs encoding the short 5′ UTR (Figure 6C). In contrast, the majority (∼80%) of transcripts encoding the Emi1 5′ UTR_long showed no detectable translation (not shown), and of the translating mRNAs, only very weak scFv-GFP fluorescence was usually detected (Figure 6C). Surprisingly, however, a very small fraction of mRNAs containing the 5′ UTR_long (∼2%) was associated with a bright scFv-GFP signal (Figure 6C, >92 bin), indicating that they are bound to many ribosomes. This was not due to ribosome stalling and subsequent (slow) accumulation of ribosomes on a subset of mRNAs, as this bright scFv-GFP signal rapidly dissipated upon harringtonine treatment (Figure S5), indicating that these mRNAs were translated at high levels. Calculation of the total number of ribosomes associated with the mRNAs, based upon scFv-GFP fluorescence intensity, revealed that 52% of all ribosomes translating the Emi1 5′ UTR_long reporter were associated with the minor (2%) fraction associated with the highest scFv-GFP intensity. These results indicate that the great majority of 5′ UTR_long transcripts are strongly translationally repressed but that a small subset of these mRNAs escape repression and undergo robust translation. Thus, substantial heterogeneity in translational efficiency can exist among different mRNA molecules within the same cell.

### Observation of Translation by Single Ribosomes

**Figure 7:**
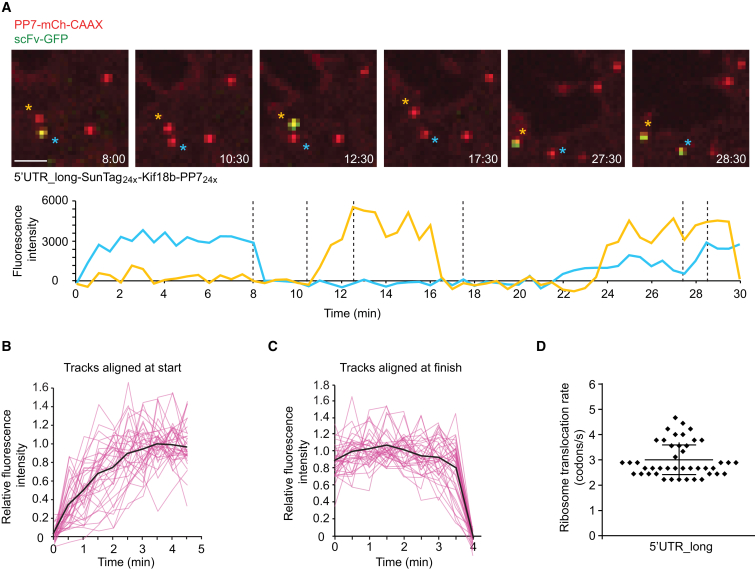
Visualizing Single Ribosomes Decoding an mRNA Molecule (A–D) Analysis of single ribosomes on the Emi1 5′UTR_long reporter mRNA. (A) Representative images of multiple single ribosome translation events of individual mRNAs (upper). ScFv-GFP intensity was quantified over time for the two mRNAs marked by asterisks with the same color (lower). (B) Increase in scFv-GFP fluorescence from single ribosome translation events aligned at the first detectable scFv-GFP signal (n = 35 individual mRNAs in pink and average in black). (C) Steady-state and then abrupt decrease in scFv-GFP fluorescence from single translating ribosomes (n = 35 individual mRNAs [pink] and average [black]). (D) Single ribosome elongation rates (n = 44) (Supplemental Experimental Procedures). Mean ± SD is shown in (D). Scale bar, 2 μm. See also Figure S6 and Movie S7.

**Figure S6:**
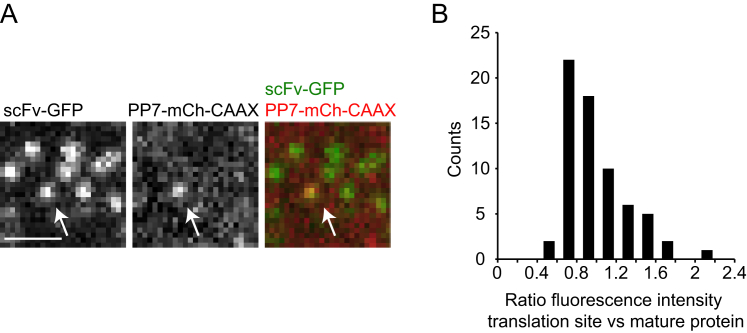
Single Ribosomes Translate the Emi1 5′UTR_Long mRNA, Related to Figure 7 U2OS cells expressing scFv-GFP, PP7-2xmCherry-CAAX and the Emi1 5′UTR_long translation reporter were imaged using a very short (30 ms) exposure time, so fully synthesized, freely diffusing mature SunTag-Kif18b molecules can be observed together with translation sites. Translation sites could be distinguished from fully synthesized SunTag molecules, as they co-migrated with mRNAs for multiple (> 5) consecutive time points. (A) Representative image of a single translation site (arrow) surrounded by multiple mature SunTag molecules. (B) Quantification of fluorescence intensities of translation sites and mature protein. The fluorescence intensity of a single translation sites was compared to the average fluorescence intensity of 5 nearby mature SunTag molecules.

Interestingly, with the Emi1 5′ UTR_long reporter, we often observed the abrupt appearance of a weak scFv-GFP signal on a transcript that was previously translationally silent. The GFP signal initially increased over time, plateaued, and then was abruptly lost after 6–8 min (Figures 7A–7C; Movie S7). This type of signal is best explained by a single ribosome sequentially decoding the 24 SunTag peptides on the mRNA, followed by the release of the newly synthesized polypeptide upon completion of translation. Consistent with this hypothesis, the absolute fluorescence intensity of such translation events at the plateau phase (when all 24 SunTag peptides have been synthesized) was very similar to the intensity of a single fully synthesized SunTag24x-Kif18b protein (Figures S6A and S6B). The duration of the scFv-GFP signal per translation event could be converted to a translocation speed of single ribosomes (Supplemental Experimental Procedures), which revealed an average elongation rate of 3 codons/s (Figure 7D). This value is similar to that determined from our bulk measurements of harringtonine-induced ribosome runoff or natural translational initiation shutdown and runoff (3–3.5 codons/s), indicating that ribosome elongation was not affected by the Emi1 5′ UTR_long. Comparison of translocation rates obtained from single ribosome translation events also revealed heterogeneity in the decoding speed of individual ribosomes in vivo (Figure 7D).

## Discussion

Using the SunTag system, we have developed an imaging method that measures the translation of individual mRNAs in living cells. Immobilization of mRNAs on the plasma membrane allows the long-term (>1 hr) observation of translation of single mRNA molecules, which enables analyses of translational initiation, elongation, and stalling in live cells for the first time. Under conditions of infrequent translational initiation, we can even observe a single ribosome decoding an entire mRNA molecule. Our observations reveal considerable and unexpected heterogeneity in the translation properties of different mRNA molecules derived from the same gene in a single cell, with some not translating, others actively translating with many ribosomes, and others bound to stalled ribosomes. The SunTag translation imaging assay should be applicable to many different cell types, including neurons and embryos, in which the localization and control of protein translation is thought to play an important role in cell function.

### Comparison of Methods to Study Translation In Vivo

Ribosome profiling, a method in which fragments of mRNAs that are protected by the ribosome are analyzed by deep sequencing (Ingolia et al., 2009), has found widespread use in measuring translation. The strength of ribosomal profiling lies in its ability to measure translation on a genome-wide scale of endogenous mRNAs. However, a limitation of ribosome profiling is the need to pool mRNAs from many thousands of cells for a single measurement. Thus, ribosome profiling in its present form cannot be used to study translation heterogeneity between different cells in a population or among different mRNA molecules in the same cell. Furthermore, since ribosome profiling requires cell lysis, only a single measurement can be made for each sample, limiting studies of temporal changes.

A number of single-cell translation reporters have been developed based on fluorescent proteins (Aakalu et al., 2001, Brittis et al., 2002, Han et al., 2014, Raab-Graham et al., 2006, Tanenbaum et al., 2015, Tatavarty et al., 2012, Yu et al., 2006). Such reporters generally rely on the accumulation of new fluorescence after the assay is initiated. Advantages of these systems are that they are generally easy to use and have single-cell sensitivity. However, they do not provide single-mRNA resolution, often do not allow continuous measurement of translation, and do not report on ribosome initiation and elongation rates.

Finally, two methods were developed recently to image translation on single mRNAs in vivo. In one approach, the first round of translation is visualized (Halstead et al., 2015). This method, however, does not allow continuous measurements of translation. The second approach involves measurements of the number of ribosomes bound to an mRNA using fluorescence fluctuation spectroscopy (Wu et al., 2015). The advantage of this method is that it can detect binding of a single fluorescent protein to an mRNA and different subcellular sites can be probed to study spatial differences in translation. The limitation of this method though is the inability to follow translation of single mRNAs over time, as these mRNAs cannot be tracked in the cell.

SunTag-based translation imaging assays are unique thus far in their ability to follow translation of individual mRNAs over time. This translation assay can be employed with either freely diffusing or tethered mRNAs, the choice of which will depend on the biological question to be addressed. In the study by Wang et al. (2016) [this issue of *Cell*], translation is observed in distinct spatial compartments in neurons using a similar SunTag-based translation imaging method with non-tethered mRNAs. In contrast, for studying ribosome translocation dynamics, the tethering assay provides the ability to track a single mRNA throughout the duration of the ribosome elongation cycle. Using this assay, we could measure polysome buildup rates over time, observe mRNAs cycling between translating and non-translating states, uncover heterogeneity in translation initiation rates (e.g., with the Emi1 5′ UTR) and even observe a single ribosome translating an entire transcript. These measurements were aided by the vastly improved signal-to-noise of the tethered assay and the ability to easily track slowly diffusing tethered mRNAs for an hour or more. These long-term observations allowed us to discover that mRNAs can reversibly switch between a translating and non-translating state and have a high variability in pause duration at the Xbp1 site. Thus, the untethered and tethered SunTag assays provide means to study translation of single mRNA molecules, which will be applicable to a wide variety of biological questions and will be complementary to existing methods of studying translation.

A drawback of our assay is the need to insert an array of SunTag peptide repeats into the mRNA of interest to fluorescently label the nascent polypeptide and the need to insert an array of PP7 binding sites in the 3′ UTR to label the mRNA. As is true of any tagging strategy, these modifications could interfere with translation and/or mRNA stability under certain conditions. We have performed a number of control experiments to ensure that binding the scFv-GFP to the nascent chain and tethering of the transcript to the membrane do not grossly perturb translation (Figures 1B and 1F). We have also shown that ribosome translocation rates and ribosome density are similar when using a reporter with a very short (5×) or long (24×) SunTag peptide array and comparing tethered and non-tethered mRNAs (Figures 2D, S2F, and S4C), indicating that many aspects of translation are not perturbed in our assay. Nevertheless, tethering of certain mRNAs to the plasma membrane may influence translation, especially for those mRNAs that undergo local translation in a specific compartment of the cell. Thus, our assay has unique advantages for certain types of measurements of translation, but appropriate controls should be performed for each experimental system or objective.

### Heterogeneity in Translation of Single mRNAs: Possible Molecular Mechanisms

**Figure S7:**
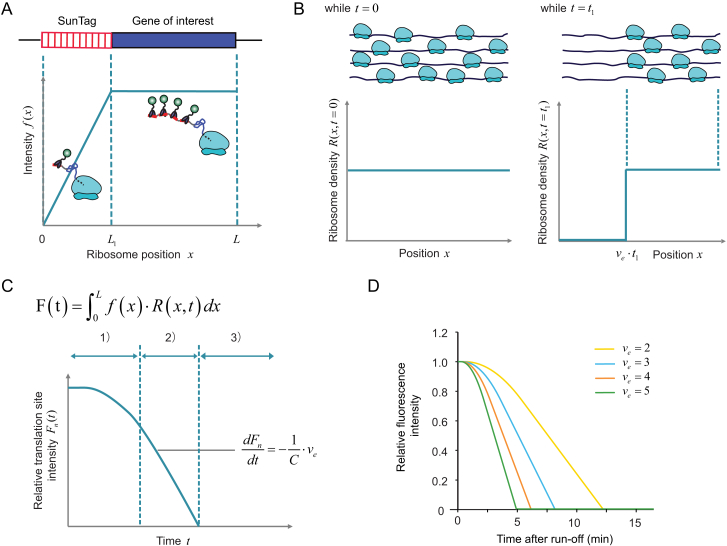
Modeling of Translation-Site Intensity, Related to Experimental Procedures (A) Intensity from a single ribosome mainly depends on ribosome location on the mRNA. Due to the synthesis of SunTag peptides, ribosome intensity will increase initially as the ribosome moves toward the 3′ end until SunTag peptides are fully synthesized and exposed. A typical curve for intensity function *f(x)* is shown. For simplicity, a linear function was used to simulate the intensity increase. (B) Ribosome density changes during ribosome run-off. When there are no new initiation events, already bound ribosomes will runoff the mRNA from 5′ to 3′ end. Examples of the ribosome density function at *t = 0* and *t = t*_*1*_ are shown. (C) Translation-site intensity is dependent on both intensity from single ribosomes as well as ribosome density throughout the mRNA. A formula describing translation-site intensity is shown on top. A typical curve of intensity change during ribosome run-off process is shown at the bottom with three clear stages labeled using numbers. Intensity decreases linearly during the second stage, whose first order derivative could be used to derive elongation rate. (D) Example results of simulations of harringtonine run-off from the Kif18b reporter (SunTag_24x_-Kif18b), which were run using different elongation rates (2, 3, 4, 5 codons/s). Run-off starts at *t = 0*.

Using our system, we measured the ribosome translocation speed on single mRNA molecules. Ribosome translocation rates have been measured in bulk previously in mouse embryonic stem cells (Ingolia et al., 2011), which yielded a translocation rate of 5.6 codons/s. Our values of 3–5 codons/s (Figure S7; Supplemental Experimental Procedures) are in general agreement with those published values and very similar to those measured by Wang et al. (2016) (4 codons/s). Our experiments, and those of Wang et al. (2016), are the first to measure ribosome translocation rates for a single mRNA species, in single cells and on single mRNAs, which provides new opportunities to study regulation of translation elongation.

We also found that translation initiation can shut down temporarily on individual mRNAs and rapidly restart (Figure 3). Such shutdown of translation initiation could be due to transient loss of eIF4E binding to the mRNA cap, mRNA decapping followed by recapping (Mukherjee et al., 2012), or transient binding of regulatory proteins. Using our mRNA tethering assay, binding and unbinding of single proteins to translating mRNA could potentially be observed using total internal reflection fluorescence (TIRF), which could open up many additional possibilities for studying translational regulation at the single-molecule level.

The pioneer round of translation, the first ribosome to initiate translation on a newly transcribed mRNA, may be especially important, as it is thought to detect defects in the mRNA, including premature stop codons (Ishigaki et al., 2001). A recently developed translation biosensor can detect the location of this pioneer round of translation (Halstead et al., 2015). However, what happens after the first ribosome initiates translation is unknown. We found that the translation initiation rate on our reporter mRNA was similar on newly transcribed, recently shut down, and re-initiating mRNAs and polysomal mRNAs (Figure 4; Supplemental Experimental Procedures), indicating that the initiation rate is independent of the number of ribosomes bound to the mRNA. The presence of introns in a gene may also affect translation initiation on newly transcribed mRNAs (Le Hir et al., 2016), which could be tested in future studies.

A subset of ribosomes stall on mRNAs in a sequence-independent fashion (Figures 2D, S2G, and S4A). One possible explanation for this is that ribosome stalling is caused by naturally occurring mRNA “damage” (i.e., chemical modifications of the nucleotides). Previous studies have found that the 8-oxoguanine modification occurs on mRNA in vivo, and such modifications cause ribosome stalling in vitro (Simms et al., 2014) and in vivo (Figure 5C). Alternatively, while we have performed numerous control experiments (Figures 5 and S4), we cannot completely exclude that the observed stalling on a small subset of mRNAs is an artifact of our construct or assay. We also observe ribosome pausing in a sequence-dependent fashion on the pause site of the Xbp1 transcription factor. Such pausing had been observed previously in bulk measurements (Ingolia et al., 2011, Yanagitani et al., 2011), but our quantitative analysis of single mRNAs revealed a high degree of variability in ribosome pausing at this site.

Finally, we show that the 5′ UTR sequence of one Emi1 transcript isoform severely inhibits translation initiation. A likely explanation for this effect is the presence of several upstream open reading frames (uORFs) in this sequence. Surprisingly, a small number of mRNA molecules encoding this 5′ UTR do undergo high levels of translation. It is possible that highly translating mRNAs are generated through alternative downstream transcription start site selection, which generates an mRNA that lacks the repressive sequence (for example, the uORFs). Alternatively, translation could occur if the 5′ UTR repressive sequence is cleaved off, followed by recapping after transcription, if a repressive protein factor dissociates, or if an inhibitory RNA secondary structure unfolds. Further studies will be required to distinguish between these possibilities.

In summary, here we have developed an imaging method that enables the measurement of ribosome initiation and translocation rates on single mRNA molecules in live cells. Future developments of this technology could include simultaneous observation of single translation factors or other regulatory molecules together with mRNAs and nascent polypeptides, which would provide a very powerful system to dissect the molecular mechanisms of translational control.

## Experimental Procedures

### Cell Culture and Drug Treatment

U2OS and HEK293 cells were grown in DMEM/5% with Pen/Strep. Plasmid transfections were performed with Fugene 6 (Roche), and stable transformants were selected with zeocin (Life Technologies). Unless noted otherwise, reporter transcripts were expressed from a doxycycline-inducible promoter, and expression of the reporter was induced with 1 μg/mL doxycycline (Sigma) for 1 hr before imaging. Harringtonine (Cayman Chemical) was used at 3 μg/mL. 5 μM 4NQO (Sigma) was added to cells for 1 hr before imaging. Puromycin (Life Technologies) was used at 100 μg/mL. Hippuristanol (a kind gift of Dr. J. Tanaka) was used at 5 μM. Cycloheximide (Sigma) was used at 200 μg/mL.

### Plasmid Sequences

Sequences of constructs used in this study are provided in the Supplemental Experimental Procedures.

### Microscopy

Cells were grown in 96-well glass bottom dishes (Matriplate, Brooks). Images were acquired using a Yokogawa CSU-X1 spinning disk confocal attached to an inverted Nikon TI microscope with Nikon Perfect Focus system, 100× NA 1.49 objective, an Andor iXon Ultra 897 EM-CCD camera, and Micro-Manager software (Edelstein et al., 2010). Single z-plane images were acquired every 30 s unless noted otherwise. During image acquisition, cells were maintained at a constant temperature of 36°C–37°C. Camera exposure times were generally set to 500 ms, unless noted otherwise. We note that stable expression of PP7-mCherry, either with or without the CAAX domain, also resulted in an accumulation of mCherry signal in lysosomes, but lysosomes could be readily distinguished from mRNA foci based on signal intensity and mobility.

### FACS

GFP and scFv-GFP (Figure 1B), mCherry, PP7-mCherry, or PP7-2xmCherry-CAAX (Figure 1F) were expressed from a constitutive promoter, while the two reporters, SunTag_24x_-mCherry and GFP-PP7_24x_ (Figures 1B and 1F, respectively) were expressed from an inducible promoter in U2OS cells expressing the Tet repressor protein, and their expression was induced 24 hr after transfection using doxycycline (1 μg/mL). This ensured that the reporters were translated in the presence of high levels of the scFv-GFP and PP7-2xmCherry-CAAX proteins. Cells were collected one day after doxycycline induction and analyzed by FACS. Cells were gated for GFP and mCherry double positivity, and the mCherry and GFP levels (Figures 1B and 1F, respectively) were analyzed using Flowjo v10.1.

### Image Analysis and Quantification

For detailed description of Image analysis and quantification, see Supplemental Experimental Procedures.

## Author contributions

M.E.T. conceived of the project with input from R.D.V.; X.Y., T.A.H., and M.E.T. performed the experiments and analyzed the data. All authors interpreted the results. X.Y. developed the mathematical model. X.Y., M.E.T., and R.D.V. wrote the manuscript with input from T.A.H.

## Supplemental Information

Document S1. Supplemental Experimental Procedures

Movie S1. Visualizing Translation of Single mRNAs in Live Cells, Related to Figure 1 A U2OS cell expressing scFv-GFP and PP7-3xmCherry was transfected with a translation reporter (SunTag_24x_-Kif18b-PP7_24x_). Images were acquired every 30 s (movie duration is 16 min) on a spinning disk confocal microscope. Red and green foci do not perfectly overlap because two color images were acquired sequentially and foci move rapidly in the cell. Movie field of view is 41 × 41 μm.

Movie S2. GFP Foci Represent Sites of Translation, Related to Figure 1 A U2OS cell expressing scFv-GFP and PP7-3xmCherry was transfected with a translation reporter (SunTag_24x_-Kif18b-PP7_24x_). Images were acquired every 30 s (movie duration is 25 min) on a spinning disk confocal microscope. At frame 8 (3.5 min after the start of the movie), 100 μg/mL puromycin is added to the cell culture medium. Bright scFv-GFP spots can be observed at the beginning of the movie, which rapidly disappear upon puromycin addition, indicating that they are sites of translation. Red and green foci do not perfectly overlap because two color images were acquired sequentially and foci move rapidly in the cell. Movie field of view is 41 × 41 μm.

Movie S3. Visualizing Translation of Membrane-Tethered mRNAs, Related to Figure 1 A U2OS cell expressing scFv-GFP (green) and PP7-2xmCherry-CAAX (red) was transfected with a translation reporter (SunTag_24x_-Kif18b-PP7_24x_). Images were acquired every 60 s (movie duration is 70 min) on a spinning disk confocal microscope focusing near the bottom plasma membrane of the cell. Individual mRNAs can be tracked for the duration of the movie, undergoing many rounds of translation. Movie field of view is 41 × 41 μm.

Movie S4. Ribosome Translocation Dynamics on Single mRNAs, Related to Figure 2 A U2OS cell expressing scFv-GFP (green) and PP7-2xmCherry-CAAX (not shown) was transfected with a translation reporter (SunTag_24x_-Kif18b-PP7_24x_). Images were acquired every 30 s (movie duration is 45 min) on a spinning disk confocal microscope focusing near the bottom plasma membrane of the cell. Harringtonine was added 2 min after the start of the movie. Bright green dots are translation sites, which become progressively dimmer after harringtonine addition due to ribosome runoff. Movie field of view is 40 × 40 μm.

Movie S5. Shutdown of Translation of a Single mRNA Molecule, Related to Figure 3 A U2OS cell expressing scFv-GFP (green) and PP7-2xmCherry-CAAX (red) was transfected with a translation reporter (SunTag_24x_-Kif18b-PP7_24x_). Images were acquired every 30 s (movie duration is 25 min) on a spinning disk confocal microscope focusing near the bottom plasma membrane of the cell. Translation on the mRNA molecule is abruptly shut down. Movie field of view is 6.6 × 6.6 μm.

Movie S6. Polysome Build-Up on Newly Transcribed mRNAs, Related to Figure 4 A U2OS cell expressing scFv-GFP (green) and PP7-2xmCherry-CAAX (red) was transfected with a translation reporter (SunTag_24x_-Kif18b-PP7_24x_) under control of a doxycycline inducible promoter. Images were acquired every 30 s (movie duration is 16 min) on a spinning disk confocal microscope focusing near the bottom plasma membrane of the cell. Doxycycline was added approximately 20 min before the start of the movie to induce transcription of the reporter gene. Several new red spots appear during the movie, which are likely newly transcribed mRNAs. These mRNAs rapidly initiate translation. Movie field of view is 8.4 × 8.4 μm.

Movie S7. Observing Single Ribosomes Translating an mRNA Molecule, Related to Figure 7 A U2OS cell expressing scFv-GFP (green) and PP7-2xmCherry-CAAX (red) is transfected with a translation reporter fused to the Emi1 5′ UTR_long that strongly represses translation initiation (5′ UTR_long-SunTag_24x_-Kif18b-PP7_24x_). Images were acquired every 30 s (movie duration is 31 min) on a spinning disk confocal microscope focusing near the bottom plasma membrane of the cell. Brief flashes of green can be observed on individual mRNA molecules, indicating a single ribosome translating the mRNA molecule. An offset was applied to the images to reduce the GFP background to allow easier detection of single ribosome transits. Movie field of view is 3.4 × 3.4 μm.

Document S2. Article plus Supplemental Information
