# Supplementary material for: Dynamics of Translation of Single mRNA Molecules In Vivo
Source: Cell. 2016 May 5;165(4):976–89. doi: 10.1016/j.cell.2016.04.034 (PMC4889334; doi:10.1016/j.cell.2016.04.034)
Supplement: Document S1. Supplemental Experimental Procedures [file mmc1.pdf]

Cell, Volume *165*

Supplemental Information

Dynamics of Translation of Single mRNA Molecules In Vivo

Xiaowei Yan, Tim A. Hoek, Ronald D. Vale, and Marvin E. Tanenbaum

Extended Experimental Procedures

General considerations for quantifying translation based on scFv-GFP fluorescence intensity values

In this study, we provide estimates for the number of ribosomes per mRNA, reveal ribosome initiation and translocation rates and provide measurements of ribosome stalling. All these values were calculated based on fluorescence intensities of the scFv-GFP antibody bound to nascent polypeptides. It is important to note that such fluorescence intensities by themselves do not provide quantitative information, but only can be interpreted after taking several factors into consideration.

1) Ribosome position along the mRNA.

Ribosome position along the mRNA affects the number of SunTag peptides that have been synthesized, and thus the amount of scFv-GFP fluorescence associated with that ribosome/nascent polypeptide. We have generated a mathematical model to correct for this effect (see the section "Modeling of the fluorescence intensity of translation sites" below, and Figure S7). It is important to note that this model assumes a homogeneous distribution of ribosomes along the mRNA which is likely accurate when averaging many mRNAs, but stochastic distributions can occur on an individual mRNA molecule. Also, non-homogenous distribution of ribosomes will not bias the estimate of ribosome number in ensemble statistics, as it is equally likely to underestimate or to overestimate ribosome number.

2) Variability in the number of scFv-GFPs bound to a SunTag peptide array.

The fluorescence intensity of a nascent polypeptide depends on the number of scFv-GFP molecules bound. The number of scFv-GFP antibody molecules bound to a single SunTag polypeptide can, however, vary. This variation may be limited though, as binding of the scFv-GFP to the 24 peptide SunTag array appears to saturate when sufficient cytoplasmic scFv-GFP is present, resulting in the binding of close to 24 scFv-GFPs per SunTag_{24x} array (Tanenbaum et al., 2014). Variation in the number of scFv-GFPs per SunTag_{24x} array will not affect the calculation

of the number of ribosomes per mRNA, as this variation will occur on both the nascent polypeptides and the fully synthesized and released SunTag protein in the same cells which is used to normalize the fluorescence intensity of the translation sites. However, when a lot of $SunTag_{24x}$ molecules have been synthesized and released into the cytoplasm, they can sequester the free scFv-GFP, leaving insufficient free scFv-GFP to bind all newly synthesized nascent SunTag peptides. Indeed, we have observed that at high expression levels of the reporter, translation sites become progressively dimmer after several hours, indicative of cytoplasmic depletion of the scFv-GFP. While such depletion of cytoplasmic scFv results both in dimmer translation sites and dimmer fully synthesized SunTag protein (used for normalization), and therefore does not affect our calculations of ribosome number per mRNA. Nonetheless, to circumvent this issue, we have used an inducible promoter to initiate reporter expression only ~1 hr before image acquisition. An additional source of variability in scFv-GFP binding to the SunTag peptides is introduced by the lag time that exists between synthesis of a new SunTag peptide and binding to the scFv-GFP. However, our experiments show that this lag time likely does not substantially decrease overall fluorescence intensity of the translation site, indicating that the binding reaction is relatively fast. This lag time likely depends on the level of free cytoplasmic scFv-GFP, and could therefore vary under different conditions.

3) Fluorescence decay of the scFv-GFP signal at translation sites.

In several experiments, including cases of natural translation shutdown and harringtonine ribosome run-off experiments, we observed a reduction in scFv-GFP fluorescence intensity at translation sites. We generally interpret this fluorescence decay as ribosomes terminating translation at the stop codon, which results in nascent chain release and ribosome recycling. While this conclusion is likely valid in most cases, alternative explanations are possible. For example, ribosomes stall for extended periods of time on mRNAs with chemical damage or on the Xbp1 nucleotide pausing sequence, but eventually scFv-GFP signal associated with stalled translation sites does decrease. While it is possible that ribosomes eventually normally read through such stall sequences and terminate normally at a stop codon, it is also possible that ribosomes are removed from mRNAs after a prolonged stalling event. Additional experiments

are required to distinguish these events, for example by placing the stall-inducing sequence upstream of the SunTag peptide array.

4) Photobleaching

Photobleaching rates were determined by measuring the total GFP signal of the entire cell over time. In experiments where photobleaching resulted in a fluorescence decrease of >10% of the initial fluorescence intensity, fluorescence intensities of translation sites were corrected for photobleaching.

Calculation of Ribosome Elongation

Ribosome elongation rates were determined by three different methods.

Population measurements from harringtonine ribosome run-off (Related to Figure 2)

The linear phase of the decrease in GFP fluorescence after new ribosome translation is prevented by addition of harringtonine provides information on the rate of ribosome movement along the mRNA. A model for the fluorescence signal is provided below (See Modeling of the fluorescence intensity of translation sites) along with details of how the data was fit to determine the elongation rate.

Calculating the ribosome elongation rate based on single mRNA run-off times (Related to figure S2E)

The total time required for run-off of all ribosomes from individual mRNAs represents the time until the last (*i.e.* most 5') ribosome completes translation after harringtonine treatment. Individual translation site intensities were quantified using the spot_counter ImageJ plugin (http://fiji.sc/SpotCounter) developed by Nico Stuurman, which draws a box around the selected translation site and scores the number of time-points that the translation site was detected. Automatic tracking was manually curated for all spots to ensure high quality tracking data. The average run-off time for all mRNAs was then determined, and we subtracted 60 s from this time, the time required for harringtonine to enter the cell (Ingolia et al., 2011). We

then divided the reporter length (1462 codons for the $SunTag_{24x}$-Kif18b-$PP7_{24x}$ reporter) by the corrected run-off time to obtain the ribosome elongation rate. This rate assumes that the final ribosome loaded near the 5' end of the mRNA, which may not be true for all mRNAs. This assumption may slightly overestimate ribosome elongation rates, as the actual distance covered by the most 5' ribosome covers until completion of translation will be slightly less than 1462 codons, if it is downstream of the start codon at the time of harringtonine addition. However, considering that most mRNAs have ~20 ribosomes and the average inter-ribosomal distance is therefore ~70 codons, the most 5' ribosome will usually be within 70 codons of the start site, which represents a 5% error.

Determining the ribosome elongation rate from single ribosome tracking data (Related to Figure 7).

By limiting translation initiation using the Emi1 5'UTR_long fused to the translation reporter, individual ribosomes could be observed decoding an mRNA molecule. For each single ribosome translation event, we measured the duration of the event, by determining the first time-point that GFP could reliably be detected over the background until the GFP signal disappeared. Only tracks were included that lasted less than 12 min. However, determining ribosome elongation rate based on such measurements was non-trivial, as this requires knowledge of the precise position of the ribosome along the transcript when the nascent chain signal is first detected; presumably a number of SunTag peptides need to have been synthesized before sufficient signal has accumulated to allow detection. We therefore needed to identify the fluorescence intensity detection limit. To accomplish this, we first determined the maximal fluorescence intensity associated with a single ribosomes; this occurs when the entire SunTag peptide array has been synthesized (Figure 7C). Maximal intensity was calculated by aligning all of the traces (n = 44) at the last time point in which fluorescence was observed and averaging the fluorescence intensity of the last 3 time points before polypeptide dissociation. We then aligned all 44 traces at the first point we could visually detect fluorescence and found that we could reliably detect signal from single ribosomes when their nascent chain-associated fluorescence was ~1/3 of the maximal intensity (Figure 7B). From this, we infer that ~8 of the 24

SunTag peptides likely have been synthesized at this point in time, which places these ribosomes on codon 245 (taking into account that SunTag peptides will not be labeled when inside the ribosome exit tunnel). Therefore, from the time of first fluorescence measurement to the release of the completed polypeptide on the stop codon (406 s on average for n = 44 individual ribosome translocations), the ribosome moves 1462-245 = 1217 codons (since the whole transcript is 1462 codons long). The ribosome elongation rate can be calculated as the number of codons / time: 1217/406 = 3.0 codons/s.

Translation Initiation Rate

Estimation of steady state initiation rate

To maintain a constant ribosome occupancy, the rate of removal of ribosomes from the mRNA molecule after completion of translation, must be balanced by new ribosome loading on the mRNA through translation initiation. To estimate the translation initiation rate, we first calculated the average inter-ribosomal distance, which is 58-146 codons assuming a ribosome number per mRNA of 10-25 ribosomes on the $SunTag_{24x}$-Kif18b reporter. Considering the ribosome elongation rate of 3.5 codons/s (for the Kif18b reporter), a ribosome will complete translation on average every 58/3.5 to 146/3.5 = 17 to 42 s. Thus, if constant ribosome occupancy is maintained, the initiation rate must be between 1/17 s^{-1} to 1/42 s^{-1} or 1.4 to 3.6 min^{-1}. These values represent average initiation rates, as the initiation rate on single mRNA molecules will vary over time.

Comparing the translation initiation rate on newly transcribed versus polysomal mRNAs.

Through the experiments in which single translating ribosomes were tracked, we determined that ribosomes could be first detected when ~1/3 of SunTag peptides had been synthesized (when the ribosome is positioned around codon 245 (see above)). Thus, when the first, pioneer ribosome was detected on a newly transcribed mRNA molecule, it was likely near this position. After initial fluorescence detection of the pioneer ribosome, translation site signal increased continuously for several min (Figure 4B). This fluorescence increase was presumably both due to the increase in the number of synthesized SunTag peptides on the pioneer ribosome, as well

as due to additional ribosomes that initiated after the pioneer ribosome. The translation site fluorescence intensity reached a steady state ~6 min after the initial detection of fluorescence (Figure 4B), indicating that the ribosome density remained constant from that point on. At a translocation rate of 3-3.5 codons/s, it would take the pioneer ribosome ~6 min to reach the stop codon from the position of initial detection (codon 245). From these calculations, we conclude that the ribosome density on the mRNA has already reached its maximal value as soon as the pioneer ribosome completes translation. Since ribosome density is a function of ribosome initiation rate and elongation rate, we infer that the initiation rate on the newly transcribed mRNA must be identical to that on mRNAs with established polysomes, assuming that the elongation rates are on average similar.

Estimation of Ribosome Number per mRNA

To obtain the number of ribosomes per mRNA molecule, we set up a normalization experiment to compare the average intensity of the single translation sites with the intensity coming from single, fully synthesized $\text{SunTag}_{24x\text{-}}$Kif18b proteins (Figure S2A). As the fully synthesized SunTag-Kif18b proteins encompass 24 copies of the SunTag peptide, their intensity should be comparable with the fluorescence intensity associated with a ribosome at the 3' end on the mRNA. However, the ribosomes at the 5' end of the mRNA will be much dimmer because they have not fully synthesized the SunTag epitopes. Thus, dividing the translation site intensity by the intensity from a single fully synthesized SunTag-Kif18b protein will underestimate the actual number of ribosomes on an mRNA. A correction factor derived from our model presented below was used to obtain a more accurate estimate of ribosome number.

Modeling of the fluorescence intensity of translation sites

Every active translation site is composed of one single mRNA with multiple ribosomes undergoing translation. The SunTag peptides produced from ribosomes are bound by scFv-GFP antibodies floating in the cytoplasm, giving rise to a fluorescence signal from the translation complex. Ribosomes on the 5' end of the mRNA are still translating part of the SunTag peptide, thus resulting in a partial fluorescence signal from the emerging polypeptide chain, while

ribosomes that are translocating on the 3' end of the gene have already synthesized the entire SunTag peptide array and are fully covered by the antibodies. Therefore, to interpret intensity changes from a transcript loaded with multiple ribosomes, we have generated a model that takes into account ribosome location as well as density on the mRNA as a function of time.

The intensity from a single ribosome will increase gradually as the ribosome moves towards the 3' end until it reaches a plateau where all SunTag peptides are synthesized (Figure S7A). This relationship is described as following:

$$f(x) = \begin{cases} g(x) & x \in [0, L_1) \\ I_{sun} & x \in [L_1, L] \end{cases} \quad \text{.. (1)}$$

The parenthesis and square bracket are notations for intervals. The two numbers are the endpoints of the interval. Parenthesis indicates exclusion of corresponding endpoint while square bracket indicates inclusion of it.

$f(x)$: intensity from a single ribosome at position x

$g(x)$: intensity from a single ribosome at position x when x is in between 0 and L_1, simplified as a linear function in the diagram in Figure S7A

x : ribosome position on the mRNA at time t

L : the length of positions that can be covered by ribosomes, which includes the open reading frame decoding both the SunTag peptides and the gene of interest (GOI), as shown in Figure S7A

L_1 : position on the transcript where intensity from a ribosome at that position reaches plateau intensity, as shown in Figure S7A

I_{sun} : the intensity of a single SunTag array that is fully covered by scFv-GFP, with the intensity depending on the number of SunTag peptides fused to the gene of interest, multiplied by the intensity of a single scFv-GFP antibody

$$I_{sun} = n_{sun} \, x \, i_{GFP} \quad \text{.. (2)}$$

n_{sun} : the number of SunTag peptides which are fused to the gene of interest (mostly 24 in this study)

i_{GFP} : the intensity of a single scFv-GFP

Thus the average intensity from a single translation site could therefore be described using the following formula:

$$F(t) = \int_0^L f(x) \cdot R(x,t) \cdot dx \quad \text{...} \quad (3)$$

$F(t)$: average fluorescence intensity from a single translation site at time t

$R(x,t)$: ribosome probability density at position x at time t.

With the assumption that ribosomes are randomly positioned in a population of transcripts (while this distribution may not be homogeneous on a single mRNA, it will approximate a random distribution when averaging hundreds of mRNAs, as is done in these experiments; thus this model is only accurate when a large number of mRNAs is analyzed), the probability density will be the same at different positions on the transcript at steady state (Figure S7B, left), which leads to:

$$R(x,t) = R_s$$

$$R_s = \frac{n_r}{L_r} \quad \text{...} \quad (4)$$

R_s : ribosome density during steady state

n_r : the number of ribosomes on a single transcript

L_r : the length of transcript that is covered by the ribosomes

Modeling of shutdown process and elongation rate

Based on the above, we are able to model fluorescence intensity as a function of time. We use the translation shutdown process as an example:

When new ribosomes are no longer added at the 5' end (*e.g.* when initiation is blocked by harringtonine), previously bound ribosomes will run off the transcript from the 5' to the 3' end. This will change the ribosome distribution as a function of time t, as described by the following equations and the illustration depicted in Figure S7B:

$$R(x,t) = \begin{cases} 0 & x \in [0, v_e \cdot t) \\ R_s & x \in [v_e \cdot t, L] \end{cases}$$

………………………………………… (5)

v_e : elongation rate; assumed to be a constant in the model.

If we incorporate this function into equation (3), we derive the following formula, which describes the change in intensity of a single translation site as a function of time ($t = 0$ marking the beginning of harringtonine treatment) (Figure S7C).

$$F(t) = \begin{cases} R_s \cdot \left[\int_{v_e \cdot t}^{L_1} g(x) \cdot dx + I_{sun}(L - L_1)\right] & t \in \left[0, \frac{L_1}{v_e}\right) \quad 1) \\ R_s \cdot I_{sun}(L - v_e \cdot t) & t \in \left[\frac{L_1}{v_e}, \frac{L}{v_e}\right) \quad 2) \\ 0 & t \in \left[\frac{L}{v_e}, \infty\right) \quad 3) \end{cases} \quad \text{…………………… (6)}$$

This formula describes absolute intensity, which is dependent on many variables including the laser power used to excite the fluorophore. Therefore, we normalize this equation to the initial steady state fluorescence using the constant C_1:

$$F_s = R_s \cdot \left[\int_0^{L_1} g(x) \cdot dx + I_{sun}(L - L_1)\right] = R_s \cdot C_1 \quad \text{…………………… (7)}$$

$$F_n(t) = {F(t)}/{F_s} = \begin{cases} \left[\int_{v_e \cdot t}^{L_1} g(x) \cdot dx + I_{sun}(L - L_1)\right] / C_1 & t \in \left[0, \frac{L_1}{v_e}\right) \quad 1) \\ I_{sun}(L - v_e \cdot t) / C_1 & t \in \left[\frac{L_1}{v_e}, \frac{L}{v_e}\right) \quad 2) \\ 0 & t \in \left[\frac{L}{v_e}, \infty\right) \quad 3) \end{cases} \quad \text{………… (8)}$$

F_s: translation site intensity during steady state

C_1 : constant number, $C_1 = \int_0^{L_1} g(x) \cdot dx + I_{sun}(L - L_1)$

To further simplify the model, a linear function was used to describe $g(x)$, and the results are shown in Figure S7D. Data acquired in real experiments have stalling events, which complicates fitting the data to the simulated results. To simplify the determination of the elongation rate from the harringtonine run-off experiments, we only fit the second stage of the curve (Figure S7C), which is given by:

$$F_n(t) = {F(t)}/{F_s} = {I_{sun}(L - v_e \cdot t)}/{C_1} = -\frac{1}{C} \cdot v_e \cdot t + \frac{L}{C} \quad \text{.....................} \quad (9)$$

C : constant number, $C = {C_1}/{I_{sun}}$

The first order derivative of this stage will give us the elongation rate v_e:

$$\frac{dF_n(t)}{dt} = -\frac{1}{C} \cdot v_e \quad \text{..} \quad (10)$$

Intensity from stalled or slowly elongating translation sites contributes about 10-20% of the initial fluorescence intensity (Figure 2D), which will lead to underestimation of the slope without correction. To overcome this, a linear regression was fit to the second half of the curve after initial run-off, and the intercept value was subtracted to exclude the influence of stalled translation sites. The curve was normalized after correction, and relative fluorescence intensity in between 40-80% was fit with a linear function to extract the slope. Elongation rate was calculated using the formula mentioned above. However, several more corrections were applied to the previous equation to account for the influence of 1) the codons translated before the SunTag array (23 aa) as well as the codons in between the SunTag array and the GOI (6 aa) 2) the nascent peptide buried in the ribosome exit tunnel (36 aa was used).

Modeling of the number of ribosomes on a single mRNA

Fully synthesized SunTag_{24x}-Kif18b proteins encompass 24 copies of the SunTag peptide, so their intensity should be comparable to the fluorescence intensity of a ribosome at the 3' end of the mRNA. However, ribosomes at the 5' end of the mRNA will be dimmer, since they have not fully synthesized the SunTag epitopes. Thus, dividing the translation site intensity by the intensity of a single fully synthesized SunTag_{24x}-Kif18b protein will underestimate the actual number of ribosomes on an mRNA.

To overcome this, a correction factor γ was applied. As described earlier in equation (4), ribosome density R_s is:

$$R_s = \frac{n_r}{L_r}$$

If we assume the transcript is fully covered by the ribosomes during steady state, the ribosome number n_r can be derived as:

$$n_r = R_s \cdot L_r = R_s \cdot L = {(F_s \cdot L)}/{C_1} = \gamma \cdot {F_s}/{I_{sun}} \quad \text{......................................} \quad (11)$$

The constant $\gamma = {2L}/{(2L - L_1)}$ when a linear function was used to describe $g(x)$. As discussed in the previous section, more corrections were applied to the equation to account for influence of things such as ribosome exit tunnel length. Thus, the actual correction factor used for the Kif18b reporter is 1.32 (1/0.76).

Plasmid sequences

SunTag$_{24x}$-Kif18b-PP7$_{24x}$ **-** Complete plasmid sequence

gacggatcgggagatctcccgatcccctatggtgcactctcagtacaatctgctctgatgccgcatagttaagccagtatctgctccctgcttgtgtgttg
gaggtcgctgagtagtgcgcgagcaaaatttaagctacaacaaggcaaggcttgaccgacaattgcatgaagaatctgcttagggttaggcgttttgc
gctgcttcgcgatgtacgggccagatatacgcgttgacattgattattgactagttattaatagtaatcaattacggggtcattagttcatagcccatata
tggagttccgcgttacataacttacggtaaatggcccgcctggctgaccgcccaacgacccccgcccattgacgtcaataatgacgtatgttcccatag
taacgccaatagggactttccattgacgtcaatgggtggagtatttacggtaaactgcccacttggcagtacatcaagtgtatcatatgccaagtacgc
cccctattgacgtcaatgacggtaaatggcccgcctggcattatgcccagtacatgaccttatgggactttcctacttggcagtacatctacgtattagtc
atcgctattaccatggtgatgcggttttggcagtacatcaatgggcgtggatagcggtttgactcacggggatttccaagtctccaccccattgacgtca
GCTCAGCTTTCTAGACCGCGcgcaaatgggcggtaggcgtgtacggtgggaggtctatataagcagagctctccctatcagtgatagagatc
tccctatcagtgatagagatcgtcgacgagctcgtttagtgaaccgtcagatcgcctggagacgccatccacgctgttttgacctccatagaagacacc
gggaccgatccagcctccggactctagcgtttaaacttAAGCTTGCCACCATGGGCGCCATCATACACGCGGCCGCAATCATcggt
ccgGGTGGATCTGGAGGTGGAGGTTCTGGAGGAGAAGAACTTTTGAGCAAGAATTATCATCTTGAGAACGAAGTG
GCTCGTCTTAAGAAAGGTTCTGGCAGTGGAGAAGAACTGCTTTCAAAGAATTACCACCTGGAAAATGAGGTAGCT
AGACTGAAAAAGGGGAGCGGAAGTGGGGAGGAGTTGCTGAGCAAAAATTATCATTTGGAGAACGAAGTAGCAC
GACTAAAGAAAGGGTCCGGATCGGGTGAGGAGTTACTCTCGAAAAATTATCATCTCGAAAACGAAGTGGCTCGGC
TAAAAAAGGGCAGTGGTTCTGGAGAAGAGCTATTATCTAAAAACTACCACCTCGAAAATGAGGTGGCACGCTTAA
AAAAGGGAAGTGGCAGTGGTGAAGAGCTACTATCCAAGAATTATCATCTTGAGAACGAGGTAGCGCGTTTGAAG
AAGGGTTCCGGCTCAGGAGAGGAACTGCTCTCGAAGAACTATCATCTTGAAAATGAGGTCGCTCGATTAAAAAAG
GGATCGGGCAGTGGTGAGGAACTACTTTCAAAGAATTACCACCTCGAAAACGAAGTAGCTCGATTAAAGAAAGGT
TCAGGGTCGGGTGAAGAATTACTGAGTAAAAATTATCATCTGGAAAATGAGGTAGCGAGACTAAAAAAGGGGAG
TGGTTCTGGCGAAGAGTTGCTATCGAAAAATTATCATCTTGAGAACGAAGTTGCTAGGCTCAAAAAGGGCTCAGG
CTCAGGCGAGGAGTTGCTCTCGAAAAACTACCACTTGGAAAATGAGGTCGCGAGGTTGAAAAAGGGGAGCGGGT
CGGGCGAGGAGTTATTGAGCAAAAACTATCATTTAGAGAACGAAGTCGCGCGCTTAAAGAAAGGCTCGGGCTCG
GGCGAAGAACTCTTATCGAAGAACTACCACCTCGAAAATGAGGTCGCCAGGTTGAAAAAGGGCAGTGGCAGCGG
GGAGGAACTCTTGAGCAAGAACTACCACTTGGAGAATGAGGTCGCGAGATTGAAGAAAGGGTCGGGGAGCGGC
GAGGAATTGCTCAGCAAGAATTATCATTTGGAGAACGAAGTCGCCAGGCTCAAGAAAGGCTCGGGGTCGGGGGA
GGAATTGTTGAGTAAAAACTACCACTTGGAAAATGAAGTCGCCAGGCTCAAAAAAGGGAGTGGGAGCGGCGAAG
AGTTATTGAGCAAAAATTACCACTTGGAGAACGAAGTGGCAAGGCTCAAGAAAGGGAGCGGCAGCGGGGAGGA
GCTCTTATCGAAGAACTACCACTTAGAGAATGAAGTCGCCCGCTTGAAGAAAGGCTCGGGGAGCGGGGAAGAGC
TCTTGAGCAAGAACTACCACTTGGAAAATGAGGTGGCGCGCTTGAAGAAAGGGAGCGGGAGCGGGGAAGAGTT
ACTATCTAAGAATTATCATCTCGAGAACGAGGTGGCTCGACTAAAGAAGGGCTCCGGCAGTGGGGAGGAACTCCT
GTCGAAGAACTATCATCTTGAAAATGAGGTTGCAAGACTTAAAAAGGGGTCCGGATCAGGTGAGGAACTACTCAG
TAAGAATTACCACCTGGAAAACGAAGTTGCACGTTTGAAGAAAGGATCAGGATCAGGCGAAGAACTGCTCTCAAA
AGATTATCATTTGGAAAATGAGGTTGCACGTTTAAAAAAGGGAAGTGGCAGTGGTGAGGAACTTCTGTCGAAAAA
TTATCATCTCGAGAATGAAGTAGCCCGACTTAAAAAGGGTTCTGGCTCGGGTCAGCGGCCGCACCGGTCAGCAGT
GGAGGACAGCACGCTGCAAGTAGTGGTACGGGTGCGGCCCCCACCCCTCGGGAGCTGGACAGTCAGCGGCGGC
CAGTGGTTCAGGTGGTGGACGAGCGGGTGCTGGTGTTTAACCCTGAGGAGCCCGATGGAGGGTTCCCTGGCCTG
AAATGGGGTGGCACCCATGATGGCCCCAAGAAGAAGGGCAAAGACCTGACGTTTGTCTTTGACCGGGTCTTTGGC
GAGGCGGCCACCCAACAGGACGTGTTCCAGCACACCACGCACAGCGTCCTGGACAGCTTCCTCCAGGGCTACAAC
TGCTCAGTGTTTGCCTACGGGGCCACCGGGGCTGGGAAGACACACACCATGCTGGGAAGGGAGGGGGACCCCG
GCATCATGTACCTGACCACCGTGGAACTGTACAGGCGCCTGGAGGCCCGCCAGCAGGAGAAGCACTTCGAGGTG
CTCATCAGCTACCAGGAGGTGTATAATGAACAGATCCATGACCTCCTGGAGCCCAAGGGGCCCCTTGCCATCCGC
GAGGACCCCGACAAGGGGGTGGTGGTGCAAGGACTTTCTTTCCACCAGCCAGCCTCAGCCGAGCAGCTGCTGGA
GATACTGACCAGGGGGAACCGTAACCGCACGCAGCACCCCACTGATGCCAACGCGACTTCCTCCCGCTCCCATGCC

ATCTTCCAGATCTTTGTGAAGCAGCAGGACCGGGTTCCAGGACTGACCCAGGCTGTCCAGGTGGCCAAGATGAGC
CTGATTGACCTGGCTGGCTCAGAGCGGGCATCCAGCACCCATGCGAAGGGGGAGCGGCTGCGGGAGGGGGCCA
ACATCAACCGCTCTCTGCTGGCGCTCATCAACGTCCTCAATGCCTTGGCCGATGCAAAGGGCCGCAAGACCGCTGT
GCCCTACGCGGACAGCGCACTGACCCGCCTGCTCAAAGACTCCCTCGGGGGCAACTGCCGCACAGTGATGATCGC
TGCCATCAGCCCCTCCAGCCTGACCTACGAGGACACGTATAATACCCTCAAATATGCCGACCGGGCCAAGGAGATC
AGGCTCTCGCTGAAGAGCAATGTGACCAGCCTGGACTGTCACATCAGCCAGTATGCTACCATCTGCCAACAGCTCC
AGGCTGAGGTAGCCGCTCTGAGGAAGAAGCTCCAAGTGTATGAGGGGGGAGGCCAGCCCCCACCACAGGACCTC
CCAGGATCTCCCAAGTCGGGACCACCACCAGAACACCTTCCCAGCTCCCCCTTGCCACCCCACCCTCCCAGCCAGCC
CTGCACCCCAGAGCTCCCTGCAGGGCCTAGAGCCCTTCAAGAGGAGAGTCTGGGGATGGAGGCCCAGGTGGAGA
GGGCCATGGAAGGGAACTCTTCAGACCAGGAGCAGTCCCCAGAGGATGAGGATGAAGGCCCAGCTGAGGAGGT
TCCAACCCAGATGCCAGAGCAGAACCCCACACATGCACTGCCAGAGTCCCCTCGCCTGACCCTGCAGCCCAAGCCA
GTCGTGGGCCACTTCTCAGCACGGGAACTGGATGGGGACCGTTCTAAGCGGTTGGCCCTAAAGGTGCTGTGCGTT
GCCCAGCGGCAGTACTCCCTGCTCCAAGCAGCCAACCTCCTGACGCCCGACATGATCACAGAGTTTGAGACCCTAC
AGCAGCTGGTGCAAGAGGAAAAAATTGAGCCTGGGGCAGAGGCCTTGAGGACTTCAGGCCTGGCCAGGGGGGC
ACCTCTGGCTCAGGAGCTGTGTTCAGAGTCAAAGCCTCCAGGATACACTGGCCCTGTGACCCGGACTATGGCGAG
GCGACTGAGTGGCCCCCTGCACACCCTGGGAATCCCGCCTGGACCCAACTGCACCCCAGCCCAGGGGTCCCGATG
GCCCATGGAGAAGAAGAGGAGGAGACCAAGCGCCTTGGAGGCAGACAGTCCCATGGCCCCAAAGCGGGGCACC
AAGCGCCAGCGCCAGTCCTTCCTGCCCTGCCTAAGGAGAGGGTCTCTGCCTGACACCCAACCTTCACAGGGGCCCA
GCACCCCCAAAGGAGAAAGGGCCTCCTCCCCCTGCCATTCCCCTCGCGTTTGCCCAGCCACAGTCATCAAAAGCCG
GGTGCCCCTGGGCCCTTCCGCCATGCAGAACTGCTCCACCCCGCTGGCTCTGCCCACTCGAGACCTCAATGCCACC
TTTGATCTCTCTGAGGAGCCTCCCTCAAAGCCCAGTTTCCATGAATGCATTGGCTGGGACAAAATACCCCAGGAGC
TGAGCAGGCTGGACCAGCCCTTCATCCCCAGGGCACCTGTGCCCCTGTTCACCATGAAGGGCCCCAAGCCAACATC
TTCCCTCCCTGGGACCTCTGCCTGCAAGAAGAAGCGCGTTGCGAGTTCCTCAGTCTCCCATGGCCGCAGCCGCATC
GCCCGCCTCCCCAGCAGCACTTTGAAGAGGCCAGCTGGGCCCCTTGTACTCCCAGAGCTGCCCTTGAGTCCCCTGT
GCCCTAGCAACCGGAGGAATGGAAAGGACCTCATCAGGGTGGGGAGAGCACTCTCAGCAGGGAACGGCGTCACC
AAGGTGTCCGATATCTAACGACACGATTCGAAGATCGCATCGCTAGCCCGGTTGTACAGGTACCGGATCCTAAGG
TACCTAATTGCCTAGAAAGGAGCAGACGATATGGCGTCGCTCCCTGCAGGTCGACTCTAGAAACCAGCAGAGCAT
ATGGGCTCGCTGGCTGCAGTATTCCCGGGTTCATTAGATCCTAAGGTACCTAATTGCCTAGAAAGGAGCAGACGA
TATGGCGTCGCTCCCTGCAGGTCGACTCTAGAAACCAGCAGAGCATATGGGCTCGCTGGCTGCAGTATTCCCGGG
TTCATTAGATCCTAAGGTACCTAATTGCCTAGAAAGGAGCAGACGATATGGCGTCGCTCCCTGCAGGTCGACTCTA
GAAACCAGCAGAGCATATGGGCTCGCTGGCTGCAGTATTCCCGGGTTCATTAGATCCTAAGGTACCTAATTGCCTA
GAAAGGAGCAGACGATATGGCGTCGCTCCCTGCAGGTCGACTCTAGAAACCAGCAGAGCATATGGGCTCGCTGG
CTGCAGTATTCCCGGGTTCATTAGATCCTAAGGTACCTAATTGCCTAGAAAGGAGCAGACGATATGGCGTCGCTCC
CTGCAGGTCGACTCTAGAAACCAGCAGAGCATATGGGCTCGCTGGCTGCAGTATTCCCGGGTTCATTAGATCCTAA
GGTACCTAATTGCCTAGAAAGGAGCAGACGATATGGCGTCGCTCCCTGCAGGTCGACTCTAGAAACCAGCAGAGC
ATATGGGCTCGCTGGCTGCAGTATTCCCGGGTTCATTAGATCCTAAGGTACCTAATTGCCTAGAAAGGAGCAGAC
GATATGGCGTCGCTCCCTGCAGGTCGACTCTAGAAACCAGCAGAGCATATGGGCTCGCTGGCTGCAGTATTCCCG
GGTTCATTAGATCCTAAGGTACCTAATTGCCTAGAAAGGAGCAGACGATATGGCGTCGCTCCCTGCAGGTCGACTC
TAGAAACCAGCAGAGCATATGGGCTCGCTGGCTGCAGTATTCCCGGGTTCATTAGATCCTAAGGTACCTAATTGCC
TAGAAAGGAGCAGACGATATGGCGTCGCTCCCTGCAGGTCGACTCTAGAAACCAGCAGAGCATATGGGCTCGCT
GGCTGCAGTATTCCCGGGTTCATTAGATCCTAAGGTACCTAATTGCCTAGAAAGGAGCAGACGATATGGCGTCGC
TCCCTGCAGGTCGACTCTAGAAACCAGCAGAGCATATGGGCTCGCTGGCTGCAGTATTCCCGGGTTCATTAGATCC
TAAGGTACCTAATTGCCTAGAAAGGAGCAGACGATATGGCGTCGCTCCCTGCAGGTCGACTCTAGAAACCAGCAG
AGCATATGGGCTCGCTGGCTGCAGTATTCCCGGGTTCATTAGATCCTAAGGTACCTAATTGCCTAGAAAGGAGCA
GACGATATGGCGTCGCTCCCTGCAGGTCGACTCTAGAAACCAGCAGAGCATATGGGCTCGCTGGCTGCAGTATTC
CCGGGTTCATTAGATCTcgcgAAGGGCGAATTCacgaGGCGCGCCcggatcGCGATCGCaatcTTAATTAACTCGAGtct
agagggcccgtttaaacccgctgatcagcctcgactgtgccttctagttgccagccatctgttgtttgcccctcccccgtgccttccttgaccctggaagg
tgccactcccactgtcctttcctaataaaatgaggaaattgcatcgcattgtctgagtaggtgtcattctattctggggggtggggtggggcaggacagc

aagggggaggattgggaagacaatagcaggcatgctggggatgcggtgggctctatggcttctgaggcggaaagaaccagctggggctctagggggg
tatccccacgcgccctgtagcggcgcattaagcgcggcgggtgtggtggttacgcgcagcgtgaccgctacacttgccagcgccctagcgcccgctcct
ttcgctttcttcccttcctttctcgccacgttcgccggctttccccgtcaagctctaaatcgggggctccctttagggttccgatttagtgctttacggcacct
cgaccccaaaaaacttgattagggtgatggttcacgtagtgggccatcgccctgatagacggtttttcgccctttgacgttggagtccacgttctttaata
gtggactcttgttccaaactggaacaacactcaaccctatctcggtctattcttttgatttataagggattttgccgatttcggcctattggttaaaaaatg
agctgatttaacaaaaatttaacgcgaattaattctgtggAATGTGTGTCAGTTAGGGTGTGGAAAGTCCCCAGGCTCCCCAGCA
GGCAGAAGTATGCAAAGCATGCATCTCAATTAGTCAGCAACCAGGTGTGGAAAGTCCCCAGGCTCCCCAGCAGGC
AGAAGTATGCAAAGCATGCATCTCAATTAGTCAGCAACCATAGTCCCGCCCCTAACTCCGCCCATCCCGCCCCTAAC
TCCGCCCAGTTCCGCCCATTCTCCGCCCCATGGCTGACTAATTTTTTTTATTTATGCAGAGGCCGAGGCCGCCTCTG
CCTCTGAGCTATTCCAGAAGTAGTGAGGAGGCTTTTTTGGAGGCCTAGGCTTTTGCAAAAAGCTCCcgggagcttgtat
atccattttcggatctgatcagcacgtgttgacaattaatcatcggcatagtatatcggcatagtataatacgacaaggtgaggaactaaaccatggcc
aagttgaccagtgccgttccggtgctcaccgcgcgcgacgtcgccggagcggtcgagttctggaccgaccggctcgggttctcccgggacttcgtgga
ggacgacttcgccggtgtggtccgggacgacgtgaccctgttcatcagcgcggtccaggaccaggtggtgccggacaacaccctggcctgggtgtgg
gtgcgcggcctggacgagctgtacgccgagtggtcggaggtcgtgtccacgaacttccgggacgcctccgggccggccatgaccgagatcggcgagc
agccgtgggggcgggagttcgccctgcgcgacccggccggcaactgcgtgcacttcgtggccgaggagcaggactgacacgtgctacgagatttcga
ttccaccgccgccttctatgaaaggttgggcttcggaatcgttttccgggacgccggctggatgatcctccagcgcggggatctcatgctggagttcttc
gcccaccccaacttgtttattgcagcttataatggttacaaataaagcaatagcatcacaaatttcacaaataaagcatttttttcactgcattctagttg
tggtttgtccaaactcatcaatgtatcttatcatgtctgtataccgtcgacctctagctagagcttggcgtaatcatggtcatagctgtttcctgtgtgaaa
ttgttatccgctcacaattccacacaacatacgagccggaagcataaagtgtaaagcctggggtgcctaatgagtgagctaactcacattaattgcgtt
gcgctcactgcccgctttccagtcgggaaacctgtcgtgccagctgcattaatgaatcggccaacgcgcggggagaggcggtttgcgtattgggcgctc
ttccgcttcctcgctcactgactcgctgcgctcggtcgttcggctgcggcgagcggtatcagctcactcaaaggcggtaatacggttatccacagaatca
ggggataacgcaggaaagaacatgtgagcaaaaggccagcaaaaggccaggaaccgtaaaaaggccgcgttgctggcgtttttccataggctccgc
ccccctgacgagcatcacaaaaatcgacgctcaagtcagaggtggcgaaacccgacaggactataaagataccaggcgtttccccctggaagctccc
tcgtgcgctctcctgttccgaccctgccgcttaccggatacctgtccgcctttctcccttcgggaagcgtggcgctttctcatagctcacgctgtaggtatc
tcagttcggtgtaggtcgttcgctccaagctgggctgtgtgcacgaaccccccgttcagcccgaccgctgcgccttatccggtaactatcgtcttgagtcc
aacccggtaagacacgacttatcgccactggcagcagccactggtaacaggattagcagagcgaggtatgtaggcggtgctacagagttcttgaagt
ggtggcctaactacggctacactagaagaacagtatttggtatctgcgctctgctgaagccagttaccttcggaaaaagagttggtagctcttgatccg
gcaaacaaaccaccgctggtagcggtttttttgtttgcaagcagcagattacgcgcagaaaaaaaggatctcaagaagatcctttgatcttttctacgg
ggtctgacgctcagtggaacgaaaactcacgttaagggattttggtcatgagattatcaaaaaggatcttcacctagatccttttaaattaaaaatgaa
gttttaaatcaatctaaagtatatatgagtaaacttggtctgacagttaccaatgcttaatcagtgaggcacctatctcagcgatctgtctatttcgttcat
ccatagttgcctgactccccgtcgtgtagataactacgatacgggagggcttaccatctggccccagtgctgcaatgataccgcgagacccacgctcac
cggctccagatttatcagcaataaaccagccagccggaagggccgagcgcagaagtggtcctgcaactttatccgcctccatccagtctattaattgtt
gccgggaagctagagtaagtagttcgccagttaatagtttgcgcaacgttgttgccattgctacaggcatcgtggtgtcacgctcgtcgtttggtatggc
ttcattcagctccggttcccaacgatcaaggcgagttacatgatcccccatgttgtgcaaaaaagcggttagctccttcggtcctccgatcgttgtcaga
agtaagttggccgcagtgttatcactcatggttatggcagcactgcataattctcttactgtcatgccatccgtaagatgcttttctgtgactggtgagtac
tcaaccaagtcattctgagaatagtgtatgcggcgaccgagttgctcttgcccggcgtcaatacgggataataccgcgccacatagcagaactttaaa
agtgctcatcattggaaaacgttcttcggggcgaaaactctcaaggatcttaccgctgttgagatccagttcgatgtaacccactcgtgcacccaactg
atcttcagcatcttttactttcaccagcgtttctgggtgagcaaaaacaggaaggcaaaatgccgcaaaaaagggaataagggcgacacggaaatgt
tgaatactcatactcttcctttttcaatattattgaagcatttatcagggttattgtctcatgagcggatacatatttgaatgtatttagaaaaataaacaa
ataggggttccgcgcacatttccccgaaaagtgccacctgacgtc

BFP codon optimized sequence
TCGGAGCTAATAAAAGAGAACATGCATATGAAACTATACATGGAGGGTACGGTAGATAACCATCACTTCAAGTGC
ACGTCGGAAGGTGAGGGTAAGCCGTACGAGGGTACGCAAACGATGCGAATAAAAGTAGTTGAGGGTGGTCCGC
TACCGTTTGCGTTTGATATATTAGCGACGTCGTTTCTATACGGTTCGAAGACGTTTATAAATCACACGCAAGGTATA
CCGGATTTTTTTAAACAATCGTTCCCGGAAGGTTTCACGTGGGAGCGAGTAACGACGTATGAAGATGGTGGTGTA
TTAACGGCGACGCAAGATACGTCGTTACAAGACGGTTGCCTAATATACAATGTTAAGATACGAGGTGTTAACTTCA

CGTCGAACGGTCCGGTTATGCAAAAGAAGACGCTAGGTTGGGAAGCGTTTACGGAAACGTTATACCCGGCGGAT
GGTGGTTTAGAAGGTCGTAATGACATGGCGTTAAAATTAGTAGGTGGTTCGCACTTAATAGCGAACATAAAGACG
ACGTACCGTTCGAAAAAACCGGCGAAGAATCTAAAGATGCCGGGTGTATACTACGTAGATTACCGACTAGAACGT
ATAAAGGAGGCGAATAACGAGACGTACGTTGAACAACATGAAGTAGCGGTAGCGCGATACTGTGATTTACCGTC
GAAGCTAGGTCACAAACTAAAT

U2AF2 codon optimized sequence
tcCgaTttcgaTgagttcgagcggcagctcaacgagaataaacaagagcgggaTaaggagaaccggcatcggaagcgcagccacagccgctcta
gaagccgggaTcgcaaacgccggagccggagccgcgaccggcgcaaccgggaccagcggagcgcctcccgggacaggAGAAGAcgcagcaa
acctttgaccagaggcgctaaagaggagcacggtggactgattAGAtcccccagacacgagaagaagaagaaggtcAGAaaatactgggacgt
gccacccccaggctttgagcacatcaccccaatgcagtacaaggccatgcaagctgcTggtcagattccagccactgctcttctccccaccatgacccc
Agacggtctggccgtgacccctacgccggtgcccgtggtcgggagccagatgaccagacaagcccggcgcctctacgtgggcaacatccccttt ggc
atcactgaggaggccatgatggatttcttcaacgcccagatgcgcctgggAggActgacccaggcccctggcaacccagtgttggctgtgcagattaa
ccaggacaagaattttgcctttttggagttccgctcagtggaTgagactacccaggctatggcctttgatggcatcatcttccagggccagtcactGaa
gatccgcaggcctcacgaTtaccagccTcttcctggcatgtcagagaacccctccgtctatgtgcctggAgttgtgtccactgtggtccccgaTtctgc
ccacaagctgttcatcggAggcCTGcccaactacctgaacgatgaccaggtcaaagagctgctgacatcctttgggcccctcaaggccttcaacctg
gtcaaggacagtgccacgggActctccaagggctacgccttctgtgagtacgtggacatcaacgtcacggatcaggccattgcTgggctgaacggca
tgcagctgggAgataagaagctgctggtccagagggcTagtgtgggagccaagaatgccacgctgagcaccatcaatcagacgcctgtgaccctgc
aagtgccgggcttgatgagctcccaggtgcagatgggcggccacccTactgaggtcctgtgcctcatgaacatggtgctgcctgaggagctgctggac
gacgaggagtatgaggagatcgtggaggatgtgcgggacgagtgcagcaagtacgggcttgtcaagtccatcgagatcccccggcctgtggacggc
gtcgaggtgcccggctgcggaaagatctttgtggagttcacctctgtgtttgactgccagaaagccatgcagggcctgacgggccgcaagttcgccaa
cagagtggttgtcacaaaatactgtgaccccgactcttatcaccgccgggacttctgg

5x SunTag array
GAAGAACTTTTGAGCAAGAATTATCATCTTGAGAACGAAGTGGCTCGTCTTAAGAAAGGTTCTGGCAGTGGAGAA
GAACTGCTTTCAAAGAATTACCACCTGGAAAATGAGGTAGCTAGACTGAAAAAGGGGAGCGGAAGTGGGGAGG
AGTTGCTGAGCAAAAATTATCATTTGGAGAACGAAGTAGCACGACTAAAGAAAGGGTCCGGATCGGGTGAGGAG
TTACTCTCGAAAAATTATCATCTCGAAAACGAAGTGGCTCGGCTAAAAAAGGGCAGTGGTTCTGGAGAAGAGCTA
TTATCTAAAAACTACCACCTCGAAAATGAGGTGGCACGCTTAAAAAAGGGAAGTGGCAGTGGT

10x SunTag array
gaagaacttttgagcaagaattatcatcttgagaacgaagtggctcgtcttaagaaaggttctggcagtggagaagaactgctttcaaagaattacca
cctggaaaatgaggtagctagactgaaaaaggggagcggaagtggggaggagttgctgagcaaaaattatcatttggagaacgaagtagcacgac
taaagaaagggtccggatcgggtgaggagttactctcgaaaaattatcatctcgaaaacgaagtggctcggctaaaaaagggcagtggttctggaga
agagctattatctaaaaactaccacctcgaaaatgaggtggcacgcttaaaaaagggaagtggcagtggtgaagagctactatccaagaattatcat
cttgagaacgaggtagcgcgtttgaagaagggttccggctcaggagaggaactgctctcgaagaactatcatcttgaaaatgaggtcgctcgattaaa
aaagggatcgggcagtggtgaggaactactttcaaagaattaccacctcgaaaacgaagtagctcgattaaagaaaggttcagggtcgggtgaaga
attactgagtaaaaattatcatctggaaaatgaggtagcgagactaaaaaaggggagtggttctggcgaggaattgctatcgaaaaattatcatcttg
agaacgaagttgctaggctcaaaaagggctcaggctcaggc

Emi1 5’UTR_long
gttaggagggagagcagagcgcgcgcgtaaatcctagagaggcgggctaagctggactgggggagggtccgtcttccggaaagtctggattcccg
gacgagccgagttgctgctcaccgaactcccgttcgagagatgatcgaagaaagtcggctaccatttgtacccatcaaagatctccagatggaagcca
gcgctgaatttgggctgagattaggacttgcaggaggccggtccagaagacggcggaaggaatcttggcgggcgcacgcatgcgtgatagaccctcc
acacgtgtggccgggccgcggcctccccgtgctcggaggtcccgcccccggccgtagcatctttccggacgtggggagccggttgtaaagaagaaag
ttctaccctttctgtcaaa

Emi1 5'UTR_short
Aaagtaccagctggcgccttttaagagatacaggtctgtgaagcaggcaggttgctcagctgcccccggagcggttcctccacctgaggcagactcca
cgtcggctggc

scFv-GCN4-sfGFP-GB1 (referred to as scFv-GFP in the manuscript)
ATGGGCCCCGACATCGTGATGACCCAGAGCCCCAGCAGCCTGAGCGCCAGCGTGGGCGACCGCGTGACCATCAC
CTGCCGCAGCAGCACCGGCGCCGTGACCACCAGCAACTACGCCAGCTGGGTGCAGGAGAAGCCCGGCAAGCTGT
TCAAGGGCCTGATCGGCGGCACCAACAACCGCGCCCCCGGCGTGCCCAGCCGCTTCAGCGGCAGCCTGATCGGCG
ACAAGGCCACCCTGACCATCAGCAGCCTGCAGCCCGAGGACTTCGCCACCTACTTCTGCGCCCTGTGGTACAGCAA
CCACTGGGTGTTCGGCCAGGGCACCAAGGTGGAGCTGAAGCGCGGCGGCGGCGGCAGCGGCGGCGGCGGCAGC
GGCGGCGGCGGCAGCAGCGGCGGCGGCAGCGAGGTGAAGCTGCTGGAGAGCGGCGGCGGCCTGGTGCAGCCC
GGCGGCAGCCTGAAGCTGAGCTGCGCCGTGAGCGGCTTCAGCCTGACCGACTACGGCGTGAACTGGGTGCGCCA
GGCCCCCGGCCGCGGCCTGGAGTGGATCGGCGTGATCTGGGGCGACGGCATCACCGACTACAACAGCGCCCTGA
AGGACCGCTTCATCATCAGCAAGGACAACGGCAAGAACACCGTGTACCTGCAGATGAGCAAGGTGCGCAGCGAC
GACACCGCCCTGTACTACTGCGTGACCGGCCTGTTCGACTACTGGGGCCAGGGCACCCTGGTGACCGTGAGCAGC
TACCCATACGATGTTCCAGATTACGCTGGTGGAGGCGGAGGTTCTGGGGGAGGAGGTAGTGGCGGTGGTGGTTC
AGGAGGCGGCGGAAGCTTGGATCCAGGTGGAGGTGGAAGCGGTAGCAAAGGAGAAGAACTTTTCACTGGAGTT
GTCCCAATTCTTGTTGAATTAGATGGTGATGTTAATGGGCACAAATTTTCTGTCCGTGGAGAGGGTGAAGGTGATG
CTACAAACGGAAAACTCACCCTTAAATTTATTTGCACTACTGGAAAACTACCTGTTCCGTGGCCAACACTTGTCACT
ACTCTGACCTATGGTGTTCAATGCTTTTCCCGTTATCCGGATCACATGAAACGGCATGACTTTTTCAAGAGTGCCAT
GCCCGAAGGTTATGTACAGGAACGCACTATATCTTTCAAAGATGACGGGACCTACAAGACGCGTGCTGAAGTCAA
GTTTGAAGGTGATACCCTTGTTAATCGTATCGAGTTAAAGGGTATTGATTTTAAAGAAGATGGAAACATTCTTGGA
CACAAACTCGAGTACAACTTTAACTCACACAATGTATACATCACGGCAGACAAACAAAAGAATGGAATCAAAGCTA
ACTTCAAAATTCGCCACAACGTTGAAGATGGTTCCGTTCAACTAGCAGACCATTATCAACAAAATACTCCAATTGGC
GATGGCCCTGTCCTTTTACCAGACAACCATTACCTGTCGACACAATCTGTCCTTTCGAAAGATCCCAACGAAAAGCG
TGACCACATGGTCCTTCTTGAGTTTGTAACTGCTGCTGGGATTACACATGGCATGGATGAGCTCTACAAAGGTGGA
GGTCGGACCGAAGAGTACAAGCTTATCCTGAACGGTAAAACCCTGAAAGGTGAAACCACCACCGAAGCTGTTGAC
GCTGCTACCGCGGAAAAAGTTTTCAAACAGTACGCTAACGACAACGGTGTTGACGGTGAATGGACCTACGACGAC
GCTACCAAAACCTTCACGGTAACCGAATAA

PP7-2xmCherry-CAAX
ATGTCCAAAACCATCGTTCTTTCGGTCGGCGAGGCTACTCGCACTCTGACTGAGATCCAGTCCACCGCAGACCGTC
AGATCTTCGAAGAGAAGGTCGGGCCTCTGGTGGGTCGGCTGCGCCTCACGGCTTCGCTCCGTCAAAACGGAGCCA
AGACCGCGTATCGCGTCAACCTAAAACTGGATCAGGCGGACGTCGTTGATTGCTCCACCAGCGTCTGCGGCGAGC
TTCCGAAAGTGCGCTACACTCAGGTATGGTCGCACGACGTGACAATCGTTGCGAATAGCACCGAGGCCTCGCGCA
AATCGTTGTACGATTTGACCAAGTCCCTCGTCGCGACCTCGCAGGTCGAAGATCTTGTCGTCAACCTTGTGCCGCT
GGGCCGTCGTGCGGACCCGCTAGCCTCCTgcggccgcGGTGGTGGTACGCGTGATTCGACTAGTGGAGGAAGCGGA
GGAGGAgtgagcaagggcgaggaggataacatggccatcatcaaggagttcatgcgcttcaaggtgcacatggagggctccgtgaacggccacg
agttcgagatcgagggcgagggcgagggccgcccctacgagggcacccagaccgccaagctgaaggtgaccaagggtggcccccctgcccttcgcct
gggacatcctgtcccctcagttcatgtacggctccaaggcctacgtgaagcaccccgccgacatccccgactacttgaagctgtccttccccgagggct
tcaagtgggagcgcgtgatgaacttcgaggacggcggcgtggtgaccgtgacccaggactcctccctgcaggacggcgagttcatctacaaggtgaa
gctgcgcggcaccaacttcccctccgacggccccgtaatgcagaagaagaccatgggctgggaggcctcctccgagcggatgtaccccgaggacggc
gccctgaagggcgagatcaagcagaggctgaagctgaaggacggcggccactacgacgctgaggtcaagaccacctacaaggccaagaagcccgt
gcagctgcccggcgcctacaacgtcaacatcaagttggacatcacctcccacaacgaggactacaccatcgtggaacagtacgaacgcgccgaggg
ccgccactccaccggcggcatggacgagctgtacaagCATGGCATGGATGAGCTCTACAAAGGTGGAGGTCGGACCGAAGAG

TACAAGCTTATCCTGAACGGTAAAACCCTGAAAGGTGAAACCACCACCGAAGCTGTTGACGCTGCTACCGCGGAA
AAAGTTTTCAAACAGTACGCTAACGACAACGGTGTTGACGGTGAATGGACCTACGACGACGCTACCAAAACCTTC
ACGGTAACCGAAGCTAGTGGTGGTGGTGGTAGCGGTGGTGGTAGCagcttaccggtcgccGAATTCGGTgtgagcaaggg
cgaggaggataacatggccatcatcaaggagttcatgcgcttcaaggtgcacatggagggctccgtgaacggccacgagttcgagatcgagggcga
gggcgagggccgcccctacgagggcacccagaccgccaagctgaaggtgaccaagggtggccccctgcccttcgcctgggacatcctgtcccctcag
ttcatgtacggctccaaggcctacgtgaagcaccccgccgacatccccgactacttgaagctgtccttccccgagggcttcaagtgggagcgcgtgatg
aacttcgaggacggcggcgtggtgaccgtgacccaggactcctccctgcaggacggcgagttcatctacaaggtgaagctgcgcggcaccaacttcc
cctccgacggccccgtaatgcagaagaagaccatgggctgggaggcctcctccgagcggatgtaccccgaggacggcgccctgaagggcgagatca
agcagaggctgaagctgaaggacggcggccactacgacgctgaggtcaagaccacctacaaggccaagaagcccgtgcagctgcccggcgcctac
aacgtcaacatcaagttggacatcacctcccacaacgaggactacaccatcgtggaacagtacgaacgcgccgagggccgccactccaccggcggc
atggacgagctgtacaagCATATGGGTGGAGGTTCTGGTGGTGGATCTGGTGGAGGTTCTGGAGGTGGAAAAATGTCC
AAGGATGGTAAGAAAAAGAAGAAGAAGTCAAAAACCAAGTGTGTTATCATGtaa

BFP-Kif18b-PP7$_{24x}$
gacggatcgggagatctcccgatcccctatggtgcactctcagtacaatctgctctgatgccgcatagttaagccagtatctgctccctgcttgtgtgttg
gaggtcgctgagtagtgcgcgagcaaaatttaagctacaacaaggcaaggcttgaccgacaattgcatgaagaatctgcttagggttaggcgttttgc
gctgcttcgcgatgtacgggccagatatacgcgttgacattgattattgactagttattaatagtaatcaattacggggtcattagttcatagcccatata
tggagttccgcgttacataacttacggtaaatggcccgcctggctgaccgcccaacgacccccgcccattgacgtcaataatgacgtatgttcccatag
taacgccaatagggactttccattgacgtcaatgggtggagtatttacggtaaactgcccacttggcagtacatcaagtgtatcatatgccaagtacgc
cccctattgacgtcaatgacggtaaatggcccgcctggcattatgcccagtacatgaccttatgggactttcctacttggcagtacatctacgtattagtc
atcgctattaccatggtgatgcggttttggcagtacatcaatgggcgtggatagcggtttgactcacggggatttccaagtctccaccccattgacgtca
ATGGGAGTTTGTTTTGGCACCAAAATCAACGGGACTTTCCAAAATGTCGTAACAACTCCGCCCCATTGAcgcaaatgg
gcggtaggcgtgtacggtgggaggtctatataagcagagctctccctatcagtgatagagatctccctatcagtgatagagatcgtcgacgagctcgtt
tagtgaaccgtcagatcgcctggagacgccatccacgctgttttgacctccatagaagacaccgggaccgatccagcctccggactctagcgtttaaa
cttAAGCTTGCCACCATGGGCGCCATCATACACGCGGCCGCAATCATcggtccgagcgagctgattaaggagaacatgcacatga
agctAtacatggagggcaccgtggacaaccatcacttcaagtgcacatccgagggcgaaggcaagccctacgagggcacccagaccatgagaatc
aaggtggtcgagggcggccctctccccttcgccttcgacatcctggctactagcttcctctacggcagcaagaccttcatcaaccacacccagggcatc
cccgacttcttcaagcagtccttccctgagggcttcacatgggagagagtcaccacatacgaagacgggggcgtgctgaccgctacccaggacacca
gcctccaggacggctgcctcatctacaacgtcaagatcagaggggtgaacttcacatccaacggccctgtgatgcagaagaaaacactcggctggga
ggccttcaccgagacgctgtaccccgctgacggcggcctggaaggcagaaacgacatggccctgaagctcgtgggcgggagccatctgatcgcaaa
catcaagaccacatatagatccaagaaacccgctaagaacctcaagatgcctggcgtctactatgtggactacagactggaaagaatcaaggaggc
caacaacgagacctacgtcgagcagcacgaggtggcagtggccagatactgcgacctccctagcaaactggggcacaaacttaatcACCGGTCA
GCAGTGGAGGACAGCACGCTGCAAGTAGTGGTACGGGTGCGGCCCCCCACCCCTCGGGAGCTGGACAGTCAGCG
GCGGCCAGTGGTTCAGGTGGTGGACGAGCGGGTGCTGGTGTTTAACCCTGAGGAGCCCGATGGAGGGTTCCCTG
GCCTGAAATGGGGTGGCACCCATGATGGCCCCAAGAAGAAGGGCAAAGACCTGACGTTTGTCTTTGACCGGGTCT
TTGGCGAGGCGGCCACCCAACAGGACGTGTTCCAGCACACCACGCACAGCGTCCTGGACAGCTTCCTCCAGGGCT
ACAACTGCTCAGTGTTTGCCTACGGGGCCACCGGGGCTGGGAAGACACACACCATGCTGGGAAGGGAGGGGGAC
CCCGGCATCATGTACCTGACCACCGTGGAACTGTACAGGCGCCTGGAGGCCCGCCAGCAGGAGAAGCACTTCGA
GGTGCTCATCAGCTACCAGGAGGTGTATAATGAACAGATCCATGACCTCCTGGAGCCCAAGGGGCCCCTTGCCAT
CCGCGAGGACCCCGACAAGGGGGTGGTGGTGCAAGGACTTTCTTTCCACCAGCCAGCCTCAGCCGAGCAGCTGCT
GGAGATACTGACCAGGGGGAACCGTAACCGCACGCAGCACCCCACTGATGCCAACGCGACTTCCTCCCGCTCCCA
TGCCATCTTCCAGATCTTTGTGAAGCAGCAGGACCGGGTTCCAGGACTGACCCAGGCTGTCCAGGTGGCCAAGAT
GAGCCTGATTGACCTGGCTGGCTCAGAGCGGGCATCCAGCACCCATGCGAAGGGGGAGCGGCTGCGGGAGGGG
GCCAACATCAACCGCTCTCTGCTGGCGCTCATCAACGTCCTCAATGCCTTGGCCGATGCAAAGGGCCGCAAGACCG
CTGTGCCCTACGCGGACAGCGCACTGACCCGCCTGCTCAAAGACTCCCTCGGGGGCAACTGCCGCACAGTGATGA
TCGCTGCCATCAGCCCCTCCAGCCTGACCTACGAGGACACGTATAATACCCTCAAATATGCCGACCGGGCCAAGGA
GATCAGGCTCTCGCTGAAGAGCAATGTGACCAGCCTGGACTGTCACATCAGCCAGTATGCTACCATCTGCCAACAG

CTCCAGGCTGAGGTAGCCGCTCTGAGGAAGAAGCTCCAAGTGTATGAGGGGGGAGGCCAGCCCCCACCACAGGA
CCTCCCAGGATCTCCCAAGTCGGGACCACCACCAGAACACCTTCCCAGCTCCCCCTTGCCACCCCACCCTCCCAGCC
AGCCCTGCACCCCAGAGCTCCCTGCAGGGCCTAGAGCCCTTCAAGAGGAGAGTCTGGGGATGGAGGCCCAGGTG
GAGAGGGCCATGGAAGGGAACTCTTCAGACCAGGAGCAGTCCCCAGAGGATGAGGATGAAGGCCCAGCTGAGG
AGGTTCCAACCCAGATGCCAGAGCAGAACCCCACACATGCACTGCCAGAGTCCCCTCGCCTGACCCTGCAGCCCAA
GCCAGTCGTGGGCCACTTCTCAGCACGGGAACTGGATGGGGACCGTTCTAAGCGGTTGGCCCTAAAGGTGCTGTG
CGTTGCCCAGCGGCAGTACTCCCTGCTCCAAGCAGCCAACCTCCTGACGCCCGACATGATCACAGAGTTTGAGACC
CTACAGCAGCTGGTGCAAGAGGAAAAAATTGAGCCTGGGGCAGAGGCCTTGAGGACTTCAGGCCTGGCCAGGG
GGGCACCTCTGGCTCAGGAGCTGTGTTCAGAGTCAAAGCCTCCAGGATACACTGGCCCTGTGACCCGGACTATGG
CGAGGCGACTGAGTGGCCCCCTGCACACCCTGGGAATCCCGCCTGGACCCAACTGCACCCCAGCCCAGGGGTCCC
GATGGCCCATGGAGAAGAAGAGGAGGAGACCAAGCGCCTTGGAGGCAGACAGTCCCATGGCCCCAAAGCGGGG
CACCAAGCGCCAGCGCCAGTCCTTCCTGCCCTGCCTAAGGAGAGGGTCTCTGCCTGACACCCAACCTTCACAGGGG
CCCAGCACCCCCAAAGGAGAAAGGGCCTCCTCCCCCTGCCATTCCCCTCGCGTTTGCCCAGCCACAGTCATCAAAA
GCCGGGTGCCCCTGGGCCCTTCCGCCATGCAGAACTGCTCCACCCCGCTGGCTCTGCCCACTCGAGACCTCAATGC
CACCTTTGATCTCTCTGAGGAGCCTCCCTCAAAGCCCAGTTTCCATGAATGCATTGGCTGGGACAAAATACCCCAG
GAGCTGAGCAGGCTGGACCAGCCCTTCATCCCCAGGGCACCTGTGCCCCTGTTCACCATGAAGGGCCCCAAGCCA
ACATCTTCCCTCCCTGGGACCTCTGCCTGCAAGAAGAAGCGCGTTGCGAGTTCCTCAGTCTCCCATGGCCGCAGCC
GCATCGCCCGCCTCCCCAGCAGCACTTTGAAGAGGCCAGCTGGGCCCCTTGTACTCCCAGAGCTGCCCTTGAGTCC
CCTGTGCCCTAGCAACCGGAGGAATGGAAAGGACCTCATCAGGGTGGGGAGAGCACTCTCAGCAGGGAACGGCG
TCACCAAGGTGTCCGATATCTAACGACACGATTCGAAGATCGCATCGCTAGCCCGGTTGTACAGGTACCGGATCCT
AAGGTACCTAATTGCCTAGAAAGGAGCAGACGATATGGCGTCGCTCCCTGCAGGTCGACTCTAGAAACCAGCAGA
GCATATGGGCTCGCTGGCTGCAGTATTCCCGGGTTCATTAGATCCTAAGGTACCTAATTGCCTAGAAAGGAGCAG
ACGATATGGCGTCGCTCCCTGCAGGTCGACTCTAGAAACCAGCAGAGCATATGGGCTCGCTGGCTGCAGTATTCC
CGGGTTCATTAGATCCTAAGGTACCTAATTGCCTAGAAAGGAGCAGACGATATGGCGTCGCTCCCTGCAGGTCGA
CTCTAGAAACCAGCAGAGCATATGGGCTCGCTGGCTGCAGTATTCCCGGGTTCATTAGATCCTAAGGTACCTAATT
GCCTAGAAAGGAGCAGACGATATGGCGTCGCTCCCTGCAGGTCGACTCTAGAAACCAGCAGAGCATATGGGCTC
GCTGGCTGCAGTATTCCCGGGTTCATTAGATCCTAAGGTACCTAATTGCCTAGAAAGGAGCAGACGATATGGCGT
CGCTCCCTGCAGGTCGACTCTAGAAACCAGCAGAGCATATGGGCTCGCTGGCTGCAGTATTCCCGGGTTCATTAGA
TCCTAAGGTACCTAATTGCCTAGAAAGGAGCAGACGATATGGCGTCGCTCCCTGCAGGTCGACTCTAGAAACCAG
CAGAGCATATGGGCTCGCTGGCTGCAGTATTCCCGGGTTCATTAGATCCTAAGGTACCTAATTGCCTAGAAAGGA
GCAGACGATATGGCGTCGCTCCCTGCAGGTCGACTCTAGAAACCAGCAGAGCATATGGGCTCGCTGGCTGCAGTA
TTCCCGGGTTCATTAGATCCTAAGGTACCTAATTGCCTAGAAAGGAGCAGACGATATGGCGTCGCTCCCTGCAGGT
CGACTCTAGAAACCAGCAGAGCATATGGGCTCGCTGGCTGCAGTATTCCCGGGTTCATTAGATCCTAAGGTACCTA
ATTGCCTAGAAAGGAGCAGACGATATGGCGTCGCTCCCTGCAGGTCGACTCTAGAAACCAGCAGAGCATATGGGC
TCGCTGGCTGCAGTATTCCCGGGTTCATTAGATCCTAAGGTACCTAATTGCCTAGAAAGGAGCAGACGATATGGC
GTCGCTCCCTGCAGGTCGACTCTAGAAACCAGCAGAGCATATGGGCTCGCTGGCTGCAGTATTCCCGGGTTCATTA
GATCCTAAGGTACCTAATTGCCTAGAAAGGAGCAGACGATATGGCGTCGCTCCCTGCAGGTCGACTCTAGAAACC
AGCAGAGCATATGGGCTCGCTGGCTGCAGTATTCCCGGGTTCATTAGATCCTAAGGTACCTAATTGCCTAGAAAG
GAGCAGACGATATGGCGTCGCTCCCTGCAGGTCGACTCTAGAAACCAGCAGAGCATATGGGCTCGCTGGCTGCAG
TATTCCCGGGTTCATTAGATCTcgcgAAGGGCGAATTCacgaGGCGCGCCcggatcGCGATCGCaatcTTAATTAACTCG
AGtctagagggcccgtttaaacccgctgatcagcctcgactgtgccttctagttgccagccatctgttgtttgcccctcccccgtgccttccttgaccctg
gaaggtgccactcccactgtcctttcctaataaaatgaggaaattgcatcgcattgtctgagtaggtgtcattctattctggggggtggggtggggcagg
acagcaagggggaggattgggaagacaatagcaggcatgctggggatgcggtgggctctatggcttctgaggcggaaagaaccagctggggctcta
gggggtatccccacgcgccctgtagcggcgcattaagcgcggcgggtgtggtggttacgcgcagcgtgaccgctacacttgccagcgccctagcgccc
gctcctttcgctttcttcccttcctttctcgccacgttcgccggctttccccgtcaagctctaaatcgggggctccctttagggttccgatttagtgctttacg
gcacctcgaccccaaaaaacttgattagggtgatggttcacgtagtgggccatcgccctgatagacggtttttcgccctttgacgttggagtccacgttc
tttaatagtggactcttgttccaaactggaacaacactcaaccctatctcggtctattcttttgatttataagggattttgccgatttcggcctattggttaa
aaaatgagctgatttaacaaaaatttaacgcgaattaattctgtggAATGTGTGTCAGTTAGGGTGTGGAAAGTCCCCAGGCTCCC

CAGCAGGCAGAAGTATGCAAAGCATGCATCTCAATTAGTCAGCAACCAGGTGTGGAAAGTCCCCAGGCTCCCCAG
CAGGCAGAAGTATGCAAAGCATGCATCTCAATTAGTCAGCAACCATAGTCCCGCCCCTAACTCCGCCCATCCCGCC
CCTAACTCCGCCCAGTTCCGCCCATTCTCCGCCCCATGGCTGACTAATTTTTTTTATTTATGCAGAGGCCGAGGCCG
CCTCTGCCTCTGAGCTATTCCAGAAGTAGTGAGGAGGCTTTTTTGGAGGCCTAGGCTTTTGCAAAAAGCTCCcggga
gcttgtatatccattttcggatctgatcagcacgtgttgacaattaatcatcggcatagtatatcggcatagtataatacgacaaggtgaggaactaaac
catggccaagttgaccagtgccgttccggtgctcaccgcgcgcgacgtcgccggagcggtcgagttctggaccgaccggctcgggttctcccgggactt
cgtggaggacgacttcgccggtgtggtccgggacgacgtgaccctgttcatcagcgcggtccaggaccaggtggtgccggacaacaccctggcctgg
gtgtgggtgcgcggcctggacgagctgtacgccgagtggtcggaggtcgtgtccacgaacttccgggacgcctccgggccggccatgaccgagatcg
gcgagcagccgtgggggcgggagttcgccctgcgcgacccggccggcaactgcgtgcacttcgtggccgaggagcaggactgacacgtgctacgag
atttcgattccaccgccgccttctatgaaaggttgggcttcggaatcgttttccgggacgccggctggatgatcctccagcgcggggatctcatgctgga
gttcttcgcccaccccaacttgtttattgcagcttataatggttacaaataaagcaatagcatcacaaatttcacaaataaagcatttttttcactgcattc
tagttgtggtttgtccaaactcatcaatgtatcttatcatgtctgtataccgtcgacctctagctagagcttggcgtaatcatggtcatagctgtttcctgtg
tgaaattgttatccgctcacaattccacacaacatacgagccggaagcataaagtgtaaagcctggggtgcctaatgagtgagctaactcacattaat
tgcgttgcgctcactgcccgctttccagtcgggaaacctgtcgtgccagctgcattaatgaatcggccaacgcgcggggagaggcggtttgcgtattgg
gcgctcttccgcttcctcgctcactgactcgctgcgctcggtcgttcggctgcggcgagcggtatcagctcactcaaaggcggtaatacggttatccaca
gaatcaggggataacgcaggaaagaacatgtgagcaaaaggccagcaaaaggccaggaaccgtaaaaaggccgcgttgctggcgtttttccatag
gctccgcccccctgacgagcatcacaaaaatcgacgctcaagtcagaggtggcgaaacccgacaggactataaagataccaggcgtttccccctgga
agctccctcgtgcgctctcctgttccgaccctgccgcttaccggatacctgtccgcctttctcccttcgggaagcgtggcgctttctcatagctcacgctgt
aggtatctcagttcggtgtaggtcgttcgctccaagctgggctgtgtgcacgaaccccccgttcagcccgaccgctgcgccttatccggtaactatcgtc
ttgagtccaacccggtaagacacgacttatcgccactggcagcagccactggtaacaggattagcagagcgaggtatgtaggcggtgctacagagtt
cttgaagtggtggcctaactacggctacactagaagaacagtatttggtatctgcgctctgctgaagccagttaccttcggaaaaagagttggtagctc
ttgatccggcaaacaaaccaccgctggtagcggtttttttgtttgcaagcagcagattacgcgcagaaaaaaaggatctcaagaagatcctttgatctt
ttctacggggtctgacgctcagtggaacgaaaactcacgttaagggattttggtcatgagattatcaaaaaggatcttcacctagatccttttaaattaa
aaatgaagttttaaatcaatctaaagtatatatgagtaaacttggtctgacagttaccaatgcttaatcagtgaggcacctatctcagcgatctgtctatt
tcgttcatccatagttgcctgactccccgtcgtgtagataactacgatacgggagggcttaccatctggccccagtgctgcaatgataccgcgagaccc
acgctcaccggctccagatttatcagcaataaaccagccagccggaagggccgagcgcagaagtggtcctgcaactttatccgcctccatccagtcta
ttaattgttgccgggaagctagagtaagtagttcgccagttaatagtttgcgcaacgttgttgccattgctacaggcatcgtggtgtcacgctcgtcgttt
ggtatggcttcattcagctccggttcccaacgatcaaggcgagttacatgatcccccatgttgtgcaaaaaagcggttagctccttcggtcctccgatcg
ttgtcagaagtaagttggccgcagtgttatcactcatggttatggcagcactgcataattctcttactgtcatgccatccgtaagatgcttttctgtgactg
gtgagtactcaaccaagtcattctgagaatagtgtatgcggcgaccgagttgctcttgcccggcgtcaatacgggataataccgcgccacatagcaga
actttaaaagtgctcatcattggaaaacgttcttcggggcgaaaactctcaaggatcttaccgctgttgagatccagttcgatgtaacccactcgtgcac
ccaactgatcttcagcatcttttactttcaccagcgtttctgggtgagcaaaaacaggaaggcaaaatgccgcaaaaaagggaataagggcgacacg
gaaatgttgaatactcatactcttcctttttcaatattattgaagcatttatcagggttattgtctcatgagcggatacatatttgaatgtatttagaaaaa
taaacaaataggggttccgcgcacatttccccgaaaagtgccacctgacgtc

tdPP7-3xmCherry
tgtcacctaaatcgtatgtgtatgatacataaggttatgtattaattgtagccgcgttctaacgacaatatgtacaagcctaattgtgtagcatctggctt
actgaagcagaccctatcatctctctcgtaaactgccgtcagagtcggtttggttggacgaaccttctgagtttctggtaacgccgtcccgcacccggaa
atggtcagcgaaccaatcagcagggtcatcgctagccagatcctctacgccggacgcatcgtggccggcatcaccggcgccacaggtgcggttgctg
gcgcctatatcgccgacatcaccgatggggaagatcgggctcgccacttcgggctcatgagcgcttgtttcggcgtgggtatggtggcaggccccgtgg
ccgggggactgttgggcgccatctccttgcatgcaccattccttgcggcggcggtgctcaacggcctcaacctactactgggctgcttcctaatgcagga
gtcgcataagggagagcgtcgaatggtgcactctcagtacaatctagctctgatgccgcatagttaagccagccccgacacccgccaacacccgctga
cgcgccctgacgggcttgtctgctcccggcatccgcttacagacaagctgtgaccgtctccgggagctgcatgtgtcagaggttttcaccgtcatcaccg
aaacgcgcgagacgaaagggcctcgtgatacgcctatttttataggttaatgtcatgataataatggtttcttagacgtcaggtggcacttttcggggaa
atgtgcgcggaacccctatttgtttatttttctaaatacattcaaatatgtatccgctcatgagacaataaccctgataaatgcttcaatatattgaaaa
aggaagagtatgagtattcaacatttccgtgtcgcccttattcccttttttgcggcattttgccttcctgtttttgctcacccagaaacgctggtgaaagta
aaagatgctgaagatcagttgggtgcacgagtgggttacatcgaactggatctcaacagcggtaagatccttgagagttttcgccccgaagaacgtttt

ccaatgatgagcacttttaaagttctgctatgtggcgcggtattatcccgtattgacgccgggcaagagcaactcggtcgccgcatacactattctcaga
atgacttggttgagtactcaccagtcacagaaaagcatcttacggatggcatgacagtaagagaattatgcagtgctgccataaccatgagtgataac
actgcggccaacttacttctgacaacgatcggaggaccgaaggagctaaccgcttttttgcacaacatgggggatcatgtaactcgccttgatcgttgg
gaaccggagctgaatgaagccataccaaacgacgagcgtgacaccacgatgcctgtagcaatggcaacaacgttgcgcaaactattaactggcgaa
ctacttactctagcttcccggcaacaattaatagactggatggaggcggataaagttgcaggaccacttctgcgctcggcccttccggctggctggttta
ttgctgataaatctggagccggtgagcgtgggtctcgcggtatcattgcagcactggggccagatggtaagccctcccgtatcgtagttatctacacga
cggggagtcaggcaactatggatgaacgaaatagacagatcgctgagataggtgcctcactgattaagcattggtaactgtcagaccaagtttactca
tatatactttagattgatttaaaacttcatttttaatttaaaaggatctaggtgaagatcctttttgataatctcatgaccaaaatcccttaacgtgagtttt
cgttccactgagcgtcagaccccgtagaaaagatcaaaggatcttcttgagatcctttttttctgcgcgtaatctgctgcttgcaaacaaaaaaaccacc
gctaccagcggtggtttgtttgccggatcaagagctaccaactctttttccgaaggtaactggcttcagcagagcgcagataccaaatactgtccttcta
gtgtagccgtagttaggccaccacttcaagaactctgtagcaccgcctacatacctcgctctgctaatcctgttaccagtggctgctgccagtggcgata
agtcgtgtcttaccgggttggactcaagacgatagttaccggataaggcgcagcggtcgggctgaacggggggttcgtgcacacagcccagcttgga
gcgaacgacctacaccgaactgagatacctacagcgtgagctatgagaaagcgccacgcttcccgaagggagaaaggcggacaggtatccggtaa
gcggcagggtcggaacaggagagcgcacgagggagcttccagggggaaacgcctggtatctttatagtcctgtcgggtttcgccacctctgacttgag
cgtcgatttttgtgatgctcgtcaggggggcggagcctatggaaaaacgccagcaacgcggcctttttacggttcctggccttttgctggccttttgctca
catgttctttcctgcgttatcccctgattctgtggataaccgtattaccgcctttgagtgagctgataccgctcgccgcagccgaacgaccgagcgcagc
gagtcagtgagcgaggaagcggaagagcgcccaatacgcaaaccgcctctccccgcgcgttggccgattcattaatgcagctgtggaatgtgtgtca
gttagggtgtggaaagtccccaggctccccagcaggcagaagtatgcaaagcatgcatctcaattagtcagcaaccaggtgtggaaagtccccaggc
tccccagcaggcagaagtatgcaaagcatgcatctcaattagtcagcaaccatagtcccgcccctaactccgcccatcccgcccctaactccgcccagt
tccgcccattctccgccccatggctgactaattttttttatttatgcagaggccgaggccgcctcggcctctgagctattccagaagtagtgaggaggctt
ttttggaggcctaggcttttgcaaaaagcttggacacaagacaggcttgcgagatatgtttgagaataccactttatcccgcgtcagggagaggcagtg
cgtaaaaagacgcggactcatgtgaaatactggtttttagtgcgccagatctctataatctcgcgcaacctattttcccctcgaacactttttaagccgta
gataaacaggctgggacacttcacatgagcgaaaaatacatcgtcacctgggacatgttgcagatccatgcacgtaaactcgcaagccgactgatgc
cttctgaacaatggaaaggcattattgccgtaagccgtggcggtctgtaccgggtgcgttactggcgcgtgaactgggtattcgtcatgtcgataccgtt
tgtatttccagctacgatcacgacaaccagcgcgagcttaaagtgctgaaacgcgcagaaggcgatggcgaaggcttcatcgttattgatgacctggt
ggataccggtggtactgcggttgcgattcgtgaaatgtatccaaaagcgcactttgtcaccatcttcgcaaaaccggctggtcgtccgctggttgatgac
tatgttgttgatatcccgcaagatacctggattgaacagccgtgggatatgggcgtcgtattcgtcccgccaatctccggtcgctaatcttttcaacgcct
ggcactgccgggcgttgttctttttaacttcaggcgggttacaatagtttccagtaagtattctggaggctgcatccatgacacaggcaaacctgagcga
aaccctgttcaaaccccgctttaaacatcctgaaacctcgacgctagtccgccgctttaatcacggcgcacaaccgcctgtgcagtcggcccttgatggt
aaaaccatccctcactggtatcgcatgattaaccgtctgatgtggatctggcgcggcattgacccacgcgaaatcctcgacgtccaggcacgtattgtg
atgagcgatgccgaacgtaccgacgatgatttatacgatacggtgattggctaccgtggcggcaactggatttatgagtgggccccggatctttgtgaa
ggaaccttacttctgtggtgtgacataattggacaaactacctacagagatttaaagctctaaggtaaatataaaatttttaagtgtataatgtgttaaa
ctactgattctaattgtttgtgtattttagattccaacctatggaactgatgaatgggagcagtggtggaatgcctttaatgaggaaaacctgttttgctc
agaagaaatgccatctagtgatgatgaggctactgctgactctcaacattctactcctccaaaaaagaagagaaaggtagaagaccccaaggacttt
ccttcagaattgctaagttttttgagtcatgctgtgtttagtaatagaactcttgcttgctttgctatttacaccacaaaggaaaaagctgcactgctatac
aagaaaattatggaaaaatattctgtaacctttataagtaggcataacagttataatcataacatactgttttttcttactccacacaggcatagagtgt
ctgctattaataactatgctcaaaaattgtgtacctttagctttttaatttgtaaaggggttaataaggaatatttgatgtatagtgccttgactagagatc
ataatcagccataccacatttgtagaggttttacttgctttaaaaaacctcccacacctccccctgaacctgaaacataaaatgaatgcaattgttgttg
ttaacttgtttattgcagcttataatggttacaaataaagcaatagcatcacaaatttcacaaataaagcatttttttcactgcattctagttgtggtttgt
ccaaactcatcaatgtatcttatcatgtctggatcaactggataactcaagctaaccaaaatcatcccaaacttcccaccccataccctattaccactgc
caattacctagtggtttcatttactctaaacctgtgattcctctgaattattttcattttaaagaaattgtatttgttaaatatgtactacaaacttagtagtt
ggaagggctaattcactcccaaagaagacaagatatccttgatctgtggatctaccacacacaaggctacttccctgattagcagaactacacaccag
ggccaggggtcagatatccactgacctttggatggtgctacaagctagtaccagttgagccagataaggtagaagaggccaataaaggagagaaca
ccagcttgttacaccctgtgagcctgcatgggatggatgacccggagagagaagtgttagagtggaggtttgacagccgcctagcatttcatcacgtg
gcccgagagctgcatccggagtacttcaagaactgctgatatcgagcttgctacaagggactttccgctggggactttccagggaggcgtggcctggg
cgggactggggagtggcgagccctcagatcctgcatataagcagctgctttttgcctgtactgggtctctctggttagaccagatctgagcctgggagct
ctctggctaactagggaacccactgcttaagcctcaataaagcttgccttgagtgcttcaagtagtgtgtgcccgtctgttgtgtgactctggtaactaga
gatccctcagacccttttagtcagtgtggaaaatctctagcagtggcgcccgaacagggacttgaaagcgaaagggaaaccagaggagctctctcga

cgcaggactcggcttgctgaagcgcgcacggcaagaggcgaggggcggcgactggtgagtacgccaaaaattttgactagcggaggctagaagga
gagagatgggtgcgagagcgtcagtattaagcgggggagaattagatcgcgatgggaaaaaattcggttaaggccagggggaaagaaaaaatata
aattaaaacatatagtatgggcaagcagggagctagaacgattcgcagttaatcctggcctgttagaaacatcagaaggctgtagacaaatactggg
acagctacaaccatcccttcagacaggatcagaagaacttagatcattatataatacagtagcaaccctctattgtgtgcatcaaaggatagagataa
aagacaccaaggaagctttagacaagatagaggaagagcaaaacaaaagtaagaccaccgcacagcaagcggccggtgatcttcagacctggac
gatatatatgagggacaattggagaagtgaattatataaatataaagtagtaaaaattgaaccattaggagtagcacccaccaaggcaaagagaag
agtggtgcagagagaaaaaagagcagtgggaataggagctttgttccttgggttcttgggagcagcaggaagcactatgggcgcagcgtcaatgac
gctgacggtacaggccagacaattattgtctggtatagtgcagcagcagaacaatttgctgagggctattgaggcgcaacagcatctgttgcaactca
cagtctggggcatcaagcagctccaggcaagaatcctggctgtggaaagatacctaaaggatcaacagctcctggggatttggggttgctctggaaa
actcatttgcaccactgctgtgccttggaatgctagttggagtaataaatctctggaacagatttggaatcacacgacctggatggagtgggacagaga
aattaacaattacacaagcttaatacactccttaattgaagaatcgcaaaaccagcaagaaaagaatgaacaagaattattggaattagataaatgg
gcaagtttgtggaattggtttaacataacaaattggctgtggtatataaaattattcataatgatagtaggaggcttggtaggtttaagaatagtttttgc
tgtactttctatagtgaatagagttaggcagggatattcaccattatcgtttcagacccacctcccaaccccgaggggacccgacaggcccgaaggaat
agaagaagaaggtggagagagagacagagacagatccattcgattagtgaacggatctcgacggtATcgccaaatggcagtattcatccacaattt
taaaagaaaaggggggattggggggtacagtgcaggggaaagaatagtagacataatagcaacagacatacaaactaaagaattacaaaaacaa
attacaaaaattcaaaattttcgggtttattacagggacagcagagatccagtttggatcgataagcttgatatcgaattcctgcagcccgataaaat
aaaagattttatttagtctccagaaaaaggggggaatgaaagaccccacctgtaggtttggcaagctagctgcagtaacgccattttgcaaggcatgg
aaaaataccaaaccaagaatagagaagttcagatcaagggcgggtacatgaaaatagctaacgttgggccaaacaggatatctgcggtgagcagtt
tcggccccggcccggggccaagaacagatggtcaccgcagtttcggccccggcccgaggccaagaacagatggtccccagatatggcccaaccctc
agcagtttcttaagacccatcagatgtttccaggctcccccaaggacctgaaatgaccctgcgccttatttgaattaaccaatcagcctgcttctcgcttc
tgttcgcgcgcttctgcttcccgagctctataaaagagctcacaacccctcactcggcgcgccagtcctccgacagactgagtcgcccgggggggatct
ggagctctcgagaattctcAcgcgccGGATCCGCCACCATGTCCAAAACCATCGTTCTTTCGGTCGGCGAGGCTACTCGCAC
TCTGACTGAGATCCAGTCCACCGCAGACCGTCAGATCTTCGAAGAGAAGGTCGGGCCTCTGGTGGGTCGGCTGCG
CCTCACGGCTTCGCTCCGTCAAAACGGAGCCAAGACCGCGTATCGCGTCAACCTAAAACTGGATCAGGCGGACGT
CGTTGATTGCTCCACCAGCGTCTGCGGCGAGCTTCCGAAAGTGCGCTACACTCAGGTATGGTCGCACGACGTGAC
AATCGTTGCGAATAGCACCGAGGCCTCGCGCAAATCGTTGTACGATTTGACCAAGTCCCTCGTCGCGACCTCGCAG
GTCGAAGATCTTGTCGTCAACCTTGTGCCGCTGGGCCGTCGTGCGGACCCGCTAGCCTCCTgcggccgcTCCAAAACC
ATCGTTCTTTCGGTCGGCGAGGCTACTCGCACTCTGACTGAGATCCAGTCCACCGCAGACCGTCAGATCTTCGAAG
AGAAGGTCGGGCCTCTGGTGGGTCGGCTGCGCCTCACGGCTTCGCTCCGTCAAAACGGAGCCAAGACCGCGTATC
GCGTCAACCTAAAACTGGATCAGGCGGACGTCGTTGATTGCTCCACCAGCGTCTGCGGCGAGCTTCCGAAAGTGC
GCTACACTCAGGTATGGTCGCACGACGTGACAATCGTTGCGAATAGCACCGAGGCCTCGCGCAAATCGTTGTACG
ATTTGACCAAGTCCCTCGTCGCGACCTCGCAGGTCGAAGATCTTGTCGTCAACCTTGTGCCGCTGGGCCGTCGTGC
GGACCCGCTAGCCTCCACGCGTGATTCGACTAGTGGAGGAAGCGGAGGAGGAgtgagcaagggcgaggaggataacatg
gccatcatcaaggagttcatgcgcttcaaggtgcacatggagggctccgtgaacggccacgagttcgagatcgagggcgagggcgagggccgcccct
acgagggcacccagaccgccaagctgaaggtgaccaagggtggccccctgcccttcgcctgggacatcctgtcccctcagttcatgtacggctccaag
gcctacgtgaagcaccccgccgacatccccgactacttgaagctgtccttccccgagggcttcaagtgggagcgcgtgatgaacttcgaggacggcgg
cgtggtgaccgtgacccaggactcctccctgcaggacggcgagttcatctacaaggtgaagctgcgcggcaccaacttcccctccgacggccccgtaa
tgcagaagaagaccatgggctgggaggcctcctccgagcggatgtaccccgaggacggcgccctgaagggcgagatcaagcagaggctgaagctg
aaggacggcggccactacgacgctgaggtcaagaccacctacaaggccaagaagcccgtgcagctgcccggcgcctacaacgtcaacatcaagttg
gacatcacctcccacaacgaggactacaccatcgtggaacagtacgaacgcgccgagggccgccactccaccggcggcatggacgagctgtacaag
CATGGCATGGATGAGCTCTACAAAGGTGGAGGTCGGACCGAAGAGTACAAGCTTATCCTGAACGGTAAAACCCTG
AAAGGTGAAACCACCACCGAAGCTGTTGACGCTGCTACCGCGGAAAAAGTTTTCAAACAGTACGCTAACGACAAC
GGTGTTGACGGTGAATGGACCTACGACGACGCTACCAAAACCTTCACGGTAACCGAAGCTAGTGGAGGAAGCGG
AGGAGGAgtgagcaagggcgaggaggataacatggccatcatcaaggagttcatgcgcttcaaggtgcacatggagggctccgtgaacggccac
gagttcgagatcgagggcgagggcgagggccgcccctacgagggcacccagaccgccaagctgaaggtgaccaagggtggccccctgcccttcgcc
tgggacatcctgtcccctcagttcatgtacggctccaaggcctacgtgaagcaccccgccgacatccccgactacttgaagctgtccttccccgagggc
ttcaagtgggagcgcgtgatgaacttcgaggacggcggcgtggtgaccgtgacccaggactcctccctgcaggacggcgagttcatctacaaggtga
agctgcgcggcaccaacttcccctccgacggccccgtaatgcagaagaagaccatgggctgggaggcctcctccgagcggatgtaccccgaggacg

gcgccctgaagggcgagatcaagcagaggctgaagctgaaggacggcggccactacgacgctgaggtcaagaccacctacaaggccaagaagccc
gtgcagctgcccggcgcctacaacgtcaacatcaagttggacatcacctcccacaacgaggactacaccatcgtggaacagtacgaacgcgccgagg
gccgccactccaccggcggcatggacgagctgtacaagCATGGCATGGATGAGCTCTACAAAGGTGGAGGTCGGACCGAAGA
GTACAAGCTTATCCTGAACGGTAAAACCCTGAAAGGTGAAACCACCACCGAAGCTGTTGACGCTGCTACCGCGGA
AAAAGTTTTCAAACAGTACGCTAACGACAACGGTGTTGACGGTGAATGGACCTACGACGACGCTACCAAAACCTT
CACGGTAACCGAAGCTAGTGGTGGTGGTGGTAGCGGTGGTGGTAGCagcttaccggtcgccGAATTCGGTgtgagcaagg
gcgaggaggataacatggccatcatcaaggagttcatgcgcttcaaggtgcacatggagggctccgtgaacggccacgagttcgagatcgagggcg
agggcgagggccgcccctacgagggcacccagaccgccaagctgaaggtgaccaagggtggcccctgcccttcgcctgggacatcctgtcccctca
gttcatgtacggctccaaggcctacgtgaagcaccccgccgacatccccgactacttgaagctgtccttccccgagggcttcaagtgggagcgcgtgat
gaacttcgaggacggcggcgtggtgaccgtgacccaggactcctccctgcaggacggcgagttcatctacaaggtgaagctgcgcggcaccaacttc
ccctccgacggccccgtaatgcagaagaagaccatgggctgggaggcctcctccgagcggatgtaccccgaggacggcgccctgaagggcgagatc
aagcagaggctgaagctgaaggacggcggccactacgacgctgaggtcaagaccacctacaaggccaagaagcccgtgcagctgcccggcgccta
caacgtcaacatcaagttggacatcacctcccacaacgaggactacaccatcgtggaacagtacgaacgcgccgagggccgccactccaccggcgg
catggacgagctgtacaagCATATGtaaGCGGCCgtacctttaagaccaatgacttacaaggcagctgtagatcttagccactttttaaaagaaa
aggggggactggaagggctaattcactcccaacgaagacaagatctgctttttgcttgtactgggtctctctggttagaccagatctgagcctgggagc
tctctggctaactagggaacccactgcttaagcctcaataaagcttgccttgagtgcttcaagtagtgtgtgcccgtctgttgtgtgactctggtaactag
agatccctcagacccttttagtcagtgtggaaaatctctagcagcatctagaattaattccgtgtattctatag

SunTag$_{24x}$-mCherry
ATGGGCGCCATCATACACGCGGCCGCAATCATcggtccgGGTGGATCTGGAGGTGGAGGTTCTGGAGGAGAAGAA
CTTTTGAGCAAGAATTATCATCTTGAGAACGAAGTGGCTCGTCTTAAGAAAGGTTCTGGCAGTGGAGAAGAACTG
CTTTCAAAGAATTACCACCTGGAAAATGAGGTAGCTAGACTGAAAAAGGGGAGCGGAAGTGGGGAGGAGTTGCT
GAGCAAAAATTATCATTTGGAGAACGAAGTAGCACGACTAAAGAAAGGGTCCGGATCGGGTGAGGAGTTACTCT
CGAAAAATTATCATCTCGAAAACGAAGTGGCTCGGCTAAAAAAGGGCAGTGGTTCTGGAGAAGAGCTATTATCTA
AAAACTACCACCTCGAAAATGAGGTGGCACGCTTAAAAAAGGGAAGTGGCAGTGGTGAAGAGCTACTATCCAAG
AATTATCATCTTGAGAACGAGGTAGCGCGTTTGAAGAAGGGTTCCGGCTCAGGAGAGGAACTGCTCTCGAAGAAC
TATCATCTTGAAAATGAGGTCGCTCGATTAAAAAAGGGATCGGGCAGTGGTGAGGAACTACTTTCAAAGAATTAC
CACCTCGAAAACGAAGTAGCTCGATTAAAGAAAGGTTCAGGGTCGGGTGAAGAATTACTGAGTAAAAATTATCAT
CTGGAAAATGAGGTAGCGAGACTAAAAAAGGGGAGTGGTTCTGGCGAAGAGTTGCTATCGAAAAATTATCATCTT
GAGAACGAAGTTGCTAGGCTCAAAAAGGGCTCAGGCTCAGGCGAGGAGTTGCTCTCGAAAAACTACCACTTGGA
AAATGAGGTCGCGAGGTTGAAAAAGGGGAGCGGGTCGGGCGAGGAGTTATTGAGCAAAAACTATCATTTAGAG
AACGAAGTCGCGCGCTTAAAGAAAGGCTCGGGCTCGGGCGAAGAACTCTTATCGAAGAACTACCACCTCGAAAAT
GAGGTCGCCAGGTTGAAAAAGGGCAGTGGCAGCGGGGAGGAACTCTTGAGCAAGAACTACCACTTGGAGAATG
AGGTCGCGAGATTGAAGAAAGGGTCGGGGAGCGGCGAGGAATTGCTCAGCAAGAATTATCATTTGGAGAACGA
AGTCGCCAGGCTCAAGAAAGGCTCGGGGTCGGGGGAGGAATTGTTGAGTAAAAACTACCACTTGGAAAATGAAG
TCGCCAGGCTCAAAAAAGGGAGTGGGAGCGGCGAAGAGTTATTGAGCAAAAATTACCACTTGGAGAACGAAGTG
GCAAGGCTCAAGAAAGGGAGCGGCAGCGGGGAGGAGCTCTTATCGAAGAACTACCACTTAGAGAATGAAGTCGC
CCGCTTGAAGAAAGGCTCGGGGAGCGGGGAAGAGCTCTTGAGCAAGAACTACCACTTGGAAAATGAGGTGGCGC
GCTTGAAGAAAGGGAGCGGGAGCGGGGAAGAGTTACTATCTAAGAATTATCATCTCGAGAACGAGGTGGCTCGA
CTAAAGAAGGGCTCCGGCAGTGGGGAGGAACTCCTGTCGAAGAACTATCATCTTGAAAATGAGGTTGCAAGACTT
AAAAAGGGGTCCGGATCAGGTGAGGAACTACTCAGTAAGAATTACCACCTGGAAAACGAAGTTGCACGTTTGAA
GAAAGGATCAGGATCAGGCGAAGAACTGCTCTCAAAAGATTATCATTTGGAAAATGAGGTTGCACGTTTAAAAAA
GGGAAGTGGCAGTGGTGAGGAACTTCTGTCGAAAAATTATCATCTCGAGAATGAAGTAGCCCGACTTAAAAAGG
GTTCTGGCTCGGGTCAGCGGCCGCACCGGTCAGTGGAGGAGAACCCTGGACCTCCCAAGAAGAAGCGCAAGGTG
GGAGGAGGCGGAACTAGTgtgagcaagggcgaggaggataacatggccatcatcaaggagttcatgcgcttcaaggtgcacatggagggc
tccgtgaacggccacgagttcgagatcgagggcgagggcgagggccgcccctacgagggcacccagaccgccaagctgaaggtgaccaagggtgg
cccctgcccttcgcctgggacatcctgtcccctcagttcatgtacggctccaaggcctacgtgaagcaccccgccgacatccccgactacttgaagctg

tccttccccgagggcttcaagtgggagcgcgtgatgaacttcgaggacggcggcgtggtgaccgtgacccaggactcctccctgcaggacggcgagtt
catctacaaggtgaagctgcgcggcaccaacttcccctccgacggccccgtaatgcagaagaagaccatgggctgggaggcctcctccgagcggatg
taccccgaggacggcgccctgaagggcgagatcaagcagaggctgaagctgaaggacggcggccactacgacgctgaggtcaagaccacctacaa
ggccaagaagcccgtgcagctgcccggcgcctacaacgtcaacatcaagttggacatcacctcccacaacgaggactacaccatcgtggaacagtac
gaacgcgccgagggccgccactccaccggcggcatggacgagctgtacaagTGA

sfGFP-NLS-PP7$_{24x}$
ATGAGCAAAGGAGAAGAACTTTTCACTGGAGTTGTCCCAATTCTTGTTGAATTAGATGGTGATGTTAATGGGCACA
AATTTTCTGTCCGTGGAGAGGGTGAAGGTGATGCTACAAACGGAAAACTCACCCTTAAATTTATTTGCACTACTGG
AAAACTACCTGTTCCGTGGCCAACACTTGTCACTACTCTGACCTATGGTGTTCAATGCTTTTCCCGTTATCCGGATCA
CATGAAACGGCATGACTTTTTCAAGAGTGCCATGCCCGAAGGTTATGTACAGGAACGCACTATATCTTTCAAAGAT
GACGGGACCTACAAGACGCGTGCTGAAGTCAAGTTTGAAGGTGATACCCTTGTTAATCGTATCGAGTTAAAGGGT
ATTGATTTTAAAGAAGATGGAAACATTCTTGGACACAAACTCGAGTACAACTTTAACTCACACAATGTATACATCAC
GGCAGACAAACAAAAGAATGGAATCAAAGCTAACTTCAAAATTCGCCACAACGTTGAAGATGGTTCCGTTCAACT
AGCAGACCATTATCAACAAAATACTCCAATTGGCGATGGCCCTGTCCTTTTACCAGACAACCATTACCTGTCGACAC
AATCTGTCCTTTCGAAAGATCCCAACGAAAAGCGTGACCACATGGTCCTTCTTGAGTTTGTAACTGCTGCTGGGATT
ACACATGGCATGGATGAGCTCTACAAACCCAAGAAGAAGCGCAAGGTGGATATCTAACGACACGATTCGAAGATC
GCATCGCTAGCCCGGTTGTACAGGTACCGGATCCTAAGGTACCTAATTGCCTAGAAAGGAGCAGACGATATGGCG
TCGCTCCCTGCAGGTCGACTCTAGAAACCAGCAGAGCATATGGGCTCGCTGGCTGCAGTATTCCCGGGTTCATTAG
ATCCTAAGGTACCTAATTGCCTAGAAAGGAGCAGACGATATGGCGTCGCTCCCTGCAGGTCGACTCTAGAAACCA
GCAGAGCATATGGGCTCGCTGGCTGCAGTATTCCCGGGTTCATTAGATCCTAAGGTACCTAATTGCCTAGAAAGG
AGCAGACGATATGGCGTCGCTCCCTGCAGGTCGACTCTAGAAACCAGCAGAGCATATGGGCTCGCTGGCTGCAGT
ATTCCCGGGTTCATTAGATCCTAAGGTACCTAATTGCCTAGAAAGGAGCAGACGATATGGCGTCGCTCCCTGCAGG
TCGACTCTAGAAACCAGCAGAGCATATGGGCTCGCTGGCTGCAGTATTCCCGGGTTCATTAGATCCTAAGGTACCT
AATTGCCTAGAAAGGAGCAGACGATATGGCGTCGCTCCCTGCAGGTCGACTCTAGAAACCAGCAGAGCATATGGG
CTCGCTGGCTGCAGTATTCCCGGGTTCATTAGATCCTAAGGTACCTAATTGCCTAGAAAGGAGCAGACGATATGGC
GTCGCTCCCTGCAGGTCGACTCTAGAAACCAGCAGAGCATATGGGCTCGCTGGCTGCAGTATTCCCGGGTTCATTA
GATCCTAAGGTACCTAATTGCCTAGAAAGGAGCAGACGATATGGCGTCGCTCCCTGCAGGTCGACTCTAGAAACC
AGCAGAGCATATGGGCTCGCTGGCTGCAGTATTCCCGGGTTCATTAGATCCTAAGGTACCTAATTGCCTAGAAAG
GAGCAGACGATATGGCGTCGCTCCCTGCAGGTCGACTCTAGAAACCAGCAGAGCATATGGGCTCGCTGGCTGCAG
TATTCCCGGGTTCATTAGATCCTAAGGTACCTAATTGCCTAGAAAGGAGCAGACGATATGGCGTCGCTCCCTGCAG
GTCGACTCTAGAAACCAGCAGAGCATATGGGCTCGCTGGCTGCAGTATTCCCGGGTTCATTAGATCCTAAGGTACC
TAATTGCCTAGAAAGGAGCAGACGATATGGCGTCGCTCCCTGCAGGTCGACTCTAGAAACCAGCAGAGCATATGG
GCTCGCTGGCTGCAGTATTCCCGGGTTCATTAGATCCTAAGGTACCTAATTGCCTAGAAAGGAGCAGACGATATG
GCGTCGCTCCCTGCAGGTCGACTCTAGAAACCAGCAGAGCATATGGGCTCGCTGGCTGCAGTATTCCCGGGTTCAT
TAGATCCTAAGGTACCTAATTGCCTAGAAAGGAGCAGACGATATGGCGTCGCTCCCTGCAGGTCGACTCTAGAAA
CCAGCAGAGCATATGGGCTCGCTGGCTGCAGTATTCCCGGGTTCATTAGATC

Xbp1 reporter
ATGGGCGCCATCATACACGCGGCCGCAATCATcggtccgGGTGGATCTGGAGGTGGAGGTTCTGGAGGAGAAGAA
CTTTTGAGCAAGAATTATCATCTTGAGAACGAAGTGGCTCGTCTTAAGAAAGGTTCTGGCAGTGGAGAAGAACTG
CTTTCAAAGAATTACCACCTGGAAAATGAGGTAGCTAGACTGAAAAAGGGGAGCGGAAGTGGGGAGGAGTTGCT
GAGCAAAAAATTATCATTTGGAGAACGAAGTAGCACGACTAAAGAAAGGGTCCGGATCGGGTGAGGAGTTACTCT
CGAAAAATTATCATCTCGAAAACGAAGTGGCTCGGCTAAAAAAGGGCAGTGGTTCTGGAGAAGAGCTATTATCTA
AAAACTACCACCTCGAAAATGAGGTGGCACGCTTAAAAAAGGGAAGTGGCAGTGGTGAAGAGCTACTATCCAAG
AATTATCATCTTGAGAACGAGGTAGCGCGTTTGAAGAAGGGTTCCGGCTCAGGAGAGGAACTGCTCTCGAAGAAC
TATCATCTTGAAAATGAGGTCGCTCGATTAAAAAAGGGATCGGGCAGTGGTGAGGAACTACTTTCAAAGAATTAC

CACCTCGAAAACGAAGTAGCTCGATTAAAGAAAGGTTCAGGGTCGGGTGAAGAATTACTGAGTAAAAATTATCAT
CTGGAAAATGAGGTAGCGAGACTAAAAAAGGGGAGTGGTTCTGGCGAAGAGTTGCTATCGAAAAATTATCATCTT
GAGAACGAAGTTGCTAGGCTCAAAAAGGGCTCAGGCTCAGGCGAGGAGTTGCTCTCGAAAAACTACCACTTGGA
AAATGAGGTCGCGAGGTTGAAAAAGGGGAGCGGGTCGGGCGAGGAGTTATTGAGCAAAAACTATCATTTAGAG
AACGAAGTCGCGCGCTTAAAGAAAGGCTCGGGCTCGGGCGAAGAACTCTTATCGAAGAACTACCACCTCGAAAAT
GAGGTCGCCAGGTTGAAAAAGGGCAGTGGCAGCGGGGAGGAACTCTTGAGCAAGAACTACCACTTGGAGAATG
AGGTCGCGAGATTGAAGAAAGGGTCGGGGAGCGGCGAGGAATTGCTCAGCAAGAATTATCATTTGGAGAACGA
AGTCGCCAGGCTCAAGAAAGGCTCGGGGTCGGGGGAGGAATTGTTGAGTAAAAACTACCACTTGGAAAATGAAG
TCGCCAGGCTCAAAAAAGGGAGTGGGAGCGGCGAAGAGTTATTGAGCAAAAATTACCACTTGGAGAACGAAGTG
GCAAGGCTCAAGAAAGGGAGCGGCAGCGGGGAGGAGCTCTTATCGAAGAACTACCACTTAGAGAATGAAGTCGC
CCGCTTGAAGAAAGGCTCGGGGAGCGGGGAAGAGCTCTTGAGCAAGAACTACCACTTGGAAAATGAGGTGGCGC
GCTTGAAGAAAGGGAGCGGGAGCGGGGAAGAGTTACTATCTAAGAATTATCATCTCGAGAACGAGGTGGCTCGA
CTAAAGAAGGGCTCCGGCAGTGGGGAGGAACTCCTGTCGAAGAACTATCATCTTGAAAATGAGGTTGCAAGACTT
AAAAAGGGGTCCGGATCAGGTGAGGAACTACTCAGTAAGAATTACCACCTGGAAAACGAAGTTGCACGTTTGAA
GAAAGGATCAGGATCAGGCGAAGAACTGCTCTCAAAAGATTATCATTTGGAAAATGAGGTTGCACGTTTAAAAAA
GGGAAGTGGCAGTGGTGAGGAACTTCTGTCGAAAAATTATCATCTCGAGAATGAAGTAGCCCGACTTAAAAAGG
GTTCTGGCTCGGGTCAGCGGCCGCACCGGTCAGCAGTGGAGGACAGCACGCTGCAAGTAGTGGTACGGGTGCGG
CCCCCCACCCCTCGGGAGCTGGACAGTCAGCGGCGGCCAGTGGTTCAGGTGGTGGACGAGCGGGTGCTGGTGTT
TAACCCTGAGGAGCCCGATGGAGGGTTCCCTGGCCTGAAATGGGGTGGCACCCATGATGGCCCCAAGAAGAAGG
GCAAAGACCTGACGTTTGTCTTTGACCGGGTCTTTGGCGAGGCGGCCACCCAACAGGACGTGTTCCAGCACACCA
CGCACAGCGTCCTGGACAGCTTCCTCCAGGGCTACAACTGCTCAGTGTTTGCCTACGGGGCCACCGGGGCTGGGA
AGACACACACCATGCTGGGAAGGGAGGGGGACCCCGGCATCATGTACCTGACCACCGTGGAACTGTACAGGCGC
CTGGAGGCCCGCCAGCAGGAGAAGCACTTCGAGGTGCTCATCAGCTACCAGGAGGTGTATAATGAACAGATCCAT
GACCTCCTGGAGCCCAAGGGGCCCCTTGCCATCCGCGAGGACCCCGACAAGGGGGTGGTGGTGCAAGGACTTTC
TTTCCACCAGCCAGCCTCAGCCGAGCAGCTGCTGGAGATACTGACCAGGGGGAACCGTAACCGCACGCAGCACCC
CACTGATGCCAACGCGACTTCCTCCCGCTCCCATGCCATCTTCCAGATCTTTGTGAAGCAGCAGGACCGGGTTCCA
GGACTGACCCAGGCTGTCCAGGTGGCCAAGATGAGCCTGATTGACCTGGCTGGCTCAGAGCGGGCATCCAGCAC
CCATGCGAAGGGGGAGCGGCTGCGGGAGGGGGCCAACATCAACCGCTCTCTGCTGGCGCTCATCAACGTCCTCA
ATGCCTTGGCCGATGCAAAGGGCCGCAAGACCGCTGTGCCCTACGCGGACAGCGCACTGACCCGCCTGCTCAAAG
ACTCCCTCGGGGGCAACTGCCGCACAGTGATGATCGCTGCCATCAGCCCCTCCAGCCTGACCTACGAGGACACGT
ATAATACCCTCAAATATGCCGACCGGGCCAAGGAGATCAGGCTCTCGCTGAAGAGCAATGTGACCAGCCTGGACT
GTCACATCAGCCAGTATGCTACCATCTGCCAACAGCTCCAGGCTGAGGTAGCCGCTCTGAGGAAGAAGCTCCAAG
TGTATGAGGGGGGAGGCCAGCCCCCACCACAGGACCTCCCAGGATCTCCCAAGTCGGGACCACCACCAGAACACC
TTCCCAGCTCCCCCTTGCCACCCCACCCTCCCAGCCAGCCCTGCACCCCAGAGCTCCCTGCAGGGCCTAGAGCCCTT
CAAGAGGAGAGTCTGGGGATGGAGGCCCAGGTGGAGAGGGCCATGGAAGGGAACTCTTCAGACCAGGAGCAGT
CCCCAGAGGATGAGGATGAAGGCCCAGCTGAGGAGGTTCCAACCCAGATGCCAGAGCAGAACCCCACACATGCA
CTGCCAGAGTCCCCTCGCCTGACCCTGCAGCCCAAGCCAGTCGTGGGCCACTTCTCAGCACGGGAACTGGATGGG
GACCGTTCTAAGCGGTTGGCCCTAAAGGTGCTGTGCGTTGCCCAGCGGCAGTACTCCCTGCTCCAAGCAGCCAACC
TCCTGACGCCCGACATGATCACAGAGTTTGAGACCCTACAGCAGCTGGTGCAAGAGGAAAAAATTGAGCCTGGGG
CAGAGGCCTTGAGGACTTCAGGCCTGGCCAGGGGGGCACCTCTGGCTCAGGAGCTGTGTTCAGAGTCAAAGCCTC
CAGGATACACTGGCCCTGTGACCCGGACTATGGCGAGGCGACTGAGTGGCCCCCTGCACACCCTGGGAATCCCGC
CTGGACCCAACTGCACCCCAGCCCAGGGGTCCCGATGGCCCATGGAGAAGAAGAGGAGGAGACCAAGCGCCTTG
GAGGCAGACAGTCCCATGGCCCCAAAGCGGGGCACCAAGCGCCAGCGCCAGTCCTTCCTGCCCTGCCTAAGGAGA
GGGTCTCTGCCTGACACCCAACCTTCACAGGGGCCCAGCACCCCCAAAGGAGAAAGGGCCTCCTCCCCCTGCCATT
CCCCTCGCGTTTGCCCAGCCACAGTCATCAAAAGCCGGGTGCCCCTGGGCCCTTCCGCCATGCAGAACTGCTCCAC
CCCGCTGGCTCTGCCCACTCGAGACCTCAATGCCACCTTTGATCTCTCTGAGGAGCCTCCCTCAAAGCCCAGTTTCC
ATGAATGCATTGGCTGGGACAAAATACCCCAGGAGCTGAGCAGGCTGGACCAGCCCTTCATCCCCAGGGCACCTG
TGCCCCTGTTCACCATGAAGGGCCCCAAGCCAACATCTTCCCTCCCTGGGACCTCTGCCTGCAAGAAGAAGCGCGT

TGCGAGTTCCTCAGTCTCCCATGGCCGCAGCCGCATCGCCCGCCTCCCCAGCAGCACTTTGAAGAGGCCAGCTGGG
CCCCTTGTACTCCCAGAGCTGCCCTTGAGTCCCCTGTGCCCTAGCAACCGGAGGAATGGAAAGGACCTCATCAGG
GTGGGGAGAGCACTCTCAGCAGGGAACGGCGTCACCAAGGTGTCCGATAAGGACCCCGTGCCCTACCAACCTCCA
TTCCTGTGTCAATGGGGCCGACATCAGCCAGCCTGGAAACCCTTGATGAATGGCGGCGGCGCCGCCAGCGCCAAA
GCCATCTAA
