# Supplementary figures and images for: Dynamics of Translation of Single mRNA Molecules In Vivo
Source: Cell. 2016 May 5;165(4):976–89. doi: 10.1016/j.cell.2016.04.034 (PMC4889334; doi:10.1016/j.cell.2016.04.034)

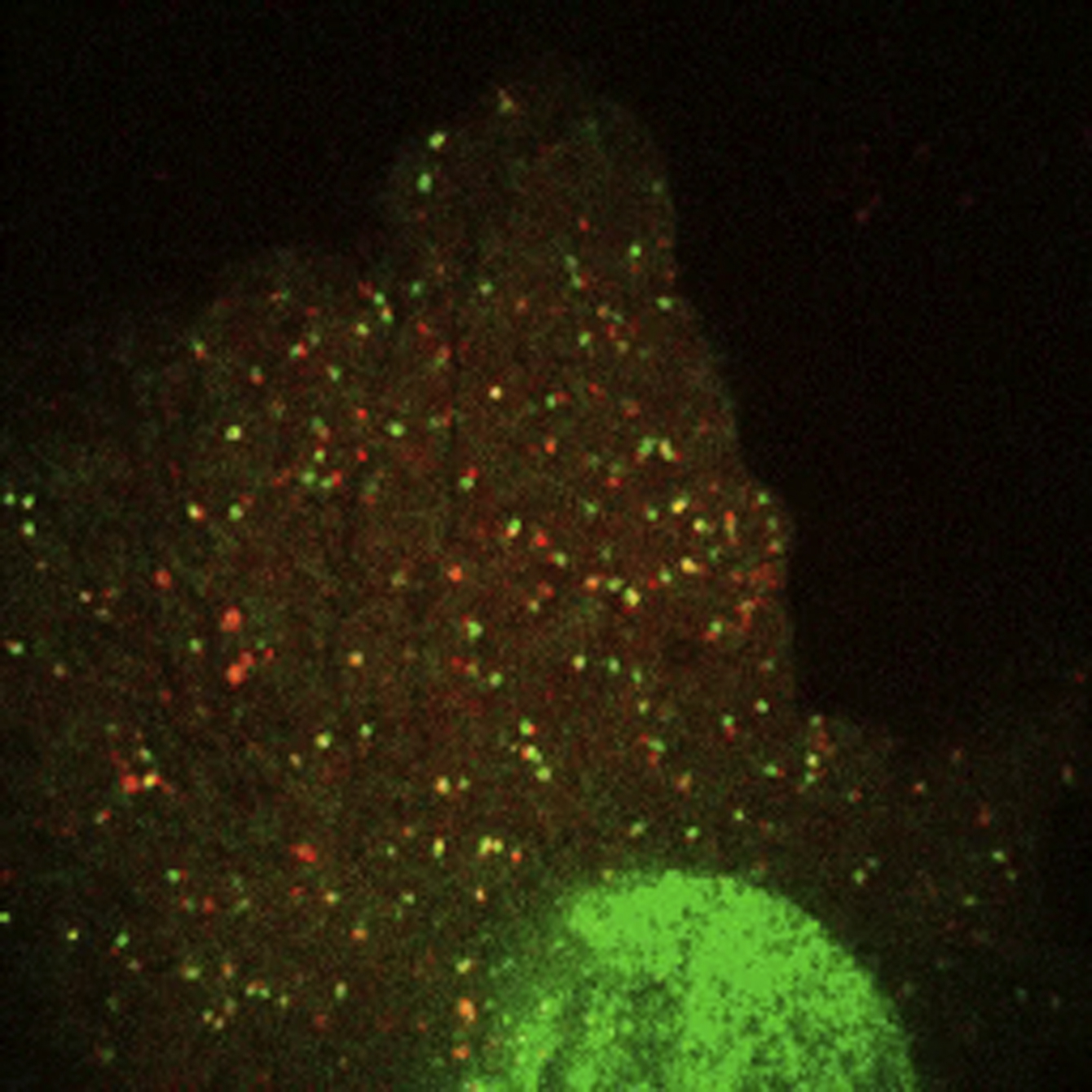

Supplement: Movie S1. Visualizing Translation of Single mRNAs in Live Cells, Related to Figure 1 — A U2OS cell expressing scFv-GFP and PP7-3xmCherry was transfected with a translation reporter (SunTag24x-Kif18b-PP724x). Images were acquired every 30 s (movie duration is 16 min) on a spinning disk confocal microscope. Red and green foci do not perfectly overlap because two color images were acquired sequentially and foci move rapidly in the cell. Movie field of view is 41 × 41 μm. [file mmc2.jpg]

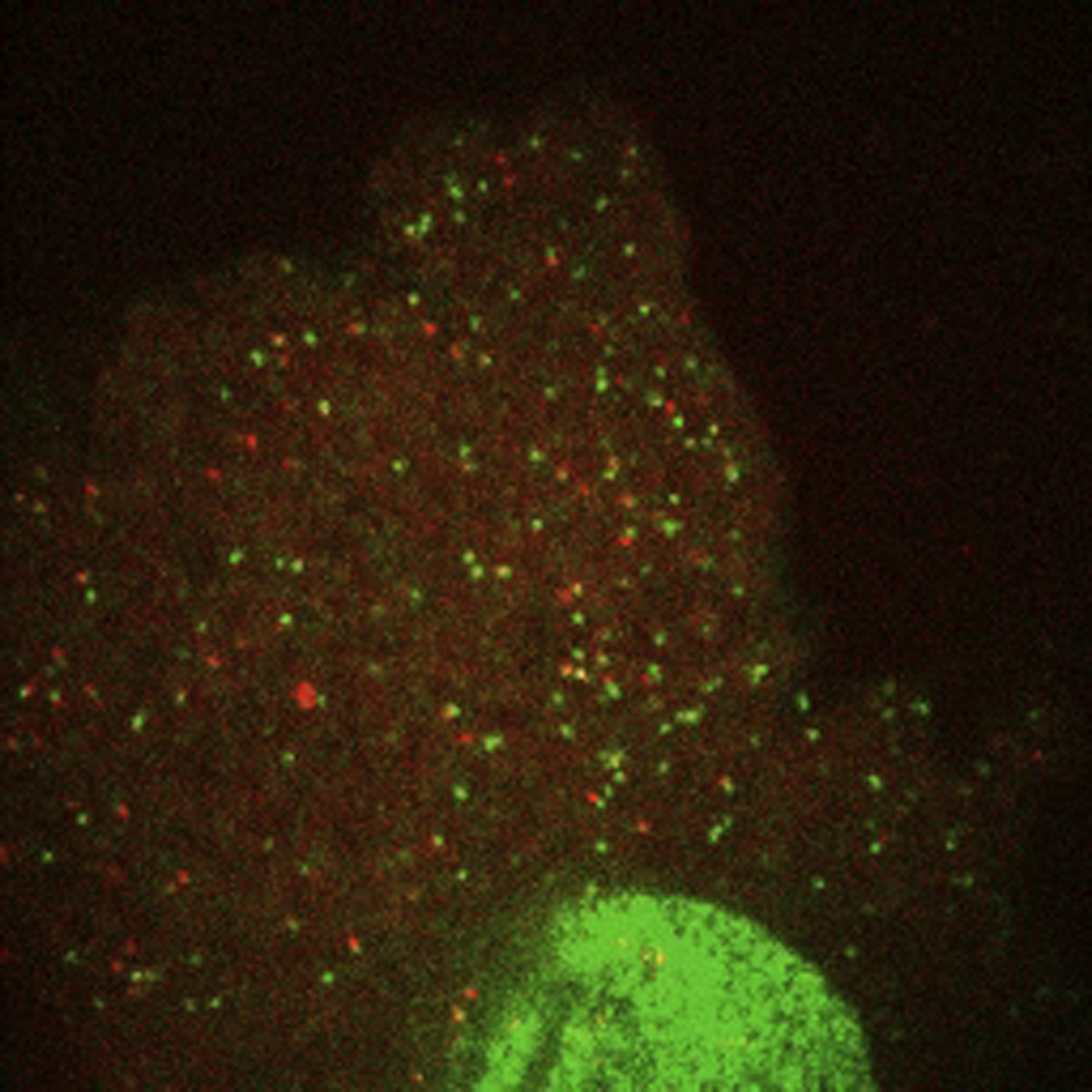

Supplement: Movie S2. GFP Foci Represent Sites of Translation, Related to Figure 1 — A U2OS cell expressing scFv-GFP and PP7-3xmCherry was transfected with a translation reporter (SunTag24x-Kif18b-PP724x). Images were acquired every 30 s (movie duration is 25 min) on a spinning disk confocal microscope. At frame 8 (3.5 min after the start of the movie), 100 μg/mL puromycin is added to the cell culture medium. Bright scFv-GFP spots can be observed at the beginning of the movie, which rapidly disappear upon puromycin addition, indicating that they are sites of translation. Red and green foci do not perfectly overlap because two color images were acquired sequentially and foci move rapidly in the cell. Movie field of view is 41 × 41 μm. [file mmc3.jpg]

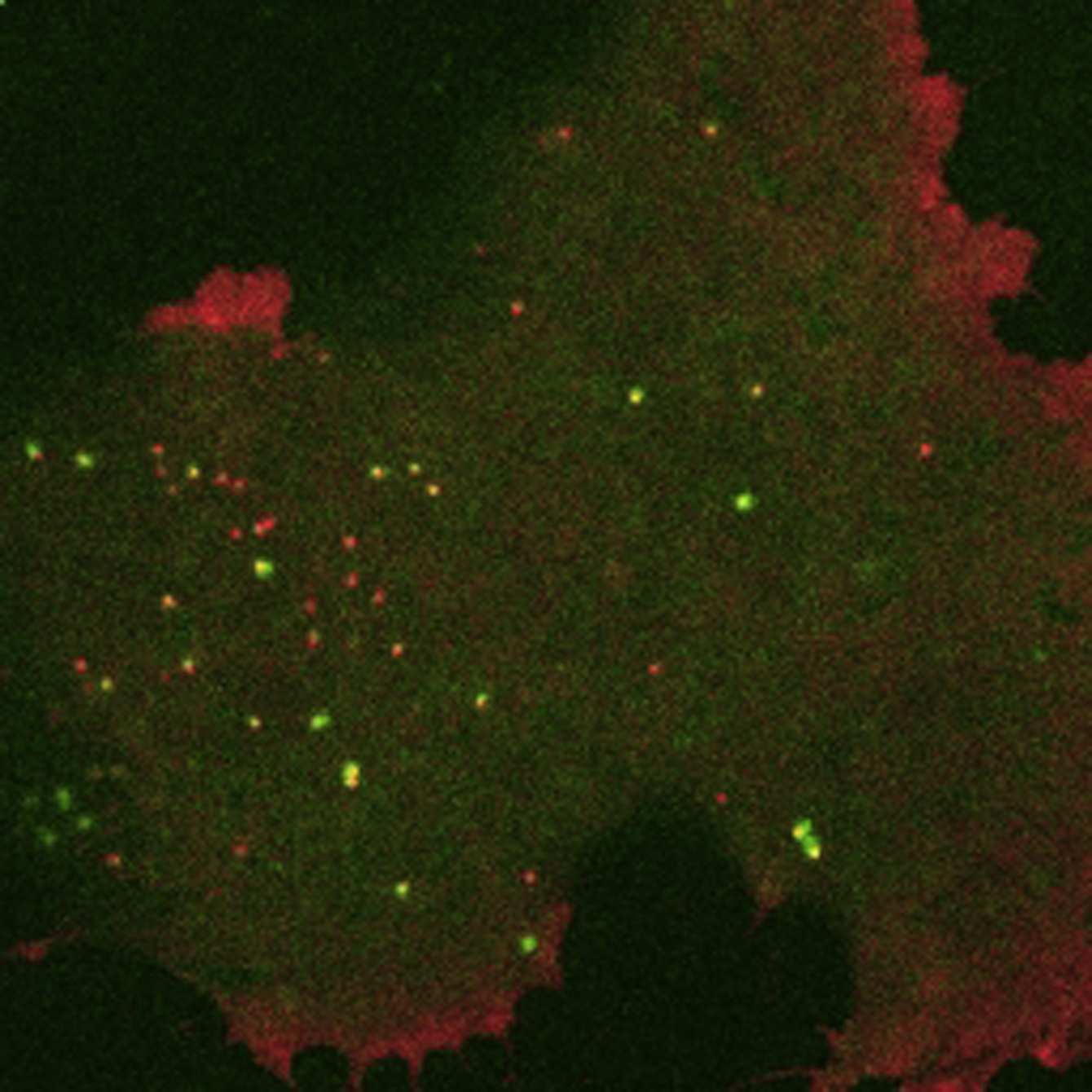

Supplement: Movie S3. Visualizing Translation of Membrane-Tethered mRNAs, Related to Figure 1 — A U2OS cell expressing scFv-GFP (green) and PP7-2xmCherry-CAAX (red) was transfected with a translation reporter (SunTag24x-Kif18b-PP724x). Images were acquired every 60 s (movie duration is 70 min) on a spinning disk confocal microscope focusing near the bottom plasma membrane of the cell. Individual mRNAs can be tracked for the duration of the movie, undergoing many rounds of translation. Movie field of view is 41 × 41 μm. [file mmc4.jpg]

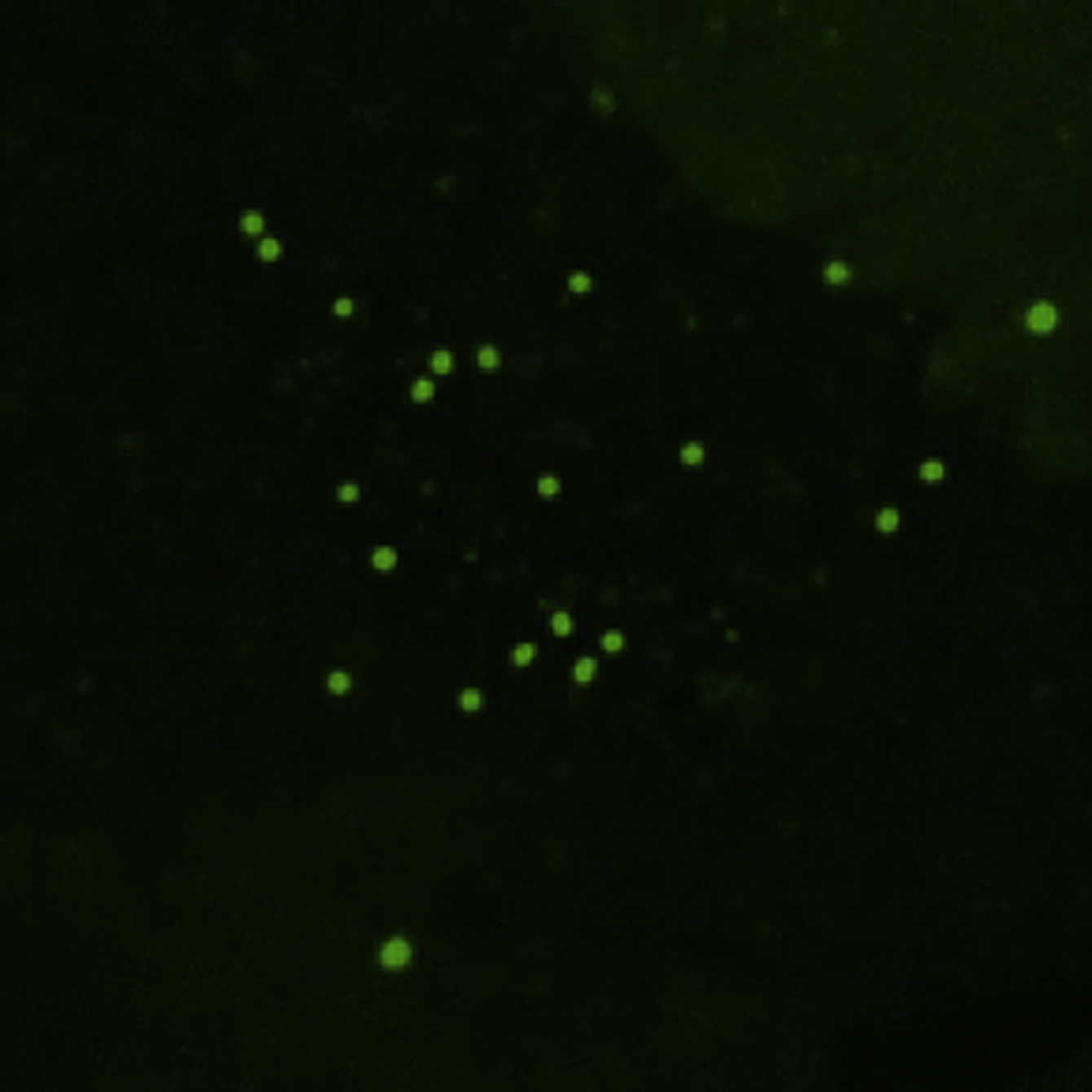

Supplement: Movie S4. Ribosome Translocation Dynamics on Single mRNAs, Related to Figure 2 — A U2OS cell expressing scFv-GFP (green) and PP7-2xmCherry-CAAX (not shown) was transfected with a translation reporter (SunTag24x-Kif18b-PP724x). Images were acquired every 30 s (movie duration is 45 min) on a spinning disk confocal microscope focusing near the bottom plasma membrane of the cell. Harringtonine was added 2 min after the start of the movie. Bright green dots are translation sites, which become progressively dimmer after harringtonine addition due to ribosome runoff. Movie field of view is 40 × 40 μm. [file mmc5.jpg]

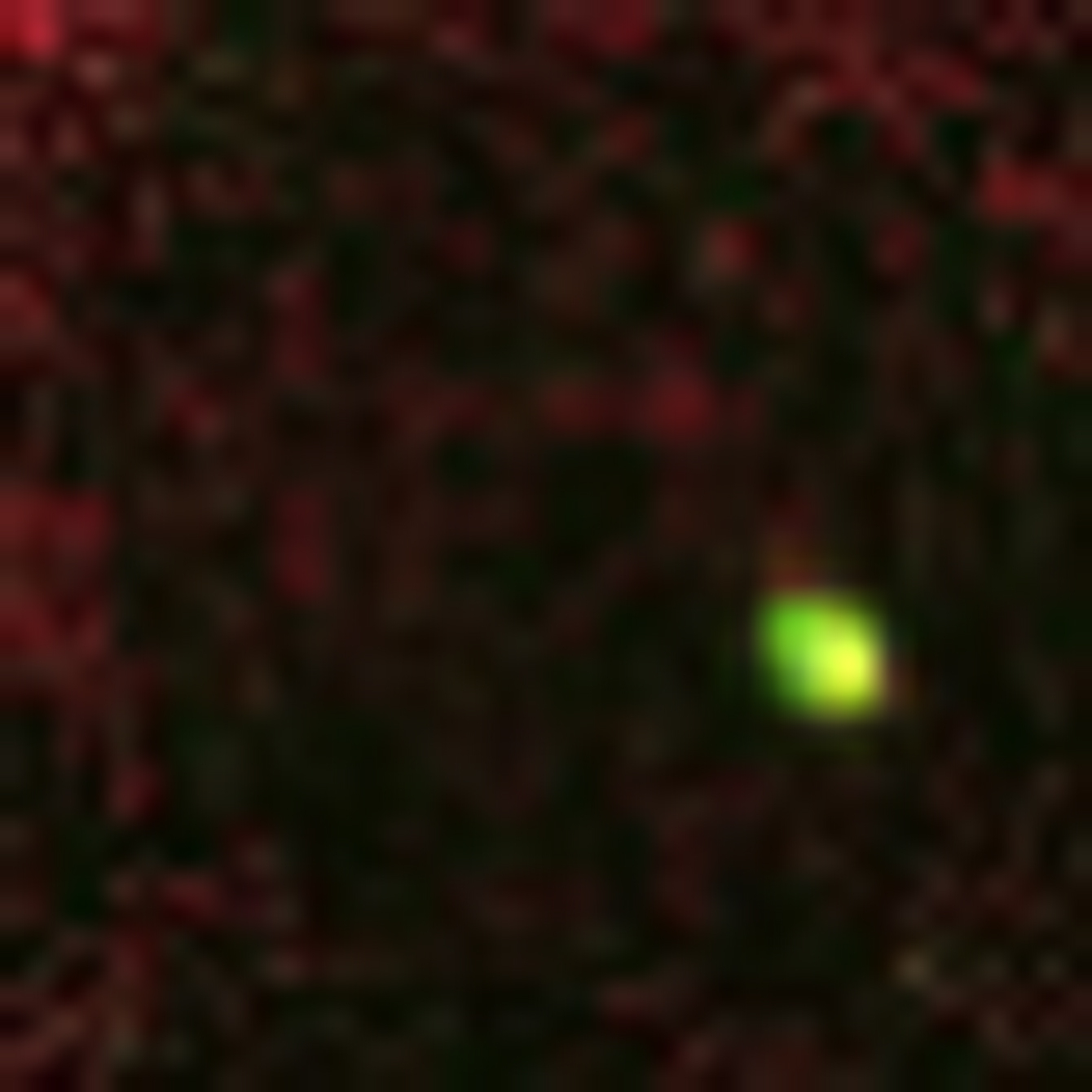

Supplement: Movie S5. Shutdown of Translation of a Single mRNA Molecule, Related to Figure 3 — A U2OS cell expressing scFv-GFP (green) and PP7-2xmCherry-CAAX (red) was transfected with a translation reporter (SunTag24x-Kif18b-PP724x). Images were acquired every 30 s (movie duration is 25 min) on a spinning disk confocal microscope focusing near the bottom plasma membrane of the cell. Translation on the mRNA molecule is abruptly shut down. Movie field of view is 6.6 × 6.6 μm. [file mmc6.jpg]

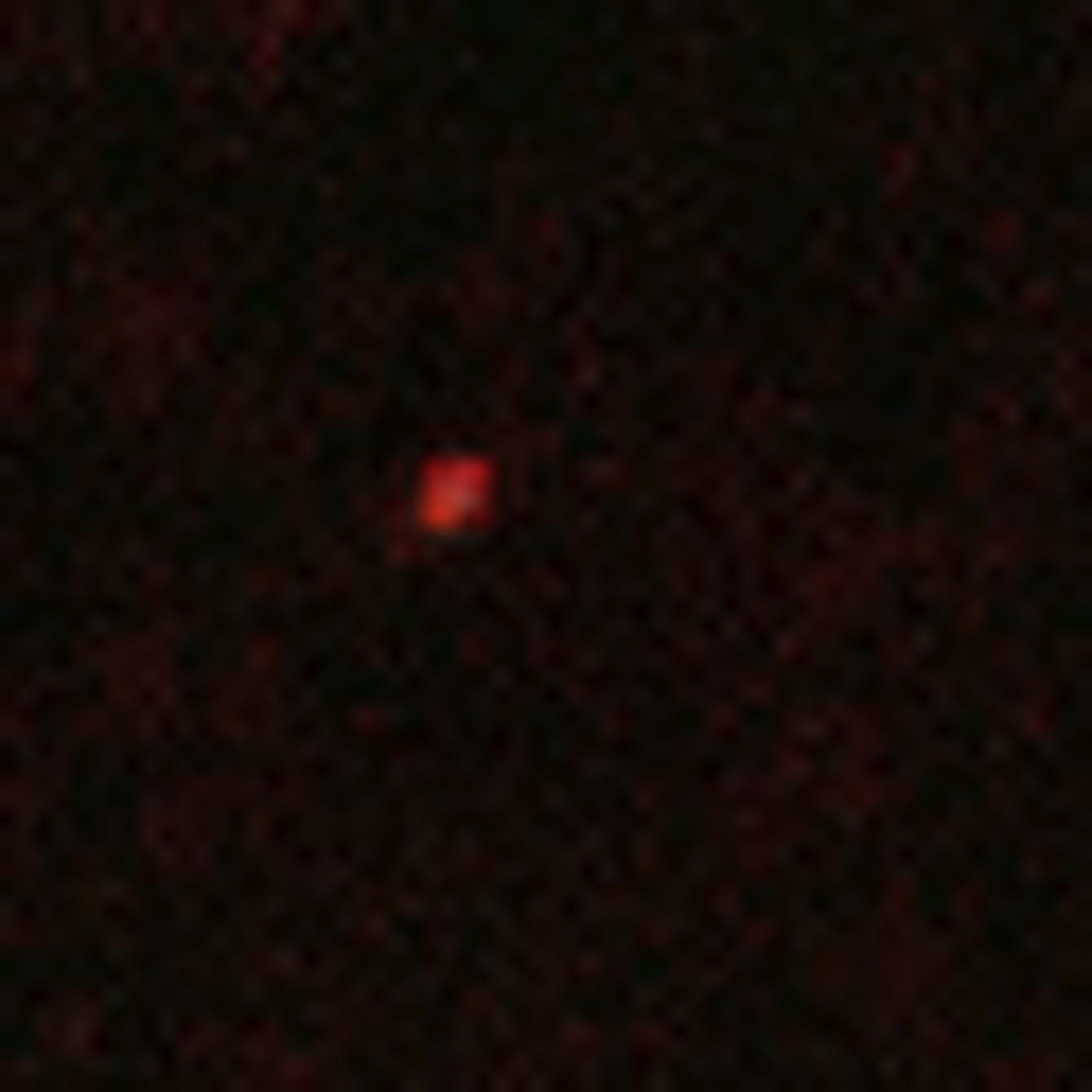

Supplement: Movie S6. Polysome Build-Up on Newly Transcribed mRNAs, Related to Figure 4 — A U2OS cell expressing scFv-GFP (green) and PP7-2xmCherry-CAAX (red) was transfected with a translation reporter (SunTag24x-Kif18b-PP724x) under control of a doxycycline inducible promoter. Images were acquired every 30 s (movie duration is 16 min) on a spinning disk confocal microscope focusing near the bottom plasma membrane of the cell. Doxycycline was added approximately 20 min before the start of the movie to induce transcription of the reporter gene. Several new red spots appear during the movie, which are likely newly transcribed mRNAs. These mRNAs rapidly initiate translation. Movie field of view is 8.4 × 8.4 μm. [file mmc7.jpg]

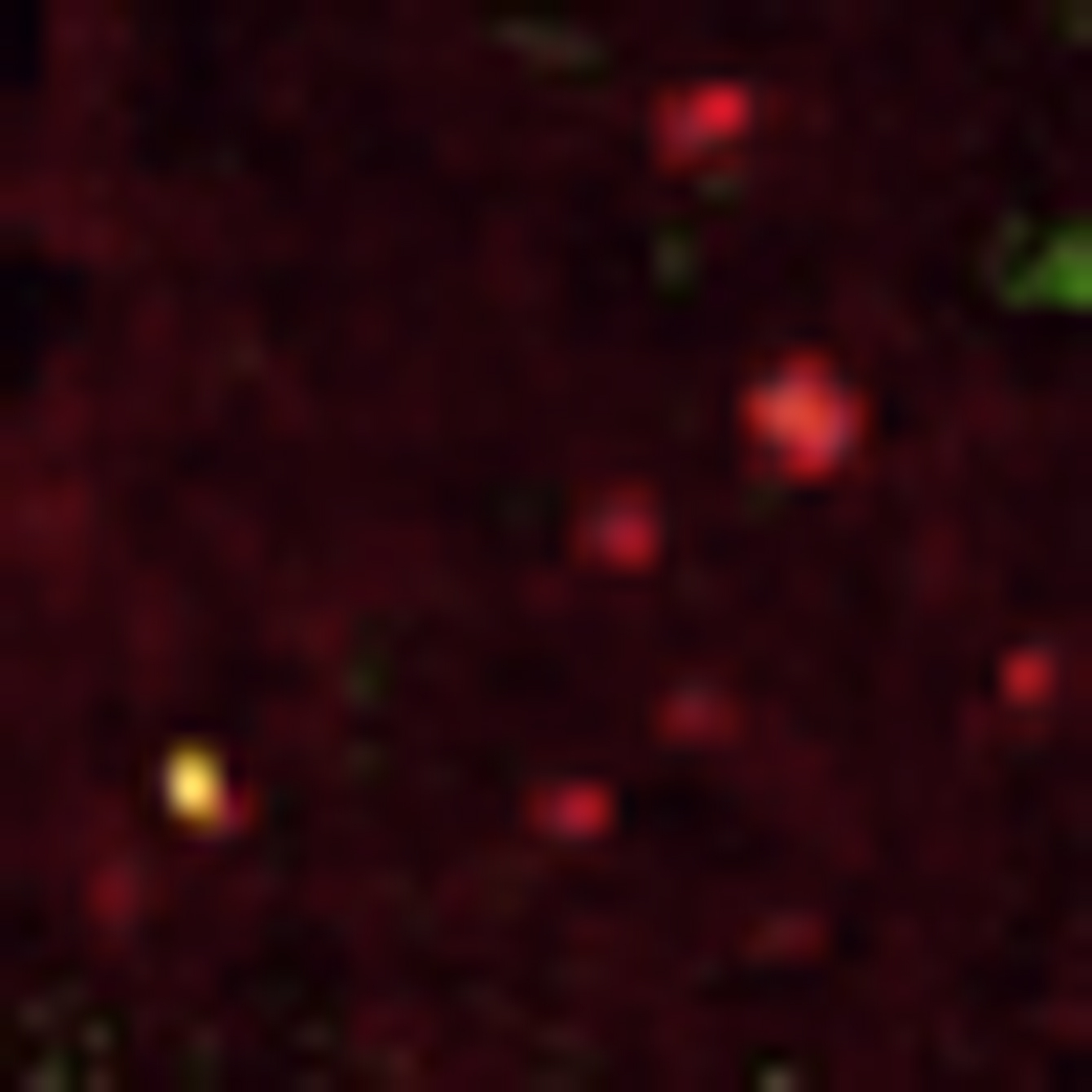

Supplement: Movie S7. Observing Single Ribosomes Translating an mRNA Molecule, Related to Figure 7 — A U2OS cell expressing scFv-GFP (green) and PP7-2xmCherry-CAAX (red) is transfected with a translation reporter fused to the Emi1 5′ UTR_long that strongly represses translation initiation (5′ UTR_long-SunTag24x-Kif18b-PP724x). Images were acquired every 30 s (movie duration is 31 min) on a spinning disk confocal microscope focusing near the bottom plasma membrane of the cell. Brief flashes of green can be observed on individual mRNA molecules, indicating a single ribosome translating the mRNA molecule. An offset was applied to the images to reduce the GFP background to allow easier detection of single ribosome transits. Movie field of view is 3.4 × 3.4 μm. [file mmc8.jpg]
