# Supplementary material for: Dynamics of Translation of Single mRNA Molecules In Vivo
Source: Cell. 2016 May 5;165(4):976–89. doi: 10.1016/j.cell.2016.04.034 (PMC4889334; doi:10.1016/j.cell.2016.04.034)
Supplement: Document S2. Article plus Supplemental Information [file mmc9.pdf]

Resource

Cell

Dynamics of Translation of Single mRNA Molecules In Vivo

Graphical Abstract

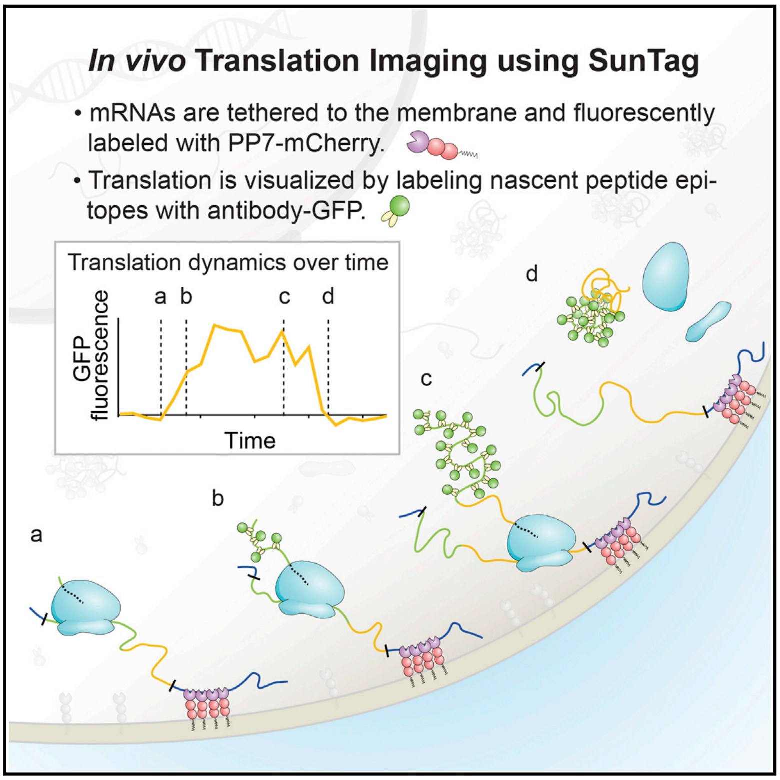

Highlights

- A new imaging method to study dynamics of translation of single mRNAs in vivo
- Measurements of ribosome initiation and elongation rates on single mRNA molecules
- mRNAs can rapidly switch between a translating and non-translating state
- Substantial heterogeneity in ribosome pausing on the Xbp1/Hac1 pause sequence

Authors

Xiaowei Yan, Tim A. Hoek, Ronald D. Vale, Marvin E. Tanenbaum

Correspondence

m.tanenbaum@hubrecht.eu

In Brief

An imaging method is developed for visualizing translation of single mRNA molecules in living cells over long periods of time (>1 hr), revealing a surprising heterogeneity in the translation of mRNA molecules expressed from the same gene in a single cell.

CrossMark

Yan et al., 2016, Cell *165*, 976–989

May 5, 2016 ©2016 Elsevier Inc.

http://dx.doi.org/10.1016/j.cell.2016.04.034

CellPress

Cell

Resource

Dynamics of Translation of Single mRNA Molecules In Vivo

Xiaowei Yan,[2] Tim A. Hoek,[1] Ronald D. Vale,[2] and Marvin E. Tanenbaum[1,*]
[1]Hubrecht Institute, The Royal Netherlands Academy of Arts and Sciences (KNAW) and University Medical Center Utrecht, Utrecht 3584CT, the Netherlands
[2]Department of Cellular and Molecular Pharmacology, Howard Hughes Medical Institute, University of California, San Francisco, San Francisco, CA 94158-2517, USA
*Correspondence: m.tanenbaum@hubrecht.eu
http://dx.doi.org/10.1016/j.cell.2016.04.034

SUMMARY

Regulation of mRNA translation, the process by which ribosomes decode mRNAs into polypeptides, is used to tune cellular protein levels. Currently, methods for observing the complete process of translation from single mRNAs in vivo are unavailable. Here, we report the long-term (>1 hr) imaging of single mRNAs undergoing hundreds of rounds of translation in live cells, enabling quantitative measurements of ribosome initiation, elongation, and stalling. This approach reveals a surprising heterogeneity in the translation of individual mRNAs within the same cell, including rapid and reversible transitions between a translating and non-translating state. Applying this method to the cell-cycle gene Emi1, we find strong overall repression of translation initiation by specific 5′ UTR sequences, but individual mRNA molecules in the same cell can exhibit dramatically different translational efficiencies. The ability to observe translation of single mRNA molecules in live cells provides a powerful tool to study translation regulation.

INTRODUCTION

Precise tuning of the expression of each gene in the genome is critical for many aspects of cell function. The level of gene expression is regulated at multiple distinct steps, including transcription, mRNA degradation, and translation (Schwanhäusser et al., 2011). Regulation of all of these steps in gene expression is important, though the relative contribution of each control mechanism varies for different biological processes (Brar et al., 2012; Jovanovic et al., 2015; Peshkin et al., 2015; Tanenbaum et al., 2015; Vardy and Orr-Weaver, 2007).

Measuring the translation rate from individual mRNAs over time provides valuable information on the mechanisms of translation and translational regulation. In vitro experiments, mainly using bacterial ribosomes, have revealed exquisite information on ribosome translocation dynamics at the single molecule level (Blanchard, 2009; Chen et al., 2012; Cornish et al., 2008; Fei et al., 2008; Wen et al., 2008; Zaher and Green, 2009), but such methods have not yet been applied in vivo. In contrast, a genome-wide snapshot of the translational efficiency of endogenous mRNAs in vivo can be obtained through the method of ribosomal profiling (Ingolia et al., 2009; Ingolia et al., 2011). However, this method requires averaging of many cells and provides limited temporal information because of the requirement to lyse cells to make these measurements. Single cell imaging studies have succeeded in measuring average protein synthesis rates (Aakalu et al., 2001; Brittis et al., 2002; Han et al., 2014; Leung et al., 2006; Tanenbaum et al., 2015; Yu et al., 2006), observing the first translation event of an mRNA (Halstead et al., 2015), localizing sub-cellular sites of translation by co-localizing mRNAs and ribosomes (Katz et al., 2016; Wu et al., 2015), and staining nascent polypeptides with small molecule dyes (Rodriguez et al., 2006).

While ribosomal profiling and other recently developed methods have provided many important new insights into the regulation of translation, many questions cannot be addressed using current technologies. For example, it is unclear to what extent different mRNA molecules produced in a single cell from the same gene behave similarly. Many methods to study translation in vivo require averaging of many mRNAs, masking potential differences between individual mRNA molecules. Such differences could arise from differential post-transcriptional regulation, such as nucleotide modifications (Choi et al., 2016; Wang et al., 2015), differential transcript lengths through use of alternative transcriptional start sites (Rojas-Duran and Gilbert, 2012) or polyadenylation site selection (Elkon et al., 2013; Gupta et al., 2014), differences in ribonucleic protein (RNP) composition (Wu et al., 2015), distinct intracellular localization (Hüttelmaier et al., 2005), or different states of RNA secondary structure (Babendure et al., 2006; Kertesz et al., 2010). Heterogeneity among mRNA molecules could have a profound impact on the total amount of polypeptide produced, as well as the localization of protein synthesis, but remains poorly studied. Furthermore, the extent to which translation of single mRNA molecules varies over time is also largely unknown. For example, translation may occur in bursts, rather than continuously (Tatavarty et al., 2012; Yu et al., 2006), and regulation of protein synthesis may occur by modulating burst size and/or frequency, which could occur either globally or on each mRNA molecule individually. In addition, the ability of an mRNA molecule to initiate translation may vary with time or spatial location, for example as cells progress through the cell cycle (Stumpf et al., 2013; Tanenbaum et al., 2015) or undergo active microtubule-based transport to particular cellular destinations

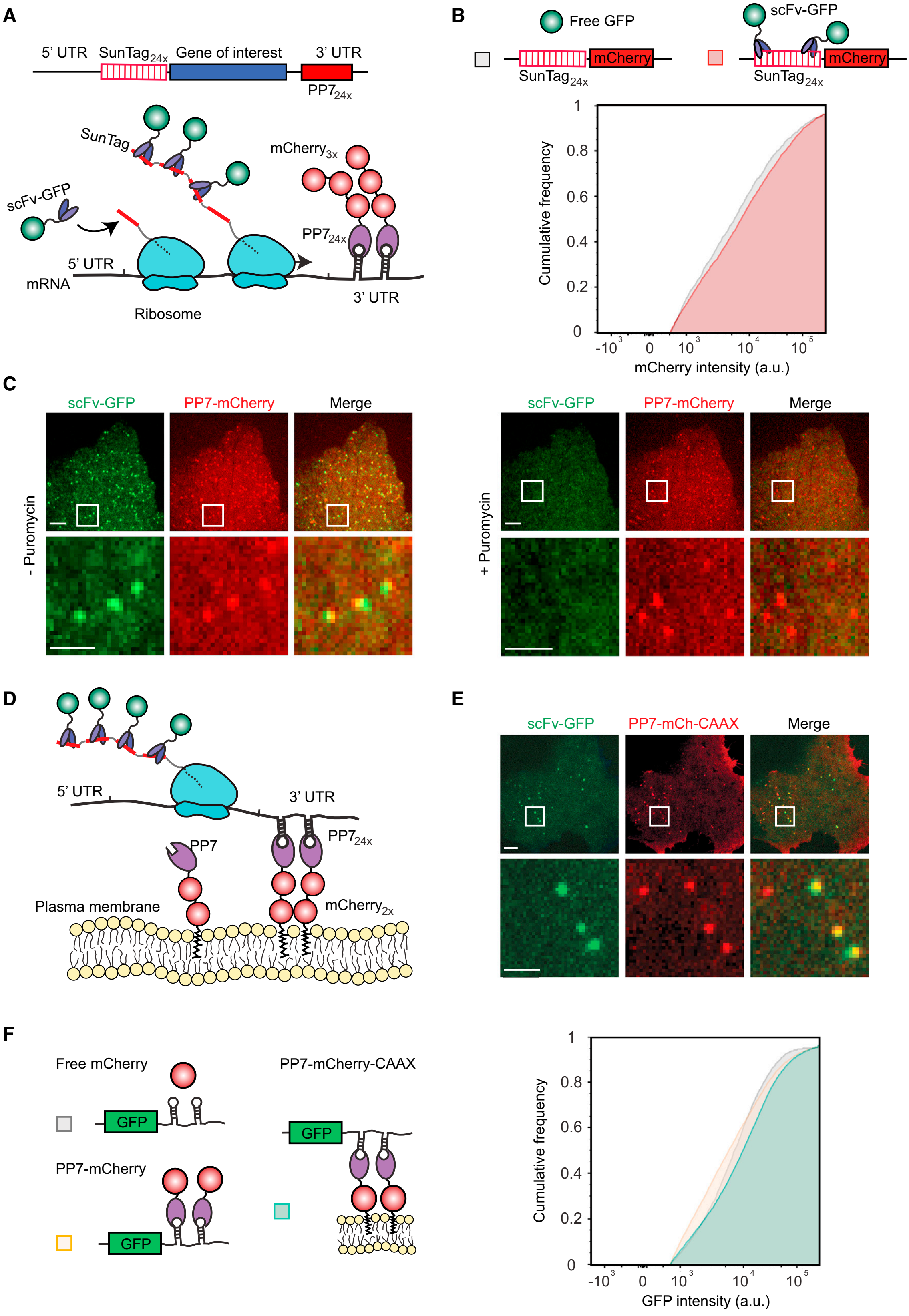

(legend on next page)

(Holt and Schuman, 2013). Such regulation could involve changes in the rates of translation initiation and/or the ribosome elongation. To address these questions, new methods are required for visualizing translation of single mRNA molecules in live cells over time.

Here, we present a method, based on the SunTag fluorescence tagging system that we recently developed (Tanenbaum et al., 2014), for measuring the translation of single mRNA molecules over long periods of time. Using this system, we have measured initiation, elongation, and stalling on individual mRNA molecules and have uncovered unexpected heterogeneity among different mRNA molecules encoded by the same gene within a single cell. Our system will be widely applicable to the study of mRNA translation in live cells.

RESULTS

An Assay for Long-Term Observation of Translation of Individual mRNAs

Observing the synthesis of a genetically encoded fluorescent protein, such as GFP, in vivo is difficult because of the relatively long maturation time required to achieve a fluorescent state. Thus, a GFP-fusion protein typically will not fluoresce until after its translation is completed. To overcome this temporal challenge and to create a sufficiently bright signal to observe protein synthesis from single mRNAs in vivo, we used our recently developed SunTag system (Tanenbaum et al., 2014). In this assay, cells are co-transfected with a reporter transcript containing an array of 24 SunTag peptides followed by a gene of interest, along with a second construct expressing a GFP-tagged single-chain intracellular antibody (scFv-GFP) that binds to the SunTag peptide with high affinity. As the SunTag peptides are translated and emerge from the ribosome exit tunnel, they are rapidly bound by the soluble and already fluorescent scFv-GFP (Figure 1A). Importantly, labeling of nascent chains using the SunTag antibody did not detectably alter protein synthesis rates of a reporter mRNA in human U2OS cells, as determined by FACS (fluorescence-activated cell sorting) analysis (Figure 1B). At the same time, the mRNA was fluorescently labeled by introducing 24 copies of a short hairpin sequence into the 3′ UTR and co-expressing the PP7 bacteriophage coat protein (Chao et al., 2008), which binds with high affinity to the hairpin sequence, fused to three copies of mCherry (PP7-mCherry) (Figure 1A).

When observed by spinning disk confocal microscopy, the co-expression of a reporter construct (SunTag$_{24x}$-Kif18b-PP7$_{24x}$, with Kif18b being a kinesin motor with a 2.5 kb coding sequence; Tanenbaum et al., 2011), scFv-GFP and PP7-mCherry, resulted in the appearance of a small number (10–50) of very bright green and red fluorescent spots per cell that co-migrated in time-lapse movies (Figure 1C; Movie S1). Spot tracking revealed that these spots diffused with a diffusion coefficient of 0.047 μm^2/s, which is slightly slower than previous measurements of mRNA diffusion (0.1–0.4 μm^2/s) (Katz et al., 2016), consistent with the fact that our reporter mRNA contains a larger open reading frame (4.4 kb versus 1.1 kb) and thus more associated ribosomes. In addition, we observed many dim GFP spots that did not co-migrate with an mCherry signal in time-lapse movies. The bright spots rapidly disappeared upon terminating translation by addition of a protein synthesis inhibitor, puromycin, which dissociates nascent polypeptides and ribosomes from mRNA (Figure 1C; Movie S2), indicating that they are sites of active translation where multiple ribosomes are engaged on a single mRNA molecule. The dim spots were unaffected by puromycin treatment, suggesting that they represent individual, fully synthesized SunTag$_{24x}$-Kif18b proteins that had already been released from the ribosome. Thus, this translation imaging assay allows visualization of ongoing translation of single mRNA molecules.

Rapid 3D diffusion of mRNAs makes it difficult to track single mRNAs for >1 min, as mRNAs continuously diffuse in and out of the z-plane of observation, and mRNAs regularly cross paths, complicating identification and tracking of individual mRNA molecules over time. To track mRNAs unambiguously for long periods of time, we added a CAAX sequence, a prenylation sequence that gets inserted into the inner leaflet of the plasma membrane, to the PP7-mCherry protein that served to tether mRNAs to the 2D plane of the plasma membrane (Figures 1D and 1E). As a result of many PP7-mCherry molecules clustering through their interaction with the multiple recognition sites on a single mRNA, bright red dots appeared on the plasma membrane at the bottom of the cell, representing a tethered mRNA molecule (Figure 1E). Tethered mRNA molecules co-migrated with scFv-GFP foci, indicating that they are sites of active translation (Figure 1E; Movie S3). Membrane tethering of the mRNA had minimal effects on the protein expression of a GFP reporter construct as analyzed by FACS (Figure 1F). While membrane tethering greatly improves the ability to visualize translation on single mRNA molecules over long periods of time and does not appear to grossly perturb mRNA translation, it is important to

Figure 1. Fluorescence Labeling of Nascent Chains to Visualize Translation of Single mRNA Molecules

(A) Schematic of nascent polypeptide labeling using the SunTag system and mRNA labeling (A) and membrane tethering (D) using the PP7 system.

(B) A mCherry-SunTag$_{24x}$ reporter gene was co-transfected with either GFP or scFv-GFP, and the expression of the SunTag$_{24x}$-mCherry reporter was determined by FACS (Experimental Procedures). Binding of the scFv-GFP to the SunTag nascent chain did not detectably alter protein expression.

(C) A representative U2OS cell is shown expressing scFv-GFP, PP7-3xmCherry, and the translation reporter (SunTag$_{24x}$-Kif18b-PP7$_{24x}$). Cytosolic translation sites (scFv-GFP) co-localize with mRNAs (PP7-3xmCherry). Ribosomes were dissociated from mRNA by addition of puromycin (right panel). Note that translation sites and mRNA do not perfectly overlap because of the brief time difference in acquiring GFP and mCherry images.

(D) Schematic of nascent polypeptide labeling and membrane tethering of the mRNA using the PP7 system.

(E) U2OS cells expressing scFv-GFP (green), PP7-2xmCherry-CAAX (red), and the translation reporter (SunTag$_{24x}$-Kif18b-PP7$_{24x}$). A single time point of the cell (top panel) and a zoomed-in view from the white-boxed area containing a few mRNAs (lower) are shown.

(F) U2OS cells were transfected with mCherry, PP7-mCherry, or PP7-mCherry-CAAX together with a GFP reporter transcript with 24 PP7 binding sites in the 3′ UTR, and GFP expression was analyzed by FACS (Experimental Procedures). Cumulative distribution of GFP expression levels from GFP-mCherry double positive cells are shown in (B) and (F) (n = 3 independent experiments). Scale bars, 5 μm (upper) and 2 μm (lower).

See also Figure S1 and Movies S1–S3.

note that some aspects of translation, especially localized translation, may be altered due to tethering (Discussion).

We first analyzed the PP7-mCherry spots observed on the plasma membrane to confirm that they contained only a single mRNA molecule. The fluorescence intensities of PP7-mCherry foci were very homogeneous (Figure S1A). Their absolute intensity was ~1.4-fold brighter, on average, than single, membrane-tethered SunTag$_{24x}$-CAAX proteins bound with scFv-mCherry, which is expected to contain 24 mCherry molecules (Figure S1B). PP7 binds as a dimer to the RNA hairpin, and each PP7 was tagged with two tandem copies of mCherry. Thus, mRNAs' spots could be expected to be four times as bright as single scFv-mCherry-SunTag$_{24x}$-CAAX spots, but previous studies suggested that only about half of PP7 binding sites may be occupied (Wu et al., 2015); thus, mRNA spots would be about 2-fold brighter than single mCherry-SunTag$_{24x}$ spots if they contain a single mRNA molecule but $\geq$4-fold brighter if they contained two or more mRNAs. These results are therefore most consistent with the mCherry-PP7 foci being single mRNA molecules rather than multiple copies of mRNAs. Further supporting this idea, we tracked 63 single mRNA foci for 30–45 min and did not find a single case in which one spot split into two, which would have been indicative of more than one mRNA molecule being present in a single spot.

Because single mRNAs were tethered to the plasma membrane through multiple PP7 molecules and thus through many CAAX membrane insertion domains, the 2D diffusion of mRNAs was extremely slow (1.06×10^{-3} $\mu m^2/s$, n = 211 mRNAs). This slow diffusion made it possible to track individual mRNAs and their associated translation sites for extended periods of time (mean tracking time >1 hr) (Figure S1C). Furthermore, the very slow diffusion rate of tethered mRNAs allowed us to image tethered translation sites using long exposure times (500–1000 ms). During this time interval, rapidly diffusing, non-tethered fully synthesized polypeptides only produced a blurred, diffuse image on the camera sensor, which enabled sites of translation to be easily distinguished from fully synthesized molecules (Figure S1D). Finally, to confirm that the scFv-GFP was binding to nascent SunTag peptides, we replaced the SunTag epitope peptides in our reporter mRNA with an unrelated nucleotide sequence (encoding BFP) and found no GFP foci formation near mRNAs (Figure S1E).

In conclusion, we have developed assays that enable both single mRNAs and their associated nascent translating polypeptides to be imaged over time. This general SunTag-based method can be performed with either freely diffusing mRNAs or mRNAs tethered to the plasma membrane, each of which has unique advantages depending on the specific biological question (Discussion). For further experiments in this study, we used the membrane-tethered system to follow translation for long periods of time.

Measurement of Ribosome Number, Initiation Rate, and Elongation Rate on Single mRNAs

To estimate the number of ribosomes translating each mRNA, we compared the scFv-GFP fluorescence intensity of translation sites with that of the single, fully synthesized SunTag$_{24x}$-Kif18b molecules present in the same cell (Figures S2A and S2B). Several considerations need to be taken into account to calculate ribosome number from the fluorescence intensities of translation sites and fully synthesized single SunTag proteins (Supplemental Experimental Procedures). First, ribosomes present at the 5′ end of the reporter transcript have translated only a subset of the 24 SunTag peptides, so the nascent polypeptide associated with these ribosomes will have lower fluorescence intensity due to fewer bound scFv-GFPs. We generated a mathematical model to correct for the difference in fluorescence intensity for ribosomes at different positions along the transcript (Supplemental Experimental Procedures). Second, if scFv-GFP-peptide has a slow on rate for the epitope in vivo, a lag time could exist between the synthesis of a SunTag peptide and binding of a scFv-GFP, which could result in the underestimation of the number of ribosomes per mRNA. To test this, cells were treated with the translation inhibitor cycloheximide (CHX), which blocks ribosome elongation by locking ribosomes on the mRNA and prevents the synthesis of new SunTag peptides, while allowing binding of scFv-GFP to existing peptides to reach equilibrium. The translation site scFv-GFP signal did not substantially increase after CHX treatment (Figure S2C), indicating that under our experimental conditions, the lag time between peptide synthesis and scFv-GFP binding does not detectably affect translation-site intensity. Based on the above controls and our mathematical model, we could estimate the ribosome number per mRNA from the fluorescence intensity of the translation site. Approximately 30% of the mRNAs did not have a corresponding GFP signal, suggesting that they were not actively translating. For the remaining 70% of the mRNAs that were translating, the majority (76%) had between 10–25 ribosomes (Figure 2A; Supplemental Experimental Procedures), corresponding to an average inter-ribosome distance of ~200–400 nucleotides (nt). We also compared translation-site intensity of two additional reporter mRNAs with either 5× or 10× SunTag peptides with the 24× peptide reporter. This analysis revealed that ribosome density was very similar on the 5× and 10× reporter (1.26-fold and 1-fold, respectively) (Figure S2D), indicating that the long 24× SunTag array does not grossly perturb ribosome loading on the reporter mRNA.

Next, we measured the translocation speed of ribosomes on single mRNAs by treating cells with harringtonine, a small molecule inhibitor of translation that stalls new ribosomes at the start of the mRNA coding sequence without affecting ribosomes further downstream (Ingolia et al., 2011). As mRNA-bound ribosomes complete translation one-by-one after harringtonine treatment, the GFP signal on mRNAs decreases (Figures 2B–2D; Movie S4). Using a simple mathematical model to fit the decay in fluorescence of a cumulative curve from many mRNAs (Figure S7; Supplemental Experimental Procedures), we estimate a ribosome translocation rate of 3.5 $\pm$ 1.1 codons/s. In a parallel approach, we also measured the total time required for runoff of all ribosomes from individual mRNAs (Figure S2E), from which we calculated a similar translation elongation rate (3.1 $\pm$ 0.14 codons/s) as the one obtained through our model (Supplemental Experimental Procedures). A reporter with only 5 instead of 24 SunTag peptides showed similar elongation kinetics (3.1 $\pm$ 0.4 codons/s) (Figure S2F), indicating that translocation rates are likely not affected by SunTag labeling of the

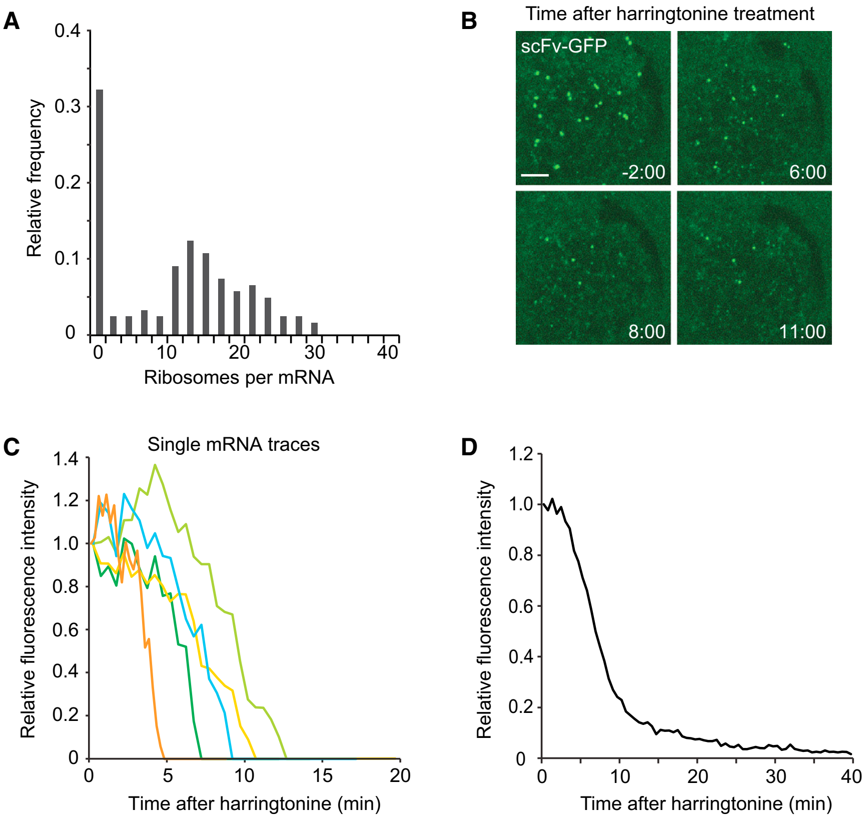

Figure 2. Measurements of Ribosome Initiation and Elongation Rates on Single mRNA Molecules

U2OS cells expressing scFv-GFP, PP7-2xmCherry-CAAX, and the translation reporter (SunTag$_{24x}$-Kif18b-PP7$_{24x}$).

(A) Distribution of the number of ribosomes bound to single mRNAs of the translation reporter (SunTag$_{24x}$-Kif18b-PP7$_{24x}$) (n = 2 independent experiments, 16 cells, 124 mRNAs), see Supplemental Experimental Procedures.

(B–D) U2OS cells expressing the translation reporter (SunTag$_{24x}$-Kif18b-PP7$_{24x}$) were treated with harringtonine at t = 0. (B) Representative images from a time-lapse movie. (C) Five representative traces of fluorescence decay on single mRNAs (of >100 analyzed). (D) Normalized quantification of the decrease in fluorescence over time from many translation sites (n = 4 independent experiments, 37 cells, 536 mRNAs). Scale bars, 5 μm.

See also Figure S2 and Movie S4.

nascent chain. Finally, we measured elongation rates of a shorter and codon-optimized reporter gene, which revealed a somewhat faster elongation rate of 4.9 codons/s (Figure S2G), indicating that elongation rates may differ on different transcripts. Using the elongation rate and ribosome density described above, we were able to estimate the translation initiation rate to be between 1.4–3.6 min^{-1} on the Kif18b reporter (Supplemental Experimental Procedures).

Together, these results provide the first in vivo measurements of the rates of ribosome initiation and translocation on single mRNA molecules in live cells.

Temporal Changes in Translation of Single mRNA Molecules

To study translation over time, we imaged cells for 2 hr and quantified the scFv-GFP signal from single mRNA molecules that could be tracked for >1 hr (Figures 3A, 3B, and S3A). The results show considerable fluctuations in the translational state of individual mRNAs over time (Figures 3A, 3B, and S3A). Such large fluctuations were not observed when cells were treated with the translation inhibitor CHX (Figure S3B), indicating they were due to changes in translation initiation and/or elongation rather than measurement noise. We also observed heterogeneity of behavior between different mRNAs. Some remained in a high translating state for >1 hr (e.g., Figures S3A12 and 13). Others shut down translation initiation and lost their scFv-GFP signal (e.g., Figures 3A, 3B, and S3A1, 3–11, and 14), which may account for the population of non-translating mRNAs observed in steady-state measurements (Figure 2A). From the progressive decline in scFv-GFP fluorescence (Figure 3C; Movie S5), we could estimate a ribosome run-off rate of 3 codons/s (Figure 3C), which is similar to that measured after addition of harringtonine (3.5 ± 1.1 codons/s) (Figure 2). Interestingly, a subset (67 of 104 mRNAs, three independent experiments, 19 cells) of these mRNAs later reinitiated translation and largely recovered their original scFv-GFP fluorescence (Figures 3A, 3B, 3D, and S3A1, 3, 5, and 8–10). Individual mRNAs even showed repeated cycling between non-translating and translating states (Figure 3A, yellow line, and S3A3, 5 and 8). Such cycles of complete translational shutdown and re-initiation occurred 0.29 ± 0.10 times per mRNA per hour (n = 4 independent experiments, 27 cells, 106 mRNAs), suggesting that most mRNAs will undergo one or more translational shutdown and re-initiation events in their lifetime. Thus, single mRNA imaging reveals reversible switching between translational shutdown and polysome formation.

After synchronized expression of the reporter construct using an inducible promoter, we often observed the initial binding events of newly transcribed mRNAs to the PP7-mCherry at the membrane (Figures 4A and 4B). Of these initial binding events, 44% of the mRNAs were associated with scFv-GFP fluorescence, indicating that they had already begun translation. However, the majority, 56% of mRNAs, initially appeared at the membrane in a non-translating state and subsequently converted to a translating state, usually within 1–5 min (Figure 4C; Movie S6). These mRNAs are likely newly transcribed mRNAs that are translating for the first time, rather than mRNAs that have already undergone translation but transitioned temporarily to a non-translating state. In support of this argument, long-term (>1 hr) imaging of single mRNAs reveals that mRNAs spend on average only 2.5% of their lifetime in such a temporary non-translating state (n = 4 independent experiments, 27 cells, 106 mRNAs), which is not sufficient to

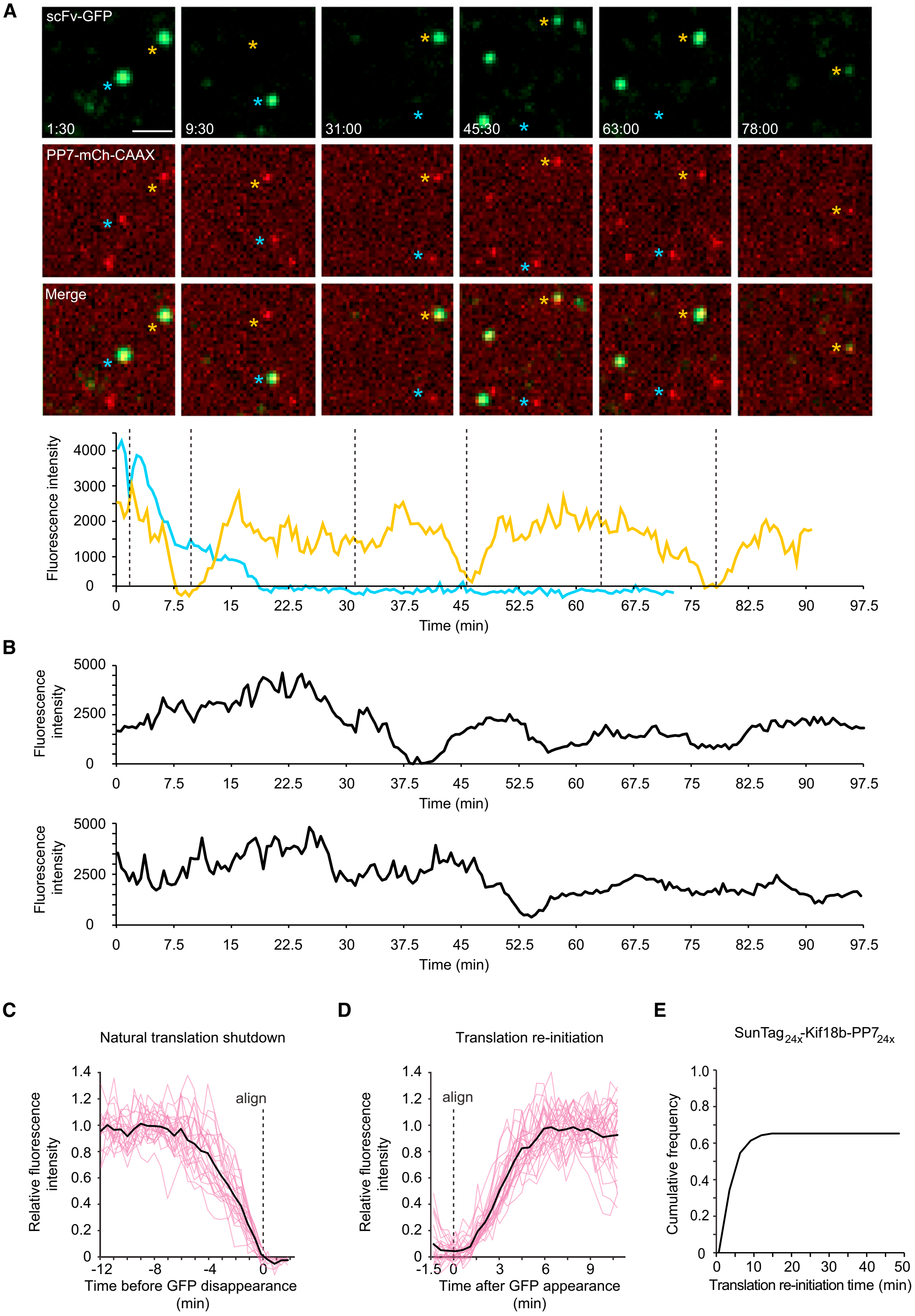

(legend on next page)

explain the 56% non-translating mRNAs that appeared at the membrane after synchronized transcription of the reporter. Rapid initiation of translation on newly transcribed mRNAs was described recently (Halstead et al., 2015), but our assay additionally allows an analysis of polysome buildup on new mRNAs (Figure 4B). Our analysis of the increase in scFv-GFP fluorescence indicates that, once the first ribosome begins chain elongation, additional ribosomes initiate translation with a rate indistinguishable from that on polysomes at steady state (Supplemental Experimental Procedures). We also examined the rate of fluorescence recovery (corresponding to polysome buildup) after complete shutdown of translation and subsequent re-initiation (Figure 4D). The polysome buildup on new transcripts was comparable to that observed for mRNAs that were cycling between translating and non-translating states (Figure 4D).

Ribosome Stalling

Several studies reported that ribosomes can pause or stall at a defined nucleic acid sequence with a regulatory function (Walter and Blobel, 1981; Yanagitani et al., 2011), at chemically modified or damaged nucleotides (Simms et al., 2014), or at regions in the RNA with a strong secondary structure (Tholstrup et al., 2012; Wen et al., 2008). We found that a subset (~5%–10%) of mRNAs retained a bright scFv-GFP signal 15 min after harringtonine treatment (Figures 2B and 2D), a time at which ribosomes translocating at ~3 codons/s should have finished translating the reporter. A similar percentage of stalled ribosomes was observed on two additional reporter transcripts, both of which were designed using optimal codon usage (Figures S2G and S4A). Ribosome stalling also was observed using hippuristanol (Figure S4B), a translation initiation inhibitor with a different mechanism of inhibition (Bordeleau et al., 2006), indicating that the stalling was not caused by harringtonine. We also observed stalls when examining ribosome runoff from non-tethered cytosolic mRNAs lacking PP7 binding sites (Figure S4C). Importantly, stalls were not observed after puromycin treatment (Figures S4D and S4E) and the prolonged (>15 min) scFv-GFP signal on mRNAs from harringtonine-treated cells rapidly disappeared upon the addition of puromycin, confirming that the observed signal indeed represents stalled ribosomes (Figure S4F). The majority of mRNAs with stalled ribosomes (33 of 43) could be tracked for >40 min, the typical duration of our harringtonine runoff experiments, indicating that they were not readily targeted by the no-go mRNA decay machinery within this time frame.

Ribosome stalls could be due to defective ribosomes causing roadblocks on the mRNA or due to defects in the mRNA. These models can potentially be distinguished by examining how such stalls are resolved. A single defective ribosome will inhibit ribosome runoff until the stalled ribosome is removed, after which, the remaining ribosomes will run off at a normal rate. In contrast, if the stalls are caused by defects to the mRNA, such as chemical damage, then each ribosome passing over the damaged nucleotide will be delayed, resulting in an overall slower scFv-GFP decay rate (Figure 5A). Long-term tracking of stalled ribosomes on single mRNAs was consistent with the latter model, indicating that ribosome stalling is likely caused by defective mRNA (Figure 5B). Consistent with the hypothesis that chemical damage to mRNA causes ribosome stalling, treatment of cells with 4-nitroquilone-1-oxide (4NQO), a potent nucleic-acid-damaging agent that causes 8-oxoguanine modifications and stalls ribosomes in vitro (Simms et al., 2014), resulted in a slow runoff on the majority of mRNAs, indicating widespread ribosome stalling (Figure 5C). Thus, chemical damage to mRNAs stalls ribosome elongation in vivo.

Regulated ribosome pausing occurs both in vitro and in vivo at asparagine 256 in the stress-related transcription factor Xbp1 (Ingolia et al., 2011; Yanagitani et al., 2011), and this ribosome pausing is important for membrane targeting of the mRNA (Yanagitani et al., 2011). To test whether our translation imaging system could recapitulate such translation pausing, we introduced a strong ribosome-pausing sequence (a point mutant of the wild-type Xbp1-pausing sequence that shows enhanced ribosome pausing [Yanagitani et al., 2011]) into the 3′ region of the coding sequence of our reporter (hereafter referred to as Xbp1 reporter). Harringtonine ribosome runoff experiments on the Xbp1 reporter revealed a delay in ribosome runoff (Figure 5D), confirming that our reporter faithfully reproduced the ribosome-pausing phenotype. To study the behavior of individual ribosomes on the Xbp1 ribosome-pausing sequence, we tracked single mRNAs during ribosome runoff. Surprisingly, the fluorescence decay was not linear, as would be expected if each ribosome paused a similar amount of time on the pause site. Rather, fluorescence decay occurred in bursts interspaced with periods in which no decay was detectable (Figures 4 and 5E, representative traces shown out of 25 analyzed). These results indicate that most ribosomes are only briefly delayed at the Xbp1 pause site, but a small subset of ribosomes remain stalled for an extended (>10 min) period of time, explaining the strong ribosome stalling phenotype observed in ensemble experiments.

Figure 3. Long-Term Dynamics of Translation of Single mRNA Molecules

U2OS cells expressing scFv-GFP, PP7-2xmCherry-CAAX, and the translation reporter (SunTag$_{24x}$-Kif18b-PP7$_{24x}$).

(A) U2OS cell expressing the SunTag$_{24x}$-Kif18b-PP7$_{24x}$ reporter was imaged by time-lapse microscopy. Blue and yellow asterisks mark two different mRNAs undergoing changes in translation over time (upper). Intensity of scFv-GFP was measured over time for the two mRNAs (lower). Colors of lines correspond to scFv-GFP intensity of translation sites marked by asterisk with the same color.

(B) ScFv-GFP intensity traces of two additional mRNA molecules.

(C) mRNAs undergoing permanent translation shutdown. Fluorescence intensity quantification is shown (n = 24 mRNAs). Average (black line) and single traces (pink lines) are shown.

(D) mRNAs undergoing translation re-activation after shutdown. Average (black line) and single traces (pink lines) are shown (n = 30 mRNAs).

(E) Time to reappearance of the first scFv-GFP fluorescence from translation sites that underwent complete translational shutdown. ~60% of the mRNAs re-initiated translation after complete shutdown and did so within 10 min (n = 104 translational sites analyzed). Scale bar, 2 μm.

See also Figure S3 and Movie S5.

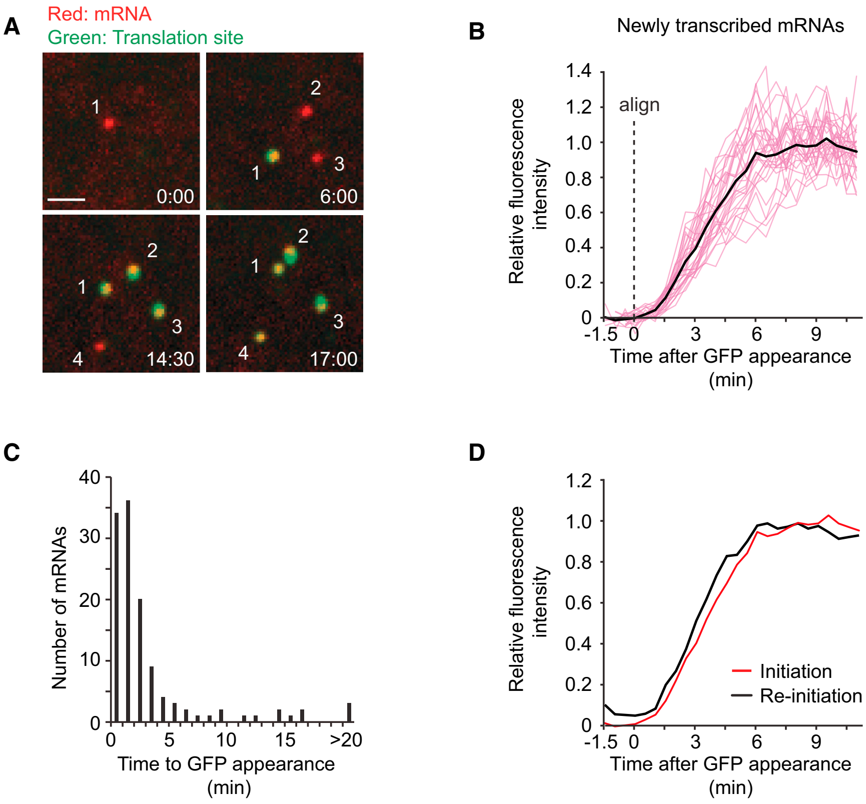

Figure 4. Analysis of Polysome Build Up on Newly Transcribed mRNAs

U2OS cells expressing scFv-GFP, PP7-2xmCherry-CAAX, and the translation reporter (SunTag$_{24x}$-Kif18b-PP7$_{24x}$).

(A) Images from a time-lapse movie of newly transcribed mRNAs undergoing the first rounds of translation.

(B) Quantification of the fluorescence intensity increase, aligned at the first time point at which scFv-GFP signal was detected (n = 30 individual mRNAs [pink lines], and average [black line] is shown).

(C) Quantification of the time between mRNA appearance and the first detection of translation by scFv-GFP fluorescence.

(D) Comparison of scFv-GFP fluorescence buildup on either new transcripts (red line) or on re-initiating mRNAs (black line). Data are re-plotted from Figures 3D and 4B. Scale bar, 2 μm.

See also Movie S6.

Translational Regulation of the Cell-Cycle Regulator Emi1

We also applied our assay to study the transcript-specific translational regulation of Emi1, a key cell-cycle regulatory protein. Our recent work reported strong translational repression of Emi1 during mitosis and found that the 3′ UTR of Emi1 is involved in this regulation (Tanenbaum et al., 2015), but a role of its 5′ UTR in translational regulation was not established. Interestingly, Emi1 has at least two splicing isoforms that differ in their 5′ UTR sequence: NM_001142522.1 (hereafter referred to as 5′ UTR_long) and NM_012177.3 (hereafter referred to as 5′ UTR_short) (Figure 6A). We found that a GFP protein fused downstream of the 5′ UTR_long was expressed at 40-fold lower levels than a GFP fused to the 5′ UTR_short (Figure 6B). Such difference in protein expression could be due to a difference in transcription rate, mRNA stability, or reduced translation initiation or elongation rates. To distinguish between these possibilities, we prepared translation reporter constructs bearing either the short or long 5′ UTR of Emi1. Robust translation was observed on ~50% of mRNAs encoding the short 5′ UTR (Figure 6C). In contrast, the majority (~80%) of transcripts encoding the Emi1 5′ UTR_long showed no detectable translation (not shown), and of the translating mRNAs, only very weak scFv-GFP fluorescence was usually detected (Figure 6C). Surprisingly, however, a very small fraction of mRNAs containing the 5′ UTR_long (~2%) was associated with a bright scFv-GFP signal (Figure 6C, >92 bin), indicating that they are bound to many ribosomes. This was not due to ribosome stalling and subsequent (slow) accumulation of ribosomes on a subset of mRNAs, as this bright scFv-GFP signal rapidly dissipated upon harringtonine treatment (Figure S5), indicating that these mRNAs were translated at high levels. Calculation of the total number of ribosomes associated with the mRNAs, based upon scFv-GFP fluorescence intensity, revealed that 52% of all ribosomes translating the Emi1 5′ UTR_long reporter were associated with the minor (2%) fraction associated with the highest scFv-GFP intensity. These results indicate that the great majority of 5′ UTR_long transcripts are strongly translationally repressed but that a small subset of these mRNAs escape repression and undergo robust translation. Thus, substantial heterogeneity in translational efficiency can exist among different mRNA molecules within the same cell.

Observation of Translation by Single Ribosomes

Interestingly, with the Emi1 5′ UTR_long reporter, we often observed the abrupt appearance of a weak scFv-GFP signal on a transcript that was previously translationally silent. The GFP signal initially increased over time, plateaued, and then was abruptly lost after 6–8 min (Figures 7A–7C; Movie S7). This type of signal is best explained by a single ribosome sequentially decoding the 24 SunTag peptides on the mRNA, followed by the release of the newly synthesized polypeptide upon completion of translation. Consistent with this hypothesis, the absolute fluorescence intensity of such translation events at the plateau phase (when all 24 SunTag peptides have been synthesized) was very similar to the intensity of a single fully synthesized SunTag24x-Kif18b protein (Figures S6A and S6B). The duration of the scFv-GFP signal per translation event could be converted to a translocation speed of single ribosomes (Supplemental Experimental Procedures), which revealed an average elongation rate of 3 codons/s (Figure 7D). This value is similar to that determined from our bulk measurements of harringtonine-induced ribosome

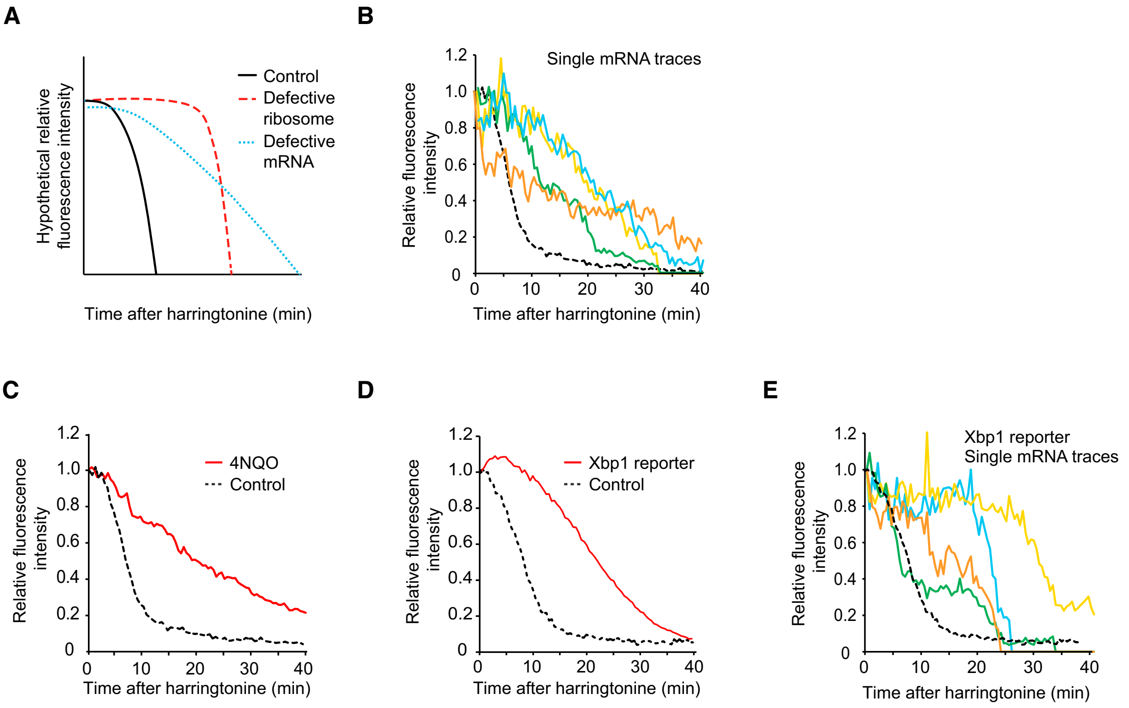

Figure 5. Dynamics of Ribosome Stalling

U2OS cells expressing scFv-GFP, PP7-2xmCherry-CAAX, and the SunTag$_{24x}$-Kif18b-PP7$_{24x}$ translation reporter (A–C) or the Xbp1 translation reporter (D–E).
(A and B) Ribosome stalling likely results from mRNA defects, model (A) and experiment (B). (B) Fluorescence intensity over time is shown for four representative stalled translation sites (colors; of 20 analyzed). Since intensity values of single mRNAs were derived from the experiments presented in Figure 2D, the average fluorescence decay presented in Figure 2D is re-plotted here for comparison (dashed black line).
(C) Nucleic acid damage through 4NQO treatment (red line) induces ribosome stalling (n = 3 independent experiments, 40 cells, 455 mRNAs). For comparison, the harringtonine runoff from control cells with the SunTag$_{24x}$-Kif18b-PP7$_{24x}$ reporter from Figure 2D is re-plotted, as these experiments were performed in parallel.
(D and E) Harringtonine runoff for the Xbp1 pause site (red line, n = 3 independent experiments, 31 cells, 990 mRNAs) (D) and control reporter (black dashed lines, n = 3 independent experiments, 27 cells, 437 mRNAs) (E).
See also Figure S4.

runoff or natural translational initiation shutdown and runoff (3–3.5 codons/s), indicating that ribosome elongation was not affected by the Emi1 5′ UTR_long. Comparison of translocation rates obtained from single ribosome translation events also revealed heterogeneity in the decoding speed of individual ribosomes in vivo (Figure 7D).

DISCUSSION

Using the SunTag system, we have developed an imaging method that measures the translation of individual mRNAs in living cells. Immobilization of mRNAs on the plasma membrane allows the long-term (>1 hr) observation of translation of single mRNA molecules, which enables analyses of translational initiation, elongation, and stalling in live cells for the first time. Under conditions of infrequent translational initiation, we can even observe a single ribosome decoding an entire mRNA molecule. Our observations reveal considerable and unexpected heterogeneity in the translation properties of different mRNA molecules derived from the same gene in a single cell, with some not translating, others actively translating with many ribosomes, and others bound to stalled ribosomes. The SunTag translation imaging assay should be applicable to many different cell types, including neurons and embryos, in which the localization and control of protein translation is thought to play an important role in cell function.

Comparison of Methods to Study Translation In Vivo

Ribosome profiling, a method in which fragments of mRNAs that are protected by the ribosome are analyzed by deep sequencing (Ingolia et al., 2009), has found widespread use in measuring translation. The strength of ribosomal profiling lies in its ability to measure translation on a genome-wide scale of endogenous mRNAs. However, a limitation of ribosome profiling is the need to pool mRNAs from many thousands of cells for a single measurement. Thus, ribosome profiling in its present form cannot be used to study translation heterogeneity between different cells in a population or among different mRNA molecules in the same cell. Furthermore, since ribosome profiling requires cell lysis, only a single measurement can be made for each sample, limiting studies of temporal changes.

A number of single-cell translation reporters have been developed based on fluorescent proteins (Aakalu et al., 2001; Brittis et al., 2002; Han et al., 2014; Raab-Graham et al., 2006; Tanenbaum et al., 2015; Tatavarty et al., 2012; Yu et al., 2006). Such reporters generally rely on the accumulation

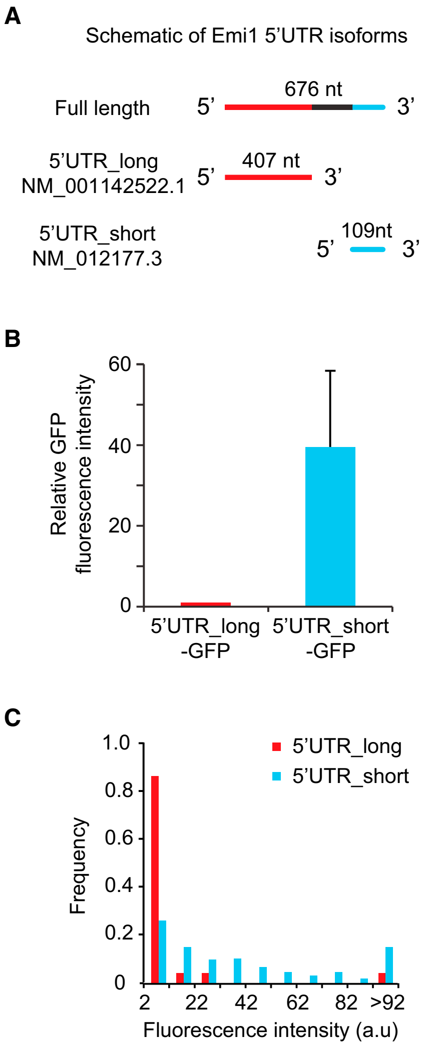

Figure 6. Differential Control of Translation Initiation by Two Emi1 Splicing Isoforms

(A) Schematic of the 5′ UTR of two Emi1 splicing isoforms.

(B) Fluorescence intensity of a GFP reporter under control of the two Emi1 isoforms (5′ UTR_long and 5′ UTR_short) expressed in HEK293 cells was measured by microscopy for single cells. Mean intensities were determined, which was corrected for background fluorescence in untransfected cells. At least 20 cells were measured per experiment per condition. Error bars, SD between experiments.

(C) Fluorescence intensity distributions of single translation sites of indicated reporters, n = 3 independent experiments, 283 mRNAs, 14 cells (5′ UTR_long) and n = 3 independent experiments, 433 mRNAs, 16 cells (5′ UTR_short). Background from adjacent regions was subtracted. Only mRNAs are plotted that had translation signal above background (with an intensity value >2; 16% and 53% of mRNAs for 5′ UTR_long and 5′ UTR_short, respectively).

See also Figure S5.

of new fluorescence after the assay is initiated. Advantages of these systems are that they are generally easy to use and have single-cell sensitivity. However, they do not provide single-mRNA resolution, often do not allow continuous measurement of translation, and do not report on ribosome initiation and elongation rates.

Finally, two methods were developed recently to image translation on single mRNAs in vivo. In one approach, the first round of translation is visualized (Halstead et al., 2015). This method, however, does not allow continuous measurements of translation. The second approach involves measurements of the number of ribosomes bound to an mRNA using fluorescence fluctuation spectroscopy (Wu et al., 2015). The advantage of this method is that it can detect binding of a single fluorescent protein to an mRNA and different subcellular sites can be probed to study spatial differences in translation. The limitation of this method though is the inability to follow translation of single mRNAs over time, as these mRNAs cannot be tracked in the cell.

SunTag-based translation imaging assays are unique thus far in their ability to follow translation of individual mRNAs over time. This translation assay can be employed with either freely diffusing or tethered mRNAs, the choice of which will depend on the biological question to be addressed. In the study by Wang et al. (2016) [this issue of *Cell*], translation is observed in distinct spatial compartments in neurons using a similar SunTag-based translation imaging method with non-tethered mRNAs. In contrast, for studying ribosome translocation dynamics, the tethering assay provides the ability to track a single mRNA throughout the duration of the ribosome elongation cycle. Using this assay, we could measure polysome buildup rates over time, observe mRNAs cycling between translating and non-translating states, uncover heterogeneity in translation initiation rates (e.g., with the Emi1 5′ UTR) and even observe a single ribosome translating an entire transcript. These measurements were aided by the vastly improved signal-to-noise of the tethered assay and the ability to easily track slowly diffusing tethered mRNAs for an hour or more. These long-term observations allowed us to discover that mRNAs can reversibly switch between a translating and non-translating state and have a high variability in pause duration at the Xbp1 site. Thus, the untethered and tethered SunTag assays provide means to study translation of single mRNA molecules, which will be applicable to a wide variety of biological questions and will be complementary to existing methods of studying translation.

A drawback of our assay is the need to insert an array of SunTag peptide repeats into the mRNA of interest to fluorescently label the nascent polypeptide and the need to insert an array of PP7 binding sites in the 3′ UTR to label the mRNA. As is true of any tagging strategy, these modifications could interfere with translation and/or mRNA stability under certain conditions. We have performed a number of control experiments to ensure that binding the scFv-GFP to the nascent chain and tethering of the transcript to the membrane do not grossly perturb translation (Figures 1B and 1F). We have also shown that ribosome translocation rates and ribosome density are similar when using a reporter with a very short (5×) or long (24×) SunTag peptide array and comparing tethered and non-tethered mRNAs (Figures 2D, S2F, and S4C), indicating that many aspects of translation are not perturbed in our assay. Nevertheless, tethering of certain mRNAs to the plasma membrane may influence translation, especially for those mRNAs that undergo local translation in a specific compartment of the cell. Thus, our assay has unique advantages for certain types of measurements of

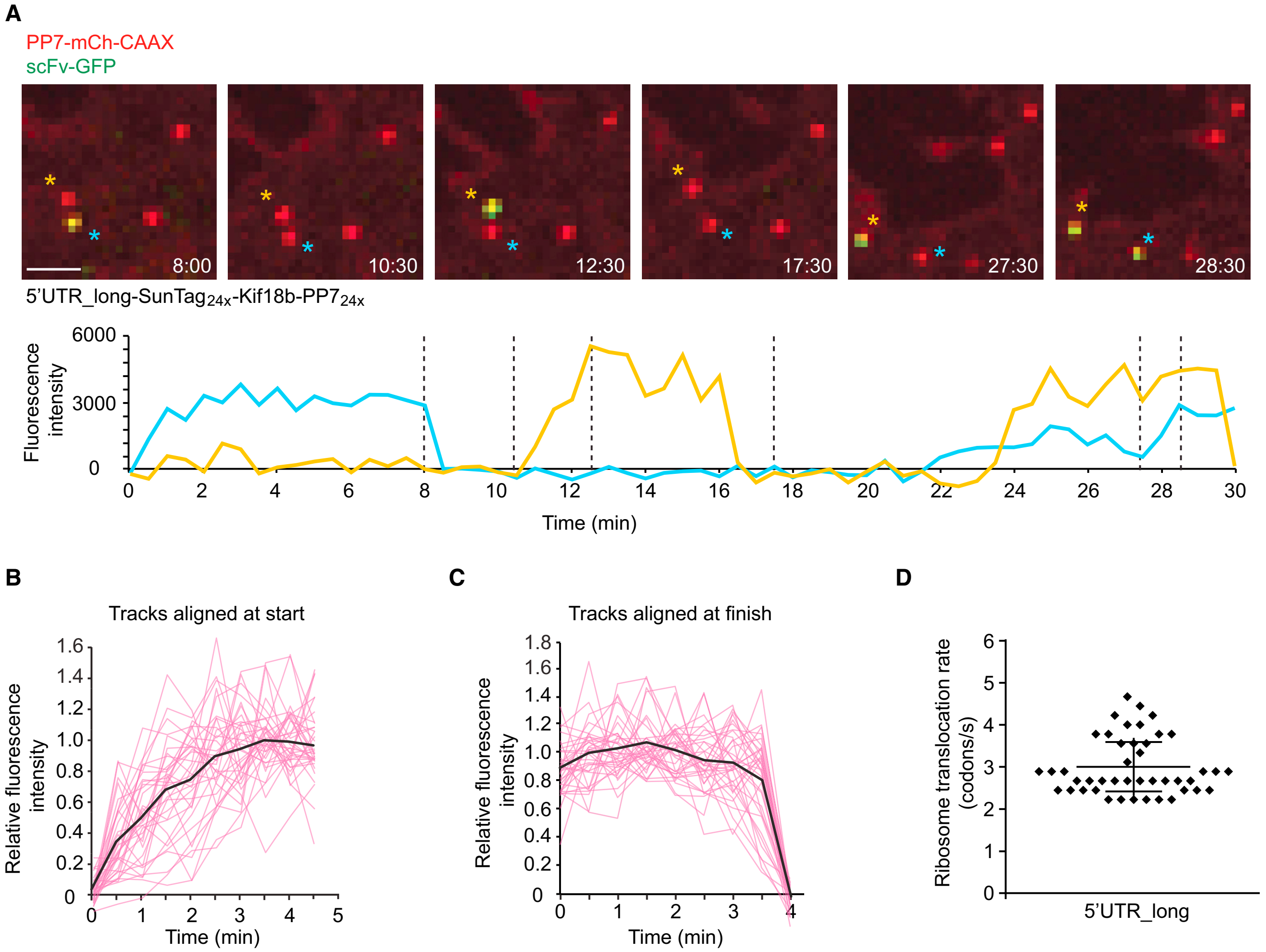

Figure 7. Visualizing Single Ribosomes Decoding an mRNA Molecule

(A–D) Analysis of single ribosomes on the Emi1 5′UTR_long reporter mRNA. (A) Representative images of multiple single ribosome translation events of individual mRNAs (upper). ScFv-GFP intensity was quantified over time for the two mRNAs marked by asterisks with the same color (lower). (B) Increase in scFv-GFP fluorescence from single ribosome translation events aligned at the first detectable scFv-GFP signal (n = 35 individual mRNAs in pink and average in black). (C) Steady-state and then abrupt decrease in scFv-GFP fluorescence from single translating ribosomes (n = 35 individual mRNAs [pink] and average [black]). (D) Single ribosome elongation rates (n = 44) (Supplemental Experimental Procedures). Mean ± SD is shown in (D). Scale bar, 2 μm.

See also Figure S6 and Movie S7.

translation, but appropriate controls should be performed for each experimental system or objective.

Heterogeneity in Translation of Single mRNAs: Possible Molecular Mechanisms

Using our system, we measured the ribosome translocation speed on single mRNA molecules. Ribosome translocation rates have been measured in bulk previously in mouse embryonic stem cells (Ingolia et al., 2011), which yielded a translocation rate of 5.6 codons/s. Our values of 3–5 codons/s (Figure S7; Supplemental Experimental Procedures) are in general agreement with those published values and very similar to those measured by Wang et al. (2016) (4 codons/s). Our experiments, and those of Wang et al. (2016), are the first to measure ribosome translocation rates for a single mRNA species, in single cells and on single mRNAs, which provides new opportunities to study regulation of translation elongation.

We also found that translation initiation can shut down temporarily on individual mRNAs and rapidly restart (Figure 3). Such shutdown of translation initiation could be due to transient loss of eIF4E binding to the mRNA cap, mRNA decapping followed by recapping (Mukherjee et al., 2012), or transient binding of regulatory proteins. Using our mRNA tethering assay, binding and unbinding of single proteins to translating mRNA could potentially be observed using total internal reflection fluorescence (TIRF), which could open up many additional possibilities for studying translational regulation at the single-molecule level.

The pioneer round of translation, the first ribosome to initiate translation on a newly transcribed mRNA, may be especially important, as it is thought to detect defects in the mRNA,

including premature stop codons (Ishigaki et al., 2001). A recently developed translation biosensor can detect the location of this pioneer round of translation (Halstead et al., 2015). However, what happens after the first ribosome initiates translation is unknown. We found that the translation initiation rate on our reporter mRNA was similar on newly transcribed, recently shut down, and re-initiating mRNAs and polysomal mRNAs (Figure 4; Supplemental Experimental Procedures), indicating that the initiation rate is independent of the number of ribosomes bound to the mRNA. The presence of introns in a gene may also affect translation initiation on newly transcribed mRNAs (Le Hir et al., 2016), which could be tested in future studies.

A subset of ribosomes stall on mRNAs in a sequence-independent fashion (Figures 2D, S2G, and S4A). One possible explanation for this is that ribosome stalling is caused by naturally occurring mRNA "damage" (i.e., chemical modifications of the nucleotides). Previous studies have found that the 8-oxoguanine modification occurs on mRNA in vivo, and such modifications cause ribosome stalling in vitro (Simms et al., 2014) and in vivo (Figure 5C). Alternatively, while we have performed numerous control experiments (Figures 5 and S4), we cannot completely exclude that the observed stalling on a small subset of mRNAs is an artifact of our construct or assay. We also observe ribosome pausing in a sequence-dependent fashion on the pause site of the Xbp1 transcription factor. Such pausing had been observed previously in bulk measurements (Ingolia et al., 2011; Yanagitani et al., 2011), but our quantitative analysis of single mRNAs revealed a high degree of variability in ribosome pausing at this site.

Finally, we show that the 5′ UTR sequence of one Emi1 transcript isoform severely inhibits translation initiation. A likely explanation for this effect is the presence of several upstream open reading frames (uORFs) in this sequence. Surprisingly, a small number of mRNA molecules encoding this 5′ UTR do undergo high levels of translation. It is possible that highly translating mRNAs are generated through alternative downstream transcription start site selection, which generates an mRNA that lacks the repressive sequence (for example, the uORFs). Alternatively, translation could occur if the 5′ UTR repressive sequence is cleaved off, followed by recapping after transcription, if a repressive protein factor dissociates, or if an inhibitory RNA secondary structure unfolds. Further studies will be required to distinguish between these possibilities.

In summary, here we have developed an imaging method that enables the measurement of ribosome initiation and translocation rates on single mRNA molecules in live cells. Future developments of this technology could include simultaneous observation of single translation factors or other regulatory molecules together with mRNAs and nascent polypeptides, which would provide a very powerful system to dissect the molecular mechanisms of translational control.

EXPERIMENTAL PROCEDURES

Cell Culture and Drug Treatment

U2OS and HEK293 cells were grown in DMEM/5% with Pen/Strep. Plasmid transfections were performed with Fugene 6 (Roche), and stable transformants were selected with zeocin (Life Technologies). Unless noted otherwise, reporter transcripts were expressed from a doxycycline-inducible promoter, and expression of the reporter was induced with 1 μg/mL doxycycline (Sigma) for 1 hr before imaging. Harringtonine (Cayman Chemical) was used at 3 μg/mL. 5 μM 4NQO (Sigma) was added to cells for 1 hr before imaging. Puromycin (Life Technologies) was used at 100 μg/mL. Hippuristanol (a kind gift of Dr. J. Tanaka) was used at 5 μM. Cycloheximide (Sigma) was used at 200 μg/mL.

Plasmid Sequences

Sequences of constructs used in this study are provided in the Supplemental Experimental Procedures.

Microscopy

Cells were grown in 96-well glass bottom dishes (Matriplate, Brooks). Images were acquired using a Yokogawa CSU-X1 spinning disk confocal attached to an inverted Nikon TI microscope with Nikon Perfect Focus system, 100× NA 1.49 objective, an Andor iXon Ultra 897 EM-CCD camera, and Micro-Manager software (Edelstein et al., 2010). Single z-plane images were acquired every 30 s unless noted otherwise. During image acquisition, cells were maintained at a constant temperature of 36°C–37°C. Camera exposure times were generally set to 500 ms, unless noted otherwise. We note that stable expression of PP7-mCherry, either with or without the CAAX domain, also resulted in an accumulation of mCherry signal in lysosomes, but lysosomes could be readily distinguished from mRNA foci based on signal intensity and mobility.

FACS

GFP and scFv-GFP (Figure 1B), mCherry, PP7-mCherry, or PP7-2xmCherry-CAAX (Figure 1F) were expressed from a constitutive promoter, while the two reporters, SunTag$_{24x}$-mCherry and GFP-PP7$_{24x}$ (Figures 1B and 1F, respectively) were expressed from an inducible promoter in U2OS cells expressing the Tet repressor protein, and their expression was induced 24 hr after transfection using doxycycline (1 μg/mL). This ensured that the reporters were translated in the presence of high levels of the scFv-GFP and PP7-2xmCherry-CAAX proteins. Cells were collected one day after doxycycline induction and analyzed by FACS. Cells were gated for GFP and mCherry double positivity, and the mCherry and GFP levels (Figures 1B and 1F, respectively) were analyzed using Flowjo v10.1.

Image Analysis and Quantification

For detailed description of Image analysis and quantification, see Supplemental Experimental Procedures.

SUPPLEMENTAL INFORMATION

Supplemental Information includes Supplemental Experimental Procedures, seven figures, and seven movies and can be found with this article online at http://dx.doi.org/10.1016/j.cell.2016.04.034.

AUTHOR CONTRIBUTIONS

M.E.T. conceived of the project with input from R.D.V.; X.Y., T.A.H., and M.E.T. performed the experiments and analyzed the data. All authors interpreted the results. X.Y. developed the mathematical model. X.Y., M.E.T., and R.D.V. wrote the manuscript with input from T.A.H.

ACKNOWLEDGMENTS

We would like to thank Nico Stuurman for developing the spot_counter plugin for imageJ and for general help with microscopy, Dr. J. Tanaka for providing hippuristanol, and S. Ruijtenberg and L. Krenning for helpful discussions. This work was supported by a European Research Council (ERC) starting grant (ERC-STG 677936-RNAREG). M.E.T. was supported by a fellowship of the Dutch Cancer Society (KWF), and R.D.V. was supported by the Howard Hughes Medical Institute. A patent has been filed for the SunTag technology (M.E.T. and R.D.V.).

Received: March 18, 2016
Revised: April 4, 2016
Accepted: April 11, 2016
Published: May 5, 2016

REFERENCES

Aakalu, G., Smith, W.B., Nguyen, N., Jiang, C., and Schuman, E.M. (2001). Dynamic visualization of local protein synthesis in hippocampal neurons. Neuron *30*, 489–502.

Babendure, J.R., Babendure, J.L., Ding, J.H., and Tsien, R.Y. (2006). Control of mammalian translation by mRNA structure near caps. RNA *12*, 851–861.

Blanchard, S.C. (2009). Single-molecule observations of ribosome function. Curr. Opin. Struct. Biol. *19*, 103–109.

Bordeleau, M.E., Mori, A., Oberer, M., Lindqvist, L., Chard, L.S., Higa, T., Belsham, G.J., Wagner, G., Tanaka, J., and Pelletier, J. (2006). Functional characterization of IRESes by an inhibitor of the RNA helicase eIF4A. Nat. Chem. Biol. *2*, 213–220.

Brar, G.A., Yassour, M., Friedman, N., Regev, A., Ingolia, N.T., and Weissman, J.S. (2012). High-resolution view of the yeast meiotic program revealed by ribosome profiling. Science *335*, 552–557.

Brittis, P.A., Lu, Q., and Flanagan, J.G. (2002). Axonal protein synthesis provides a mechanism for localized regulation at an intermediate target. Cell *110*, 223–235.

Chao, J.A., Patskovsky, Y., Almo, S.C., and Singer, R.H. (2008). Structural basis for the coevolution of a viral RNA-protein complex. Nat. Struct. Mol. Biol. *15*, 103–105.

Chen, J., Tsai, A., O'Leary, S.E., Petrov, A., and Puglisi, J.D. (2012). Unraveling the dynamics of ribosome translocation. Curr. Opin. Struct. Biol. *22*, 804–814.

Choi, J., Ieong, K.W., Demirci, H., Chen, J., Petrov, A., Prabhakar, A., O'Leary, S.E., Dominissini, D., Rechavi, G., Soltis, S.M., et al. (2016). N(6)-methyladenosine in mRNA disrupts tRNA selection and translation-elongation dynamics. Nat. Struct. Mol. Biol. *23*, 110–115.

Cornish, P.V., Ermolenko, D.N., Noller, H.F., and Ha, T. (2008). Spontaneous intersubunit rotation in single ribosomes. Mol. Cell *30*, 578–588.

Edelstein, A., Amodaj, N., Hoover, K., Vale, R., and Stuurman, N. (2010). Computer Control of Microscopes using μManager. In Current Protocols in Molecular Biology, F.M. Ausubel, ed. (John Wiley & Sons), pp. 14.20.1–14.20.17.

Elkon, R., Ugalde, A.P., and Agami, R. (2013). Alternative cleavage and polyadenylation: extent, regulation and function. Nat. Rev. Genet. *14*, 496–506.

Fei, J., Kosuri, P., MacDougall, D.D., and Gonzalez, R.L., Jr. (2008). Coupling of ribosomal L1 stalk and tRNA dynamics during translation elongation. Mol. Cell *30*, 348–359.

Gupta, I., Clauder-Münster, S., Klaus, B., Järvelin, A.I., Aiyar, R.S., Benes, V., Wilkening, S., Huber, W., Pelechano, V., and Steinmetz, L.M. (2014). Alternative polyadenylation diversifies post-transcriptional regulation by selective RNA-protein interactions. Mol. Syst. Biol. *10*, 719.

Halstead, J.M., Lionnet, T., Wilbertz, J.H., Wippich, F., Ephrussi, A., Singer, R.H., and Chao, J.A. (2015). Translation. An RNA biosensor for imaging the first round of translation from single cells to living animals. Science *347*, 1367–1671.

Han, K., Jaimovich, A., Dey, G., Ruggero, D., Meyuhas, O., Sonenberg, N., and Meyer, T. (2014). Parallel measurement of dynamic changes in translation rates in single cells. Nat. Methods *11*, 86–93.

Holt, C.E., and Schuman, E.M. (2013). The central dogma decentralized: new perspectives on RNA function and local translation in neurons. Neuron *80*, 648–657.

Hüttelmaier, S., Zenklusen, D., Lederer, M., Dictenberg, J., Lorenz, M., Meng, X., Bassell, G.J., Condeelis, J., and Singer, R.H. (2005). Spatial regulation of beta-actin translation by Src-dependent phosphorylation of ZBP1. Nature *438*, 512–515.

Ingolia, N.T., Ghaemmaghami, S., Newman, J.R., and Weissman, J.S. (2009). Genome-wide analysis in vivo of translation with nucleotide resolution using ribosome profiling. Science *324*, 218–223.

Ingolia, N.T., Lareau, L.F., and Weissman, J.S. (2011). Ribosome profiling of mouse embryonic stem cells reveals the complexity and dynamics of mammalian proteomes. Cell *147*, 789–802.

Ishigaki, Y., Li, X., Serin, G., and Maquat, L.E. (2001). Evidence for a pioneer round of mRNA translation: mRNAs subject to nonsense-mediated decay in mammalian cells are bound by CBP80 and CBP20. Cell *106*, 607–617.

Jovanovic, M., Rooney, M.S., Mertins, P., Przybylski, D., Chevrier, N., Satija, R., Rodriguez, E.H., Fields, A.P., Schwartz, S., Raychowdhury, R., et al. (2015). Immunogenetics. Dynamic profiling of the protein life cycle in response to pathogens. Science *347*, 1259038.

Katz, Z.B., English, B.P., Lionnet, T., Yoon, Y.J., Monnier, N., Ovryn, B., Bathe, M., and Singer, R.H. (2016). Mapping translation 'hot-spots' in live cells by tracking single molecules of mRNA and ribosomes. eLife *5*, e10415.

Kertesz, M., Wan, Y., Mazor, E., Rinn, J.L., Nutter, R.C., Chang, H.Y., and Segal, E. (2010). Genome-wide measurement of RNA secondary structure in yeast. Nature *467*, 103–107.

Le Hir, H., Saulière, J., and Wang, Z. (2016). The exon junction complex as a node of post-transcriptional networks. Nat. Rev. Mol. Cell Biol. *17*, 41–54.

Leung, K.M., van Horck, F.P., Lin, A.C., Allison, R., Standart, N., and Holt, C.E. (2006). Asymmetrical beta-actin mRNA translation in growth cones mediates attractive turning to netrin-1. Nat. Neurosci. *9*, 1247–1256.

Mukherjee, C., Patil, D.P., Kennedy, B.A., Bakthavachalu, B., Bundschuh, R., and Schoenberg, D.R. (2012). Identification of cytoplasmic capping targets reveals a role for cap homeostasis in translation and mRNA stability. Cell Rep. *2*, 674–684.

Peshkin, L., Wühr, M., Pearl, E., Haas, W., Freeman, R.M., Jr., Gerhart, J.C., Klein, A.M., Horb, M., Gygi, S.P., and Kirschner, M.W. (2015). On the Relationship of Protein and mRNA Dynamics in Vertebrate Embryonic Development. Dev. Cell *35*, 383–394.

Raab-Graham, K.F., Haddick, P.C., Jan, Y.N., and Jan, L.Y. (2006). Activity- and mTOR-dependent suppression of Kv1.1 channel mRNA translation in dendrites. Science *314*, 144–148.

Rodriguez, A.J., Shenoy, S.M., Singer, R.H., and Condeelis, J. (2006). Visualization of mRNA translation in living cells. J. Cell Biol. *175*, 67–76.

Rojas-Duran, M.F., and Gilbert, W.V. (2012). Alternative transcription start site selection leads to large differences in translation activity in yeast. RNA *18*, 2299–2305.

Schwanhäusser, B., Busse, D., Li, N., Dittmar, G., Schuchhardt, J., Wolf, J., Chen, W., and Selbach, M. (2011). Global quantification of mammalian gene expression control. Nature *473*, 337–342.

Simms, C.L., Hudson, B.H., Mosior, J.W., Rangwala, A.S., and Zaher, H.S. (2014). An active role for the ribosome in determining the fate of oxidized mRNA. Cell Rep. *9*, 1256–1264.

Stumpf, C.R., Moreno, M.V., Olshen, A.B., Taylor, B.S., and Ruggero, D. (2013). The translational landscape of the mammalian cell cycle. Mol. Cell *52*, 574–582.

Tanenbaum, M.E., Macurek, L., van der Vaart, B., Galli, M., Akhmanova, A., and Medema, R.H. (2011). A complex of Kif18b and MCAK promotes microtubule depolymerization and is negatively regulated by Aurora kinases. Curr. Biol. *21*, 1356–1365.

Tanenbaum, M.E., Gilbert, L.A., Qi, L.S., Weissman, J.S., and Vale, R.D. (2014). A protein-tagging system for signal amplification in gene expression and fluorescence imaging. Cell *159*, 635–646.

Tanenbaum, M.E., Stern-Ginossar, N., Weissman, J.S., and Vale, R.D. (2015). Regulation of mRNA translation during mitosis. eLife *4*.

Tatavarty, V., Ifrim, M.F., Levin, M., Korza, G., Barbarese, E., Yu, J., and Carson, J.H. (2012). Single-molecule imaging of translational output from individual RNA granules in neurons. Mol. Biol. Cell *23*, 918–929.

Tholstrup, J., Oddershede, L.B., and Sørensen, M.A. (2012). mRNA pseudoknot structures can act as ribosomal roadblocks. Nucleic Acids Res. *40*, 303–313.

Vardy, L., and Orr-Weaver, T.L. (2007). Regulating translation of maternal messages: multiple repression mechanisms. Trends Cell Biol. *17*, 547–554.

Walter, P., and Blobel, G. (1981). Translocation of proteins across the endoplasmic reticulum III. Signal recognition protein (SRP) causes signal sequence-dependent and site-specific arrest of chain elongation that is released by microsomal membranes. J. Cell Biol. *91*, 557–561.

Wang, X., Zhao, B.S., Roundtree, I.A., Lu, Z., Han, D., Ma, H., Weng, X., Chen, K., Shi, H., and He, C. (2015). N(6)-methyladenosine Modulates Messenger RNA Translation Efficiency. Cell *161*, 1388–1399.

Wang, C., Han, B., Zhou, R., and Zhuang, X. (2016). Real-time imaging of translation on single mRNA transcripts in live cells. Cell *165*, this issue, 990–1001.

Wen, J.D., Lancaster, L., Hodges, C., Zeri, A.C., Yoshimura, S.H., Noller, H.F., Bustamante, C., and Tinoco, I. (2008). Following translation by single ribosomes one codon at a time. Nature *452*, 598–603.

Wu, B., Buxbaum, A.R., Katz, Z.B., Yoon, Y.J., and Singer, R.H. (2015). Quantifying Protein-mRNA Interactions in Single Live Cells. Cell *162*, 211–220.

Yanagitani, K., Kimata, Y., Kadokura, H., and Kohno, K. (2011). Translational pausing ensures membrane targeting and cytoplasmic splicing of XBP1u mRNA. Science *331*, 586–589.

Yu, J., Xiao, J., Ren, X., Lao, K., and Xie, X.S. (2006). Probing gene expression in live cells, one protein molecule at a time. Science *311*, 1600–1603.

Zaher, H.S., and Green, R. (2009). Quality control by the ribosome following peptide bond formation. Nature *457*, 161–166.

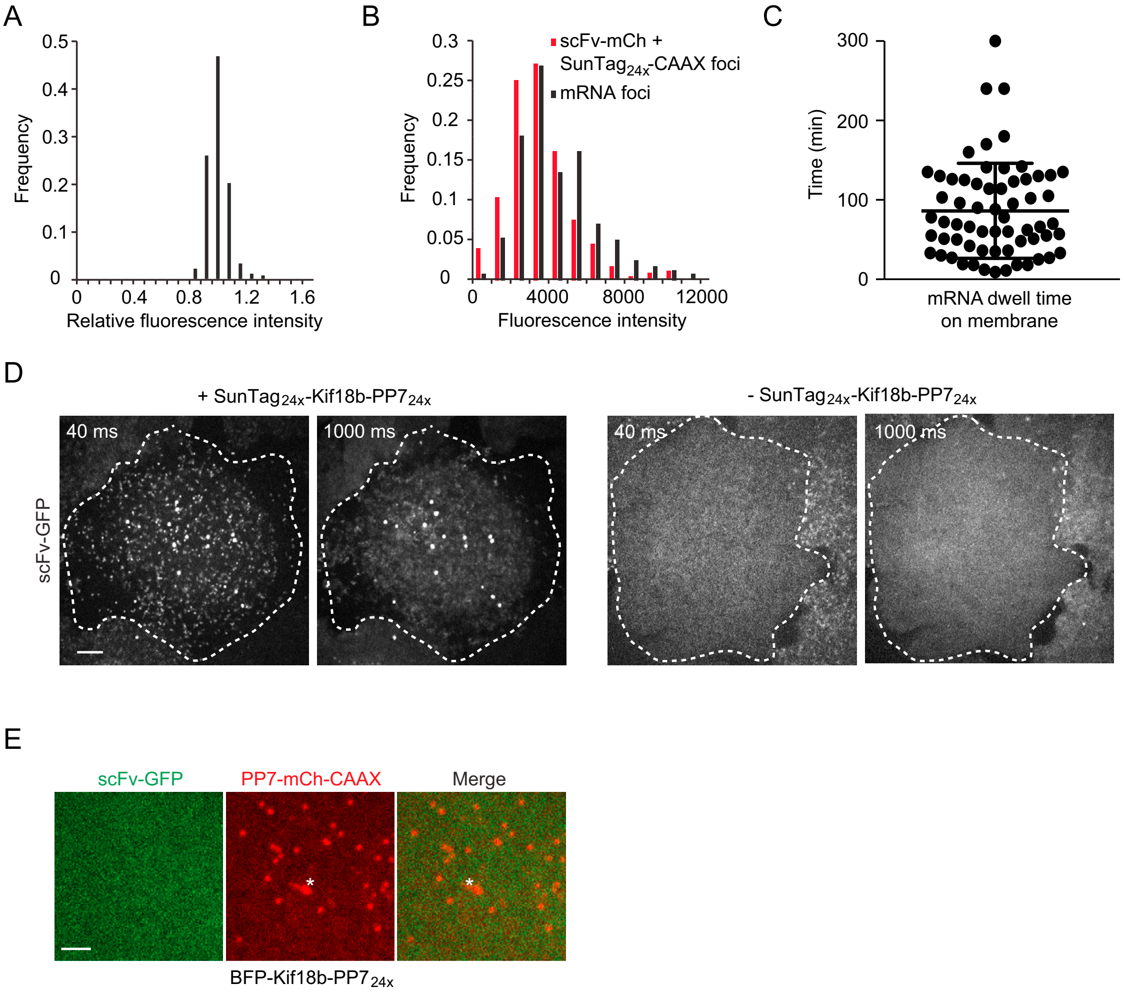

Figure S1. Validation of Single Molecule Translation Visualization Assay, Related to Figure 1

(A–C) U2OS cells expressing scFv-GFP, PP7-2xmCherry-CAAX and the $SunTag_{24x}$-Kif18b-$PP7_{24x}$ translation reporter. (A) Fluorescence intensity of mRNA foci was measured and was corrected for background fluorescence. The average corrected mRNA fluorescence intensity was set to 1 for each separate cell (n = 3 experiments, 14 cells, 278 mRNAs). (B) Intensity of single mRNA foci was measured and corrected for background, but intensity was not normalized as in (A) to allow comparison of absolute intensities (Black bars, n = 3 independent experiments, 22 cells, 377 mRNAs). In parallel, U2OS cells co-expressing $SunTag_{24x}$–CAAX and scFv-mCherry were imaged and the intensities of single membrane bound scFv-mCherry-$SunTag_{24x}$ foci was measured (Red bars, n = 4 independent experiments, 24 cells, 162 mRNAs). (C) Dwell time of tethered mRNAs on the membrane. The time between mRNA appearance at the focal plane of the membrane and its disappearance was scored. mRNA disappearance was due to mRNA detachment or degradation, not photobleaching. Mean and SD are indicated.

(D) Cells expressing scFv-GFP with (left two images) or without (right two images) the $SunTag_{24x}$-Kif18b-$PP7_{24x}$ reporter were imaged with indicated exposure time. Dotted line shows outline of the cell.

(E) U2OS cells expressing scFv-GFP, PP7-2xmCherry-CAAX and the BFP-Kif18b-$PP7_{24x}$ translation reporter. Representative image is shown. Asterisk indicates lysosome.

Scale bars are 5 μm (D) and 2 μm (E).

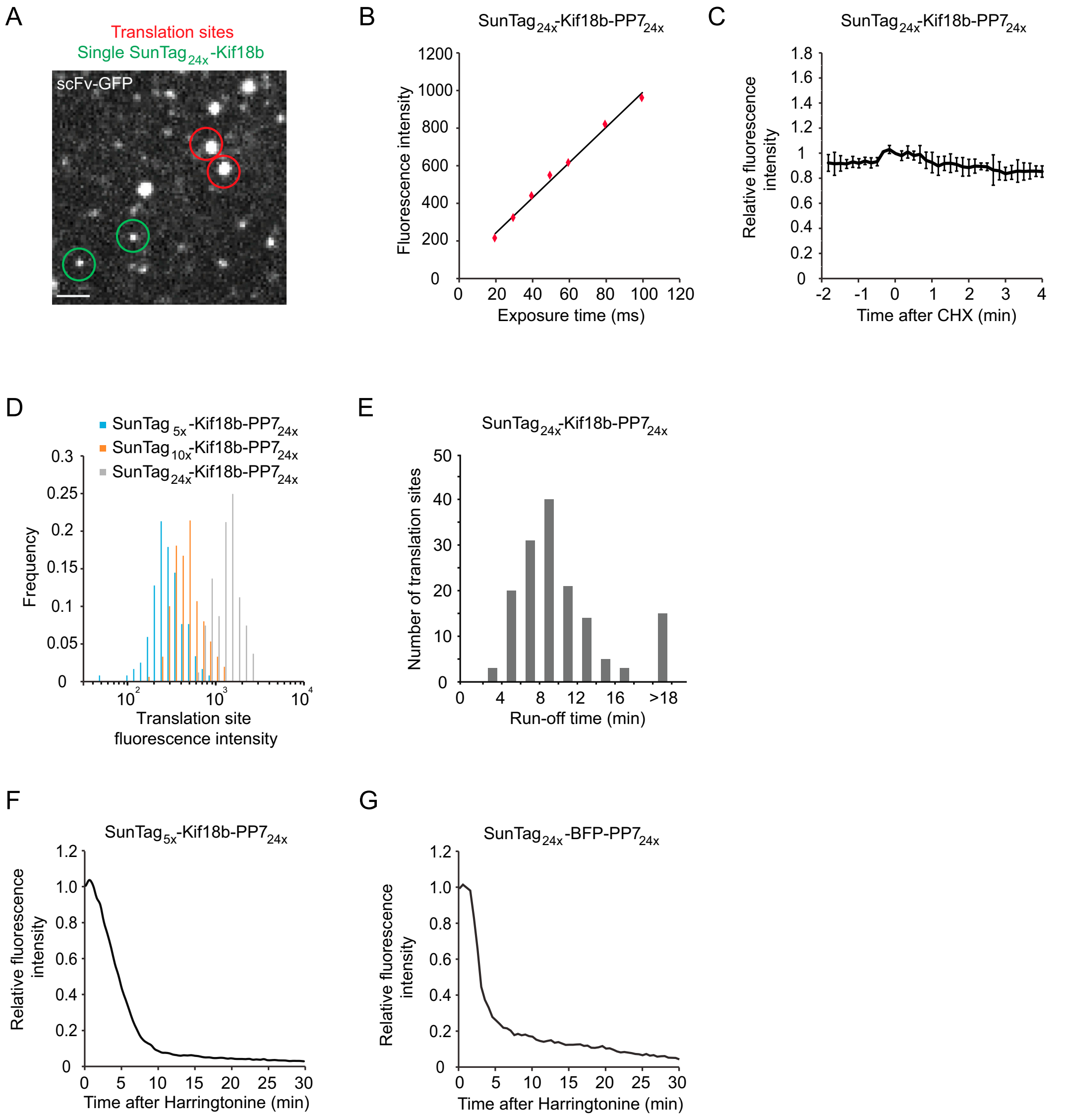

Figure S2. Quantification of Ribosome Number and Elongation Speed on Single mRNAs, Related to Figure 2

U2OS cells expressing scFv-GFP, PP7-2xmCherry-CAAX and indicated translation reporters.

(A) Images were acquired using short exposure times (40 ms), limiting motion blurring of fast moving particles, so both translation sites (red circle) and single, fully synthesized, freely diffusing SunTag proteins (green circles) could be observed as distinct foci. Fluorescence intensity of single $SunTag_{24x}$-Kif18b foci and single translation site was quantified in the same cell using a ROI with fixed size. (n = 45 translation sites, 15 cells, 3 experiments).

(B) To determine whether the exposure time of 40 ms used in (A) was sufficiently short to prevent a reduction in fluorescence intensity of foci due to motion blurring, we measured the intensity of single fully synthesized $SunTag_{24x}$-Kif18b foci at different exposure times. Fluorescence intensities of the ~25 brightest foci per image were measured. Results show a linear relationship between exposure time and fluorescence intensity at short exposure times, indicating that exposure times where short enough to prevent reduction in fluorescence intensity of foci due to motion blurring (n = 3 independent experiments, 18 cells and 400-500 spots).

(C) Cells were treated with 200 μg/mL CHX at t = 0 and fluorescence intensities of translation sites were measured over time. Note that fluorescence does not increase upon CHX treatment (n = 3 independent experiments, 31 cells, 209 mRNAs). Error bars indicate SD.

(D) scFv-GFP fluorescence intensity of translation sites using reporters with varying numbers of SunTag peptides (5×, 10× and 24×). (n = 117, 149, 80 translation sites for the 5×, 10× and 24× reporters, respectively).

(E–G) Cells were treated with harringtonine at t = 0 and translation-site intensity was quantified over time. (E) Histogram of the total run-off time, measured from the time of harringtonine treatment to the final disappearance of the scFv-GFP signal. 60 s was subtracted from all times to correct for the time required for harringtonine to enter the cell. (F and G) ScFv-GFP fluorescence intensity was measured over time after harringtonine addition (F, n = 3 independent experiments, 39 cells, 1883 mRNAs) (G, n = 3 independent experiments, 30 cells, 378 mRNAs).

Scale bar, 2 μm.

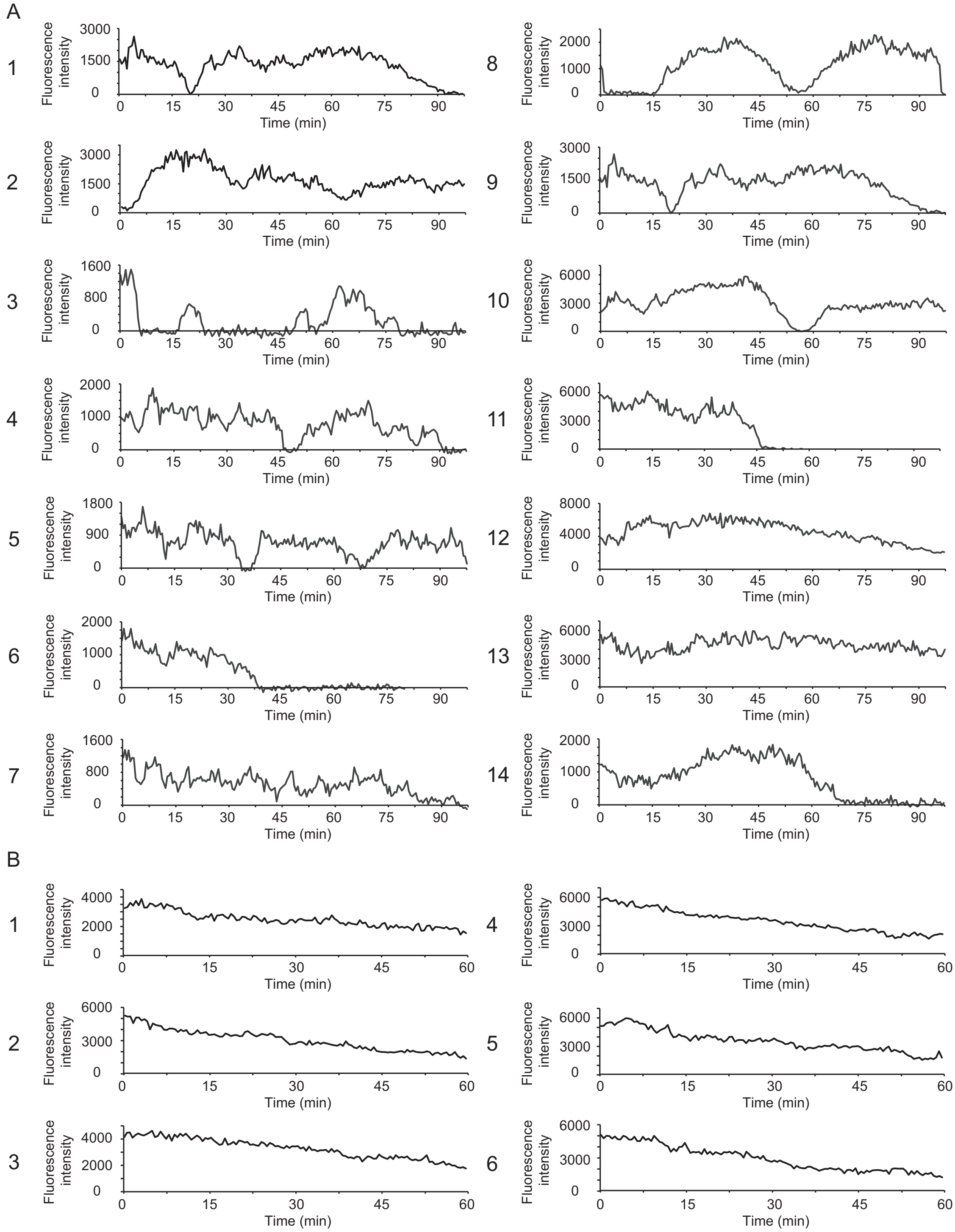

Figure S3. Translation Dynamics of Single mRNA Molecules, Related to Figure 3

U2OS cells expressing scFv-GFP, PP7-2xmCherry-CAAX and the translation reporter (SunTag$_{24x}$-Kif18b-PP7$_{24x}$) were imaged by time-lapse microscopy for 2 hr (A) or 1 hr (B) and the fluorescence intensity of single translation sites was tracked over time. 14 traces of untreated cells (A) or 6 traces of CHX treated cells (B) are shown. Note that the intensity of translation sites in CHX-treated cells slowly decreases over time, which is likely due to a decrease in the ribosome number per mRNA after prolonged CHX treatment.

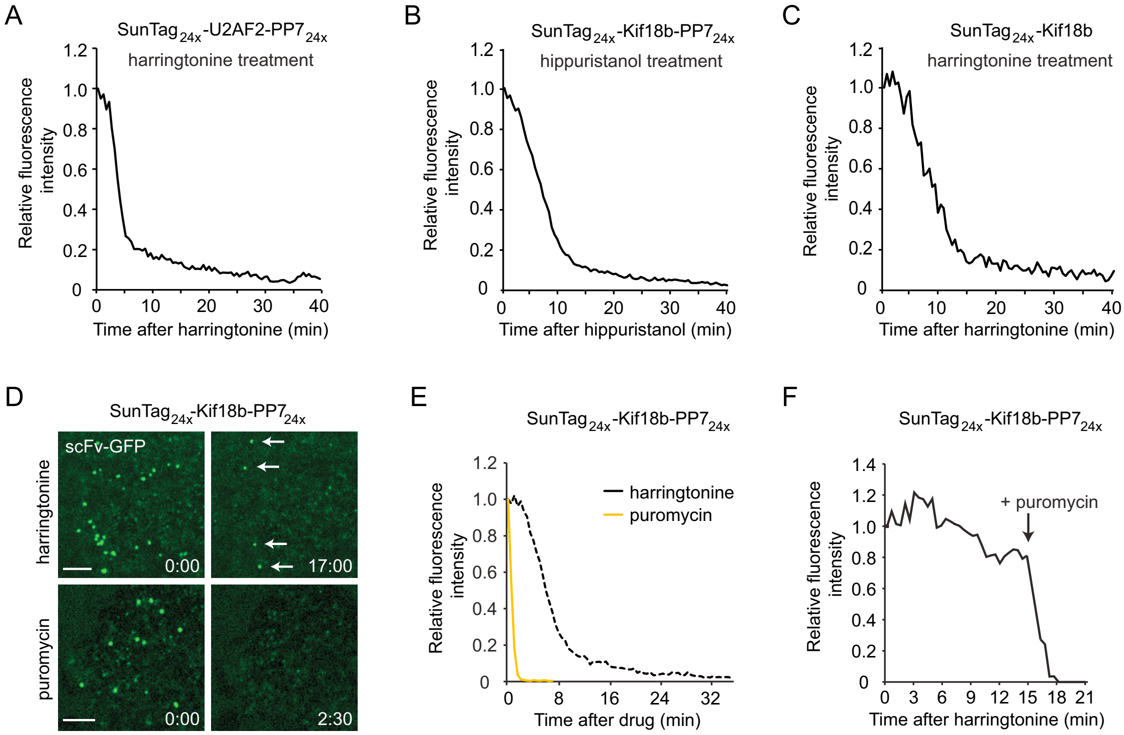

Figure S4. Validation of the Ribosome Stalling Phenotype, Related to Figure 5

(A) U2OS cells expressing scFv-GFP, PP7-2xmCherry-CAAX and indicated translation reporters were treated with harringtonine at t = 0 and translation-site intensity was quantified over time. Reporter containing a codon optimized version of the U2AF2 coding sequence (n = 3 independent experiments, 29 cells, 512 mRNAs).

(B) U2OS cells expressing the translation reporter (SunTag$_{24x}$-Kif18b-PP7$_{24x}$) were treated with another translation initiation inhibitor (hippuristanol) and translation-site intensity was quantified over time (n = 2 independent experiments, 14 cells, 515 mRNAs).

(C) U2OS cells expressing scFv-GFP, PP7-2xmCherry-CAAX and indicated translation reporters were treated with harringtonine at t = 0 and translation-site intensity was quantified over time. Harringtonine run-off experiments were also performed on a translation reporter lacking PP7 binding sites (SunTag$_{24x}$-Kif18b) (n = 2 independent experiments, 19 cells, 248 mRNAs).

(D) U2OS cells expressing scFv-GFP, PP7-2xmCherry-CAAX and indicated translation reporters were treated with harringtonine at t = 0 and translation-site intensity was quantified over time. Representative images in which stalled ribosome can be observed after harringtonine treatment (arrows). No ribosome stalling is observed after puromycin treatment (lower panel).

(E) U2OS cells expressing scFv-GFP, PP7-2xmCherry-CAAX and indicated translation reporters were treated with harringtonine at t = 0 and translation-site intensity was quantified over time. At t = 0, either harringtonine (re-plotted from Figure 2D) or puromycin (n = 3 independent experiments, 22 cells, 403 mRNAs) was added and translation-site intensity was quantified over time.

(F) U2OS cells expressing scFv-GFP, PP7-2xmCherry-CAAX and indicated translation reporters were treated with harringtonine at t = 0 and translation-site intensity was quantified over time. Sequential addition of harringtonine and then puromycin (n = 7 mRNAs). Scale bar, 5 μm.

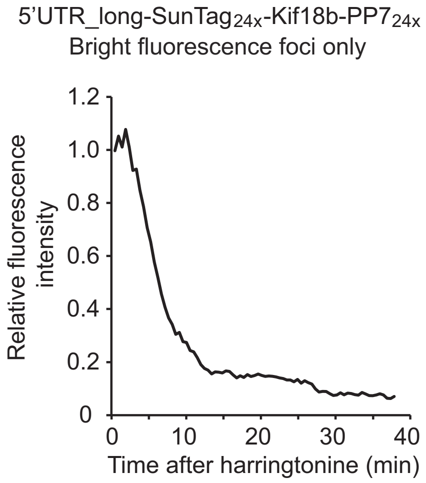

Figure S5. Ribosome Elongation Rates on Emi1 5′UTR_Long Containing mRNAs, Related to Figure 6

U2OS cells expressing scFv-GFP, PP7-2xmCherry-CAAX and the Emi1 5′UTR_long translation reporter were treated with harringtonine at t = 0. The fluorescence intensity of very bright translation sites was quantified over time (n = 3 independent experiments, 29 cells, 39 mRNAs).

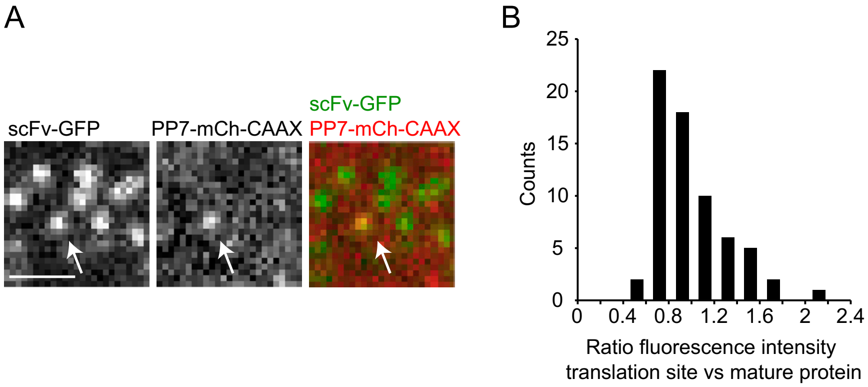

Figure S6. Single Ribosomes Translate the Emi1 5′UTR_Long mRNA, Related to Figure 7

U2OS cells expressing scFv-GFP, PP7-2xmCherry-CAAX and the Emi1 5′UTR_long translation reporter were imaged using a very short (30 ms) exposure time, so fully synthesized, freely diffusing mature SunTag-Kif18b molecules can be observed together with translation sites. Translation sites could be distinguished from fully synthesized SunTag molecules, as they co-migrated with mRNAs for multiple (> 5) consecutive time points.

(A) Representative image of a single translation site (arrow) surrounded by multiple mature SunTag molecules.

(B) Quantification of fluorescence intensities of translation sites and mature protein. The fluorescence intensity of a single translation sites was compared to the average fluorescence intensity of 5 nearby mature SunTag molecules.

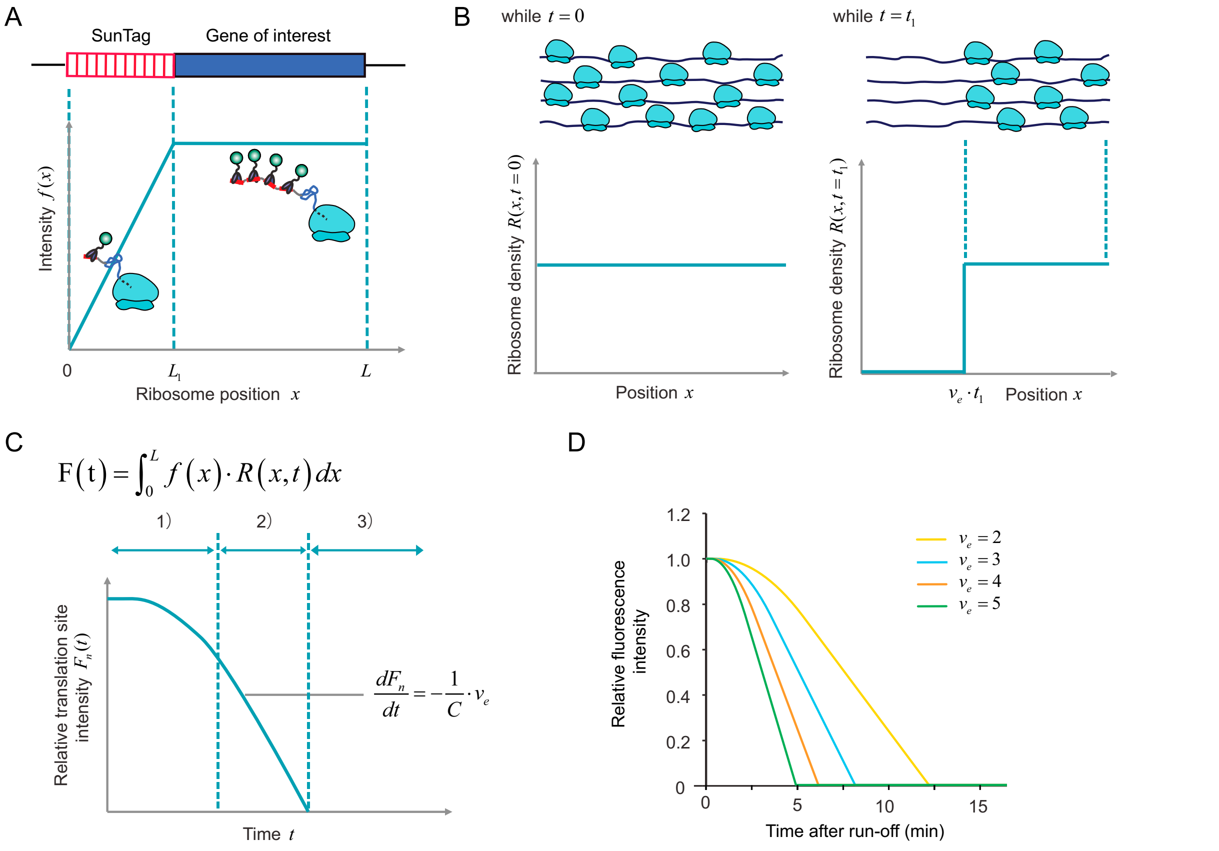

Figure S7. Modeling of Translation-Site Intensity, Related to Experimental Procedures

(A) Intensity from a single ribosome mainly depends on ribosome location on the mRNA. Due to the synthesis of SunTag peptides, ribosome intensity will increase initially as the ribosome moves toward the 3′ end until SunTag peptides are fully synthesized and exposed. A typical curve for intensity function *f(x)* is shown. For simplicity, a linear function was used to simulate the intensity increase.

(B) Ribosome density changes during ribosome run-off. When there are no new initiation events, already bound ribosomes will runoff the mRNA from 5′ to 3′ end. Examples of the ribosome density function at $t = 0$ and $t = t_1$ are shown.

(C) Translation-site intensity is dependent on both intensity from single ribosomes as well as ribosome density throughout the mRNA. A formula describing translation-site intensity is shown on top. A typical curve of intensity change during ribosome run-off process is shown at the bottom with three clear stages labeled using numbers. Intensity decreases linearly during the second stage, whose first order derivative could be used to derive elongation rate.

(D) Example results of simulations of harringtonine run-off from the Kif18b reporter ($SunTag_{24x}$-Kif18b), which were run using different elongation rates (2, 3, 4, 5 codons/s). Run-off starts at $t = 0$.

Cell, Volume *165*

Supplemental Information

Dynamics of Translation of
Single mRNA Molecules In Vivo

Xiaowei Yan, Tim A. Hoek, Ronald D. Vale, and Marvin E. Tanenbaum

Extended Experimental Procedures

General considerations for quantifying translation based on scFv-GFP fluorescence intensity values

In this study, we provide estimates for the number of ribosomes per mRNA, reveal ribosome initiation and translocation rates and provide measurements of ribosome stalling. All these values were calculated based on fluorescence intensities of the scFv-GFP antibody bound to nascent polypeptides. It is important to note that such fluorescence intensities by themselves do not provide quantitative information, but only can be interpreted after taking several factors into consideration.

1) Ribosome position along the mRNA.

Ribosome position along the mRNA affects the number of SunTag peptides that have been synthesized, and thus the amount of scFv-GFP fluorescence associated with that ribosome/nascent polypeptide. We have generated a mathematical model to correct for this effect (see the section "Modeling of the fluorescence intensity of translation sites" below, and Figure S7). It is important to note that this model assumes a homogeneous distribution of ribosomes along the mRNA which is likely accurate when averaging many mRNAs, but stochastic distributions can occur on an individual mRNA molecule. Also, non-homogenous distribution of ribosomes will not bias the estimate of ribosome number in ensemble statistics, as it is equally likely to underestimate or to overestimate ribosome number.

2) Variability in the number of scFv-GFPs bound to a SunTag peptide array.

The fluorescence intensity of a nascent polypeptide depends on the number of scFv-GFP molecules bound. The number of scFv-GFP antibody molecules bound to a single SunTag polypeptide can, however, vary. This variation may be limited though, as binding of the scFv-GFP to the 24 peptide SunTag array appears to saturate when sufficient cytoplasmic scFv-GFP is present, resulting in the binding of close to 24 scFv-GFPs per $SunTag_{24x}$ array (Tanenbaum et al., 2014). Variation in the number of scFv-GFPs per $SunTag_{24x}$ array will not affect the calculation

of the number of ribosomes per mRNA, as this variation will occur on both the nascent polypeptides and the fully synthesized and released SunTag protein in the same cells which is used to normalize the fluorescence intensity of the translation sites. However, when a lot of $SunTag_{24x}$ molecules have been synthesized and released into the cytoplasm, they can sequester the free scFv-GFP, leaving insufficient free scFv-GFP to bind all newly synthesized nascent SunTag peptides. Indeed, we have observed that at high expression levels of the reporter, translation sites become progressively dimmer after several hours, indicative of cytoplasmic depletion of the scFv-GFP. While such depletion of cytoplasmic scFv results both in dimmer translation sites and dimmer fully synthesized SunTag protein (used for normalization), and therefore does not affect our calculations of ribosome number per mRNA. Nonetheless, to circumvent this issue, we have used an inducible promoter to initiate reporter expression only ~1 hr before image acquisition. An additional source of variability in scFv-GFP binding to the SunTag peptides is introduced by the lag time that exists between synthesis of a new SunTag peptide and binding to the scFv-GFP. However, our experiments show that this lag time likely does not substantially decrease overall fluorescence intensity of the translation site, indicating that the binding reaction is relatively fast. This lag time likely depends on the level of free cytoplasmic scFv-GFP, and could therefore vary under different conditions.

3) Fluorescence decay of the scFv-GFP signal at translation sites.

In several experiments, including cases of natural translation shutdown and harringtonine ribosome run-off experiments, we observed a reduction in scFv-GFP fluorescence intensity at translation sites. We generally interpret this fluorescence decay as ribosomes terminating translation at the stop codon, which results in nascent chain release and ribosome recycling. While this conclusion is likely valid in most cases, alternative explanations are possible. For example, ribosomes stall for extended periods of time on mRNAs with chemical damage or on the Xbp1 nucleotide pausing sequence, but eventually scFv-GFP signal associated with stalled translation sites does decrease. While it is possible that ribosomes eventually normally read through such stall sequences and terminate normally at a stop codon, it is also possible that ribosomes are removed from mRNAs after a prolonged stalling event. Additional experiments

are required to distinguish these events, for example by placing the stall-inducing sequence upstream of the SunTag peptide array.

4) Photobleaching

Photobleaching rates were determined by measuring the total GFP signal of the entire cell over time. In experiments where photobleaching resulted in a fluorescence decrease of >10% of the initial fluorescence intensity, fluorescence intensities of translation sites were corrected for photobleaching.

Calculation of Ribosome Elongation

Ribosome elongation rates were determined by three different methods.

Population measurements from harringtonine ribosome run-off (Related to Figure 2)

The linear phase of the decrease in GFP fluorescence after new ribosome translation is prevented by addition of harringtonine provides information on the rate of ribosome movement along the mRNA. A model for the fluorescence signal is provided below (See Modeling of the fluorescence intensity of translation sites) along with details of how the data was fit to determine the elongation rate.

Calculating the ribosome elongation rate based on single mRNA run-off times (Related to figure S2E)

The total time required for run-off of all ribosomes from individual mRNAs represents the time until the last (*i.e.* most 5') ribosome completes translation after harringtonine treatment. Individual translation site intensities were quantified using the spot_counter ImageJ plugin (http://fiji.sc/SpotCounter) developed by Nico Stuurman, which draws a box around the selected translation site and scores the number of time-points that the translation site was detected. Automatic tracking was manually curated for all spots to ensure high quality tracking data. The average run-off time for all mRNAs was then determined, and we subtracted 60 s from this time, the time required for harringtonine to enter the cell (Ingolia et al., 2011). We

then divided the reporter length (1462 codons for the $SunTag_{24x}$-Kif18b-$PP7_{24x}$ reporter) by the corrected run-off time to obtain the ribosome elongation rate. This rate assumes that the final ribosome loaded near the 5’ end of the mRNA, which may not be true for all mRNAs. This assumption may slightly overestimate ribosome elongation rates, as the actual distance covered by the most 5’ ribosome covers until completion of translation will be slightly less than 1462 codons, if it is downstream of the start codon at the time of harringtonine addition. However, considering that most mRNAs have ~20 ribosomes and the average inter-ribosomal distance is therefore ~70 codons, the most 5’ ribosome will usually be within 70 codons of the start site, which represents a 5% error.

Determining the ribosome elongation rate from single ribosome tracking data (Related to Figure 7).

By limiting translation initiation using the Emi1 5’UTR_long fused to the translation reporter, individual ribosomes could be observed decoding an mRNA molecule. For each single ribosome translation event, we measured the duration of the event, by determining the first time-point that GFP could reliably be detected over the background until the GFP signal disappeared. Only tracks were included that lasted less than 12 min. However, determining ribosome elongation rate based on such measurements was non-trivial, as this requires knowledge of the precise position of the ribosome along the transcript when the nascent chain signal is first detected; presumably a number of SunTag peptides need to have been synthesized before sufficient signal has accumulated to allow detection. We therefore needed to identify the fluorescence intensity detection limit. To accomplish this, we first determined the maximal fluorescence intensity associated with a single ribosomes; this occurs when the entire SunTag peptide array has been synthesized (Figure 7C). Maximal intensity was calculated by aligning all of the traces (n = 44) at the last time point in which fluorescence was observed and averaging the fluorescence intensity of the last 3 time points before polypeptide dissociation. We then aligned all 44 traces at the first point we could visually detect fluorescence and found that we could reliably detect signal from single ribosomes when their nascent chain-associated fluorescence was ~1/3 of the maximal intensity (Figure 7B). From this, we infer that ~8 of the 24

SunTag peptides likely have been synthesized at this point in time, which places these ribosomes on codon 245 (taking into account that SunTag peptides will not be labeled when inside the ribosome exit tunnel). Therefore, from the time of first fluorescence measurement to the release of the completed polypeptide on the stop codon (406 s on average for n = 44 individual ribosome translocations), the ribosome moves 1462-245 = 1217 codons (since the whole transcript is 1462 codons long). The ribosome elongation rate can be calculated as the number of codons / time: 1217/406 = 3.0 codons/s.

Translation Initiation Rate

Estimation of steady state initiation rate

To maintain a constant ribosome occupancy, the rate of removal of ribosomes from the mRNA molecule after completion of translation, must be balanced by new ribosome loading on the mRNA through translation initiation. To estimate the translation initiation rate, we first calculated the average inter-ribosomal distance, which is 58-146 codons assuming a ribosome number per mRNA of 10-25 ribosomes on the $SunTag_{24x}$-Kif18b reporter. Considering the ribosome elongation rate of 3.5 codons/s (for the Kif18b reporter), a ribosome will complete translation on average every 58/3.5 to 146/3.5 = 17 to 42 s. Thus, if constant ribosome occupancy is maintained, the initiation rate must be between 1/17 s^{-1} to 1/42 s^{-1} or 1.4 to 3.6 min^{-1}. These values represent average initiation rates, as the initiation rate on single mRNA molecules will vary over time.

Comparing the translation initiation rate on newly transcribed versus polysomal mRNAs.

Through the experiments in which single translating ribosomes were tracked, we determined that ribosomes could be first detected when ~1/3 of SunTag peptides had been synthesized (when the ribosome is positioned around codon 245 (see above)). Thus, when the first, pioneer ribosome was detected on a newly transcribed mRNA molecule, it was likely near this position. After initial fluorescence detection of the pioneer ribosome, translation site signal increased continuously for several min (Figure 4B). This fluorescence increase was presumably both due to the increase in the number of synthesized SunTag peptides on the pioneer ribosome, as well

as due to additional ribosomes that initiated after the pioneer ribosome. The translation site fluorescence intensity reached a steady state ~6 min after the initial detection of fluorescence (Figure 4B), indicating that the ribosome density remained constant from that point on. At a translocation rate of 3-3.5 codons/s, it would take the pioneer ribosome ~6 min to reach the stop codon from the position of initial detection (codon 245). From these calculations, we conclude that the ribosome density on the mRNA has already reached its maximal value as soon as the pioneer ribosome completes translation. Since ribosome density is a function of ribosome initiation rate and elongation rate, we infer that the initiation rate on the newly transcribed mRNA must be identical to that on mRNAs with established polysomes, assuming that the elongation rates are on average similar.

Estimation of Ribosome Number per mRNA

To obtain the number of ribosomes per mRNA molecule, we set up a normalization experiment to compare the average intensity of the single translation sites with the intensity coming from single, fully synthesized $SunTag_{24x}$-Kif18b proteins (Figure S2A). As the fully synthesized SunTag-Kif18b proteins encompass 24 copies of the SunTag peptide, their intensity should be comparable with the fluorescence intensity associated with a ribosome at the 3’ end on the mRNA. However, the ribosomes at the 5’ end of the mRNA will be much dimmer because they have not fully synthesized the SunTag epitopes. Thus, dividing the translation site intensity by the intensity from a single fully synthesized SunTag-Kif18b protein will underestimate the actual number of ribosomes on an mRNA. A correction factor derived from our model presented below was used to obtain a more accurate estimate of ribosome number.

Modeling of the fluorescence intensity of translation sites

Every active translation site is composed of one single mRNA with multiple ribosomes undergoing translation. The SunTag peptides produced from ribosomes are bound by scFv-GFP antibodies floating in the cytoplasm, giving rise to a fluorescence signal from the translation complex. Ribosomes on the 5’ end of the mRNA are still translating part of the SunTag peptide, thus resulting in a partial fluorescence signal from the emerging polypeptide chain, while

ribosomes that are translocating on the 3' end of the gene have already synthesized the entire SunTag peptide array and are fully covered by the antibodies. Therefore, to interpret intensity changes from a transcript loaded with multiple ribosomes, we have generated a model that takes into account ribosome location as well as density on the mRNA as a function of time.

The intensity from a single ribosome will increase gradually as the ribosome moves towards the 3' end until it reaches a plateau where all SunTag peptides are synthesized (Figure S7A). This relationship is described as following:

$$f(x) = \begin{cases} g(x) & x \in [0, L_1) \\ I_{sun} & x \in [L_1, L] \end{cases} \quad \text{.. (1)}$$

The parenthesis and square bracket are notations for intervals. The two numbers are the endpoints of the interval. Parenthesis indicates exclusion of corresponding endpoint while square bracket indicates inclusion of it.

$f(x)$: intensity from a single ribosome at position x

$g(x)$: intensity from a single ribosome at position x when x is in between 0 and L_1, simplified as a linear function in the diagram in Figure S7A

x : ribosome position on the mRNA at time t

L : the length of positions that can be covered by ribosomes, which includes the open reading frame decoding both the SunTag peptides and the gene of interest (GOI), as shown in Figure S7A

L_1 : position on the transcript where intensity from a ribosome at that position reaches plateau intensity, as shown in Figure S7A

I_{sun} : the intensity of a single SunTag array that is fully covered by scFv-GFP, with the intensity depending on the number of SunTag peptides fused to the gene of interest, multiplied by the intensity of a single scFv-GFP antibody

$$I_{sun} = n_{sun} \times i_{GFP} \quad \text{.. (2)}$$

n_{sun} : the number of SunTag peptides which are fused to the gene of interest (mostly 24 in this study)

i_{GFP} : the intensity of a single scFv-GFP

Thus the average intensity from a single translation site could therefore be described using the following formula:

$$F(t) = \int_0^L f(x) \cdot R(x,t) \cdot dx \quad \text{......} \quad (3)$$

$F(t)$: average fluorescence intensity from a single translation site at time t

$R(x,t)$: ribosome probability density at position x at time t.

With the assumption that ribosomes are randomly positioned in a population of transcripts (while this distribution may not be homogeneous on a single mRNA, it will approximate a random distribution when averaging hundreds of mRNAs, as is done in these experiments; thus this model is only accurate when a large number of mRNAs is analyzed), the probability density will be the same at different positions on the transcript at steady state (Figure S7B, left), which leads to:

$$R(x,t) = R_s$$

$$R_s = \frac{n_r}{L_r} \quad \text{......} \quad (4)$$

R_s : ribosome density during steady state

n_r : the number of ribosomes on a single transcript

L_r : the length of transcript that is covered by the ribosomes

Modeling of shutdown process and elongation rate

Based on the above, we are able to model fluorescence intensity as a function of time. We use the translation shutdown process as an example:

When new ribosomes are no longer added at the 5' end (*e.g.* when initiation is blocked by harringtonine), previously bound ribosomes will run off the transcript from the 5' to the 3' end. This will change the ribosome distribution as a function of time t, as described by the following equations and the illustration depicted in Figure S7B:

$$R(x,t) = \begin{cases} 0 & x \in [0, v_e \cdot t) \\ R_s & x \in [v_e \cdot t, L] \end{cases}$$

………………………………………… (5)

v_e : elongation rate; assumed to be a constant in the model.

If we incorporate this function into equation (3), we derive the following formula, which describes the change in intensity of a single translation site as a function of time ($t = 0$ marking the beginning of harringtonine treatment) (Figure S7C).

$$F(t) = \begin{cases} R_s \cdot \left[\int_{v_e \cdot t}^{L_1} g(x) \cdot dx + I_{sun}(L - L_1)\right] & t \in \left[0, \frac{L_1}{v_e}\right) \quad 1) \\ R_s \cdot I_{sun}(L - v_e \cdot t) & t \in \left[\frac{L_1}{v_e}, \frac{L}{v_e}\right) \quad 2) \\ 0 & t \in \left[\frac{L}{v_e}, \infty\right) \quad 3) \end{cases}$$ ………………… (6)

This formula describes absolute intensity, which is dependent on many variables including the laser power used to excite the fluorophore. Therefore, we normalize this equation to the initial steady state fluorescence using the constant C_1:

$$F_s = R_s \cdot \left[\int_0^{L_1} g(x) \cdot dx + I_{sun}(L - L_1)\right] = R_s \cdot C_1$$ ………………… (7)

$$F_n(t) = {F(t)}/{F_s} = \begin{cases} \left[\int_{v_e \cdot t}^{L_1} g(x) \cdot dx + I_{sun}(L - L_1)\right] / C_1 & t \in \left[0, \frac{L_1}{v_e}\right) \quad 1) \\ I_{sun}(L - v_e \cdot t) / C_1 & t \in \left[\frac{L_1}{v_e}, \frac{L}{v_e}\right) \quad 2) \\ 0 & t \in \left[\frac{L}{v_e}, \infty\right) \quad 3) \end{cases}$$ ………… (8)

F_s: translation site intensity during steady state

C_1 : constant number, $C_1 = \int_0^{L_1} g(x) \cdot dx + I_{sun}(L - L_1)$

To further simplify the model, a linear function was used to describe $g(x)$, and the results are shown in Figure S7D. Data acquired in real experiments have stalling events, which complicates fitting the data to the simulated results. To simplify the determination of the elongation rate from the harringtonine run-off experiments, we only fit the second stage of the curve (Figure S7C), which is given by:

$$F_n(t) = {F(t)}/{F_s} = {I_{sun}(L - v_e \cdot t)}/{C_1} = -\frac{1}{C} \cdot v_e \cdot t + \frac{L}{C} \qquad \text{......................} \quad (9)$$

C : constant number, $C = {C_1}/{I_{sun}}$

The first order derivative of this stage will give us the elongation rate v_e:

$$\frac{dF_n(t)}{dt} = -\frac{1}{C} \cdot v_e \qquad \text{..} \quad (10)$$

Intensity from stalled or slowly elongating translation sites contributes about 10-20% of the initial fluorescence intensity (Figure 2D), which will lead to underestimation of the slope without correction. To overcome this, a linear regression was fit to the second half of the curve after initial run-off, and the intercept value was subtracted to exclude the influence of stalled translation sites. The curve was normalized after correction, and relative fluorescence intensity in between 40-80% was fit with a linear function to extract the slope. Elongation rate was calculated using the formula mentioned above. However, several more corrections were applied to the previous equation to account for the influence of 1) the codons translated before the SunTag array (23 aa) as well as the codons in between the SunTag array and the GOI (6 aa) 2) the nascent peptide buried in the ribosome exit tunnel (36 aa was used).

Modeling of the number of ribosomes on a single mRNA

Fully synthesized $SunTag_{24x}$-Kif18b proteins encompass 24 copies of the SunTag peptide, so their intensity should be comparable to the fluorescence intensity of a ribosome at the 3' end of the mRNA. However, ribosomes at the 5' end of the mRNA will be dimmer, since they have not fully synthesized the SunTag epitopes. Thus, dividing the translation site intensity by the intensity of a single fully synthesized $SunTag_{24x}$-Kif18b protein will underestimate the actual number of ribosomes on an mRNA.

To overcome this, a correction factor γ was applied. As described earlier in equation (4), ribosome density R_s is:

$$R_s = \frac{n_r}{L_r}$$

If we assume the transcript is fully covered by the ribosomes during steady state, the ribosome number n_r can be derived as:

$$n_r = R_s \cdot L_r = R_s \cdot L = {(F_s \cdot L)}/{C_1} = \gamma \cdot {F_s}/{I_{sun}} \quad \text{......................................} \quad (11)$$

The constant $\gamma = {2L}/{(2L - L_1)}$ when a linear function was used to describe $g(x)$. As discussed in the previous section, more corrections were applied to the equation to account for influence of things such as ribosome exit tunnel length. Thus, the actual correction factor used for the Kif18b reporter is 1.32 (1/0.76).

Plasmid sequences

SunTag$_{24x}$-Kif18b-PP7$_{24x}$ - Complete plasmid sequence

gacggatcgggagatctcccgatcccctatggtgcactctcagtacaatctgctctgatgccgcatagttaagccagtatctgctccctgcttgtgtgttg
gaggtcgctgagtagtgcgcgagcaaaatttaagctacaacaaggcaaggcttgaccgacaattgcatgaagaatctgcttagggttaggcgttttgc
gctgcttcgcgatgtacgggccagatatacgcgttgacattgattattgactagttattaatagtaatcaattacggggtcattagttcatagcccatata
tggagttccgcgttacataacttacggtaaatggcccgcctggctgaccgcccaacgacccccgcccattgacgtcaataatgacgtatgttcccatag
taacgccaatagggactttccattgacgtcaatgggtggagtatttacggtaaactgcccacttggcagtacatcaagtgtatcatatgccaagtacgc
cccctattgacgtcaatgacggtaaatggcccgcctggcattatgcccagtacatgaccttatgggactttcctacttggcagtacatctacgtattagtc
atcgctattaccatggtgatgcggttttggcagtacatcaatgggcgtggatagcggtttgactcacggggatttccaagtctccaccccattgacgtca
GCTCAGCTTTCTAGACCGCGcgcaaatgggcggtaggcgtgtacggtgggaggtctatataagcagagctctccctatcagtgatagagatc
tccctatcagtgatagagatcgtcgacgagctcgtttagtgaaccgtcagatcgcctggagacgccatccacgctgttttgacctccatagaagacacc
gggaccgatccagcctccggactctagcgtttaaacttAAGCTTGCCACCATGGGCGCCATCATACACGCGGCCGCAATCATcggt
ccgGGTGGATCTGGAGGTGGAGGTTCTGGAGGAGAAGAACTTTTGAGCAAGAATTATCATCTTGAGAACGAAGTG
GCTCGTCTTAAGAAAGGTTCTGGCAGTGGAGAAGAACTGCTTTCAAAGAATTACCACCTGGAAAATGAGGTAGCT
AGACTGAAAAAGGGGAGCGGAAGTGGGGAGGAGTTGCTGAGCAAAAATTATCATTTGGAGAACGAAGTAGCAC
GACTAAAGAAAGGGTCCGGATCGGGTGAGGAGTTACTCTCGAAAAATTATCATCTCGAAAACGAAGTGGCTCGGC
TAAAAAAGGGCAGTGGTTCTGGAGAAGAGCTATTATCTAAAAACTACCACCTCGAAAATGAGGTGGCACGCTTAA
AAAAGGGAAGTGGCAGTGGTGAAGAGCTACTATCCAAGAATTATCATCTTGAGAACGAGGTAGCGCGTTTGAAG
AAGGGTTCCGGCTCAGGAGAGGAACTGCTCTCGAAGAACTATCATCTTGAAAATGAGGTCGCTCGATTAAAAAAG
GGATCGGGCAGTGGTGAGGAACTACTTTCAAAGAATTACCACCTCGAAAACGAAGTAGCTCGATTAAAGAAAGGT
TCAGGGTCGGGTGAAGAATTACTGAGTAAAAATTATCATCTGGAAAATGAGGTAGCGAGACTAAAAAAGGGGAG
TGGTTCTGGCGAAGAGTTGCTATCGAAAAATTATCATCTTGAGAACGAAGTTGCTAGGCTCAAAAAGGGCTCAGG
CTCAGGCGAGGAGTTGCTCTCGAAAAACTACCACTTGGAAAATGAGGTCGCGAGGTTGAAAAAGGGGAGCGGGT
CGGGCGAGGAGTTATTGAGCAAAAACTATCATTTAGAGAACGAAGTCGCGCGCTTAAAGAAAGGCTCGGGCTCG
GGCGAAGAACTCTTATCGAAGAACTACCACCTCGAAAATGAGGTCGCCAGGTTGAAAAAGGGCAGTGGCAGCGG
GGAGGAACTCTTGAGCAAGAACTACCACTTGGAGAATGAGGTCGCGAGATTGAAGAAAGGGTCGGGGAGCGGC
GAGGAATTGCTCAGCAAGAATTATCATTTGGAGAACGAAGTCGCCAGGCTCAAGAAAGGCTCGGGGTCGGGGGA
GGAATTGTTGAGTAAAAACTACCACTTGGAAAATGAAGTCGCCAGGCTCAAAAAAGGGAGTGGGAGCGGCGAAG
AGTTATTGAGCAAAAATTACCACTTGGAGAACGAAGTGGCAAGGCTCAAGAAAGGGAGCGGCAGCGGGGAGGA
GCTCTTATCGAAGAACTACCACTTAGAGAATGAAGTCGCCCGCTTGAAGAAAGGCTCGGGGAGCGGGGAAGAGC
TCTTGAGCAAGAACTACCACTTGGAAAATGAGGTGGCGCGCTTGAAGAAAGGGAGCGGGAGCGGGGAAGAGTT
ACTATCTAAGAATTATCATCTCGAGAACGAGGTGGCTCGACTAAAGAAGGGCTCCGGCAGTGGGGAGGAACTCCT
GTCGAAGAACTATCATCTTGAAAATGAGGTTGCAAGACTTAAAAAGGGGTCCGGATCAGGTGAGGAACTACTCAG
TAAGAATTACCACCTGGAAAACGAAGTTGCACGTTTGAAGAAAGGATCAGGATCAGGCGAAGAACTGCTCTCAAA
AGATTATCATTTGGAAAATGAGGTTGCACGTTTAAAAAAGGGAAGTGGCAGTGGTGAGGAACTTCTGTCGAAAAA
TTATCATCTCGAGAATGAAGTAGCCCGACTTAAAAAGGGTTCTGGCTCGGGTCAGCGGCCGCACCGGTCAGCAGT
GGAGGACAGCACGCTGCAAGTAGTGGTACGGGTGCGGCCCCCCACCCCTCGGGAGCTGGACAGTCAGCGGCGGC
CAGTGGTTCAGGTGGTGGACGAGCGGGTGCTGGTGTTTAACCCTGAGGAGCCCGATGGAGGGTTCCCTGGCCTG
AAATGGGGTGGCACCCATGATGGCCCCAAGAAGAAGGGCAAAGACCTGACGTTTGTCTTTGACCGGGTCTTTGGC
GAGGCGGCCACCCAACAGGACGTGTTCCAGCACACCACGCACAGCGTCCTGGACAGCTTCCTCCAGGGCTACAAC
TGCTCAGTGTTTGCCTACGGGGCCACCGGGGCTGGGAAGACACACACCATGCTGGGAAGGGAGGGGGACCCCG
GCATCATGTACCTGACCACCGTGGAACTGTACAGGCGCCTGGAGGCCCGCCAGCAGGAGAAGCACTTCGAGGTG
CTCATCAGCTACCAGGAGGTGTATAATGAACAGATCCATGACCTCCTGGAGCCCAAGGGGCCCCTTGCCATCCGC
GAGGACCCCGACAAGGGGGTGGTGGTGCAAGGACTTTCTTTCCACCAGCCAGCCTCAGCCGAGCAGCTGCTGGA
GATACTGACCAGGGGGAACCGTAACCGCACGCAGCACCCCACTGATGCCAACGCGACTTCCTCCCGCTCCCATGCC

ATCTTCCAGATCTTTGTGAAGCAGCAGGACCGGGTTCCAGGACTGACCCAGGCTGTCCAGGTGGCCAAGATGAGC
CTGATTGACCTGGCTGGCTCAGAGCGGGCATCCAGCACCCATGCGAAGGGGGAGCGGCTGCGGGAGGGGGCCA
ACATCAACCGCTCTCTGCTGGCGCTCATCAACGTCCTCAATGCCTTGGCCGATGCAAAGGGCCGCAAGACCGCTGT
GCCCTACGCGGACAGCGCACTGACCCGCCTGCTCAAAGACTCCCTCGGGGGCAACTGCCGCACAGTGATGATCGC
TGCCATCAGCCCCTCCAGCCTGACCTACGAGGACACGTATAATACCCTCAAATATGCCGACCGGGCCAAGGAGATC
AGGCTCTCGCTGAAGAGCAATGTGACCAGCCTGGACTGTCACATCAGCCAGTATGCTACCATCTGCCAACAGCTCC
AGGCTGAGGTAGCCGCTCTGAGGAAGAAGCTCCAAGTGTATGAGGGGGGAGGCCAGCCCCCACCACAGGACCTC
CCAGGATCTCCCAAGTCGGGACCACCACCAGAACACCTTCCCAGCTCCCCCTTGCCACCCCACCCTCCCAGCCAGCC
CTGCACCCCAGAGCTCCCTGCAGGGCCTAGAGCCCTTCAAGAGGAGAGTCTGGGGATGGAGGCCCAGGTGGAGA
GGGCCATGGAAGGGAACTCTTCAGACCAGGAGCAGTCCCCAGAGGATGAGGATGAAGGCCCAGCTGAGGAGGT
TCCAACCCAGATGCCAGAGCAGAACCCCACACATGCACTGCCAGAGTCCCCTCGCCTGACCCTGCAGCCCAAGCCA
GTCGTGGGCCACTTCTCAGCACGGGAACTGGATGGGGACCGTTCTAAGCGGTTGGCCCTAAAGGTGCTGTGCGTT
GCCCAGCGGCAGTACTCCCTGCTCCAAGCAGCCAACCTCCTGACGCCCGACATGATCACAGAGTTTGAGACCCTAC
AGCAGCTGGTGCAAGAGGAAAAAATTGAGCCTGGGGCAGAGGCCTTGAGGACTTCAGGCCTGGCCAGGGGGGC
ACCTCTGGCTCAGGAGCTGTGTTCAGAGTCAAAGCCTCCAGGATACACTGGCCCTGTGACCCGGACTATGGCGAG
GCGACTGAGTGGCCCCCTGCACACCCTGGGAATCCCGCCTGGACCCAACTGCACCCCAGCCCAGGGGTCCCGATG
GCCCATGGAGAAGAAGAGGAGGAGACCAAGCGCCTTGGAGGCAGACAGTCCCATGGCCCCAAAGCGGGGCACC
AAGCGCCAGCGCCAGTCCTTCCTGCCCTGCCTAAGGAGAGGGTCTCTGCCTGACACCCAACCTTCACAGGGGCCCA
GCACCCCCAAAGGAGAAAGGGCCTCCTCCCCCTGCCATTCCCCTCGCGTTTGCCCAGCCACAGTCATCAAAAGCCG
GGTGCCCCTGGGCCCTTCCGCCATGCAGAACTGCTCCACCCCGCTGGCTCTGCCCACTCGAGACCTCAATGCCACC
TTTGATCTCTCTGAGGAGCCTCCCTCAAAGCCCAGTTTCCATGAATGCATTGGCTGGGACAAAATACCCCAGGAGC
TGAGCAGGCTGGACCAGCCCTTCATCCCCAGGGCACCTGTGCCCCTGTTCACCATGAAGGGCCCCAAGCCAACATC
TTCCCTCCCTGGGACCTCTGCCTGCAAGAAGAAGCGCGTTGCGAGTTCCTCAGTCTCCCATGGCCGCAGCCGCATC
GCCCGCCTCCCCAGCAGCACTTTGAAGAGGCCAGCTGGGCCCCTTGTACTCCCAGAGCTGCCCTTGAGTCCCCTGT
GCCCTAGCAACCGGAGGAATGGAAAGGACCTCATCAGGGTGGGGAGAGCACTCTCAGCAGGGAACGGCGTCACC
AAGGTGTCCGATATCTAACGACACGATTCGAAGATCGCATCGCTAGCCCGGTTGTACAGGTACCGGATCCTAAGG
TACCTAATTGCCTAGAAAGGAGCAGACGATATGGCGTCGCTCCCTGCAGGTCGACTCTAGAAACCAGCAGAGCAT
ATGGGCTCGCTGGCTGCAGTATTCCCGGGTTCATTAGATCCTAAGGTACCTAATTGCCTAGAAAGGAGCAGACGA
TATGGCGTCGCTCCCTGCAGGTCGACTCTAGAAACCAGCAGAGCATATGGGCTCGCTGGCTGCAGTATTCCCGGG
TTCATTAGATCCTAAGGTACCTAATTGCCTAGAAAGGAGCAGACGATATGGCGTCGCTCCCTGCAGGTCGACTCTA
GAAACCAGCAGAGCATATGGGCTCGCTGGCTGCAGTATTCCCGGGTTCATTAGATCCTAAGGTACCTAATTGCCTA
GAAAGGAGCAGACGATATGGCGTCGCTCCCTGCAGGTCGACTCTAGAAACCAGCAGAGCATATGGGCTCGCTGG
CTGCAGTATTCCCGGGTTCATTAGATCCTAAGGTACCTAATTGCCTAGAAAGGAGCAGACGATATGGCGTCGCTCC
CTGCAGGTCGACTCTAGAAACCAGCAGAGCATATGGGCTCGCTGGCTGCAGTATTCCCGGGTTCATTAGATCCTAA
GGTACCTAATTGCCTAGAAAGGAGCAGACGATATGGCGTCGCTCCCTGCAGGTCGACTCTAGAAACCAGCAGAGC
ATATGGGCTCGCTGGCTGCAGTATTCCCGGGTTCATTAGATCCTAAGGTACCTAATTGCCTAGAAAGGAGCAGAC
GATATGGCGTCGCTCCCTGCAGGTCGACTCTAGAAACCAGCAGAGCATATGGGCTCGCTGGCTGCAGTATTCCCG
GGTTCATTAGATCCTAAGGTACCTAATTGCCTAGAAAGGAGCAGACGATATGGCGTCGCTCCCTGCAGGTCGACTC
TAGAAACCAGCAGAGCATATGGGCTCGCTGGCTGCAGTATTCCCGGGTTCATTAGATCCTAAGGTACCTAATTGCC
TAGAAAGGAGCAGACGATATGGCGTCGCTCCCTGCAGGTCGACTCTAGAAACCAGCAGAGCATATGGGCTCGCT
GGCTGCAGTATTCCCGGGTTCATTAGATCCTAAGGTACCTAATTGCCTAGAAAGGAGCAGACGATATGGCGTCGC
TCCCTGCAGGTCGACTCTAGAAACCAGCAGAGCATATGGGCTCGCTGGCTGCAGTATTCCCGGGTTCATTAGATCC
TAAGGTACCTAATTGCCTAGAAAGGAGCAGACGATATGGCGTCGCTCCCTGCAGGTCGACTCTAGAAACCAGCAG
AGCATATGGGCTCGCTGGCTGCAGTATTCCCGGGTTCATTAGATCCTAAGGTACCTAATTGCCTAGAAAGGAGCA
GACGATATGGCGTCGCTCCCTGCAGGTCGACTCTAGAAACCAGCAGAGCATATGGGCTCGCTGGCTGCAGTATTC
CCGGGTTCATTAGATCTcgcgAAGGGCGAATTCacgaGGCGCGCCggatcGCGATCGCaatcTTAATTAACTCGAGtct
agagggcccgtttaaacccgctgatcagcctcgactgtgccttctagttgccagccatctgttgtttgcccctcccccgtgccttccttgaccctggaagg
tgccactcccactgtcctttcctaataaaatgaggaaattgcatcgcattgtctgagtaggtgtcattctattctggggggtggggtggggcaggacagc

aagggggaggattgggaagacaatagcaggcatgctggggatgcggtgggctctatggcttctgaggcggaaagaaccagctggggctctagggggg
tatccccacgcgccctgtagcggcgcattaagcgcggcgggtgtggtggttacgcgcagcgtgaccgctacacttgccagcgccctagcgcccgctcct
ttcgctttcttcccttcctttctcgccacgttcgccggctttccccgtcaagctctaaatcgggggctccctttagggttccgatttagtgctttacggcacct
cgaccccaaaaaacttgattagggtgatggttcacgtagtgggccatcgccctgatagacggtttttcgccctttgacgttggagtccacgttctttaata
gtggactcttgttccaaactggaacaacactcaaccctatctcggtctattcttttgatttataagggattttgccgatttcggcctattggttaaaaaatg
agctgatttaacaaaaatttaacgcgaattaattctgtggAATGTGTGTCAGTTAGGGTGTGGAAAGTCCCCAGGCTCCCCAGCA
GGCAGAAGTATGCAAAGCATGCATCTCAATTAGTCAGCAACCAGGTGTGGAAAGTCCCCAGGCTCCCCAGCAGGC
AGAAGTATGCAAAGCATGCATCTCAATTAGTCAGCAACCATAGTCCCGCCCCTAACTCCGCCCATCCCGCCCCTAAC
TCCGCCCAGTTCCGCCCATTCTCCGCCCCATGGCTGACTAATTTTTTTTATTTATGCAGAGGCCGAGGCCGCCTCTG
CCTCTGAGCTATTCCAGAAGTAGTGAGGAGGCTTTTTTGGAGGCCTAGGCTTTTGCAAAAAGCTCCcgggagcttgtat
atccattttcggatctgatcagcacgtgttgacaattaatcatcggcatagtatatcggcatagtataatacgacaaggtgaggaactaaaccatggcc
aagttgaccagtgccgttccggtgctcaccgcgcgcgacgtcgccggagcggtcgagttctggaccgaccggctcgggttctcccgggacttcgtgga
ggacgacttcgccggtgtggtccgggacgacgtgaccctgttcatcagcgcggtccaggaccaggtggtgccggacaacaccctggcctgggtgtgg
gtgcgcggcctggacgagctgtacgccgagtggtcggaggtcgtgtccacgaacttccgggacgcctccgggccggccatgaccgagatcggcgagc
agccgtgggggcgggagttcgccctgcgcgacccggccggcaactgcgtgcacttcgtggccgaggagcaggactgacacgtgctacgagatttcga
ttccaccgccgccttctatgaaaggttgggcttcggaatcgttttccgggacgccggctggatgatcctccagcgcggggatctcatgctggagttcttc
gcccaccccaacttgtttattgcagcttataatggttacaaataaagcaatagcatcacaaatttcacaaataaagcatttttttcactgcattctagttg
tggtttgtccaaactcatcaatgtatcttatcatgtctgtataccgtcgacctctagctagagcttggcgtaatcatggtcatagctgtttcctgtgtgaaa
ttgttatccgctcacaattccacacaacatacgagccggaagcataaagtgtaaagcctggggtgcctaatgagtgagctaactcacattaattgcgtt
gcgctcactgcccgctttccagtcgggaaacctgtcgtgccagctgcattaatgaatcggccaacgcgcggggagaggcggtttgcgtattgggcgctc
ttccgcttcctcgctcactgactcgctgcgctcggtcgttcggctgcggcgagcggtatcagctcactcaaaggcggtaatacggttatccacagaatca
ggggataacgcaggaaagaacatgtgagcaaaaggccagcaaaaggccaggaaccgtaaaaaggccgcgttgctggcgtttttccataggctccgc
ccccctgacgagcatcacaaaaatcgacgctcaagtcagaggtggcgaaacccgacaggactataaagataccaggcgtttccccctggaagctccc
tcgtgcgctctcctgttccgaccctgccgcttaccggatacctgtccgcctttctcccttcgggaagcgtggcgctttctcatagctcacgctgtaggtatc
tcagttcggtgtaggtcgttcgctccaagctgggctgtgtgcacgaaccccccgttcagcccgaccgctgcgccttatccggtaactatcgtcttgagtcc
aacccggtaagacacgacttatcgccactggcagcagccactggtaacaggattagcagagcgaggtatgtaggcggtgctacagagttcttgaagt
ggtggcctaactacggctacactagaagaacagtatttggtatctgcgctctgctgaagccagttaccttcggaaaaagagttggtagctcttgatccg
gcaaacaaaccaccgctggtagcggtttttttgtttgcaagcagcagattacgcgcagaaaaaaaggatctcaagaagatcctttgatcttttctacgg
ggtctgacgctcagtggaacgaaaactcacgttaagggattttggtcatgagattatcaaaaaggatcttcacctagatccttttaaattaaaaatgaa
gttttaaatcaatctaaagtatatatgagtaaacttggtctgacagttaccaatgcttaatcagtgaggcacctatctcagcgatctgtctatttcgttcat
ccatagttgcctgactccccgtcgtgtagataactacgatacgggagggcttaccatctggccccagtgctgcaatgataccgcgagacccacgctcac
cggctccagatttatcagcaataaaccagccagccggaagggccgagcgcagaagtggtcctgcaactttatccgcctccatccagtctattaattgtt
gccgggaagctagagtaagtagttcgccagttaatagtttgcgcaacgttgttgccattgctacaggcatcgtggtgtcacgctcgtcgtttggtatggc
ttcattcagctccggttcccaacgatcaaggcgagttacatgatcccccatgttgtgcaaaaaagcggttagctccttcggtcctccgatcgttgtcaga
agtaagttggccgcagtgttatcactcatggttatggcagcactgcataattctcttactgtcatgccatccgtaagatgcttttctgtgactggtgagtac
tcaaccaagtcattctgagaatagtgtatgcggcgaccgagttgctcttgcccggcgtcaatacgggataataccgcgccacatagcagaactttaaa
agtgctcatcattggaaaacgttcttcggggcgaaaactctcaaggatcttaccgctgttgagatccagttcgatgtaacccactcgtgcacccaactg
atcttcagcatcttttactttcaccagcgtttctgggtgagcaaaaacaggaaggcaaaatgccgcaaaaaagggaataagggcgacacggaaatgt
tgaatactcatactcttcctttttcaatattattgaagcatttatcagggttattgtctcatgagcggatacatatttgaatgtatttagaaaaataaacaa
ataggggttccgcgcacatttccccgaaaagtgccacctgacgtc

BFP codon optimized sequence
TCGGAGCTAATAAAAGAGAACATGCATATGAAACTATACATGGAGGGTACGGTAGATAACCATCACTTCAAGTGC
ACGTCGGAAGGTGAGGGTAAGCCGTACGAGGGTACGCAAACGATGCGAATAAAAGTAGTTGAGGGTGGTCCGC
TACCGTTTGCGTTTGATATATTAGCGACGTCGTTTCTATACGGTTCGAAGACGTTTATAAATCACACGCAAGGTATA
CCGGATTTTTTTAAACAATCGTTCCCGGAAGGTTTCACGTGGGAGCGAGTAACGACGTATGAAGATGGTGGTGTA
TTAACGGCGACGCAAGATACGTCGTTACAAGACGGTTGCCTAATATACAATGTTAAGATACGAGGTGTTAACTTCA

CGTCGAACGGTCCGGTTATGCAAAAGAAGACGCTAGGTTGGGAAGCGTTTACGGAAACGTTATACCCGGCGGAT
GGTGGTTTAGAAGGTCGTAATGACATGGCGTTAAAATTAGTAGGTGGTTCGCACTTAATAGCGAACATAAAGACG
ACGTACCGTTCGAAAAAACCGGCGAAGAATCTAAAGATGCCGGGTGTATACTACGTAGATTACCGACTAGAACGT
ATAAAGGAGGCGAATAACGAGACGTACGTTGAACAACATGAAGTAGCGGTAGCGCGATACTGTGATTTACCGTC
GAAGCTAGGTCACAAACTAAAT

U2AF2 codon optimized sequence
tcCgaTttcgaTgagttcgagcggcagctcaacgagaataaacaagagcgggaTaaggagaaccggcatcggaagcgcagccacagccgctcta
gaagccgggaTcgcaaacgccggagccggagccgcgaccggcgcaaccgggaccagcggagcgcctcccgggacaggAGAAGAcgcagcaa
acctttgaccagaggcgctaaagaggagcacggtggactgattAGAtcccccagacacgagaagaagaagaaggtcAGAaaatactgggacgt
gccacccccaggctttgagcacatcaccccaatgcagtacaaggccatgcaagctgcTggtcagattccagccactgctcttctccccaccatgacccc
Agacggtctggccgtgaccctacgccggtgcccgtggtcgggagccagatgaccagacaagcccggcgcctctacgtgggcaacatcccctttggc
atcactgaggaggccatgatggatttcttcaacgcccagatgcgcctgggAggActgacccaggcccctggcaacccagtgttggctgtgcagattaa
ccaggacaagaattttgcctttttggagttccgctcagtggaTgagactacccaggctatggcctttgatggcatcatcttccagggccagtcactGaa
gatccgcaggcctcacgaTtaccagccTcttcctggcatgtcagagaacccctccgtctatgtgcctggAgttgtgtccactgtggtccccgaTtctgc
ccacaagctgttcatcggAggcCTGcccaactacctgaacgatgaccaggtcaaagagctgctgacatcctttgggcccctcaaggccttcaacctg
gtcaaggacagtgccacgggActctccaagggctacgccttctgtgagtacgtggacatcaacgtcacggatcaggccattgcTgggctgaacggca
tgcagctgggAgataagaagctgctggtccagagggcTagtgtgggagccaagaatgccacgctgagcaccatcaatcagacgcctgtgaccctgc
aagtgccgggcttgatgagctcccaggtgcagatggcggccacccTactgaggtcctgtgcctcatgaacatggtgctgcctgaggagctgctggac
gacgaggagtatgaggagatcgtggaggatgtgcgggacgagtgcagcaagtacgggcttgtcaagtccatcgagatcccccggcctgtggacggc
gtcgaggtgcccggctgcggaaagatctttgtggagttcacctctgtgtttgactgccagaaagccatgcagggcctgacgggccgcaagttcgccaa
cagagtggttgtcacaaaatactgtgaccccgactcttatcaccgccgggacttctgg

5x SunTag array
GAAGAACTTTTGAGCAAGAATTATCATCTTGAGAACGAAGTGGCTCGTCTTAAGAAAGGTTCTGGCAGTGGAGAA
GAACTGCTTTCAAAGAATTACCACCTGGAAAATGAGGTAGCTAGACTGAAAAAGGGGAGCGGAAGTGGGGAGG
AGTTGCTGAGCAAAAATTATCATTTGGAGAACGAAGTAGCACGACTAAAGAAAGGGTCCGGATCGGGTGAGGAG
TTACTCTCGAAAAATTATCATCTCGAAAACGAAGTGGCTCGGCTAAAAAAGGGCAGTGGTTCTGGAGAAGAGCTA
TTATCTAAAAACTACCACCTCGAAAATGAGGTGGCACGCTTAAAAAAGGGAAGTGGCAGTGGT

10x SunTag array
gaagaacttttgagcaagaattatcatcttgagaacgaagtggctcgtcttaagaaaggttctggcagtggagaagaactgctttcaaagaattacca
cctggaaaatgaggtagctagactgaaaaaggggagcggaagtggggaggagttgctgagcaaaaattatcatttggagaacgaagtagcacgac
taaagaaagggtccggatcgggtgaggagttactctcgaaaaattatcatctcgaaaacgaagtggctcggctaaaaaagggcagtggttctggaga
agagctattatctaaaaactaccacctcgaaaatgaggtggcacgcttaaaaaagggaagtggcagtggtgaagagctactatccaagaattatcat
cttgagaacgaggtagcgcgtttgaagaagggttccggctcaggagaggaactgctctcgaagaactatcatcttgaaaatgaggtcgctcgattaaa
aaagggatcgggcagtggtgaggaactactttcaaagaattaccacctcgaaaacgaagtagctcgattaaagaaaggttcagggtcgggtgaaga
attactgagtaaaaattatcatctggaaaatgaggtagcgagactaaaaaaggggagtggttctggcgaggaattgctatcgaaaaattatcatcttg
agaacgaagttgctaggctcaaaaagggctcaggctcaggc

Emi1 5’UTR_long
gttaggagggagagcagagcgcgcgcgtaaatcctagagaggcgggctaagctggactggggggagggtccgtcttccggaaagtctggattcccg
gacgagccgagttgctgctcaccgaactcccgttcgagagatgatcgaagaaagtcggctaccatttgtacccatcaaagatctccagatggaagcca
gcgctgaatttgggctgagattaggacttgcaggaggccggtccagaagacggcggaaggaatcttggcgggcgcacgcatgcgtgatagaccctcc
acacgtgtggccgggccgcggcctccccgtgctcggaggtcccgcccccggccgtagcatctttccggacgtggggagccggttgtaaagaagaaag
ttctaccctttctgtcaaa

Emi1 5’UTR_short
Aaagtaccagctggcgccttttaagagatacaggtctgtgaagcaggcaggttgctcagctgcccccggagcggttcctccacctgaggcagactcca
cgtcggctggc

scFv-GCN4-sfGFP-GB1 (referred to as scFv-GFP in the manuscript)
ATGGGCCCCGACATCGTGATGACCCAGAGCCCCAGCAGCCTGAGCGCCAGCGTGGGCGACCGCGTGACCATCAC
CTGCCGCAGCAGCACCGGCGCCGTGACCACCAGCAACTACGCCAGCTGGGTGCAGGAGAAGCCCGGCAAGCTGT
TCAAGGGCCTGATCGGCGGCACCAACAACCGCGCCCCCGGCGTGCCCAGCCGCTTCAGCGGCAGCCTGATCGGCG
ACAAGGCCACCCTGACCATCAGCAGCCTGCAGCCCGAGGACTTCGCCACCTACTTCTGCGCCCTGTGGTACAGCAA
CCACTGGGTGTTCGGCCAGGGCACCAAGGTGGAGCTGAAGCGCGGCGGCGGCGGCAGCGGCGGCGGCGGCAGC
GGCGGCGGCGGCAGCAGCGGCGGCGGCAGCGAGGTGAAGCTGCTGGAGAGCGGCGGCGGCCTGGTGCAGCCC
GGCGGCAGCCTGAAGCTGAGCTGCGCCGTGAGCGGCTTCAGCCTGACCGACTACGGCGTGAACTGGGTGCGCCA
GGCCCCCGGCCGCGGCCTGGAGTGGATCGGCGTGATCTGGGGCGACGGCATCACCGACTACAACAGCGCCCTGA
AGGACCGCTTCATCATCAGCAAGGACAACGGCAAGAACACCGTGTACCTGCAGATGAGCAAGGTGCGCAGCGAC
GACACCGCCCTGTACTACTGCGTGACCGGCCTGTTCGACTACTGGGGCCAGGGCACCCTGGTGACCGTGAGCAGC
TACCCATACGATGTTCCAGATTACGCTGGTGGAGGCGGAGGTTCTGGGGGAGGAGGTAGTGGCGGTGGTGGTTC
AGGAGGCGGCGGAAGCTTGGATCCAGGTGGAGGTGGAAGCGGTAGCAAAGGAGAAGAACTTTTCACTGGAGTT
GTCCCAATTCTTGTTGAATTAGATGGTGATGTTAATGGGCACAAATTTTCTGTCCGTGGAGAGGGTGAAGGTGATG
CTACAAACGGAAAACTCACCCTTAAATTTATTTGCACTACTGGAAAACTACCTGTTCCGTGGCCAACACTTGTCACT
ACTCTGACCTATGGTGTTCAATGCTTTTCCCGTTATCCGGATCACATGAAACGGCATGACTTTTTCAAGAGTGCCAT
GCCCGAAGGTTATGTACAGGAACGCACTATATCTTTCAAAGATGACGGGACCTACAAGACGCGTGCTGAAGTCAA
GTTTGAAGGTGATACCCTTGTTAATCGTATCGAGTTAAAGGGTATTGATTTTAAAGAAGATGGAAACATTCTTGGA
CACAAACTCGAGTACAACTTTAACTCACACAATGTATACATCACGGCAGACAAACAAAAGAATGGAATCAAAGCTA
ACTTCAAAATTCGCCACAACGTTGAAGATGGTTCCGTTCAACTAGCAGACCATTATCAACAAAATACTCCAATTGGC
GATGGCCCTGTCCTTTTACCAGACAACCATTACCTGTCGACACAATCTGTCCTTTCGAAAGATCCCAACGAAAAGCG
TGACCACATGGTCCTTCTTGAGTTTGTAACTGCTGCTGGGATTACACATGGCATGGATGAGCTCTACAAAGGTGGA
GGTCGGACCGAAGAGTACAAGCTTATCCTGAACGGTAAAACCCTGAAAGGTGAAACCACCACCGAAGCTGTTGAC
GCTGCTACCGCGGAAAAAGTTTTCAAACAGTACGCTAACGACAACGGTGTTGACGGTGAATGGACCTACGACGAC
GCTACCAAAACCTTCACGGTAACCGAATAA

PP7-2xmCherry-CAAX
ATGTCCAAAACCATCGTTCTTTCGGTCGGCGAGGCTACTCGCACTCTGACTGAGATCCAGTCCACCGCAGACCGTC
AGATCTTCGAAGAGAAGGTCGGGCCTCTGGTGGGTCGGCTGCGCCTCACGGCTTCGCTCCGTCAAAACGGAGCCA
AGACCGCGTATCGCGTCAACCTAAAACTGGATCAGGCGGACGTCGTTGATTGCTCCACCAGCGTCTGCGGCGAGC
TTCCGAAAGTGCGCTACACTCAGGTATGGTCGCACGACGTGACAATCGTTGCGAATAGCACCGAGGCCTCGCGCA
AATCGTTGTACGATTTGACCAAGTCCCTCGTCGCGACCTCGCAGGTCGAAGATCTTGTCGTCAACCTTGTGCCGCT
GGGCCGTCGTGCGGACCCGCTAGCCTCCTgcggccgcGGTGGTGGTACGCGTGATTCGACTAGTGGAGGAAGCGGA
GGAGGAgtgagcaagggcgaggaggataacatggccatcatcaaggagttcatgcgcttcaaggtgcacatggagggctccgtgaacggccacg
agttcgagatcgagggcgagggcgagggccgcccctacgagggcacccagaccgccaagctgaaggtgaccaagggtggcccctgcccttcgcct
gggacatcctgtcccctcagttcatgtacggctccaaggcctacgtgaagcaccccgccgacatccccgactacttgaagctgtccttccccgagggct
tcaagtgggagcgcgtgatgaacttcgaggacggcggcgtggtgaccgtgacccaggactcctccctgcaggacggcgagttcatctacaaggtgaa
gctgcgcggcaccaacttcccctccgacggccccgtaatgcagaagaagaccatgggctgggaggcctcctccgagcggatgtaccccgaggacggc
gccctgaagggcgagatcaagcagaggctgaagctgaaggacggcggccactacgacgctgaggtcaagaccacctacaaggccaagaagcccgt
gcagctgcccggcgcctacaacgtcaacatcaagttggacatcacctcccacaacgaggactacaccatcgtggaacagtacgaacgcgccgaggg
ccgccactccaccggcggcatggacgagctgtacaagCATGGCATGGATGAGCTCTACAAAGGTGGAGGTCGGACCGAAGAG

TACAAGCTTATCCTGAACGGTAAAACCCTGAAAGGTGAAACCACCACCGAAGCTGTTGACGCTGCTACCGCGGAA
AAAGTTTTCAAACAGTACGCTAACGACAACGGTGTTGACGGTGAATGGACCTACGACGACGCTACCAAAACCTTC
ACGGTAACCGAAGCTAGTGGTGGTGGTGGTAGCGGTGGTGGTAGCagcttaccggtcgccGAATTCGGTgtgagcaaggg
cgaggaggataacatggccatcatcaaggagttcatgcgcttcaaggtgcacatggagggctccgtgaacggccacgagttcgagatcgagggcga
gggcgagggccgcccctacgagggcacccagaccgccaagctgaaggtgaccaagggtggccccctgcccttcgcctgggacatcctgtcccctcag
ttcatgtacggctccaaggcctacgtgaagcaccccgccgacatccccgactacttgaagctgtccttccccgagggcttcaagtgggagcgcgtgatg
aacttcgaggacggcggcgtggtgaccgtgacccaggactcctccctgcaggacggcgagttcatctacaaggtgaagctgcgcggcaccaacttcc
cctccgacggccccgtaatgcagaagaagaccatgggctgggaggcctcctccgagcggatgtaccccgaggacggcgccctgaagggcgagatca
agcagaggctgaagctgaaggacggcggccactacgacgctgaggtcaagaccacctacaaggccaagaagcccgtgcagctgcccggcgcctac
aacgtcaacatcaagttggacatcacctcccacaacgaggactacaccatcgtggaacagtacgaacgcgccgagggccgccactccaccggcggc
atggacgagctgtacaagCATATGGGTGGAGGTTCTGGTGGTGGATCTGGTGGAGGTTCTGGAGGTGGAAAAATGTCC
AAGGATGGTAAGAAAAAGAAGAAGTCAAAAACCAAGTGTGTTATCATGtaa

BFP-Kif18b-PP7$_{24x}$
gacggatcgggagatctcccgatcccctatggtgcactctcagtacaatctgctctgatgccgcatagttaagccagtatctgctccctgcttgtgtgttg
gaggtcgctgagtagtgcgcgagcaaaatttaagctacaacaaggcaaggcttgaccgacaattgcatgaagaatctgcttagggttaggcgttttgc
gctgcttcgcgatgtacgggccagatatacgcgttgacattgattattgactagttattaatagtaatcaattacggggtcattagttcatagcccatata
tggagttccgcgttacataacttacggtaaatggcccgcctggctgaccgcccaacgacccccgcccattgacgtcaataatgacgtatgttcccatag
taacgccaatagggactttccattgacgtcaatgggtggagtatttacggtaaactgcccacttggcagtacatcaagtgtatcatatgccaagtacgc
cccctattgacgtcaatgacggtaaatggcccgcctggcattatgcccagtacatgaccttatgggactttcctacttggcagtacatctacgtattagtc
atcgctattaccatggtgatgcggttttggcagtacatcaatgggcgtggatagcggtttgactcacggggatttccaagtctccaccccattgacgtca
ATGGGAGTTTGTTTTGGCACCAAAATCAACGGGACTTTCCAAAATGTCGTAACAACTCCGCCCCATTGAcgcaaatgg
gcggtaggcgtgtacggtgggaggtctatataagcagagctctccctatcagtgatagagatctccctatcagtgatagagatcgtcgacgagctcgtt
tagtgaaccgtcagatcgcctggagacgccatccacgctgttttgacctccatagaagacaccgggaccgatccagcctccggactctagcgtttaaa
cttAAGCTTGCCACCATGGGCGCCATCATACACGCGGCCGCAATCATcggtccgagcgagctgattaaggagaacatgcacatga
agctAtacatggagggcaccgtggacaaccatcacttcaagtgcacatccgagggcgaaggcaagccctacgagggcacccagaccatgagaatc
aaggtggtcgagggcggccctctccccttcgccttcgacatcctggctactagcttcctctacggcagcaagaccttcatcaaccacacccagggcatc
cccgacttcttcaagcagtccttccctgagggcttcacatgggagagagtcaccacatacgaagacgggggcgtgctgaccgctacccaggacacca
gcctccaggacggctgcctcatctacaacgtcaagatcagaggggtgaacttcacatccaacggccctgtgatgcagaagaaaacactcggctggga
ggccttcaccgagacgctgtaccccgctgacggcggcctggaaggcagaaacgacatggccctgaagctcgtgggcgggagccatctgatcgcaaa
catcaagaccacatatagatccaagaaacccgctaagaacctcaagatgcctggcgtctactatgtggactacagactggaaagaatcaaggaggc
caacaacgagacctacgtcgagcagcacgaggtggcagtggccagatactgcgacctccctagcaaactggggcacaaacttaatcACCGGTCA
GCAGTGGAGGACAGCACGCTGCAAGTAGTGGTACGGGTGCGGCCCCCCACCCCTCGGGAGCTGGACAGTCAGCG
GCGGCCAGTGGTTCAGGTGGTGGACGAGCGGGTGCTGGTGTTTAACCCTGAGGAGCCCGATGGAGGGTTCCCTG
GCCTGAAATGGGGTGGCACCCATGATGGCCCCAAGAAGAAGGGCAAAGACCTGACGTTTGTCTTTGACCGGGTCT
TTGGCGAGGCGGCCACCCAACAGGACGTGTTCCAGCACACCACGCACAGCGTCCTGGACAGCTTCCTCCAGGGCT
ACAACTGCTCAGTGTTTGCCTACGGGGCCACCGGGGCTGGGAAGACACACACCATGCTGGGAAGGGAGGGGGAC
CCCGGCATCATGTACCTGACCACCGTGGAACTGTACAGGCGCCTGGAGGCCCGCCAGCAGGAGAAGCACTTCGA
GGTGCTCATCAGCTACCAGGAGGTGTATAATGAACAGATCCATGACCTCCTGGAGCCCAAGGGGCCCCTTGCCAT
CCGCGAGGACCCCGACAAGGGGGTGGTGGTGCAAGGACTTTCTTTCCACCAGCCAGCCTCAGCCGAGCAGCTGCT
GGAGATACTGACCAGGGGGAACCGTAACCGCACGCAGCACCCCACTGATGCCAACGCGACTTCCTCCCGCTCCCA
TGCCATCTTCCAGATCTTTGTGAAGCAGCAGGACCGGGTTCCAGGACTGACCCAGGCTGTCCAGGTGGCCAAGAT
GAGCCTGATTGACCTGGCTGGCTCAGAGCGGGCATCCAGCACCCATGCGAAGGGGGAGCGGCTGCGGGAGGGG
GCCAACATCAACCGCTCTCTGCTGGCGCTCATCAACGTCCTCAATGCCTTGGCCGATGCAAAGGGCCGCAAGACCG
CTGTGCCCTACGCGGACAGCGCACTGACCCGCCTGCTCAAAGACTCCCTCGGGGGCAACTGCCGCACAGTGATGA
TCGCTGCCATCAGCCCCTCCAGCCTGACCTACGAGGACACGTATAATACCCTCAAATATGCCGACCGGGCCAAGGA
GATCAGGCTCTCGCTGAAGAGCAATGTGACCAGCCTGGACTGTCACATCAGCCAGTATGCTACCATCTGCCAACAG

CTCCAGGCTGAGGTAGCCGCTCTGAGGAAGAAGCTCCAAGTGTATGAGGGGGGAGGCCAGCCCCCACCACAGGA
CCTCCCAGGATCTCCCAAGTCGGGACCACCACCAGAACACCTTCCCAGCTCCCCCTTGCCACCCCACCCTCCCAGCC
AGCCCTGCACCCCAGAGCTCCCTGCAGGGCCTAGAGCCCTTCAAGAGGAGAGTCTGGGGATGGAGGCCCAGGTG
GAGAGGGCCATGGAAGGGAACTCTTCAGACCAGGAGCAGTCCCCAGAGGATGAGGATGAAGGCCCAGCTGAGG
AGGTTCCAACCCAGATGCCAGAGCAGAACCCCACACATGCACTGCCAGAGTCCCCTCGCCTGACCCTGCAGCCCAA
GCCAGTCGTGGGCCACTTCTCAGCACGGGAACTGGATGGGGACCGTTCTAAGCGGTTGGCCCTAAAGGTGCTGTG
CGTTGCCCAGCGGCAGTACTCCCTGCTCCAAGCAGCCAACCTCCTGACGCCCGACATGATCACAGAGTTTGAGACC
CTACAGCAGCTGGTGCAAGAGGAAAAAATTGAGCCTGGGGCAGAGGCCTTGAGGACTTCAGGCCTGGCCAGGG
GGGCACCTCTGGCTCAGGAGCTGTGTTCAGAGTCAAAGCCTCCAGGATACACTGGCCCTGTGACCCGGACTATGG
CGAGGCGACTGAGTGGCCCCCTGCACACCCTGGGAATCCCGCCTGGACCCAACTGCACCCCAGCCCAGGGGTCCC
GATGGCCCATGGAGAAGAAGAGGAGGAGACCAAGCGCCTTGGAGGCAGACAGTCCCATGGCCCCAAAGCGGGG
CACCAAGCGCCAGCGCCAGTCCTTCCTGCCCTGCCTAAGGAGAGGGTCTCTGCCTGACACCCAACCTTCACAGGGG
CCCAGCACCCCCAAAGGAGAAAGGGCCTCCTCCCCCTGCCATTCCCCTCGCGTTTGCCCAGCCACAGTCATCAAAA
GCCGGGTGCCCCTGGGCCCTTCCGCCATGCAGAACTGCTCCACCCCGCTGGCTCTGCCCACTCGAGACCTCAATGC
CACCTTTGATCTCTCTGAGGAGCCTCCCTCAAAGCCCAGTTTCCATGAATGCATTGGCTGGGACAAAATACCCCAG
GAGCTGAGCAGGCTGGACCAGCCCTTCATCCCCAGGGCACCTGTGCCCCTGTTCACCATGAAGGGCCCCAAGCCA
ACATCTTCCCTCCCTGGGACCTCTGCCTGCAAGAAGAAGCGCGTTGCGAGTTCCTCAGTCTCCCATGGCCGCAGCC
GCATCGCCCGCCTCCCCAGCAGCACTTTGAAGAGGCCAGCTGGGCCCCTTGTACTCCCAGAGCTGCCCTTGAGTCC
CCTGTGCCCTAGCAACCGGAGGAATGGAAAGGACCTCATCAGGGTGGGGAGAGCACTCTCAGCAGGGAACGGCG
TCACCAAGGTGTCCGATATCTAACGACACGATTCGAAGATCGCATCGCTAGCCCGGTTGTACAGGTACCGGATCCT
AAGGTACCTAATTGCCTAGAAAGGAGCAGACGATATGGCGTCGCTCCCTGCAGGTCGACTCTAGAAACCAGCAGA
GCATATGGGCTCGCTGGCTGCAGTATTCCCGGGTTCATTAGATCCTAAGGTACCTAATTGCCTAGAAAGGAGCAG
ACGATATGGCGTCGCTCCCTGCAGGTCGACTCTAGAAACCAGCAGAGCATATGGGCTCGCTGGCTGCAGTATTCC
CGGGTTCATTAGATCCTAAGGTACCTAATTGCCTAGAAAGGAGCAGACGATATGGCGTCGCTCCCTGCAGGTCGA
CTCTAGAAACCAGCAGAGCATATGGGCTCGCTGGCTGCAGTATTCCCGGGTTCATTAGATCCTAAGGTACCTAATT
GCCTAGAAAGGAGCAGACGATATGGCGTCGCTCCCTGCAGGTCGACTCTAGAAACCAGCAGAGCATATGGGCTC
GCTGGCTGCAGTATTCCCGGGTTCATTAGATCCTAAGGTACCTAATTGCCTAGAAAGGAGCAGACGATATGGCGT
CGCTCCCTGCAGGTCGACTCTAGAAACCAGCAGAGCATATGGGCTCGCTGGCTGCAGTATTCCCGGGTTCATTAGA
TCCTAAGGTACCTAATTGCCTAGAAAGGAGCAGACGATATGGCGTCGCTCCCTGCAGGTCGACTCTAGAAACCAG
CAGAGCATATGGGCTCGCTGGCTGCAGTATTCCCGGGTTCATTAGATCCTAAGGTACCTAATTGCCTAGAAAGGA
GCAGACGATATGGCGTCGCTCCCTGCAGGTCGACTCTAGAAACCAGCAGAGCATATGGGCTCGCTGGCTGCAGTA
TTCCCGGGTTCATTAGATCCTAAGGTACCTAATTGCCTAGAAAGGAGCAGACGATATGGCGTCGCTCCCTGCAGGT
CGACTCTAGAAACCAGCAGAGCATATGGGCTCGCTGGCTGCAGTATTCCCGGGTTCATTAGATCCTAAGGTACCTA
ATTGCCTAGAAAGGAGCAGACGATATGGCGTCGCTCCCTGCAGGTCGACTCTAGAAACCAGCAGAGCATATGGGC
TCGCTGGCTGCAGTATTCCCGGGTTCATTAGATCCTAAGGTACCTAATTGCCTAGAAAGGAGCAGACGATATGGC
GTCGCTCCCTGCAGGTCGACTCTAGAAACCAGCAGAGCATATGGGCTCGCTGGCTGCAGTATTCCCGGGTTCATTA
GATCCTAAGGTACCTAATTGCCTAGAAAGGAGCAGACGATATGGCGTCGCTCCCTGCAGGTCGACTCTAGAAACC
AGCAGAGCATATGGGCTCGCTGGCTGCAGTATTCCCGGGTTCATTAGATCCTAAGGTACCTAATTGCCTAGAAAG
GAGCAGACGATATGGCGTCGCTCCCTGCAGGTCGACTCTAGAAACCAGCAGAGCATATGGGCTCGCTGGCTGCAG
TATTCCCGGGTTCATTAGATCTcgcgAAGGGCGAATTCacgaGGCGCGCCggatcGCGATCGCaatcTTAATTAACTCG
AGtctagagggcccgtttaaacccgctgatcagcctcgactgtgccttctagttgccagccatctgttgtttgcccctcccccgtgccttccttgaccctg
gaaggtgccactcccactgtcctttcctaataaaatgaggaaattgcatcgcattgtctgagtaggtgtcattctattctggggggtggggtggggcagg
acagcaagggggaggattgggaagacaatagcaggcatgctggggatgcggtgggctctatggcttctgaggcggaaagaaccagctggggctcta
gggggtatccccacgcgccctgtagcggcgcattaagcgcggcgggtgtggtggttacgcgcagcgtgaccgctacacttgccagcgccctagcgccc
gctcctttcgctttcttcccttcctttctcgccacgttcgccggctttccccgtcaagctctaaatcgggggctccctttagggttccgatttagtgctttacg
gcacctcgaccccaaaaaacttgattagggtgatggttcacgtagtgggccatcgccctgatagacggtttttcgccctttgacgttggagtccacgttc
tttaatagtggactcttgttccaaactggaacaacactcaaccctatctcggtctattcttttgatttataagggattttgccgatttcggcctattggttaa
aaaatgagctgatttaacaaaaatttaacgcgaattaattctgtggAATGTGTGTCAGTTAGGGTGTGGAAAGTCCCCAGGCTCCC

CAGCAGGCAGAAGTATGCAAAGCATGCATCTCAATTAGTCAGCAACCAGGTGTGGAAAGTCCCCAGGCTCCCCAG
CAGGCAGAAGTATGCAAAGCATGCATCTCAATTAGTCAGCAACCATAGTCCCGCCCCTAACTCCGCCCATCCCGCC
CCTAACTCCGCCCAGTTCCGCCCATTCTCCGCCCCATGGCTGACTAATTTTTTTTATTTATGCAGAGGCCGAGGCCG
CCTCTGCCTCTGAGCTATTCCAGAAGTAGTGAGGAGGCTTTTTTGGAGGCCTAGGCTTTTGCAAAAAGCTCCcggga
gcttgtatatccattttcggatctgatcagcacgtgttgacaattaatcatcggcatagtatatcggcatagtataatacgacaaggtgaggaactaaac
catggccaagttgaccagtgccgttccggtgctcaccgcgcgcgacgtcgccggagcggtcgagttctggaccgaccggctcgggttctcccgggactt
cgtggaggacgacttcgccggtgtggtccgggacgacgtgaccctgttcatcagcgcggtccaggaccaggtggtgccggacaacaccctggcctgg
gtgtgggtgcgcggcctggacgagctgtacgccgagtggtcggaggtcgtgtccacgaacttccgggacgcctccgggccggccatgaccgagatcg
gcgagcagccgtgggggcgggagttcgccctgcgcgacccggccggcaactgcgtgcacttcgtggccgaggagcaggactgacacgtgctacgag
atttcgattccaccgccgccttctatgaaaggttgggcttcggaatcgttttccgggacgccggctggatgatcctccagcgcggggatctcatgctgga
gttcttcgcccaccccaacttgtttattgcagcttataatggttacaaataaagcaatagcatcacaaatttcacaaataaagcatttttttcactgcattc
tagttgtggtttgtccaaactcatcaatgtatcttatcatgtctgtataccgtcgacctctagctagagcttggcgtaatcatggtcatagctgtttcctgtg
tgaaattgttatccgctcacaattccacacaacatacgagccggaagcataaagtgtaaagcctggggtgcctaatgagtgagctaactcacattaat
tgcgttgcgctcactgcccgctttccagtcgggaaacctgtcgtgccagctgcattaatgaatcggccaacgcgcggggagaggcggtttgcgtattgg
gcgctcttccgcttcctcgctcactgactcgctgcgctcggtcgttcggctgcggcgagcggtatcagctcactcaaaggcggtaatacggttatccaca
gaatcaggggataacgcaggaaagaacatgtgagcaaaaggccagcaaaaggccaggaaccgtaaaaaggccgcgttgctggcgtttttccatag
gctccgcccccctgacgagcatcacaaaaatcgacgctcaagtcagaggtggcgaaacccgacaggactataaagataccaggcgtttccccctgga
agctccctcgtgcgctctcctgttccgaccctgccgcttaccggatacctgtccgcctttctcccttcgggaagcgtggcgctttctcatagctcacgctgt
aggtatctcagttcggtgtaggtcgttcgctccaagctgggctgtgtgcacgaaccccccgttcagcccgaccgctgcgccttatccggtaactatcgtc
ttgagtccaacccggtaagacacgacttatcgccactggcagcagccactggtaacaggattagcagagcgaggtatgtaggcggtgctacagagtt
cttgaagtggtggcctaactacggctacactagaagaacagtatttggtatctgcgctctgctgaagccagttaccttcggaaaaagagttggtagctc
ttgatccggcaaacaaaccaccgctggtagcggtttttttgtttgcaagcagcagattacgcgcagaaaaaaaggatctcaagaagatcctttgatctt
ttctacggggtctgacgctcagtggaacgaaaactcacgttaagggattttggtcatgagattatcaaaaaggatcttcacctagatccttttaaattaa
aaatgaagttttaaatcaatctaaagtatatatgagtaaacttggtctgacagttaccaatgcttaatcagtgaggcacctatctcagcgatctgtctatt
tcgttcatccatagttgcctgactccccgtcgtgtagataactacgatacgggagggcttaccatctggccccagtgctgcaatgataccgcgagaccc
acgctcaccggctccagatttatcagcaataaaccagccagccggaagggccgagcgcagaagtggtcctgcaactttatccgcctccatccagtcta
ttaattgttgccgggaagctagagtaagtagttcgccagttaatagtttgcgcaacgttgttgccattgctacaggcatcgtggtgtcacgctcgtcgttt
ggtatggcttcattcagctccggttcccaacgatcaaggcgagttacatgatcccccatgttgtgcaaaaaagcggttagctccttcggtcctccgatcg
ttgtcagaagtaagttggccgcagtgttatcactcatggttatggcagcactgcataattctcttactgtcatgccatccgtaagatgcttttctgtgactg
gtgagtactcaaccaagtcattctgagaatagtgtatgcggcgaccgagttgctcttgcccggcgtcaatacgggataataccgcgccacatagcaga
actttaaaagtgctcatcattggaaaacgttcttcggggcgaaaactctcaaggatcttaccgctgttgagatccagttcgatgtaacccactcgtgcac
ccaactgatcttcagcatcttttactttcaccagcgtttctgggtgagcaaaaacaggaaggcaaaatgccgcaaaaaagggaataagggcgacacg
gaaatgttgaatactcatactcttcctttttcaatattattgaagcatttatcagggttattgtctcatgagcggatacatatttgaatgtatttagaaaaa
taaacaaataggggttccgcgcacatttccccgaaaagtgccacctgacgtc

tdPP7-3xmCherry
tgtcacctaaatcgtatgtgtatgatacataaggttatgtattaattgtagccgcgttctaacgacaatatgtacaagcctaattgtgtagcatctggctt
actgaagcagaccctatcatctctctcgtaaactgccgtcagagtcggtttggttggacgaaccttctgagtttctggtaacgccgtcccgcacccggaa
atggtcagcgaaccaatcagcagggtcatcgctagccagatcctctacgccggacgcatcgtggccggcatcaccggcgccacaggtgcggttgctg
gcgcctatatcgccgacatcaccgatggggaagatcgggctcgccacttcgggctcatgagcgcttgtttcggcgtgggtatggtggcaggccccgtgg
ccgggggactgttgggcgccatctccttgcatgcaccattccttgcggcggcggtgctcaacggcctcaacctactactgggctgcttcctaatgcagga
gtcgcataagggagagcgtcgaatggtgcactctcagtacaatctagctctgatgccgcatagttaagccagccccgacacccgccaacacccgctga
cgcgccctgacgggcttgtctgctcccggcatccgcttacagacaagctgtgaccgtctccgggagctgcatgtgtcagaggttttcaccgtcatcaccg
aaacgcgcgagacgaaagggcctcgtgatacgcctatttttataggttaatgtcatgataataatggtttcttagacgtcaggtggcacttttcggggaa
atgtgcgcggaacccctatttgtttatttttctaaatacattcaaatatgtatccgctcatgagacaataaccctgataaatgcttcaataatattgaaaa
aggaagagtatgagtattcaacatttccgtgtcgcccttattcccttttttgcggcattttgccttcctgtttttgctcacccagaaacgctggtgaaagta
aaagatgctgaagatcagttgggtgcacgagtgggttacatcgaactggatctcaacagcggtaagatccttgagagttttcgccccgaagaacgtttt

ccaatgatgagcacttttaaagttctgctatgtggcgcggtattatcccgtattgacgccgggcaagagcaactcggtcgccgcatacactattctcaga
atgacttggttgagtactcaccagtcacagaaaagcatcttacggatggcatgacagtaagagaattatgcagtgctgccataaccatgagtgataac
actgcggccaacttacttctgacaacgatcggaggaccgaaggagctaaccgcttttttgcacaacatgggggatcatgtaactcgccttgatcgttgg
gaaccggagctgaatgaagccataccaaacgacgagcgtgacaccacgatgcctgtagcaatggcaacaacgttgcgcaaactattaactggcgaa
ctacttactctagcttcccggcaacaattaatagactggatggaggcggataaagttgcaggaccacttctgcgctcggcccttccggctggctggttta
ttgctgataaatctggagccggtgagcgtgggtctcgcggtatcattgcagcactggggccagatggtaagccctcccgtatcgtagttatctacacga
cggggagtcaggcaactatggatgaacgaaatagacagatcgctgagataggtgcctcactgattaagcattggtaactgtcagaccaagtttactca
tatatactttagattgatttaaaacttcatttttaatttaaaaggatctaggtgaagatcctttttgataatctcatgaccaaaatcccttaacgtgagtttt
cgttccactgagcgtcagaccccgtagaaaagatcaaaggatcttcttgagatcctttttttctgcgcgtaatctgctgcttgcaaacaaaaaaaccacc
gctaccagcggtggtttgtttgccggatcaagagctaccaactctttttccgaaggtaactggcttcagcagagcgcagataccaaatactgtccttcta
gtgtagccgtagttaggccaccacttcaagaactctgtagcaccgcctacatacctcgctctgctaatcctgttaccagtggctgctgccagtggcgata
agtcgtgtcttaccgggttggactcaagacgatagttaccggataaggcgcagcggtcgggctgaacggggggttcgtgcacacagcccagcttgga
gcgaacgacctacaccgaactgagatacctacagcgtgagctatgagaaagcgccacgcttcccgaagggagaaaggcggacaggtatccggtaa
gcggcagggtcggaacaggagagcgcacgagggagcttccagggggaaacgcctggtatctttatagtcctgtcgggtttcgccacctctgacttgag
cgtcgatttttgtgatgctcgtcaggggggcggagcctatggaaaaacgccagcaacgcggcctttttacggttcctggccttttgctggccttttgctca
catgttctttcctgcgttatcccctgattctgtggataaccgtattaccgcctttgagtgagctgataccgctcgccgcagccgaacgaccgagcgcagc
gagtcagtgagcgaggaagcggaagagcgcccaatacgcaaaccgcctctccccgcgcgttggccgattcattaatgcagctgtggaatgtgtgtca
gttagggtgtggaaagtccccaggctccccagcaggcagaagtatgcaaagcatgcatctcaattagtcagcaaccaggtgtggaaagtccccaggc
tccccagcaggcagaagtatgcaaagcatgcatctcaattagtcagcaaccatagtcccgcccctaactccgcccatcccgcccctaactccgcccagt
tccgcccattctccgccccatggctgactaattttttttatttatgcagaggccgaggccgcctcggcctctgagctattccagaagtagtgaggaggctt
ttttggaggcctaggcttttgcaaaaagcttggacacaagacaggcttgcgagatatgtttgagaataccactttatcccgcgtcagggagaggcagtg
cgtaaaaagacgcggactcatgtgaaatactggtttttagtgcgccagatctctataatctcgcgcaacctattttcccctcgaacactttttaagccgta
gataaacaggctgggacacttcacatgagcgaaaaatacatcgtcacctgggacatgttgcagatccatgcacgtaaactcgcaagccgactgatgc
cttctgaacaatggaaaggcattattgccgtaagccgtggcggtctgtaccgggtgcgttactggcgcgtgaactgggtattcgtcatgtcgataccgtt
tgtatttccagctacgatcacgacaaccagcgcgagcttaaagtgctgaaacgcgcagaaggcgatggcgaaggcttcatcgttattgatgacctggt
ggataccggtggtactgcggttgcgattcgtgaaatgtatccaaaagcgcactttgtcaccatcttcgcaaaaccggctggtcgtccgctggttgatgac
tatgttgttgatatcccgcaagatacctggattgaacagccgtgggatatgggcgtcgtattcgtcccgccaatctccggtcgctaatcttttcaacgcct
ggcactgccgggcgttgttctttttaacttcaggcgggttacaatagtttccagtaagtattctggaggctgcatccatgacacaggcaaacctgagcga
aaccctgttcaaaccccgctttaaacatcctgaaacctcgacgctagtccgccgctttaatcacggcgcacaaccgcctgtgcagtcggcccttgatggt
aaaaccatccctcactggtatcgcatgattaaccgtctgatgtggatctggcgcggcattgacccacgcgaaatcctcgacgtccaggcacgtattgtg
atgagcgatgccgaacgtaccgacgatgatttatacgatacggtgattggctaccgtggcggcaactggatttatgagtgggccccggatctttgtgaa
ggaaccttacttctgtggtgtgacataattggacaaactacctacagagatttaaagctctaaggtaaatataaaatttttaagtgtataatgtgttaaa
ctactgattctaattgtttgtgtattttagattccaacctatggaactgatgaatgggagcagtggtggaatgcctttaatgaggaaaacctgttttgctc
agaagaaatgccatctagtgatgatgaggctactgctgactctcaacattctactcctccaaaaaagaagagaaaggtagaagaccccaaggacttt
ccttcagaattgctaagttttttgagtcatgctgtgtttagtaatagaactcttgcttgctttgctatttacaccacaaaggaaaaagctgcactgctatac
aagaaaattatggaaaaatattctgtaacctttataagtaggcataacagttataatcataacatactgttttttcttactccacacaggcatagagtgt
ctgctattaataactatgctcaaaaattgtgtacctttagctttttaatttgtaaaggggttaataaggaatatttgatgtatagtgccttgactagagatc
ataatcagccataccacatttgtagaggttttacttgctttaaaaaacctcccacacctccccctgaacctgaaacataaaatgaatgcaattgttgttg
ttaacttgtttattgcagcttataatggttacaaataaagcaatagcatcacaaatttcacaaataaagcatttttttcactgcattctagttgtggtttgt
ccaaactcatcaatgtatcttatcatgtctggatcaactggataactcaagctaaccaaaatcatcccaaacttcccaccccataccctattaccactgc
caattacctagtggtttcatttactctaaacctgtgattcctctgaattattttcattttaaagaaattgtatttgttaaatatgtactacaaacttagtagtt
ggaagggctaattcactcccaaagaagacaagatatccttgatctgtggatctaccacacacaaggctacttccctgattagcagaactacacaccag
ggccaggggtcagatatccactgacctttggatggtgctacaagctagtaccagttgagccagataaggtagaagaggccaataaaggagagaaca
ccagcttgttacaccctgtgagcctgcatgggatggatgacccggagagagaagtgttagagtggaggtttgacagccgcctagcatttcatcacgtg
gcccgagagctgcatccggagtacttcaagaactgctgatatcgagcttgctacaagggactttccgctggggactttccagggaggcgtggcctggg
cgggactggggagtggcgagccctcagatcctgcatataagcagctgctttttgcctgtactgggtctctctggttagaccagatctgagcctgggagct
ctctggctaactagggaacccactgcttaagcctcaataaagcttgccttgagtgcttcaagtagtgtgtgcccgtctgttgtgtgactctggtaactaga
gatccctcagacccttttagtcagtgtggaaaatctctagcagtggcgcccgaacagggacttgaaagcgaaagggaaaccagaggagctctctcga

cgcaggactcggcttgctgaagcgcgcacggcaagaggcgaggggcggcgactggtgagtacgccaaaaattttgactagcggaggctagaagga
gagagatgggtgcgagagcgtcagtattaagcgggggagaattagatcgcgatgggaaaaaattcggttaaggccagggggaaagaaaaaatata
aattaaaacatatagtatgggcaagcagggagctagaacgattcgcagttaatcctggcctgttagaaacatcagaaggctgtagacaaatactggg
acagctacaaccatcccttcagacaggatcagaagaacttagatcattatataatacagtagcaaccctctattgtgtgcatcaaaggatagagataa
aagacaccaaggaagctttagacaagatagaggaagagcaaaacaaaagtaagaccaccgcacagcaagcggccggtgatcttcagacctggac
gatatatatgagggacaattggagaagtgaattatataaatataaagtagtaaaaattgaaccattaggagtagcacccaccaaggcaaagagaag
agtggtgcagagagaaaaaagagcagtgggaataggagctttgttccttgggttcttgggagcagcaggaagcactatgggcgcagcgtcaatgac
gctgacggtacaggccagacaattattgtctggtatagtgcagcagcagaacaatttgctgagggctattgaggcgcaacagcatctgttgcaactca
cagtctggggcatcaagcagctccaggcaagaatcctggctgtggaaagatacctaaaggatcaacagctcctggggatttggggttgctctggaaa
actcatttgcaccactgctgtgccttggaatgctagttggagtaataaatctctggaacagatttggaatcacacgacctggatggagtgggacagaga
aattaacaattacacaagcttaatacactccttaattgaagaatcgcaaaaccagcaagaaaagaatgaacaagaattattggaattagataaatgg
gcaagtttgtggaattggtttaacataacaaattggctgtggtatataaaattattcataatgatagtaggaggcttggtaggtttaagaatagtttttgc
tgtactttctatagtgaatagagttaggcagggatattcaccattatcgtttcagacccacctcccaaccccgaggggacccgacaggcccgaaggaat
agaagaagaaggtggagagagagacagagacagatccattcgattagtgaacggatctcgacggtATcgccaaatggcagtattcatccacaattt
taaaagaaaaggggggattggggggtacagtgcaggggaaagaatagtagacataatagcaacagacatacaaactaaagaattacaaaaacaa
attacaaaaattcaaaattttcgggtttattacagggacagcagagatccagtttggatcgataagcttgatatcgaattcctgcagccccgataaaat
aaaagattttatttagtctccagaaaaaggggggaatgaaagaccccacctgtaggtttggcaagctagctgcagtaacgccattttgcaaggcatgg
aaaaataccaaaccaagaatagagaagttcagatcaagggcgggtacatgaaaatagctaacgttgggccaaacaggatatctgcggtgagcagtt
tcggccccggcccggggccaagaacagatggtcaccgcagtttcggccccggcccgaggccaagaacagatggtccccagatatggcccaaccctc
agcagtttcttaagacccatcagatgtttccaggctcccccaaggacctgaaatgaccctgcgccttatttgaattaaccaatcagcctgcttctcgcttc
tgttcgcgcgcttctgcttcccgagctctataaaagagctcacaacccctcactcggcgcgccagtcctccgacagactgagtcgcccgggggggatct
ggagctctcgagaattctcAcgcgccGGATCCGCCACCATGTCCAAAACCATCGTTCTTTCGGTCGGCGAGGCTACTCGCAC
TCTGACTGAGATCCAGTCCACCGCAGACCGTCAGATCTTCGAAGAGAAGGTCGGGCCTCTGGTGGGTCGGCTGCG
CCTCACGGCTTCGCTCCGTCAAAACGGAGCCAAGACCGCGTATCGCGTCAACCTAAAACTGGATCAGGCGGACGT
CGTTGATTGCTCCACCAGCGTCTGCGGCGAGCTTCCGAAAGTGCGCTACACTCAGGTATGGTCGCACGACGTGAC
AATCGTTGCGAATAGCACCGAGGCCTCGCGCAAATCGTTGTACGATTTGACCAAGTCCCTCGTCGCGACCTCGCAG
GTCGAAGATCTTGTCGTCAACCTTGTGCCGCTGGGCCGTCGTGCGGACCCGCTAGCCTCCTgcggccgcTCCAAAACC
ATCGTTCTTTCGGTCGGCGAGGCTACTCGCACTCTGACTGAGATCCAGTCCACCGCAGACCGTCAGATCTTCGAAG
AGAAGGTCGGGCCTCTGGTGGGTCGGCTGCGCCTCACGGCTTCGCTCCGTCAAAACGGAGCCAAGACCGCGTATC
GCGTCAACCTAAAACTGGATCAGGCGGACGTCGTTGATTGCTCCACCAGCGTCTGCGGCGAGCTTCCGAAAGTGC
GCTACACTCAGGTATGGTCGCACGACGTGACAATCGTTGCGAATAGCACCGAGGCCTCGCGCAAATCGTTGTACG
ATTTGACCAAGTCCCTCGTCGCGACCTCGCAGGTCGAAGATCTTGTCGTCAACCTTGTGCCGCTGGGCCGTCGTGC
GGACCCGCTAGCCTCCACGCGTGATTCGACTAGTGGAGGAAGCGGAGGAGGAgtgagcaagggcgaggaggataacatg
gccatcatcaaggagttcatgcgcttcaaggtgcacatggagggctccgtgaacggccacgagttcgagatcgagggcgagggcgagggccgcccct
acgagggcacccagaccgccaagctgaaggtgaccaagggtggcccccctgcccttcgcctgggacatcctgtcccctcagttcatgtacggctccaag
gcctacgtgaagcaccccgccgacatccccgactacttgaagctgtccttccccgagggcttcaagtgggagcgcgtgatgaacttcgaggacggcgg
cgtggtgaccgtgacccaggactcctccctgcaggacggcgagttcatctacaaggtgaagctgcgcggcaccaacttcccctccgacggccccgtaa
tgcagaagaagaccatgggctgggaggcctcctccgagcggatgtaccccgaggacggcgccctgaagggcgagatcaagcagaggctgaagctg
aaggacggcggccactacgacgctgaggtcaagaccacctacaaggccaagaagcccgtgcagctgcccggcgcctacaacgtcaacatcaagttg
gacatcacctcccacaacgaggactacaccatcgtggaacagtacgaacgcgccgagggccgccactccaccggcggcatggacgagctgtacaag
CATGGCATGGATGAGCTCTACAAAGGTGGAGGTCGGACCGAAGAGTACAAGCTTATCCTGAACGGTAAAACCCTG
AAAGGTGAAACCACCACCGAAGCTGTTGACGCTGCTACCGCGGAAAAAGTTTTCAAACAGTACGCTAACGACAAC
GGTGTTGACGGTGAATGGACCTACGACGACGCTACCAAAACCTTCACGGTAACCGAAGCTAGTGGAGGAAGCGG
AGGAGGAgtgagcaagggcgaggaggataacatggccatcatcaaggagttcatgcgcttcaaggtgcacatggagggctccgtgaacggccac
gagttcgagatcgagggcgagggcgagggccgcccctacgagggcacccagaccgccaagctgaaggtgaccaagggtggcccccctgcccttcgcc
tgggacatcctgtcccctcagttcatgtacggctccaaggcctacgtgaagcaccccgccgacatccccgactacttgaagctgtccttccccgagggc
ttcaagtgggagcgcgtgatgaacttcgaggacggcggcgtggtgaccgtgacccaggactcctccctgcaggacggcgagttcatctacaaggtga
agctgcgcggcaccaacttcccctccgacggccccgtaatgcagaagaagaccatgggctgggaggcctcctccgagcggatgtaccccgaggacg

gcgccctgaagggcgagatcaagcagaggctgaagctgaaggacggcggccactacgacgctgaggtcaagaccacctacaaggccaagaagccc
gtgcagctgcccggcgcctacaacgtcaacatcaagttggacatcacctcccacaacgaggactacaccatcgtggaacagtacgaacgcgccgagg
gccgccactccaccggcggcatggacgagctgtacaagCATGGCATGGATGAGCTCTACAAAGGTGGAGGTCGGACCGAAGA
GTACAAGCTTATCCTGAACGGTAAAACCCTGAAAGGTGAAACCACCACCGAAGCTGTTGACGCTGCTACCGCGGA
AAAAGTTTTCAAACAGTACGCTAACGACAACGGTGTTGACGGTGAATGGACCTACGACGACGCTACCAAAACCTT
CACGGTAACCGAAGCTAGTGGTGGTGGTGGTAGCGGTGGTGGTAGCagcttaccggtcgccGAATTCGGTgtgagcaagg
gcgaggaggataacatggccatcatcaaggagttcatgcgcttcaaggtgcacatggagggctccgtgaacggccacgagttcgagatcgagggcg
agggcgagggccgcccctacgagggcacccagaccgccaagctgaaggtgaccaagggtggcccccctgcccttcgcctgggacatcctgtcccctca
gttcatgtacggctccaaggcctacgtgaagcaccccgccgacatccccgactacttgaagctgtccttccccgagggcttcaagtgggagcgcgtgat
gaacttcgaggacggcggcgtggtgaccgtgacccaggactcctccctgcaggacggcgagttcatctacaaggtgaagctgcgcggcaccaacttc
ccctccgacggccccgtaatgcagaagaagaccatgggctgggaggcctcctccgagcggatgtaccccgaggacggcgccctgaagggcgagatc
aagcagaggctgaagctgaaggacggcggccactacgacgctgaggtcaagaccacctacaaggccaagaagcccgtgcagctgcccggcgccta
caacgtcaacatcaagttggacatcacctcccacaacgaggactacaccatcgtggaacagtacgaacgcgccgagggccgccactccaccggcgg
catggacgagctgtacaagCATATGtaaGCGGCCgtacctttaagaccaatgacttacaaggcagctgtagatcttagccactttttaaaagaaa
aggggggactggaagggctaattcactcccaacgaagacaagatctgctttttgcttgtactgggtctctctggttagaccagatctgagcctgggagc
tctctggctaactagggaacccactgcttaagcctcaataaagcttgccttgagtgcttcaagtagtgtgtgcccgtctgttgtgtgactctggtaactag
agatccctcagacccttttagtcagtgtggaaaatctctagcagcatctagaattaattccgtgtattctatag

SunTag$_{24x}$-mCherry

ATGGGCGCCATCATACACGCGGCCGCAATCATcggtccgGGTGGATCTGGAGGTGGAGGTTCTGGAGGAGAAGAA
CTTTTGAGCAAGAATTATCATCTTGAGAACGAAGTGGCTCGTCTTAAGAAAGGTTCTGGCAGTGGAGAAGAACTG
CTTTCAAAGAATTACCACCTGGAAAATGAGGTAGCTAGACTGAAAAAGGGGAGCGGAAGTGGGGAGGAGTTGCT
GAGCAAAAATTATCATTTGGAGAACGAAGTAGCACGACTAAAGAAAGGGTCCGGATCGGGTGAGGAGTTACTCT
CGAAAAATTATCATCTCGAAAACGAAGTGGCTCGGCTAAAAAAGGGCAGTGGTTCTGGAGAAGAGCTATTATCTA
AAAACTACCACCTCGAAAATGAGGTGGCACGCTTAAAAAAGGGAAGTGGCAGTGGTGAAGAGCTACTATCCAAG
AATTATCATCTTGAGAACGAGGTAGCGCGTTTGAAGAAGGGTTCCGGCTCAGGAGAGGAACTGCTCTCGAAGAAC
TATCATCTTGAAAATGAGGTCGCTCGATTAAAAAAGGGATCGGGCAGTGGTGAGGAACTACTTTCAAAGAATTAC
CACCTCGAAAACGAAGTAGCTCGATTAAAGAAAGGTTCAGGGTCGGGTGAAGAATTACTGAGTAAAAATTATCAT
CTGGAAAATGAGGTAGCGAGACTAAAAAAGGGGAGTGGTTCTGGCGAAGAGTTGCTATCGAAAAATTATCATCTT
GAGAACGAAGTTGCTAGGCTCAAAAAGGGCTCAGGCTCAGGCGAGGAGTTGCTCTCGAAAAACTACCACTTGGA
AAATGAGGTCGCGAGGTTGAAAAAGGGGAGCGGGTCGGGCGAGGAGTTATTGAGCAAAAACTATCATTTAGAG
AACGAAGTCGCGCGCTTAAAGAAAGGCTCGGGCTCGGGCGAAGAACTCTTATCGAAGAACTACCACCTCGAAAAT
GAGGTCGCCAGGTTGAAAAAGGGCAGTGGCAGCGGGGAGGAACTCTTGAGCAAGAACTACCACTTGGAGAATG
AGGTCGCGAGATTGAAGAAAGGGTCGGGGAGCGGCGAGGAATTGCTCAGCAAGAATTATCATTTGGAGAACGA
AGTCGCCAGGCTCAAGAAAGGCTCGGGGTCGGGGGAGGAATTGTTGAGTAAAAACTACCACTTGGAAAATGAAG
TCGCCAGGCTCAAAAAAGGGAGTGGGAGCGGCGAAGAGTTATTGAGCAAAAATTACCACTTGGAGAACGAAGTG
GCAAGGCTCAAGAAAGGGAGCGGCAGCGGGGAGGAGCTCTTATCGAAGAACTACCACTTAGAGAATGAAGTCGC
CCGCTTGAAGAAAGGCTCGGGGAGCGGGGAAGAGCTCTTGAGCAAGAACTACCACTTGGAAAATGAGGTGGCGC
GCTTGAAGAAAGGGAGCGGGAGCGGGGAAGAGTTACTATCTAAGAATTATCATCTCGAGAACGAGGTGGCTCGA
CTAAAGAAGGGCTCCGGCAGTGGGGAGGAACTCCTGTCGAAGAACTATCATCTTGAAAATGAGGTTGCAAGACTT
AAAAAGGGGTCCGGATCAGGTGAGGAACTACTCAGTAAGAATTACCACCTGGAAAACGAAGTTGCACGTTTGAA
GAAAGGATCAGGATCAGGCGAAGAACTGCTCTCAAAAGATTATCATTTGGAAAATGAGGTTGCACGTTTAAAAAA
GGGAAGTGGCAGTGGTGAGGAACTTCTGTCGAAAAATTATCATCTCGAGAATGAAGTAGCCCGACTTAAAAAGG
GTTCTGGCTCGGGTCAGCGGCCGCACCGGTCAGTGGAGGAGAACCCTGGACCTCCCAAGAAGAAGCGCAAGGTG
GGAGGAGGCGGAACTAGTgtgagcaagggcgaggaggataacatggccatcatcaaggagttcatgcgcttcaaggtgcacatggagggc
tccgtgaacggccacgagttcgagatcgagggcgagggcgagggccgcccctacgagggcacccagaccgccaagctgaaggtgaccaagggtgg
cccccctgcccttcgcctgggacatcctgtcccctcagttcatgtacggctccaaggcctacgtgaagcaccccgccgacatccccgactacttgaagctg

tccttccccgagggcttcaagtgggagcgcgtgatgaacttcgaggacggcggcgtggtgaccgtgacccaggactcctccctgcaggacggcgagtt
catctacaaggtgaagctgcgcggcaccaacttcccctccgacggccccgtaatgcagaagaagaccatgggctgggaggcctcctccgagcggatg
taccccgaggacggcgccctgaagggcgagatcaagcagaggctgaagctgaaggacggcggccactacgacgctgaggtcaagaccacctacaa
ggccaagaagcccgtgcagctgcccggcgcctacaacgtcaacatcaagttggacatcacctcccacaacgaggactacaccatcgtggaacagtac
gaacgcgccgagggccgccactccaccggcggcatggacgagctgtacaagTGA

sfGFP-NLS-$PP7_{24x}$
ATGAGCAAAGGAGAAGAACTTTTCACTGGAGTTGTCCCAATTCTTGTTGAATTAGATGGTGATGTTAATGGGCACA
AATTTTCTGTCCGTGGAGAGGGTGAAGGTGATGCTACAAACGGAAAACTCACCCTTAAATTTATTTGCACTACTGG
AAAACTACCTGTTCCGTGGCCAACACTTGTCACTACTCTGACCTATGGTGTTCAATGCTTTTCCCGTTATCCGGATCA
CATGAAACGGCATGACTTTTTCAAGAGTGCCATGCCCGAAGGTTATGTACAGGAACGCACTATATCTTTCAAAGAT
GACGGGACCTACAAGACGCGTGCTGAAGTCAAGTTTGAAGGTGATACCCTTGTTAATCGTATCGAGTTAAAGGGT
ATTGATTTTAAAGAAGATGGAAACATTCTTGGACACAAACTCGAGTACAACTTTAACTCACACAATGTATACATCAC
GGCAGACAAACAAAAGAATGGAATCAAAGCTAACTTCAAAATTCGCCACAACGTTGAAGATGGTTCCGTTCAACT
AGCAGACCATTATCAACAAAATACTCCAATTGGCGATGGCCCTGTCCTTTTACCAGACAACCATTACCTGTCGACAC
AATCTGTCCTTTCGAAAGATCCCAACGAAAAGCGTGACCACATGGTCCTTCTTGAGTTTGTAACTGCTGCTGGGATT
ACACATGGCATGGATGAGCTCTACAAACCCAAGAAGAAGCGCAAGGTGGATATCTAACGACACGATTCGAAGATC
GCATCGCTAGCCCGGTTGTACAGGTACCGGATCCTAAGGTACCTAATTGCCTAGAAAGGAGCAGACGATATGGCG
TCGCTCCCTGCAGGTCGACTCTAGAAACCAGCAGAGCATATGGGCTCGCTGGCTGCAGTATTCCCGGGTTCATTAG
ATCCTAAGGTACCTAATTGCCTAGAAAGGAGCAGACGATATGGCGTCGCTCCCTGCAGGTCGACTCTAGAAACCA
GCAGAGCATATGGGCTCGCTGGCTGCAGTATTCCCGGGTTCATTAGATCCTAAGGTACCTAATTGCCTAGAAAGG
AGCAGACGATATGGCGTCGCTCCCTGCAGGTCGACTCTAGAAACCAGCAGAGCATATGGGCTCGCTGGCTGCAGT
ATTCCCGGGTTCATTAGATCCTAAGGTACCTAATTGCCTAGAAAGGAGCAGACGATATGGCGTCGCTCCCTGCAGG
TCGACTCTAGAAACCAGCAGAGCATATGGGCTCGCTGGCTGCAGTATTCCCGGGTTCATTAGATCCTAAGGTACCT
AATTGCCTAGAAAGGAGCAGACGATATGGCGTCGCTCCCTGCAGGTCGACTCTAGAAACCAGCAGAGCATATGGG
CTCGCTGGCTGCAGTATTCCCGGGTTCATTAGATCCTAAGGTACCTAATTGCCTAGAAAGGAGCAGACGATATGGC
GTCGCTCCCTGCAGGTCGACTCTAGAAACCAGCAGAGCATATGGGCTCGCTGGCTGCAGTATTCCCGGGTTCATTA
GATCCTAAGGTACCTAATTGCCTAGAAAGGAGCAGACGATATGGCGTCGCTCCCTGCAGGTCGACTCTAGAAACC
AGCAGAGCATATGGGCTCGCTGGCTGCAGTATTCCCGGGTTCATTAGATCCTAAGGTACCTAATTGCCTAGAAAG
GAGCAGACGATATGGCGTCGCTCCCTGCAGGTCGACTCTAGAAACCAGCAGAGCATATGGGCTCGCTGGCTGCAG
TATTCCCGGGTTCATTAGATCCTAAGGTACCTAATTGCCTAGAAAGGAGCAGACGATATGGCGTCGCTCCCTGCAG
GTCGACTCTAGAAACCAGCAGAGCATATGGGCTCGCTGGCTGCAGTATTCCCGGGTTCATTAGATCCTAAGGTACC
TAATTGCCTAGAAAGGAGCAGACGATATGGCGTCGCTCCCTGCAGGTCGACTCTAGAAACCAGCAGAGCATATGG
GCTCGCTGGCTGCAGTATTCCCGGGTTCATTAGATCCTAAGGTACCTAATTGCCTAGAAAGGAGCAGACGATATG
GCGTCGCTCCCTGCAGGTCGACTCTAGAAACCAGCAGAGCATATGGGCTCGCTGGCTGCAGTATTCCCGGGTTCAT
TAGATCCTAAGGTACCTAATTGCCTAGAAAGGAGCAGACGATATGGCGTCGCTCCCTGCAGGTCGACTCTAGAAA
CCAGCAGAGCATATGGGCTCGCTGGCTGCAGTATTCCCGGGTTCATTAGATC

Xbp1 reporter
ATGGGCGCCATCATACACGCGGCCGCAATCATcggtccgGGTGGATCTGGAGGTGGAGGTTCTGGAGGAGAAGAA
CTTTTGAGCAAGAATTATCATCTTGAGAACGAAGTGGCTCGTCTTAAGAAAGGTTCTGGCAGTGGAGAAGAACTG
CTTTCAAAGAATTACCACCTGGAAAATGAGGTAGCTAGACTGAAAAAGGGGAGCGGAAGTGGGGAGGAGTTGCT
GAGCAAAAATTATCATTTGGAGAACGAAGTAGCACGACTAAAGAAAGGGTCCGGATCGGGTGAGGAGTTACTCT
CGAAAAATTATCATCTCGAAAACGAAGTGGCTCGGCTAAAAAAGGGCAGTGGTTCTGGAGAAGAGCTATTATCTA
AAAACTACCACCTCGAAAATGAGGTGGCACGCTTAAAAAAGGGAAGTGGCAGTGGTGAAGAGCTACTATCCAAG
AATTATCATCTTGAGAACGAGGTAGCGCGTTTGAAGAAGGGTTCCGGCTCAGGAGAGGAACTGCTCTCGAAGAAC
TATCATCTTGAAAATGAGGTCGCTCGATTAAAAAAGGGATCGGGCAGTGGTGAGGAACTACTTTCAAAGAATTAC

CACCTCGAAAACGAAGTAGCTCGATTAAAGAAAGGTTCAGGGTCGGGTGAAGAATTACTGAGTAAAAATTATCAT
CTGGAAAATGAGGTAGCGAGACTAAAAAAGGGGAGTGGTTCTGGCGAAGAGTTGCTATCGAAAAATTATCATCTT
GAGAACGAAGTTGCTAGGCTCAAAAAGGGCTCAGGCTCAGGCGAGGAGTTGCTCTCGAAAAACTACCACTTGGA
AAATGAGGTCGCGAGGTTGAAAAAGGGGAGCGGGTCGGGCGAGGAGTTATTGAGCAAAAACTATCATTTAGAG
AACGAAGTCGCGCGCTTAAAGAAAGGCTCGGGCTCGGGCGAAGAACTCTTATCGAAGAACTACCACCTCGAAAAT
GAGGTCGCCAGGTTGAAAAAGGGCAGTGGCAGCGGGGAGGAACTCTTGAGCAAGAACTACCACTTGGAGAATG
AGGTCGCGAGATTGAAGAAAGGGTCGGGGAGCGGCGAGGAATTGCTCAGCAAGAATTATCATTTGGAGAACGA
AGTCGCCAGGCTCAAGAAAGGCTCGGGGTCGGGGGAGGAATTGTTGAGTAAAAACTACCACTTGGAAAATGAAG
TCGCCAGGCTCAAAAAAGGGAGTGGGAGCGGCGAAGAGTTATTGAGCAAAAATTACCACTTGGAGAACGAAGTG
GCAAGGCTCAAGAAAGGGAGCGGCAGCGGGGAGGAGCTCTTATCGAAGAACTACCACTTAGAGAATGAAGTCGC
CCGCTTGAAGAAAGGCTCGGGGAGCGGGGAAGAGCTCTTGAGCAAGAACTACCACTTGGAAAATGAGGTGGCGC
GCTTGAAGAAAGGGAGCGGGAGCGGGGAAGAGTTACTATCTAAGAATTATCATCTCGAGAACGAGGTGGCTCGA
CTAAAGAAGGGCTCCGGCAGTGGGGAGGAACTCCTGTCGAAGAACTATCATCTTGAAAATGAGGTTGCAAGACTT
AAAAAGGGGTCCGGATCAGGTGAGGAACTACTCAGTAAGAATTACCACCTGGAAAACGAAGTTGCACGTTTGAA
GAAAGGATCAGGATCAGGCGAAGAACTGCTCTCAAAAGATTATCATTTGGAAAATGAGGTTGCACGTTTAAAAAA
GGGAAGTGGCAGTGGTGAGGAACTTCTGTCGAAAAATTATCATCTCGAGAATGAAGTAGCCCGACTTAAAAAGG
GTTCTGGCTCGGGTCAGCGGCCGCACCGGTCAGCAGTGGAGGACAGCACGCTGCAAGTAGTGGTACGGGTGCGG
CCCCCCACCCCTCGGGAGCTGGACAGTCAGCGGCGGCCAGTGGTTCAGGTGGTGGACGAGCGGGTGCTGGTGTT
TAACCCTGAGGAGCCCGATGGAGGGTTCCCTGGCCTGAAATGGGGTGGCACCCATGATGGCCCCAAGAAGAAGG
GCAAAGACCTGACGTTTGTCTTTGACCGGGTCTTTGGCGAGGCGGCCACCCAACAGGACGTGTTCCAGCACACCA
CGCACAGCGTCCTGGACAGCTTCCTCCAGGGCTACAACTGCTCAGTGTTTGCCTACGGGGCCACCGGGGCTGGGA
AGACACACACCATGCTGGGAAGGGAGGGGGACCCCGGCATCATGTACCTGACCACCGTGGAACTGTACAGGCGC
CTGGAGGCCCGCCAGCAGGAGAAGCACTTCGAGGTGCTCATCAGCTACCAGGAGGTGTATAATGAACAGATCCAT
GACCTCCTGGAGCCCAAGGGGCCCCTTGCCATCCGCGAGGACCCCGACAAGGGGGTGGTGGTGCAAGGACTTTC
TTTCCACCAGCCAGCCTCAGCCGAGCAGCTGCTGGAGATACTGACCAGGGGGAACCGTAACCGCACGCAGCACCC
CACTGATGCCAACGCGACTTCCTCCCGCTCCCATGCCATCTTCCAGATCTTTGTGAAGCAGCAGGACCGGGTTCCA
GGACTGACCCAGGCTGTCCAGGTGGCCAAGATGAGCCTGATTGACCTGGCTGGCTCAGAGCGGGCATCCAGCAC
CCATGCGAAGGGGGAGCGGCTGCGGGAGGGGGCCAACATCAACCGCTCTCTGCTGGCGCTCATCAACGTCCTCA
ATGCCTTGGCCGATGCAAAGGGCCGCAAGACCGCTGTGCCCTACGCGGACAGCGCACTGACCCGCCTGCTCAAAG
ACTCCCTCGGGGGCAACTGCCGCACAGTGATGATCGCTGCCATCAGCCCCTCCAGCCTGACCTACGAGGACACGT
ATAATACCCTCAAATATGCCGACCGGGCCAAGGAGATCAGGCTCTCGCTGAAGAGCAATGTGACCAGCCTGGACT
GTCACATCAGCCAGTATGCTACCATCTGCCAACAGCTCCAGGCTGAGGTAGCCGCTCTGAGGAAGAAGCTCCAAG
TGTATGAGGGGGGAGGCCAGCCCCCACCACAGGACCTCCCAGGATCTCCCAAGTCGGGACCACCACCAGAACACC
TTCCCAGCTCCCCCTTGCCACCCCACCCTCCCAGCCAGCCCTGCACCCCAGAGCTCCCTGCAGGGCCTAGAGCCCTT
CAAGAGGAGAGTCTGGGGATGGAGGCCCAGGTGGAGAGGGCCATGGAAGGGAACTCTTCAGACCAGGAGCAGT
CCCCAGAGGATGAGGATGAAGGCCCAGCTGAGGAGGTTCCAACCCAGATGCCAGAGCAGAACCCCACACATGCA
CTGCCAGAGTCCCCTCGCCTGACCCTGCAGCCCAAGCCAGTCGTGGGCCACTTCTCAGCACGGGAACTGGATGGG
GACCGTTCTAAGCGGTTGGCCCTAAAGGTGCTGTGCGTTGCCCAGCGGCAGTACTCCCTGCTCCAAGCAGCCAACC
TCCTGACGCCCGACATGATCACAGAGTTTGAGACCCTACAGCAGCTGGTGCAAGAGGAAAAAATTGAGCCTGGGG
CAGAGGCCTTGAGGACTTCAGGCCTGGCCAGGGGGGCACCTCTGGCTCAGGAGCTGTGTTCAGAGTCAAAGCCTC
CAGGATACACTGGCCCTGTGACCCGGACTATGGCGAGGCGACTGAGTGGCCCCCTGCACACCCTGGGAATCCCGC
CTGGACCCAACTGCACCCCAGCCCAGGGGTCCCGATGGCCCATGGAGAAGAAGAGGAGGAGACCAAGCGCCTTG
GAGGCAGACAGTCCCATGGCCCCAAAGCGGGGCACCAAGCGCCAGCGCCAGTCCTTCCTGCCCTGCCTAAGGAGA
GGGTCTCTGCCTGACACCCAACCTTCACAGGGGCCCAGCACCCCCAAAGGAGAAAGGGCCTCCTCCCCCTGCCATT
CCCCTCGCGTTTGCCCAGCCACAGTCATCAAAAGCCGGGTGCCCCTGGGCCCTTCCGCCATGCAGAACTGCTCCAC
CCCGCTGGCTCTGCCCACTCGAGACCTCAATGCCACCTTTGATCTCTCTGAGGAGCCTCCCTCAAAGCCCAGTTTCC
ATGAATGCATTGGCTGGGACAAAATACCCCAGGAGCTGAGCAGGCTGGACCAGCCCTTCATCCCCAGGGCACCTG
TGCCCCTGTTCACCATGAAGGGCCCCAAGCCAACATCTTCCCTCCCTGGGACCTCTGCCTGCAAGAAGAAGCGCGT

TGCGAGTTCCTCAGTCTCCCATGGCCGCAGCCGCATCGCCCGCCTCCCCAGCAGCACTTTGAAGAGGCCAGCTGGG
CCCCTTGTACTCCCAGAGCTGCCCTTGAGTCCCCTGTGCCCTAGCAACCGGAGGAATGGAAAGGACCTCATCAGG
GTGGGGAGAGCACTCTCAGCAGGGAACGGCGTCACCAAGGTGTCCGATAAGGACCCCGTGCCCTACCAACCTCCA
TTCCTGTGTCAATGGGGCCGACATCAGCCAGCCTGGAAACCCTTGATGAATGGCGGCGGCGCCGCCAGCGCCAAA
GCCATCTAA
